# Self-assembled micro-computed tomography for dental education

**DOI:** 10.1371/journal.pone.0209698

**Published:** 2018-12-26

**Authors:** Che-Wei Liao, Lih-Jyh Fuh, Yen-Wen Shen, Heng-Li Huang, Chih-Wei Kuo, Ming-Tzu Tsai, Jui-Ting Hsu

**Affiliations:** 1 Graduate Institute of Biomedical Sciences, China Medical University, Taichung, Taiwan, ROC; 2 School of Dentistry, College of Dentistry, China Medical University, Taichung, Taiwan, ROC; 3 Department of Dentistry, China Medical University and Hospital, Taichung, Taiwan, ROC; 4 Department of Bioinformatics and Medical Engineering, Asia University, Taichung, Taiwan, ROC; 5 Materials & Electro-Optics Research Division, National Chung-Shan Institute of Science & Technology, Taoyuan City, Taiwan, ROC; 6 Department of Biomedical Engineering Hungkuang University, Taichung, Taiwan, ROC; National Taiwan University, School of Dentistry, TAIWAN

## Abstract

This study used available or purchased equipment and an image reconstruction system developed by the college of dentistry to establish a basic self-assembled micro-computed tomography (micro-CT) system. Such a system would be suitable for teaching dental radiology to dental students. Specifically, it could help students to understand the principles governing dental cone-beam computed tomography (CBCT) and provide graduate students with a system for scanning small samples (e.g., individual teeth) during the early stages of research. The self-assembled micro-CT system was constructed using a portable dental X-ray tube, an intraoral digital X-ray detector, a high-precision rotation stage, related bracket accessories, and a notebook computer. Reconstructed images and three-dimensional models of the maxillary right third molar were produced using the self-assembled micro-CT system and an advanced commercially available micro-CT system (Skyscan 2211). Subsequently, the reconstructed images and 3D models produced using the two systems were compared by two senior dentists to determine whether considerable visual differences could be observed. Finally, the signal-to-noise ratio (SNR) was used for quantitative analysis and to compare the systems. Although the self-assembled micro-CT system produced image boundaries that were not as sharp as those of Skyscan 2211, the images were nonetheless remarkably similar. In addition, the two micro-CT systems produced 3D models that were almost identical in appearance and root canal shape. Quantitative analysis revealed that Skyscan 2211 had produced a SNR that was superior to that of the self-assembled micro-CT system, with the difference ranging from 36.77% to 136.22%; enamel, which has a higher density, exhibited lower SNR differences, whereas dentin, which has a lower density, exhibited higher SNR differences. The self-assembled micro-CT system with a resolution of 36 μm was created using a portable dental X-ray tube and an intraoral digital X-ray detector. Although the scanning time was relatively long (~30 min to scan images of a tooth), the images were adequate in the preliminary stage of experiments. More importantly, students were afforded the opportunity to observe the process of assembling and disassembling each component of a micro-CT scanner and thereby achieve a more comprehensive understanding of the principles governing micro-CT and dental CBCT.

## Introduction

Micro computed tomography (micro-CT) is a noninvasive detection tool that employs radiation and digital X-ray detectors to capture images of a sample’s internal structure (on a micro level) without damaging the sample [1]. In general, a resolution of less than 50 μm is considered micro-CT. Currently, a resolution of 10–30 μm is commonly used to scan small samples (e.g., a single tooth or animal bone) [2–4]. A micro-CT system contains the following primary components: an X-ray tube, an intraoral digital X-ray detector, an electric rotation stage, a computer, and lead shielding [5]. Micro-CT scans can be divided into two major types: in vivo and ex vivo scans. In vivo scans involve placing an object on a stationary platform with an X-ray tube and digital X-ray detector rotating around the object. Ex vivo scans entail placing an object on a rotating platform with the X-ray tube and digital X-ray detector in fixed positions. Even the most basic micro-CT scanners cost at least US$100,000, and advanced micro-CT scanners can cost as much as US$1,000,000. Micro-CT mostly employs cone-beam X-rays and microfocal spot tubes. Digital X-ray detectors are primarily used to quickly receive two-dimensional (2D) digital X-ray images created during the scanning process. These images are stored in a computer for processing after all the scanning images have been taken. For ex vivo micro-CT, an electric rotation stage is used to precisely control the amount of sample rotation for the X-ray tube and digital X-ray detector to scan the sample [6]. A computer is used to control the overall motion of the micro-CT scanner and reconstruct the three-dimensional (3D) images (i.e., the final images), and lead shielding is used as protection preventing the X-rays from harming operators during the scanning process.

Many studies have examined how to construct micro-CT systems [6]. However, because microfocal spot X-ray tubes and X-ray detectors are expensive (approximately US$10,000 and US$30,000, respectively) and because such scanners are relatively complex [7], they are generally not used in experiments conducted during courses offered at related university departments (e.g., the departments of medical imaging, radiological sciences, and biomedical engineering). Consequently, most students studying courses offered by these departments only learn about micro-CT or CT hardware components from teaching materials or slideshows provided by their teachers, which means they are unable to physically assemble such hardware for themselves. Micro-CT is similar to dental cone-beam computed tomography (CBCT) (widely used by the dental industry) in both principle and hardware composition; that is, they both employ cone beams to scan and form images [8]. CBCT technology has become more advanced and has increased in popularity in clinical settings. Currently, dental students studying CBCT on dental radiography courses–can only acquire knowledge of the equipment from teaching materials or slideshows provided by their teachers. Most dental students are not granted the opportunity to assemble or disassemble CBCT equipment [9].

Therefore, providing students studying in colleges of dentistry with an opportunity to assemble micro-CT, CT, or CBCT hardware can facilitate a deeper understand of the principles governing such hardware and their scanning methods. This could be helpful for dental students; because of they may need to purchase a dental CBCT machine for their clinics after they graduate from dental school. Micro-CT systems consist of two main components: X-ray tubes and intraoral digital X-ray detectors. This study used equipment owned and funded by the related university departments to establish a basic self-assembled micro-CT system. Such a system could be used by students in dental radiology courses to improve their understanding of the principles governing such systems.

## Materials and methods

### System description and scanner geometry

**Fig 1:**
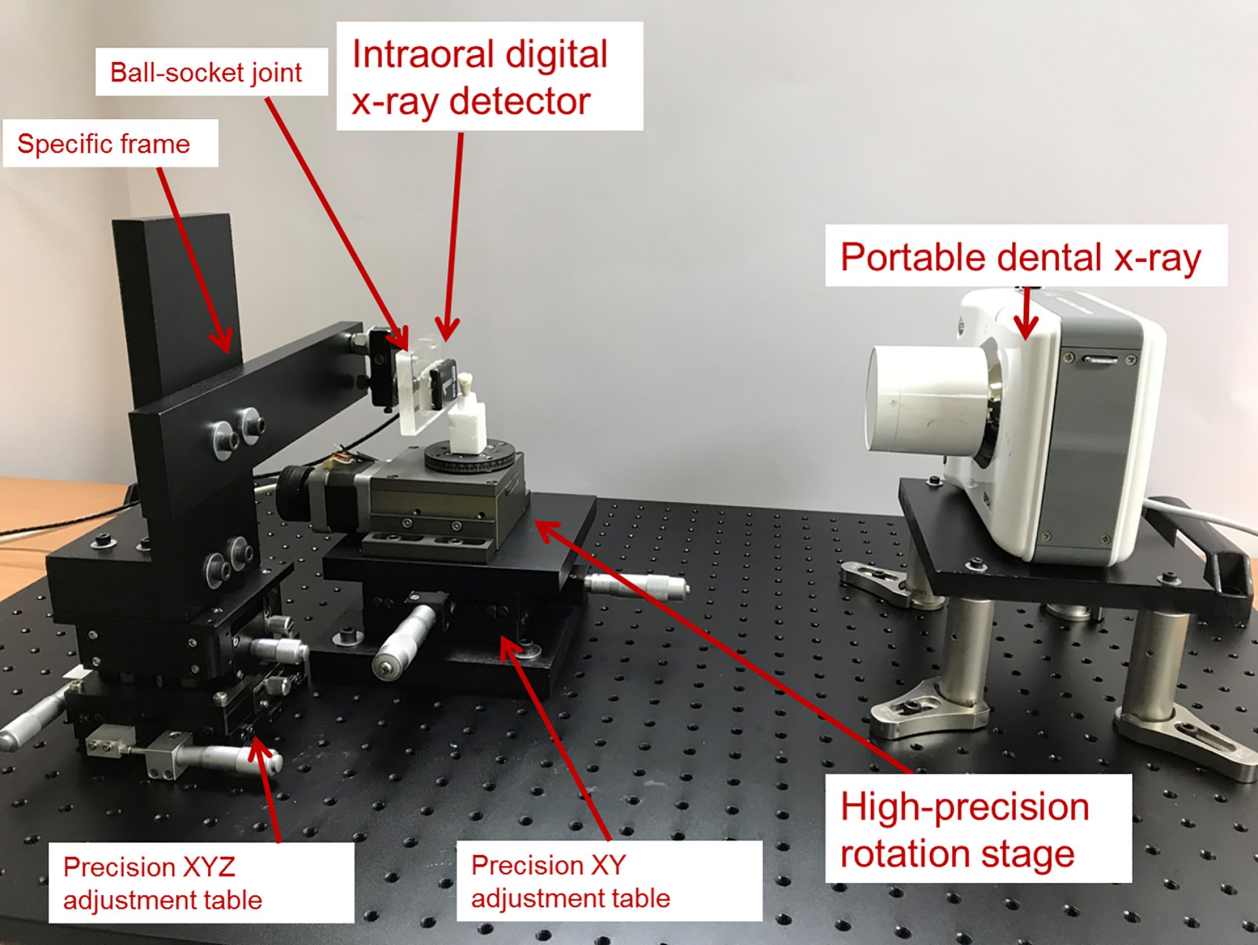
Main components of the experimental micro-CT prototype.

The self-assembled micro-CT system contained the following components: a portable dental X-ray tube, an intraoral digital X-ray detector, a high-precision rotation stage, a computer, lead shielding, and a micro-motion platform (Fig 1). The specifications of the components are as follows: the X-ray tube model was a BPD-I (BEMEMS, Seoul, South Korea) with a tube voltage of 60 kV, a tube current of 2 mA, an exposure time of 0.01–2.00 s, and a focal spot size of 0.8 mm; the X-ray tube was fixed to an optical platform. The digital X-ray detector model was RVG6200-SIZE1 (Carestream Dental, Stuttgart, Germany) with a field of view of 22.2 × 29.6 mm^2^ and a pixel size of 20.83 × 20.83 μm^2^ (24 lp/mm); the digital X-ray detector was connected and fixed to a precision XYZ adjustment table on the optical board using brackets. The rotation stage model was MR-60G (Tanlian E-O Co., Ltd., Taoyuan, Taiwan) with a maximum load of 50 N, a minimum rotational resolution of 0.005°, and a maximum rotational speed of 5 rotations/s; the rotation stage was fixed to a precision XY adjustment table on the optical board.

**Fig 2:**
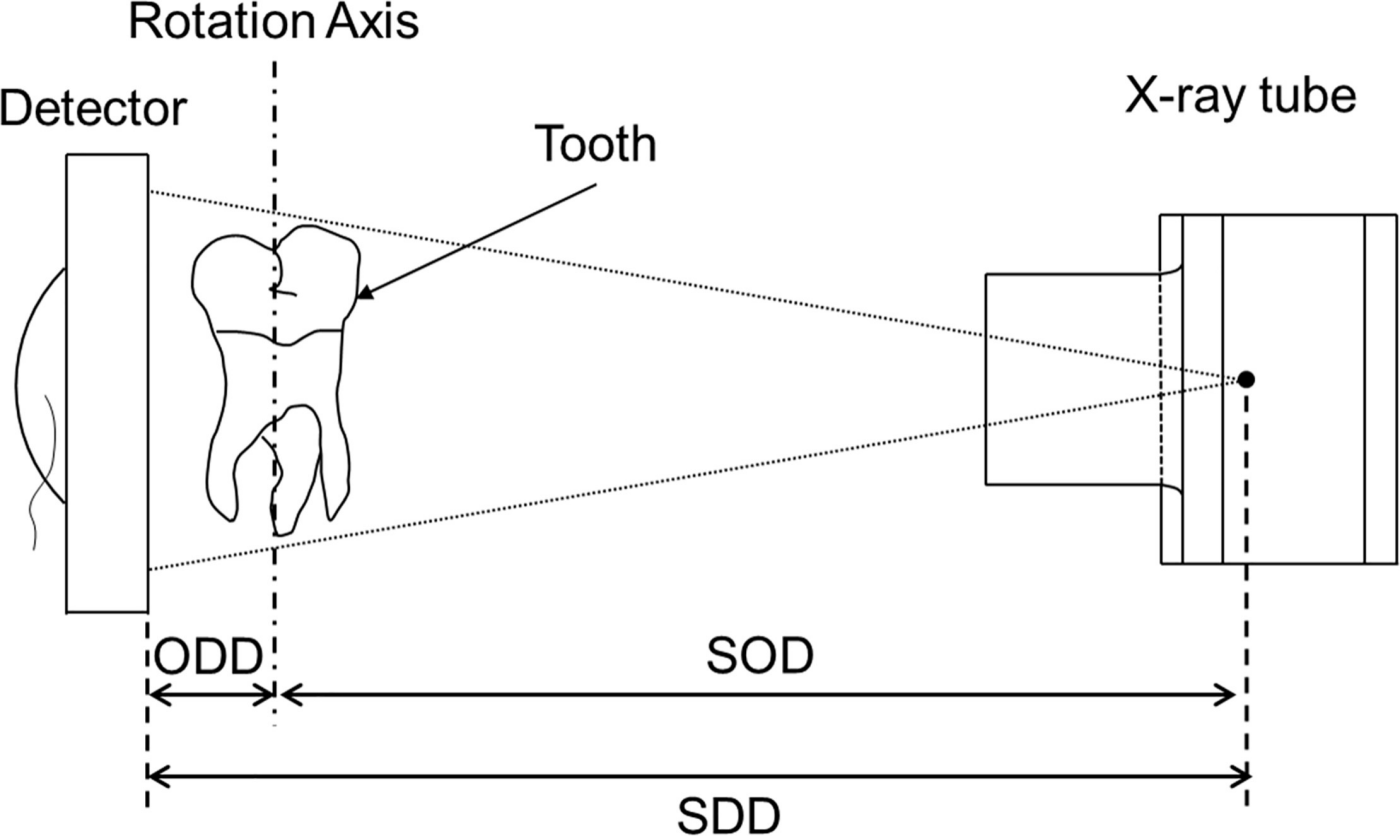
Geometry of the micro-CT system. The diagram shows the design used in the experiments. (SOD, source–object distance; ODD, object–detector distance; SDD, source–detector distance).

The micro-CT hardware contained an X-ray tube on one side and a digital X-ray detector on the other side; the sample was placed on a high-precision rotation stage (controlled by a computer) between the two components (Fig 2). The source–object distance (SOD) and source–detector distance (SDD) were set to 361 and 375 mm, respectively.

The magnification factor of the 2D projection images produced using micro-CT was denoted by M.

$$M=\frac{SDD}{SOD}=\frac{375}{361}=1.03878$$

In theory, the spatial resolution of micro-CT is influenced by two factors: the focal spot size of the X-ray tube (ρf) and the focal spot size of the digital X-ray detector (ρd).

The resolution of the digital X-ray detector (θd) was calculated using the following equation:

$$\theta d=\frac{\rho d}{M}=\frac{20.83333}{1.03878}=20.05556$$

Because the focal spot size of the X-ray tube at the center of the field of view had a resolution of (θf), was calculated using the following equation:

$$\theta f=\theta f\times \frac{Ml}{M}=800\times \frac{1.03878te}{1.03878}=29.86667$$

Finally, by using the Gaussian distributions for θf and θd, the spatial resolution for the reconstructed micro-CT images can be calculated using the following equation:

$$\theta =\sqrt{\theta f^{2}+\theta d^{2}}=\sqrt{29.86667^{2}+20.05556^{2}}=35.97559$$

### Tooth scanning and image reconstruction

The self-assembled micro-CT system and an advanced commercially available micro-CT scanner (Skyscan 2211; Bruker, Kontich, Belgium) were used to separately scan the same maxillary right third molar. The quality of the scanned images was then compared. The scanning parameters of the self-assembled micro-CT system were as follows: the sample was rotated and scanned at increments of 1°, with the rotation angle precisely controlled by the high-precision rotation stage. After every rotation, the sample was stationary, and the X-ray tube was activated for a single exposure. The X-ray tube was set to adult mode and a wisdom tooth was scanned. The X-ray tube voltage and current were set to 60 kV and 2 mA, respectively, and the exposure time was 0.70 s. During exposure, the digital X-ray detector received captured images. The images had to be manually saved one at a time, and the data format used was TIF. To enable comparison between images scanned using the two micro-CT systems at the same resolution, the Skyscan 2211 was also used with a resolution of 36 μm and the sample was rotated and scanned the sample at increments of 1°. The tube voltage and current for the Skyscan 2211 were 120 kV and 280 μA, respectively. To eliminate the effect of using a reconstructed image calculation method on reconstructed images, the 2D projection images scanned using the two micro-CT systems were reconstructed using NRecon software (Bruker, Kontich, Belgium). For the images created using the Skyscan 2211, the ring artifact correction and beam hardening correction were set to 5% and 10%, respectively, during the image reconstruction process. By contrast, for the images captured using the self-assembled micro-CT system, the ring artifact correction and beam hardening correction were both set to 0%. Then, CTVox software (Bruker, Kontich, Belgium) was used for 3D reconstruction. All experimental procedures were executed with the ethical approval of the Institutional Review Board of China Medical University Hospital. (CMUH 107-REC3-092)

### Comparison of micro-CT images captured using the commercial and self-assembled scanners

The quality of the images taken using the two micro-CT scanners was compared. Both quantitative and qualitative comparisons were performed. For qualitative comparison, the images taken with the Skyscan 221 were set as the standard. Experienced image readers were asked to examine the images to compare their quality and observe the original sample to determine whether the image profiles presented by the two scanners matched that of the sample. Quantitative image quality evaluation involves examining the signal-to-noise ratio (SNR). The SNR is the ratio of signal strength to background noise. A high SNR indicates favorable image quality. All the SNRs of the reconstructed images in this study were calculated using ImageJ software version 1.52a (Rasband, W.S., US National Institutes of Health, Bethesda, MD, USA). Three images were obtained from both the Skyscan 2211 and self-assembled micro-CT system at the same position in the buccal–lingual sections. Two regions of interest (ROIs) were selected on each image for measuring the SNR of enamel and dentin.

The equation for calculating SNR is $SNR=\frac{{\vec{f}}^{*}}{\sigma_{\vec{f}}}$,

where ${\vec{f}}^{*}$ indicates the mean of the grayscale values of the ROI and $\sigma_{\vec{f}}$ denotes the standard deviation of the grayscale values of the ROI.

## Results

**Fig 3:**
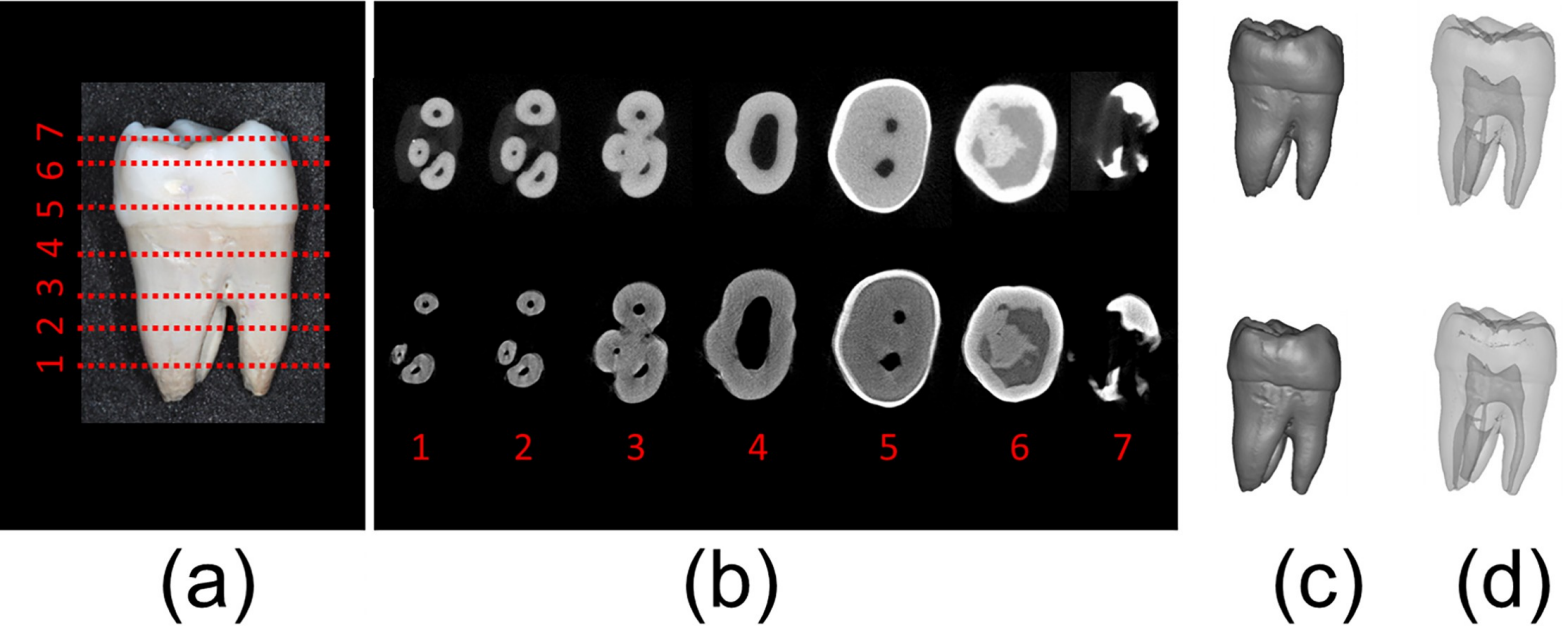
(a) Third molar used in this experiment; (b) reconstruction images from (top) the Skyscan 2211 and (bottom) the self-assembled micro-CT; (c) 3D surface model established from (top) the Skyscan 2211 and (bottom) the self-assembled micro-CT; and (d) translucent model established from (top) the Skyscan 2211 and (bottom) the self-assembled micro-CT.

Fig 3A displays a picture of an actual maxillary right third molar. A total of seven reconstructed images at different axial heights were selected for comparison. The top and bottom rows in Fig 3B display the images taken with the Skyscan 2211 and self-assembled micro-CT system, respectively. These images reveal that although the edges of the images taken with the self-assembled micro-CT system were not as sharp in the edge as those in the images taken with the Skyscan 2211, the images were nonetheless similar. The 3D models constructed from the images taken with the two scanners (Fig 3C) exhibit no clear differences to the naked eye. Two senior dentists with over 20 years of experience, Dr. L. J. Fuh and Dr. Y. W. Shen, admitted that without prompting they could not distinguish the models through comparison of the 3D models’ appearances with the actual tooth. In addition, the root canal shapes inside the tooth revealed that the images were also almost identical.

**Fig 4:**
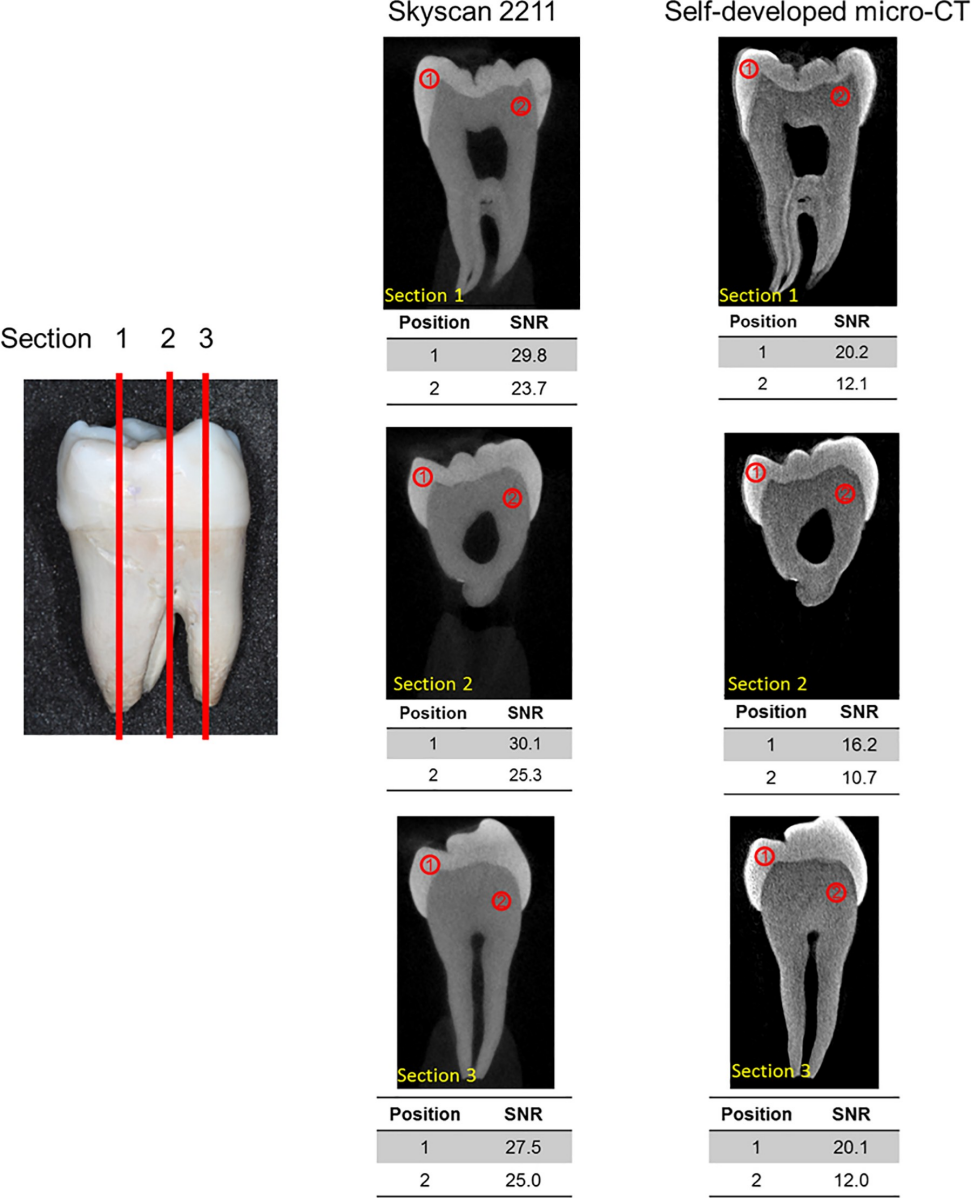
Quantitative analysis of the three buccal-lingual reconstructed images of the third molar captured with (left) the Skyscan 2211 and (right) the self-assembled micro-CT system. SNR, signal-to-noise ratio.

The results of quantitative image analysis of the image SNRs are shown in Fig 4. In this experiment, the locations of enamel and dentin in the tooth were measured at three cross-sections. SNRs of the images captured with the Skyscan 2211 were all higher than those captured with the self-assembled micro-CT system. The differences in the SNRs of enamel and dentin ranged between 36.8% and 55.9% and between 95.3% and 136.2%, respectively.

## Discussion

Dental CBCT has been widely used in clinic dentistry. Therefore, improving students’ knowledge of CBCT before they graduate from dental school is crucial. In brief, the reconstruction theories and major components of dental CBCT and micro-CT are highly similar. Although various studies have proposed self-assembled micro-CT scanners, the equipment involved in such proposals has been high-end and expensive. This study employed existing materials and funding available at most colleges of dentistry or departments of oral medicine—namely a portable dental X-ray tube, a intraoral digital X-ray detector, and US$2,500—to produce a self-assembled micro-CT scanner with an image resolution of 36 μm. Although the quantitative quality of the image produced by the self-assembled micro-CT scanner was inferior to that of a commercially available product, senior dentists deemed the results acceptable to the naked eye and suitable for use in early stage analysis or when a few micro-CT images are required for related studies. More importantly, this method could be used to provide dental students with the opportunity to assemble a micro-CT scanner, thereby developing their experience with operating such hardware and improving their understanding regarding the principles governing micro-CT and CBCT.

Currently, basic knowledge of dental radiology is taught using textbook materials or multimedia tools explaining radiation devices for capturing medical images. Consequently, students rarely have opportunities to interact with real devices or assemble such devices themselves. However, assembling a functioning micro-CT device for imaging could be achieved with a smaller budget. Constructing such a device with students can greatly benefit their learning and leave them a lasting impression. Additionally, constructing such a system requires a thorough understanding of each part in terms of the image reconstruction theories underlying micro-CT, CT, and dental CBCT. An understanding of hardware components, the principles of operation, and the image processing of a micro-CT unit is helpful for dental students because they will likely be required to purchase a dental CBCT machine for their clinics after graduating from dental school and will therefore be responsible for its operation.

Micro-CT also plays an increasingly critical role in medical research. Because it uses a nondestructive method to reveal the internal structure of the test samples, micro-CT is especially useful for dentistry or orthopedic research. In dentistry, for example, micro-CT can be employed as an expository tool for obtaining a tooth’s profile and root canal shape [10–12]. In dental implant surgery, micro-CT can be employed to obtain microstructure parameters when trephine is used to access the bone at the dental implant site [13]. In orthopedics, micro-CT is the standard method for evaluating the parameters of trabecular bone microstructures [14–17]. Therefore, many academic institutes have purchased micro-CT scanners as basic research devices.

Commercial micro-CT scanners are expensive [2]. Micro-CT scanners with basic functions cost approximately US$100,000, and high-end micro-CT scanners can cost as much as US$1 million. However, this study demonstrated that a basic micro-CT scanner can be self-assembled in colleges of dentistry and related departments by using existing teaching equipment, namely a portable dental X-ray tube and intraoral digital X-ray detector. An additional US$2,500 was needed for additional items, namely a high-precision rotation stage (approximately US$1,500), optical breadboards (approximately US$400), an XYX axis, and an XY axis precision fine-tuning table (approximately US$600). Free software such as NRecon, PITRE, H-PITRE, and Athabasca Recon are available to conduct back projection on 2D projection images for image reconstruction [18]. Many open access image reconstruction codes in Matlab or C are available online. Therefore, once the hardware for obtaining 2D projection images is available, image reconstruction can be accomplished using free resources.

After 2D projection images were captured from 360° around the sample at a resolution of 36 μm with both the self-assembled micro-CT system and Skyscan 2211, the images were reconstructed and compared. Observation with the naked eye revealed that the overall image resolution of the reconstructed Skyscan 2211 images was superior to that of the self-assembled micro-CT system, and the boundaries between materials of different densities were also more clearly defined. The higher quality of the Skyscan 2211 images may have been a result of misalignment of the self-assembled micro-CT system, although the relative positions of the hardware components were fine-tuned prior to taking the images. Nevertheless, from a macroscopic view, the two micro-CT scanners demonstrated identification ability for tissues of different densities. Both the profile structure and structure of the inner tissues were clearly identifiable in pictures taken using both scanners. Two senior dentists also determined that the image quality from both scanners was sufficient for identifying the profile of a tooth and internal root canal shape.

Quantitative comparisons of the image quality were also conducted. The spatial geometric positions at the three layers were identified and were discovered to be identical to the reconstructed images obtained using the self-assembled micro-CT system and Skyscan 2211. ROIs at those identical positions on the enamel and dentin were utilized to conduct quantitative calculation of the image SNRs. Using quantitative data, image quality was compared in depth. Although the image quality of the two did not differ greatly to the naked eye, the images taken using the Skyscan 2211 had higher SNRs, and thus superior image quality. Comparison of the reconstructed images of the two micro-CT systems revealed that whereas the SNRs of enamel ranged between 36.8% and 55.9%, those of dentin ranged between 95.3% and 136.2%. This could be because the tissue density of dentin is relatively low, resulting in a low brightness value on the gray scale of the image, which consequently influenced the SNRs.

This study used an X-ray tube, digital X-ray detector together, and small budget to assemble a low-level micro-CT scanner; however, this scanner had several limitations. First, because the digital X-ray detector adopted in this study was an intraoral digital periapical film, with low receiving and transmitting speeds for X-ray images, a 5-s break was required between each image capture. In addition, the system could not save images automatically after they were taken. Taking a 2D projection image every 1°, resulting in 360 images, required approximately 30 min. This time consumption was far greater than that required by commercially available micro-CT scanners. Second, to prevent the focal spot size of the X-ray tube limiting the spatial resolution, the focal spot size in a typical micro-CT scanner is smaller than 50 μm. However, the X-ray tube used in the present study was the existing device available at the study location, which was a portable dental X-ray tube with a focal spot size of 800 μm. The system constructed in this study utilized an extremely small object-detection distance d to reduce the focal spot size at the cost of low image resolution. Due to limits in the magnification factor and use of an intraoral digital X-ray detector, the sample size of the objects for imaging must be small, approximately 2 cm in diameter or smaller, such as a human tooth or a rat bone. Third, the system used digital vernier calipers and a precision XYZ adjustment table to adjust the relative position of the hardware. However, misalignment of the hardware was still possible, resulting in blurred images. In the future, a two-ring calibration phantom or image feature points for alinement correction to improve reconstructed image quality.

## Conclusion

By using existing equipment in colleges of dentistry or departments of oral medicine specifically, a portable dental X-ray tube, and an intraoral digital X-ray detector, and—with a small budget of US$2,500, a micro-CT scanner with image resolution of 36 μm was assembled. Although objects took longer to image (one tooth taking ~30 min), the images were suitable for early stage analysis or research requiring only a few micro-CT images. Moreover, the process of assembling a micro-CT scanner could be used to provide students in colleges of dentistry with hands-on experience of hardware assembly, helping them further understand the principles of micro-CT imaging. This is helpful for dental students because a more comprehensive understanding of how such a system is assembled and operated will enable students make more informed decisions regarding purchasing a suitable system after they have graduated from dental school.

## Supporting information

S1 File The reconstructed images obtained from Skyscan 2211 micro-CT. (ZIP)

S2 File The reconstructed images obtained from self-assembled micro-CT. (ZIP)
