# Supplementary figures and images for: Self-assembled micro-computed tomography for dental education (part 2 of 4)
Source: PLoS One. 2018 Dec 26;13(12):e0209698. doi: 10.1371/journal.pone.0209698 (PMC6306236; doi:10.1371/journal.pone.0209698)

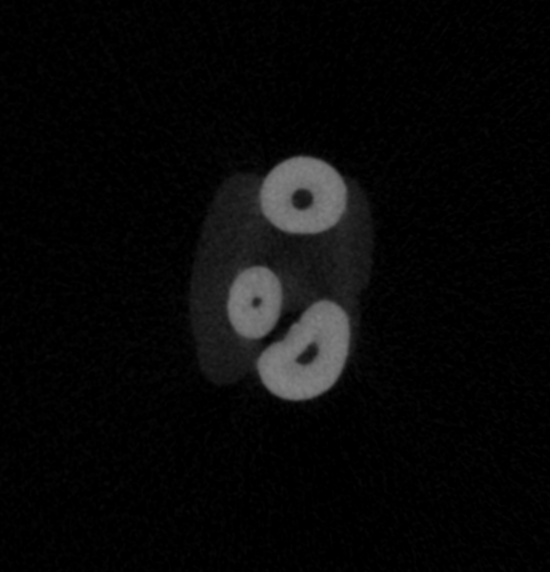

Supplement: S1 File — (ZIP) [file pone.0209698.s001.zip › Skyscan 2211 micro-CT/skyscan2211_0102.tif]

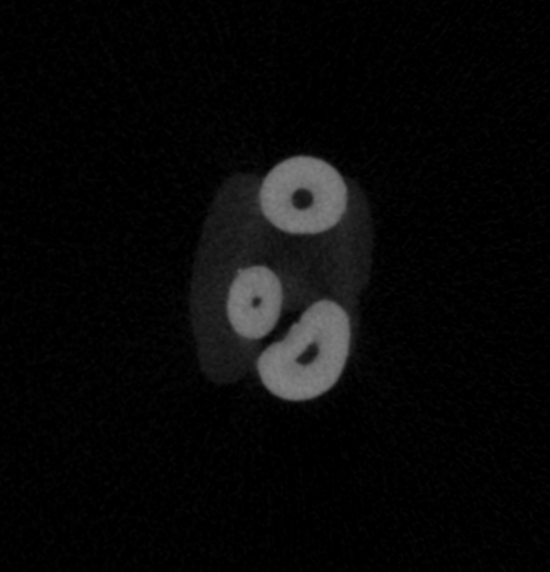

Supplement: S1 File — (ZIP) [file pone.0209698.s001.zip › Skyscan 2211 micro-CT/skyscan2211_0103.tif]

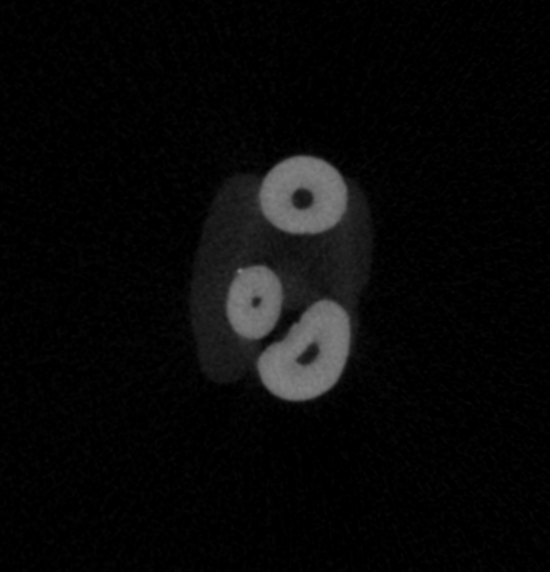

Supplement: S1 File — (ZIP) [file pone.0209698.s001.zip › Skyscan 2211 micro-CT/skyscan2211_0104.tif]

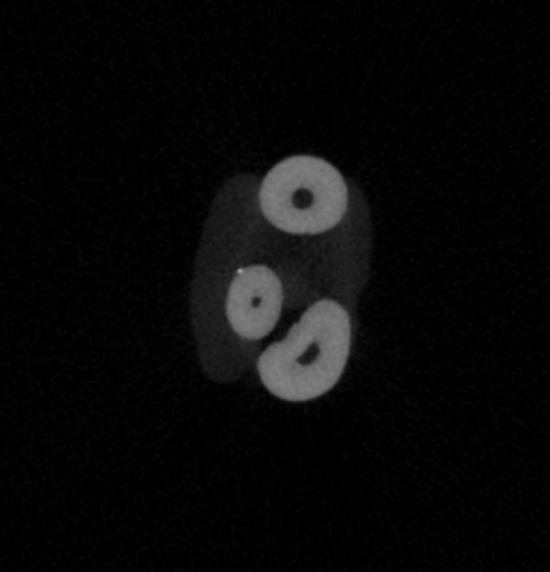

Supplement: S1 File — (ZIP) [file pone.0209698.s001.zip › Skyscan 2211 micro-CT/skyscan2211_0105.tif]

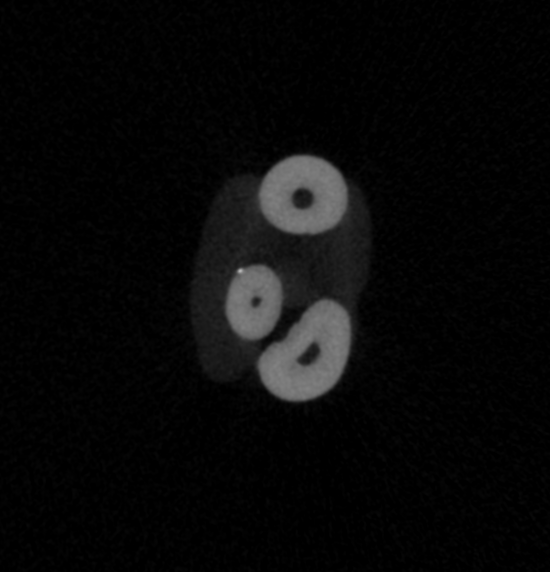

Supplement: S1 File — (ZIP) [file pone.0209698.s001.zip › Skyscan 2211 micro-CT/skyscan2211_0106.tif]

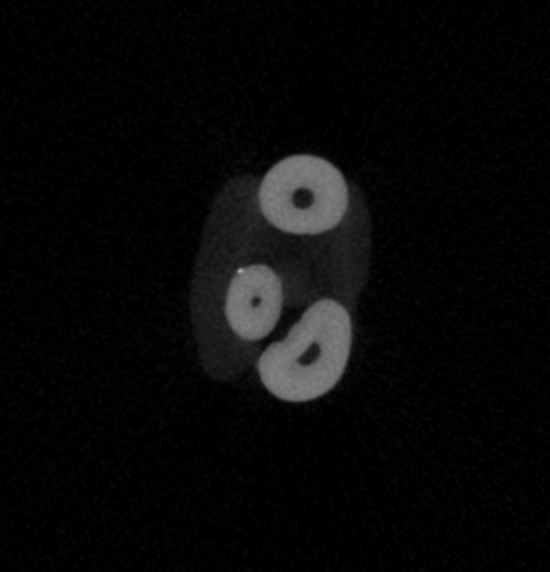

Supplement: S1 File — (ZIP) [file pone.0209698.s001.zip › Skyscan 2211 micro-CT/skyscan2211_0107.tif]

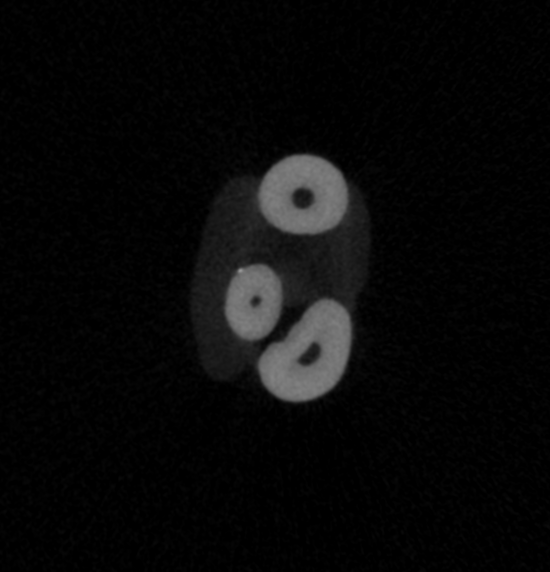

Supplement: S1 File — (ZIP) [file pone.0209698.s001.zip › Skyscan 2211 micro-CT/skyscan2211_0108.tif]

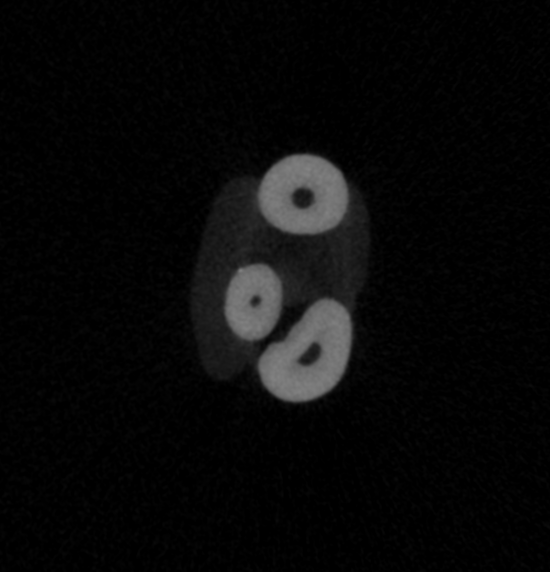

Supplement: S1 File — (ZIP) [file pone.0209698.s001.zip › Skyscan 2211 micro-CT/skyscan2211_0109.tif]

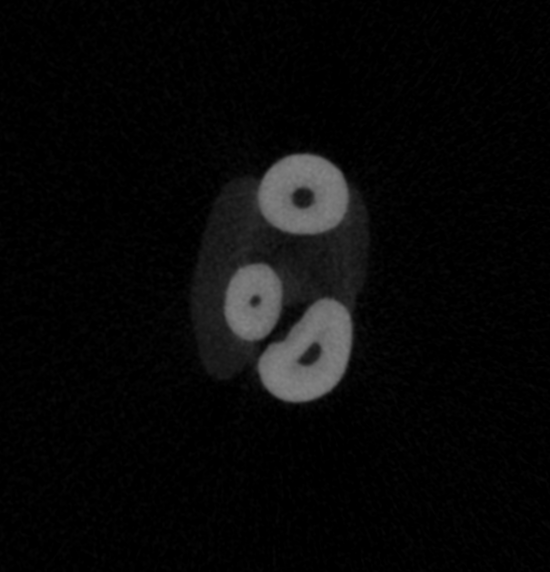

Supplement: S1 File — (ZIP) [file pone.0209698.s001.zip › Skyscan 2211 micro-CT/skyscan2211_0110.tif]

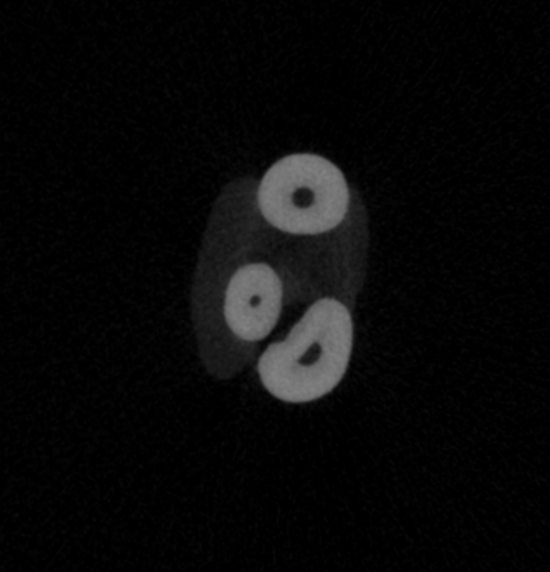

Supplement: S1 File — (ZIP) [file pone.0209698.s001.zip › Skyscan 2211 micro-CT/skyscan2211_0111.tif]

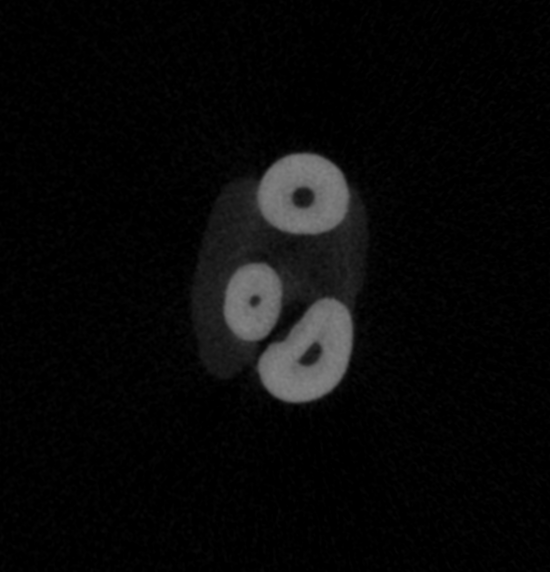

Supplement: S1 File — (ZIP) [file pone.0209698.s001.zip › Skyscan 2211 micro-CT/skyscan2211_0112.tif]

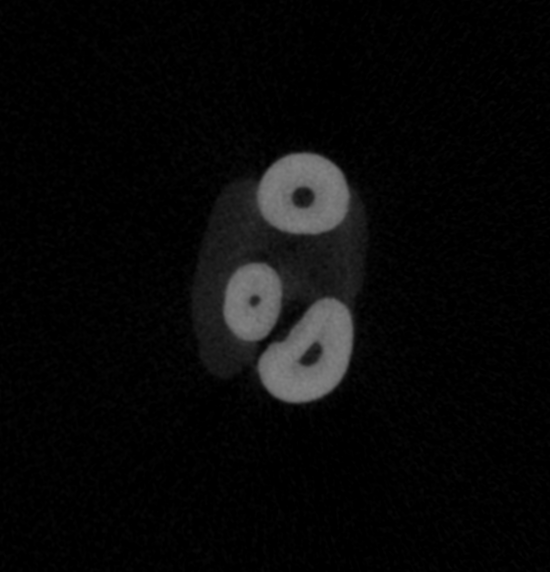

Supplement: S1 File — (ZIP) [file pone.0209698.s001.zip › Skyscan 2211 micro-CT/skyscan2211_0113.tif]

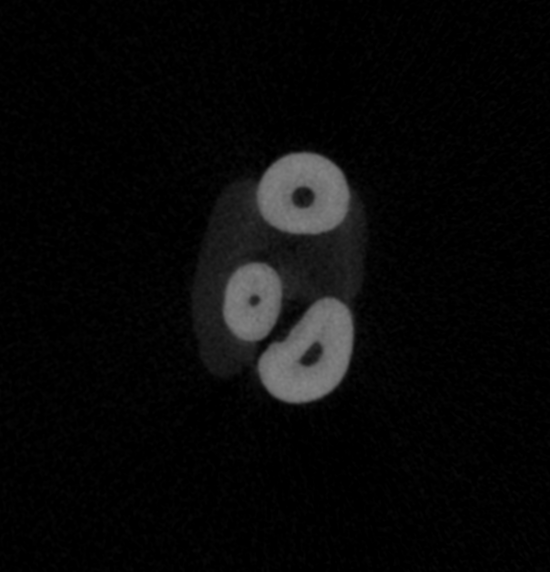

Supplement: S1 File — (ZIP) [file pone.0209698.s001.zip › Skyscan 2211 micro-CT/skyscan2211_0114.tif]

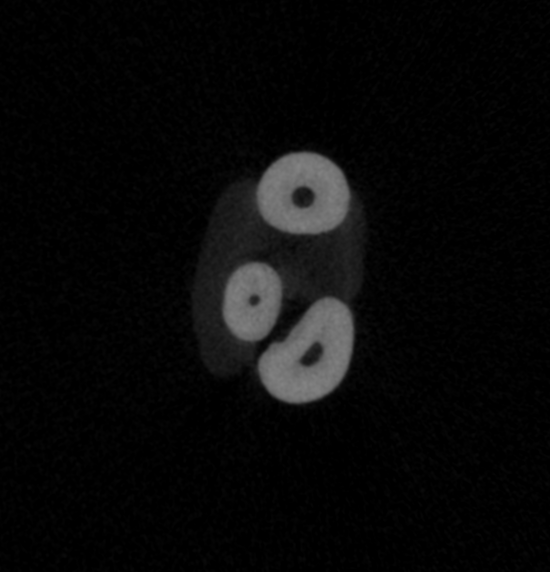

Supplement: S1 File — (ZIP) [file pone.0209698.s001.zip › Skyscan 2211 micro-CT/skyscan2211_0115.tif]

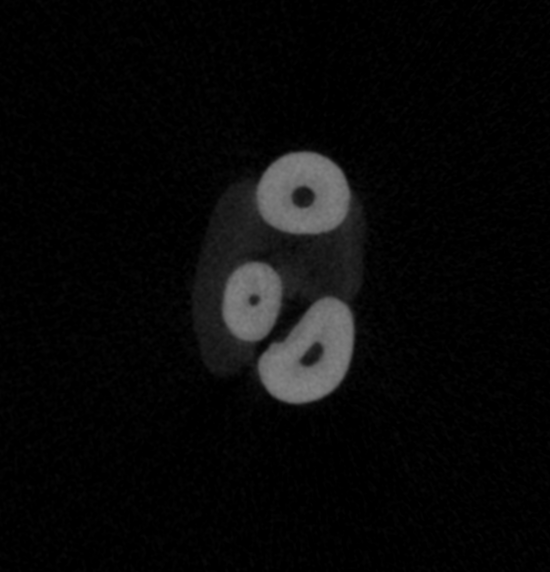

Supplement: S1 File — (ZIP) [file pone.0209698.s001.zip › Skyscan 2211 micro-CT/skyscan2211_0116.tif]

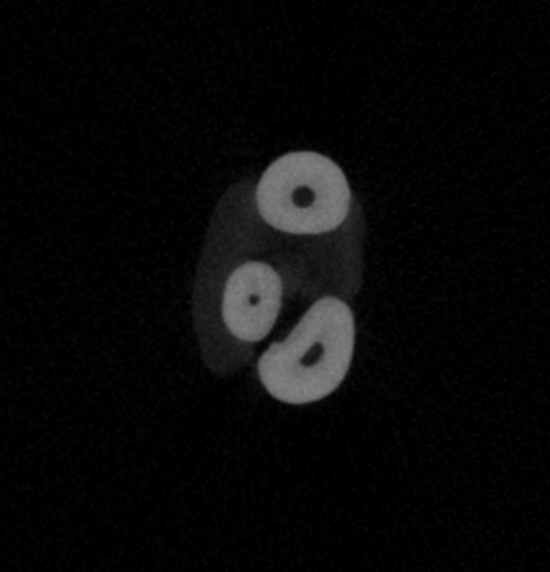

Supplement: S1 File — (ZIP) [file pone.0209698.s001.zip › Skyscan 2211 micro-CT/skyscan2211_0117.tif]

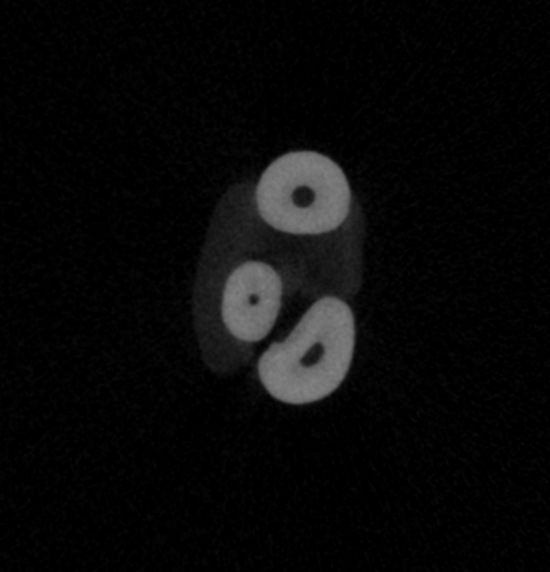

Supplement: S1 File — (ZIP) [file pone.0209698.s001.zip › Skyscan 2211 micro-CT/skyscan2211_0118.tif]

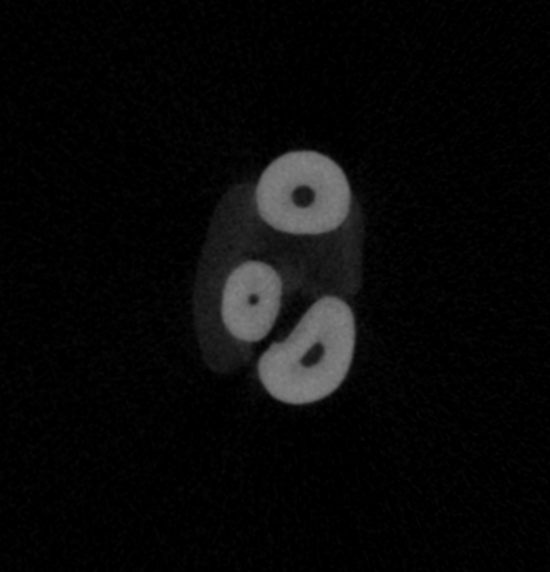

Supplement: S1 File — (ZIP) [file pone.0209698.s001.zip › Skyscan 2211 micro-CT/skyscan2211_0119.tif]

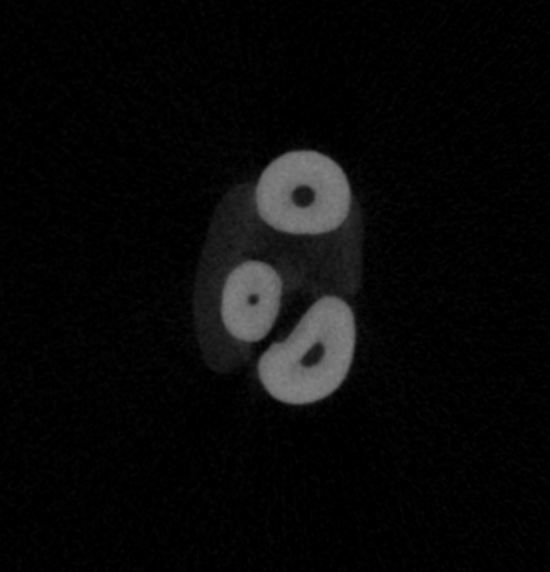

Supplement: S1 File — (ZIP) [file pone.0209698.s001.zip › Skyscan 2211 micro-CT/skyscan2211_0120.tif]

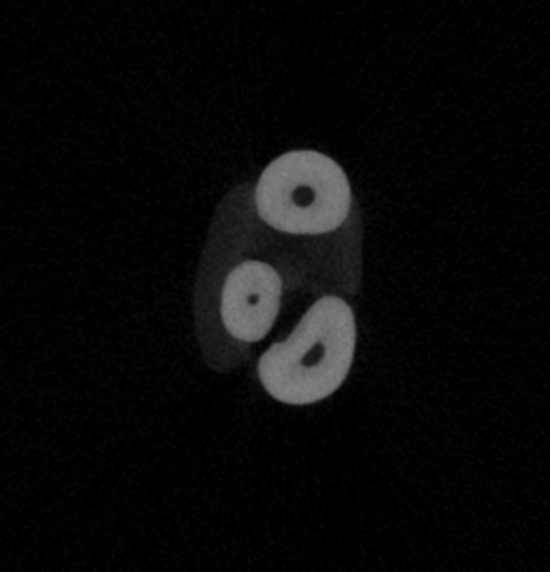

Supplement: S1 File — (ZIP) [file pone.0209698.s001.zip › Skyscan 2211 micro-CT/skyscan2211_0121.tif]

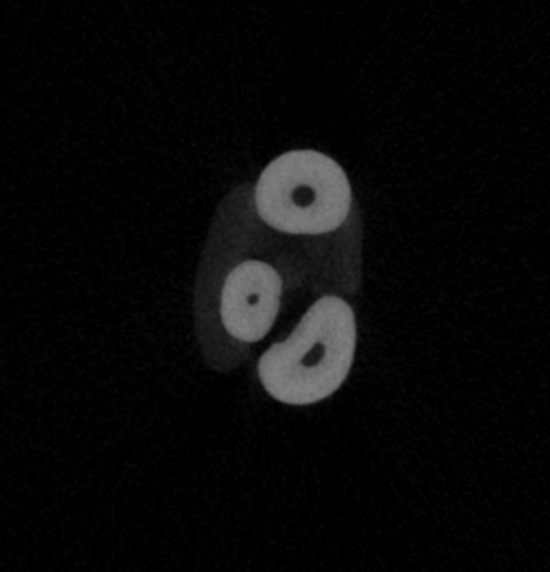

Supplement: S1 File — (ZIP) [file pone.0209698.s001.zip › Skyscan 2211 micro-CT/skyscan2211_0122.tif]

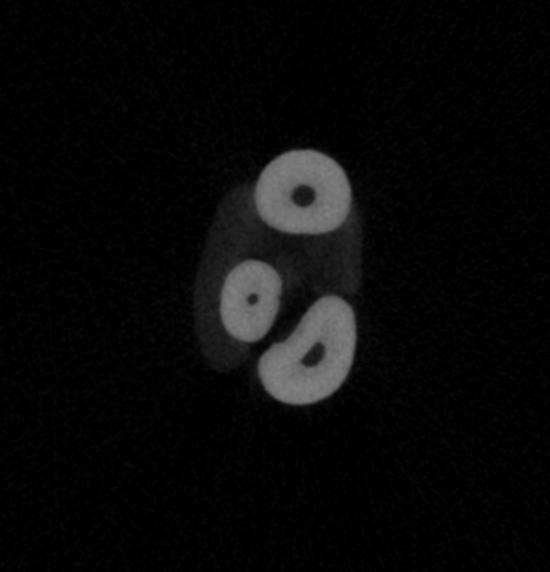

Supplement: S1 File — (ZIP) [file pone.0209698.s001.zip › Skyscan 2211 micro-CT/skyscan2211_0123.tif]

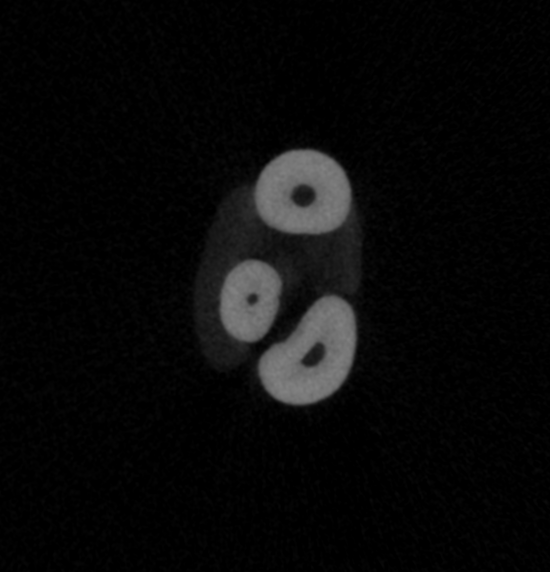

Supplement: S1 File — (ZIP) [file pone.0209698.s001.zip › Skyscan 2211 micro-CT/skyscan2211_0124.tif]

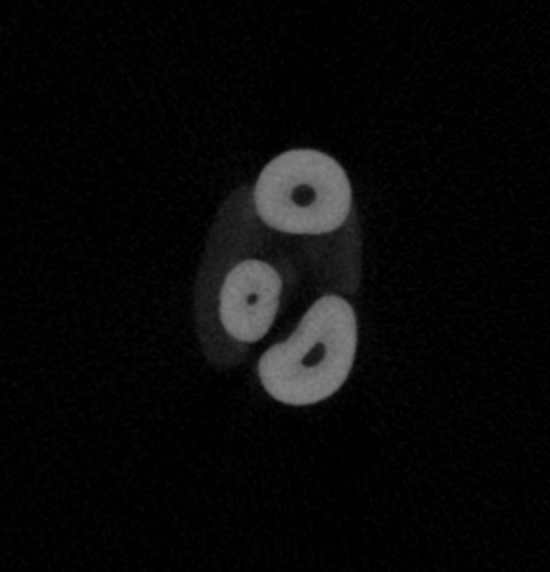

Supplement: S1 File — (ZIP) [file pone.0209698.s001.zip › Skyscan 2211 micro-CT/skyscan2211_0125.tif]

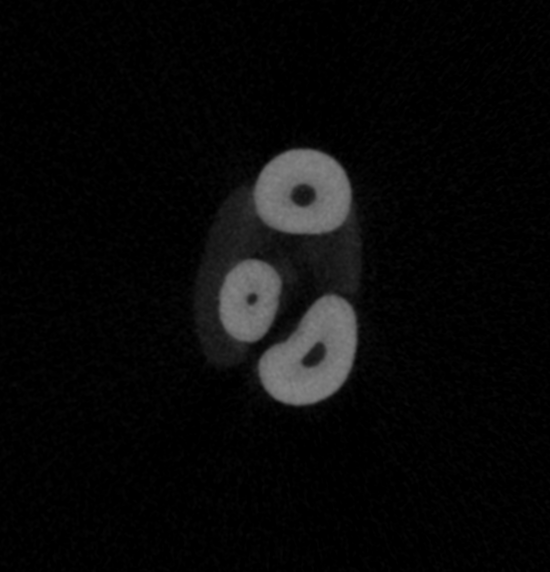

Supplement: S1 File — (ZIP) [file pone.0209698.s001.zip › Skyscan 2211 micro-CT/skyscan2211_0126.tif]

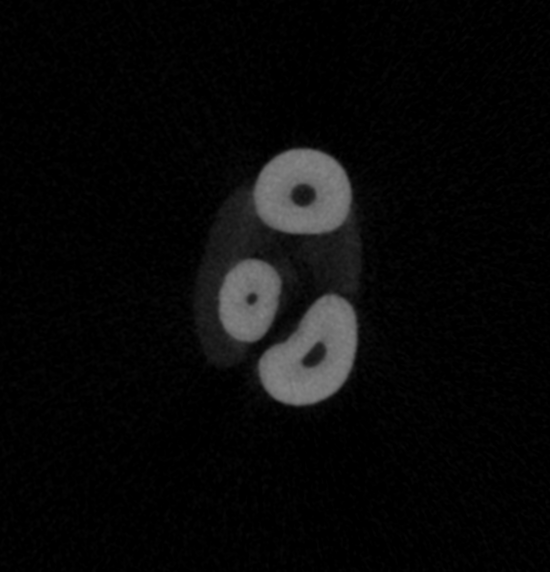

Supplement: S1 File — (ZIP) [file pone.0209698.s001.zip › Skyscan 2211 micro-CT/skyscan2211_0127.tif]

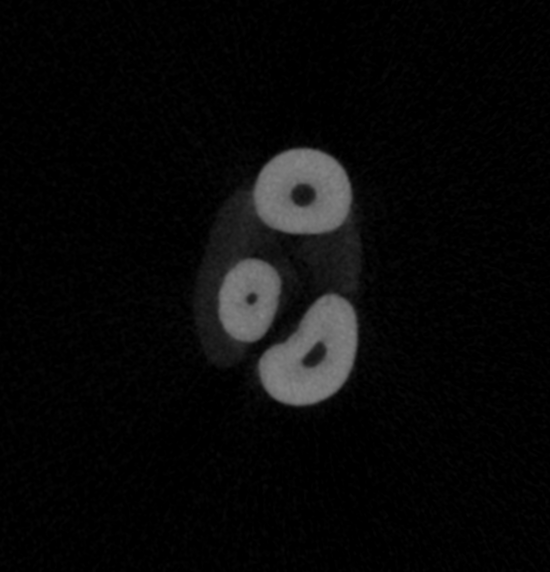

Supplement: S1 File — (ZIP) [file pone.0209698.s001.zip › Skyscan 2211 micro-CT/skyscan2211_0128.tif]

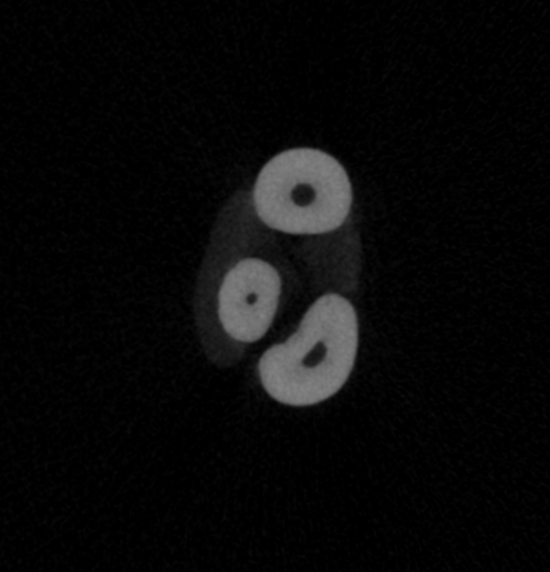

Supplement: S1 File — (ZIP) [file pone.0209698.s001.zip › Skyscan 2211 micro-CT/skyscan2211_0129.tif]

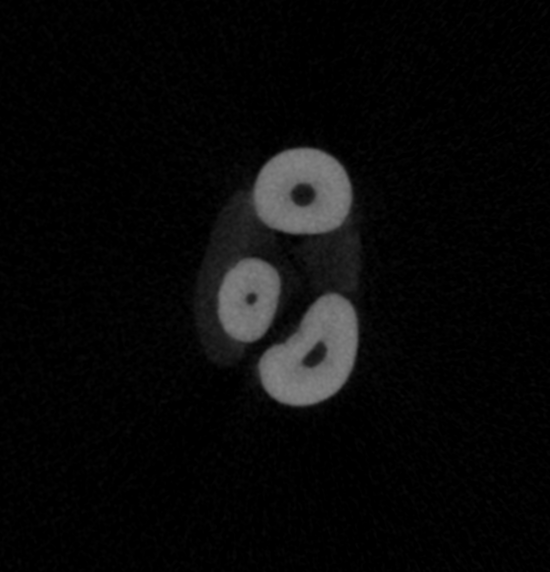

Supplement: S1 File — (ZIP) [file pone.0209698.s001.zip › Skyscan 2211 micro-CT/skyscan2211_0130.tif]

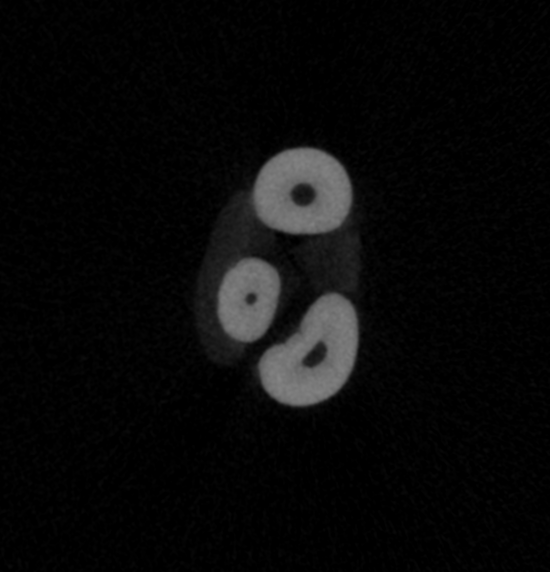

Supplement: S1 File — (ZIP) [file pone.0209698.s001.zip › Skyscan 2211 micro-CT/skyscan2211_0131.tif]

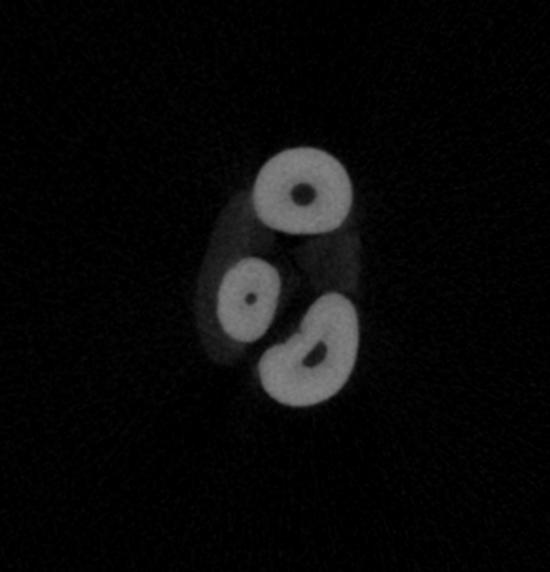

Supplement: S1 File — (ZIP) [file pone.0209698.s001.zip › Skyscan 2211 micro-CT/skyscan2211_0132.tif]

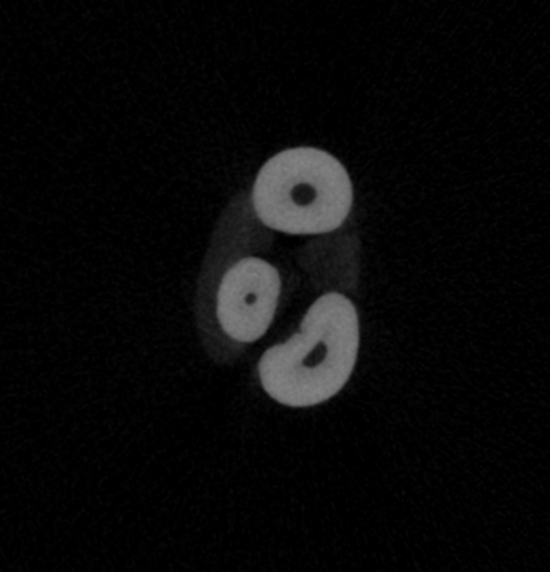

Supplement: S1 File — (ZIP) [file pone.0209698.s001.zip › Skyscan 2211 micro-CT/skyscan2211_0133.tif]

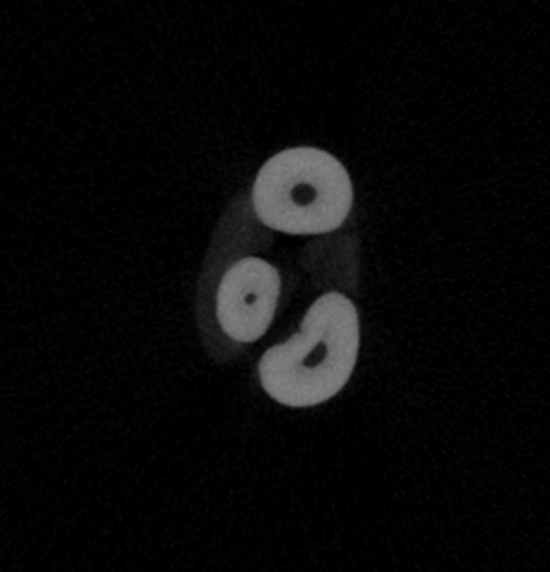

Supplement: S1 File — (ZIP) [file pone.0209698.s001.zip › Skyscan 2211 micro-CT/skyscan2211_0134.tif]

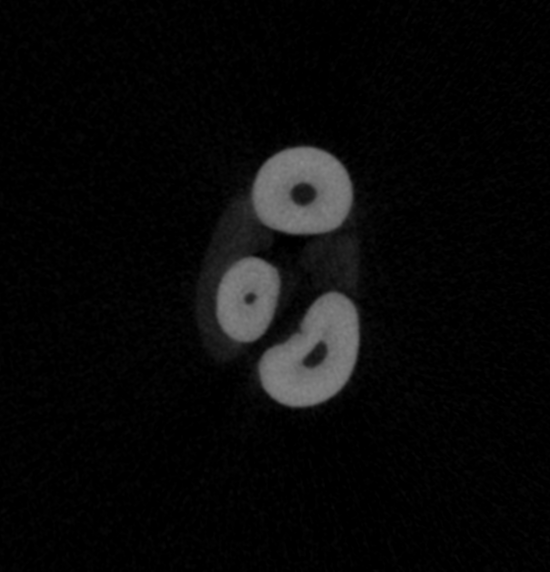

Supplement: S1 File — (ZIP) [file pone.0209698.s001.zip › Skyscan 2211 micro-CT/skyscan2211_0135.tif]

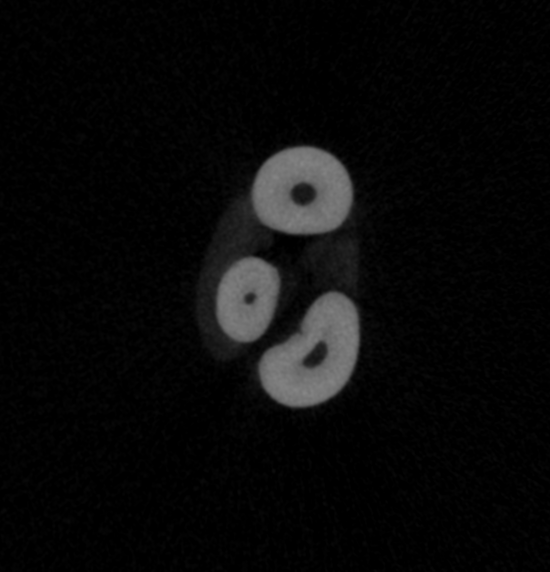

Supplement: S1 File — (ZIP) [file pone.0209698.s001.zip › Skyscan 2211 micro-CT/skyscan2211_0136.tif]

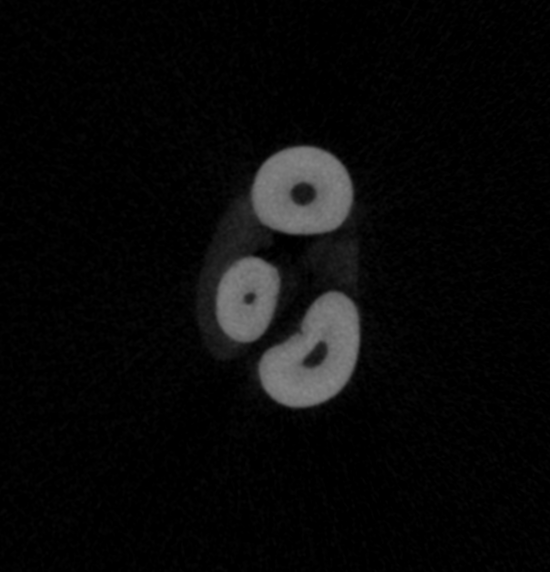

Supplement: S1 File — (ZIP) [file pone.0209698.s001.zip › Skyscan 2211 micro-CT/skyscan2211_0137.tif]

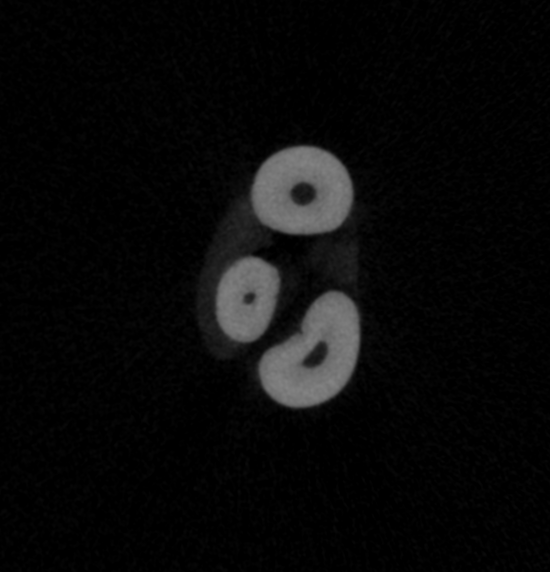

Supplement: S1 File — (ZIP) [file pone.0209698.s001.zip › Skyscan 2211 micro-CT/skyscan2211_0138.tif]

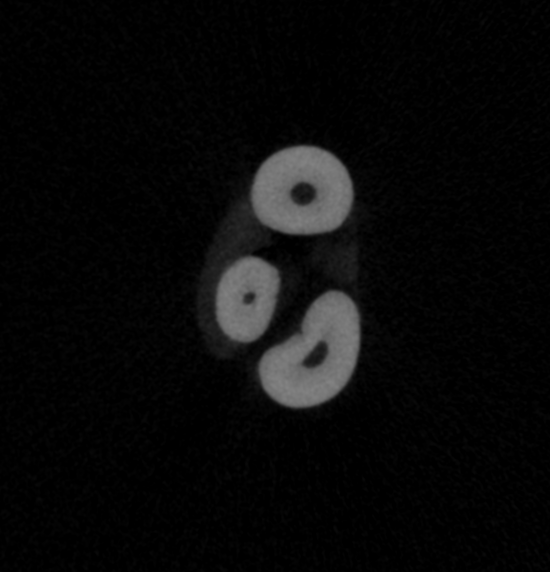

Supplement: S1 File — (ZIP) [file pone.0209698.s001.zip › Skyscan 2211 micro-CT/skyscan2211_0139.tif]

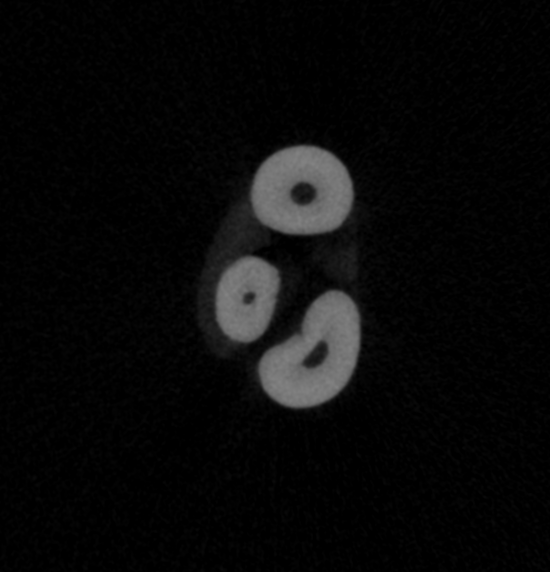

Supplement: S1 File — (ZIP) [file pone.0209698.s001.zip › Skyscan 2211 micro-CT/skyscan2211_0140.tif]

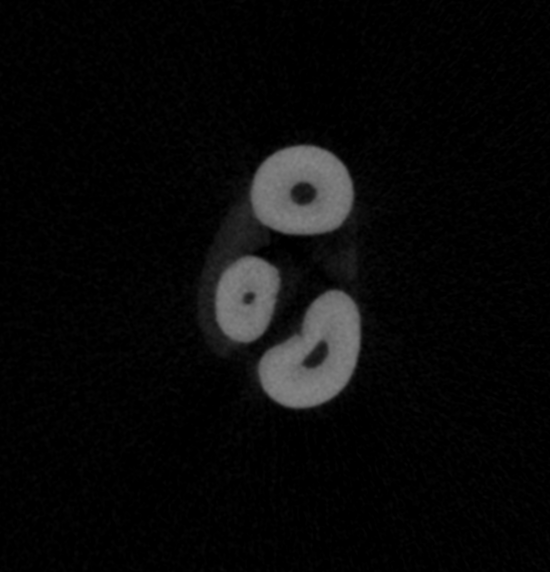

Supplement: S1 File — (ZIP) [file pone.0209698.s001.zip › Skyscan 2211 micro-CT/skyscan2211_0141.tif]

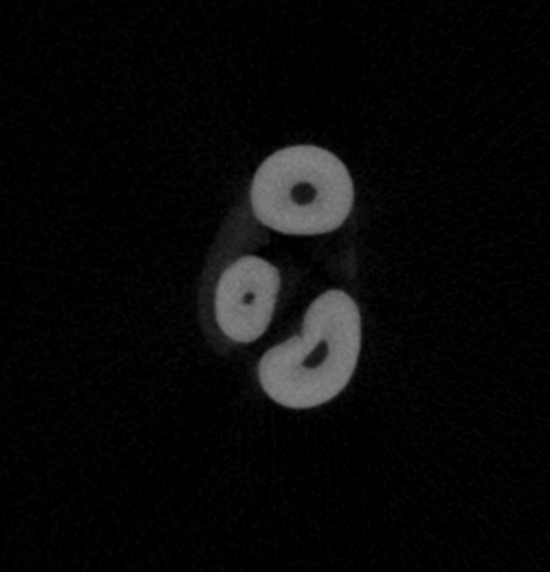

Supplement: S1 File — (ZIP) [file pone.0209698.s001.zip › Skyscan 2211 micro-CT/skyscan2211_0142.tif]

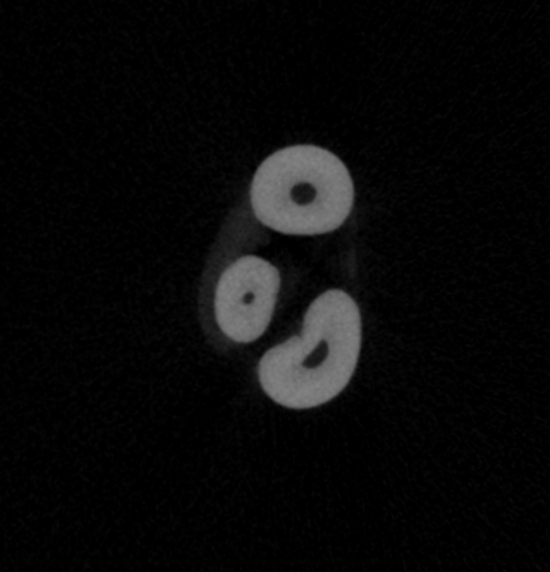

Supplement: S1 File — (ZIP) [file pone.0209698.s001.zip › Skyscan 2211 micro-CT/skyscan2211_0143.tif]

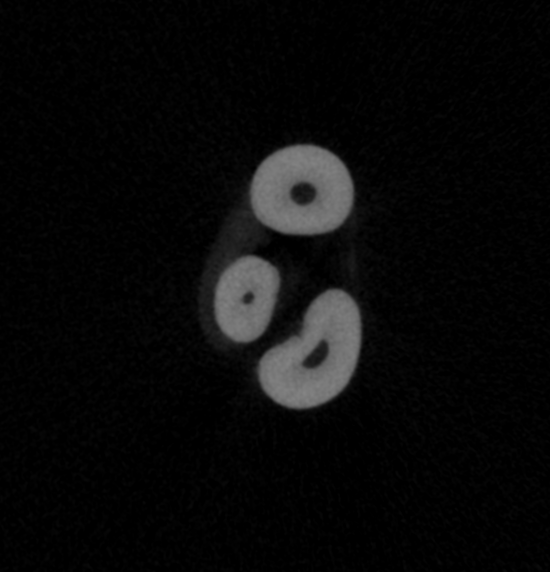

Supplement: S1 File — (ZIP) [file pone.0209698.s001.zip › Skyscan 2211 micro-CT/skyscan2211_0144.tif]

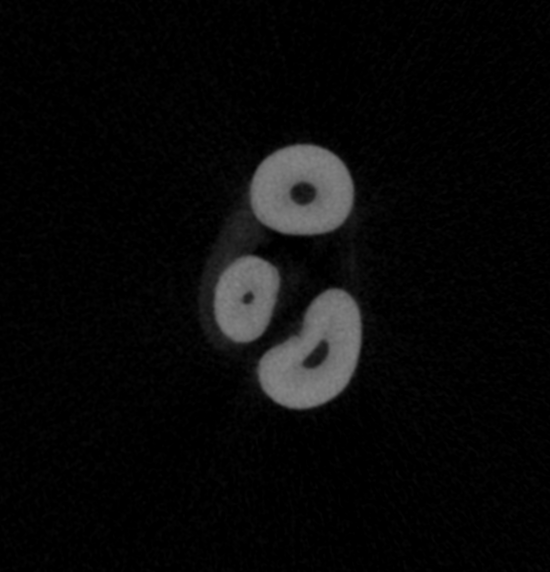

Supplement: S1 File — (ZIP) [file pone.0209698.s001.zip › Skyscan 2211 micro-CT/skyscan2211_0145.tif]

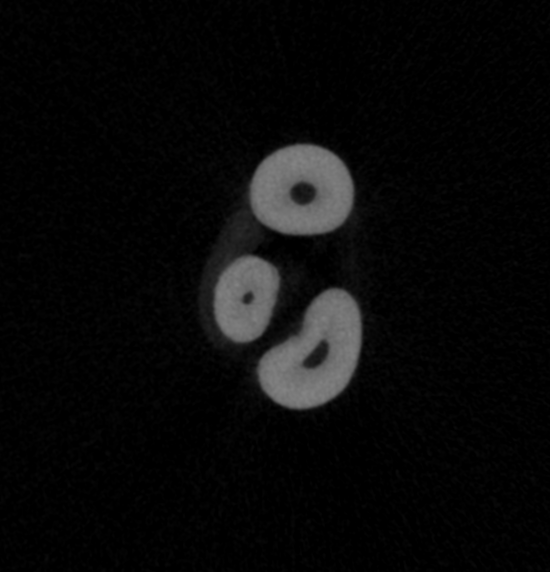

Supplement: S1 File — (ZIP) [file pone.0209698.s001.zip › Skyscan 2211 micro-CT/skyscan2211_0146.tif]

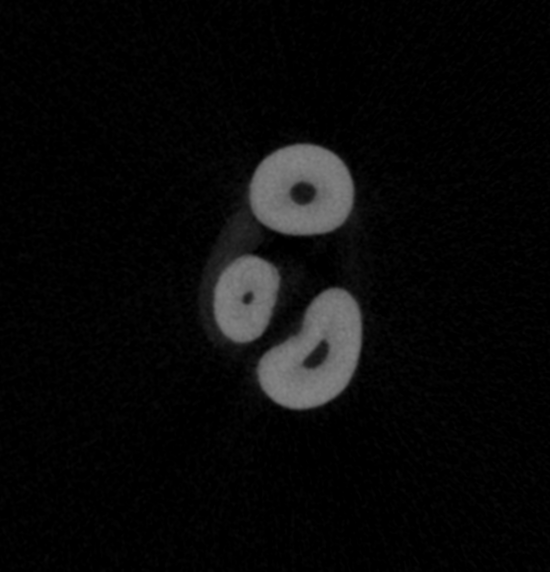

Supplement: S1 File — (ZIP) [file pone.0209698.s001.zip › Skyscan 2211 micro-CT/skyscan2211_0147.tif]

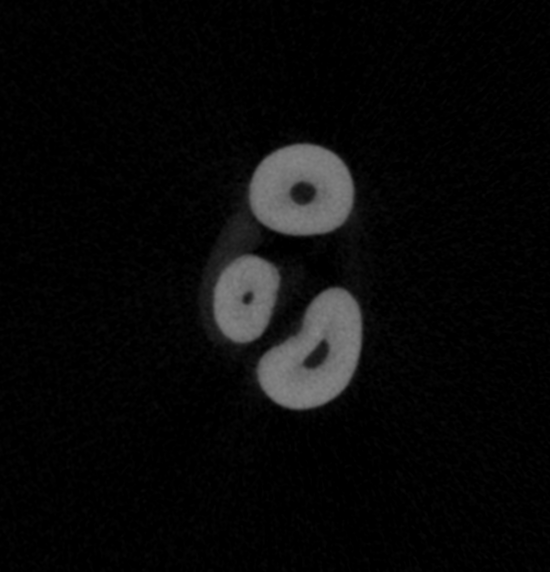

Supplement: S1 File — (ZIP) [file pone.0209698.s001.zip › Skyscan 2211 micro-CT/skyscan2211_0148.tif]

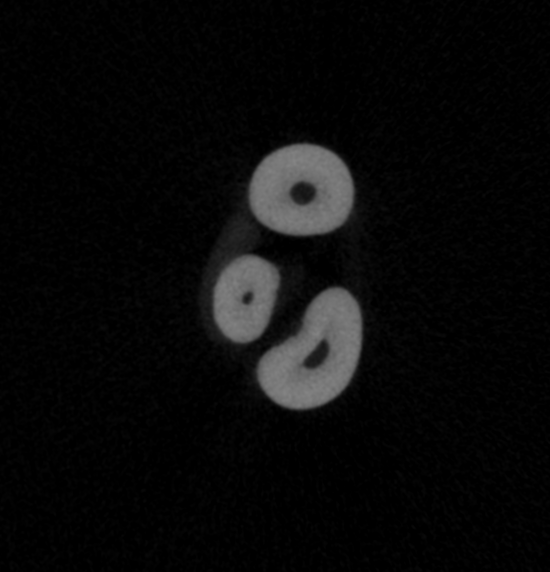

Supplement: S1 File — (ZIP) [file pone.0209698.s001.zip › Skyscan 2211 micro-CT/skyscan2211_0149.tif]

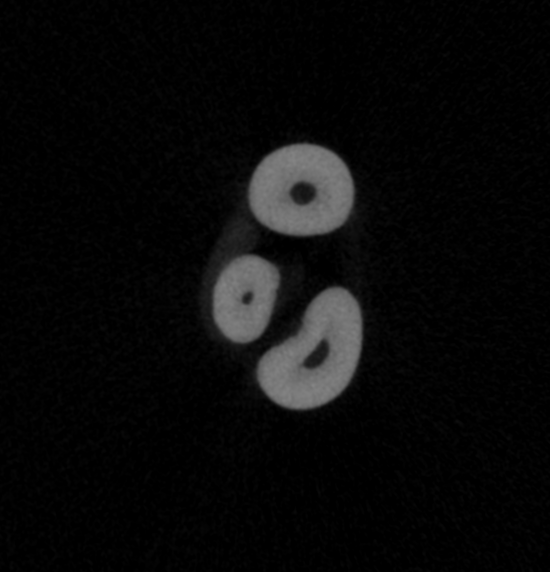

Supplement: S1 File — (ZIP) [file pone.0209698.s001.zip › Skyscan 2211 micro-CT/skyscan2211_0150.tif]

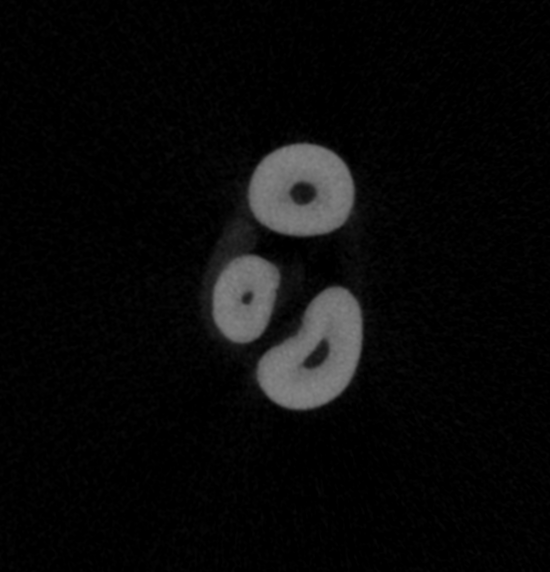

Supplement: S1 File — (ZIP) [file pone.0209698.s001.zip › Skyscan 2211 micro-CT/skyscan2211_0151.tif]

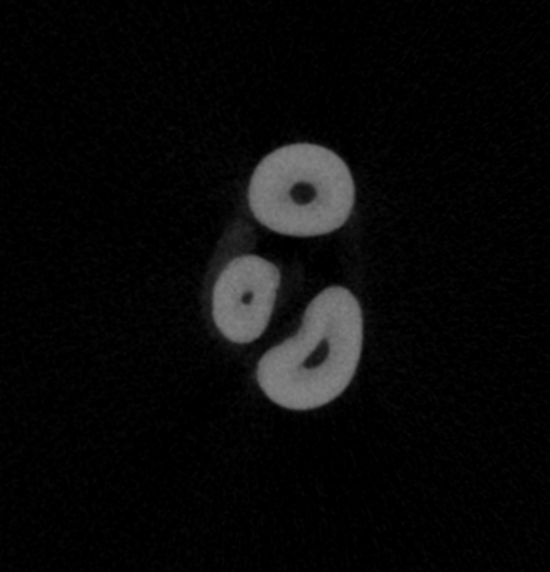

Supplement: S1 File — (ZIP) [file pone.0209698.s001.zip › Skyscan 2211 micro-CT/skyscan2211_0152.tif]

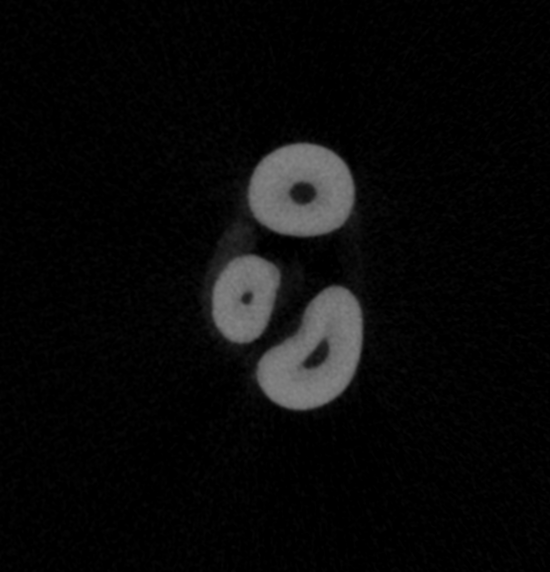

Supplement: S1 File — (ZIP) [file pone.0209698.s001.zip › Skyscan 2211 micro-CT/skyscan2211_0153.tif]

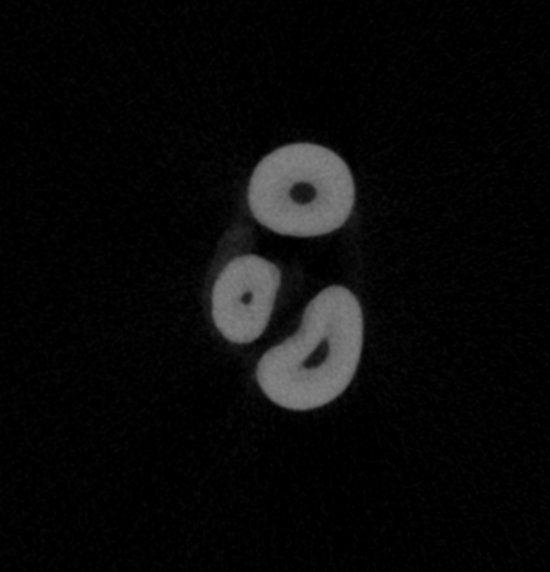

Supplement: S1 File — (ZIP) [file pone.0209698.s001.zip › Skyscan 2211 micro-CT/skyscan2211_0154.tif]

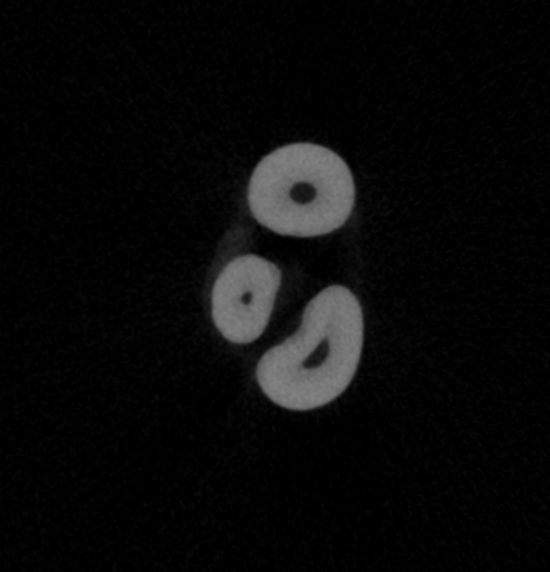

Supplement: S1 File — (ZIP) [file pone.0209698.s001.zip › Skyscan 2211 micro-CT/skyscan2211_0155.tif]

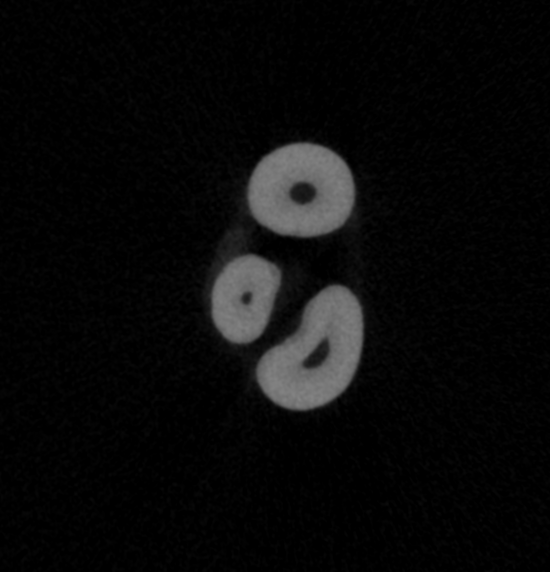

Supplement: S1 File — (ZIP) [file pone.0209698.s001.zip › Skyscan 2211 micro-CT/skyscan2211_0156.tif]

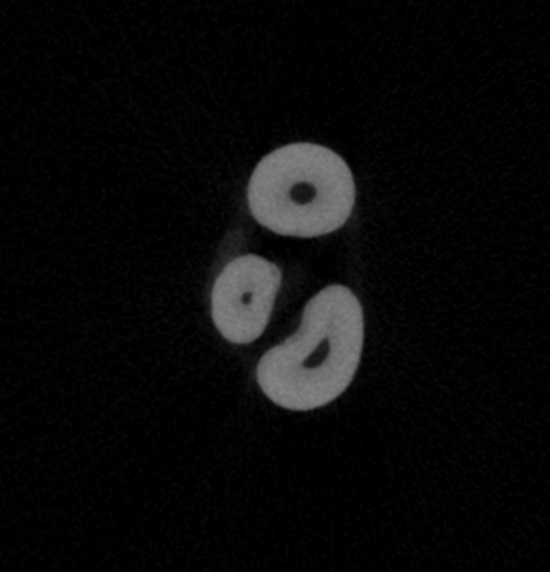

Supplement: S1 File — (ZIP) [file pone.0209698.s001.zip › Skyscan 2211 micro-CT/skyscan2211_0157.tif]

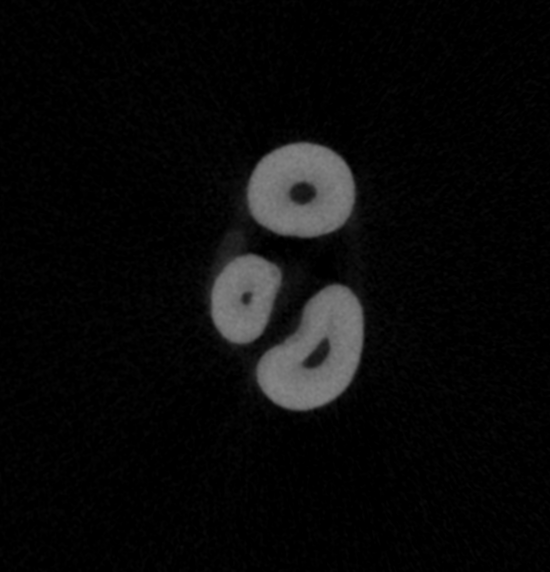

Supplement: S1 File — (ZIP) [file pone.0209698.s001.zip › Skyscan 2211 micro-CT/skyscan2211_0158.tif]

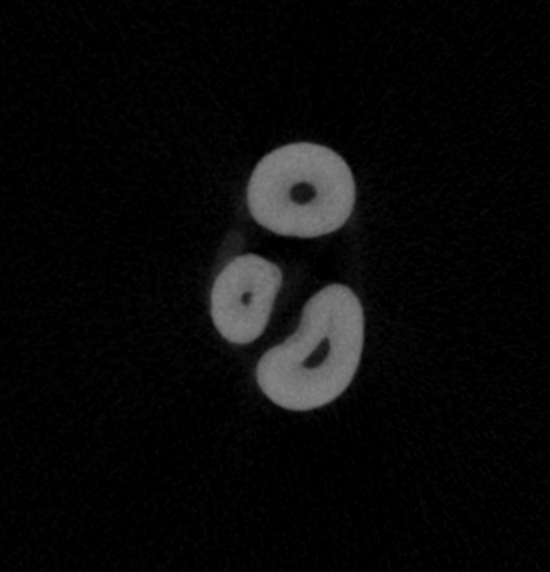

Supplement: S1 File — (ZIP) [file pone.0209698.s001.zip › Skyscan 2211 micro-CT/skyscan2211_0159.tif]

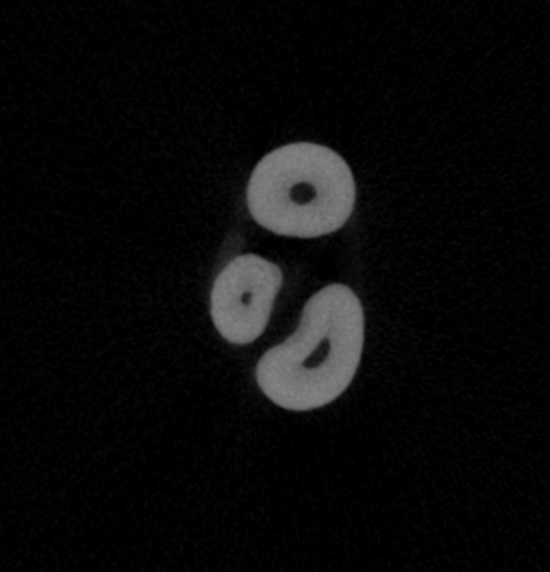

Supplement: S1 File — (ZIP) [file pone.0209698.s001.zip › Skyscan 2211 micro-CT/skyscan2211_0160.tif]

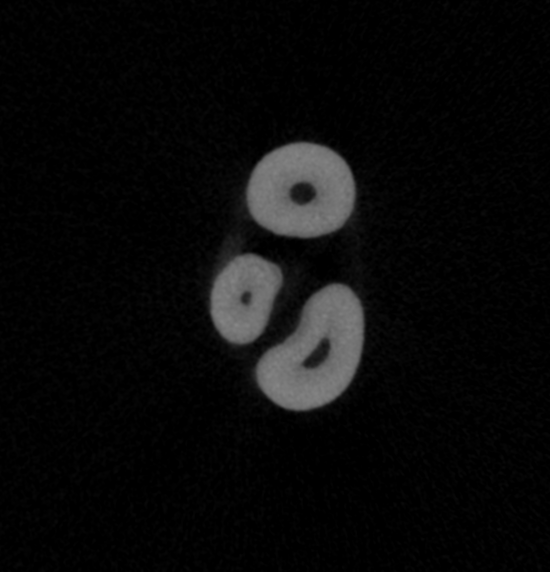

Supplement: S1 File — (ZIP) [file pone.0209698.s001.zip › Skyscan 2211 micro-CT/skyscan2211_0161.tif]

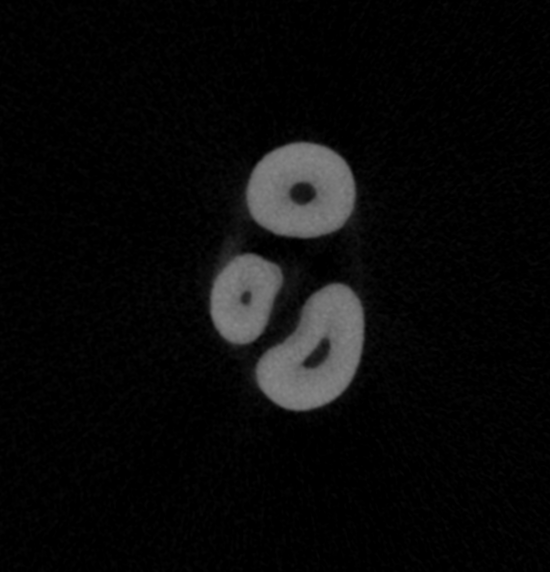

Supplement: S1 File — (ZIP) [file pone.0209698.s001.zip › Skyscan 2211 micro-CT/skyscan2211_0162.tif]

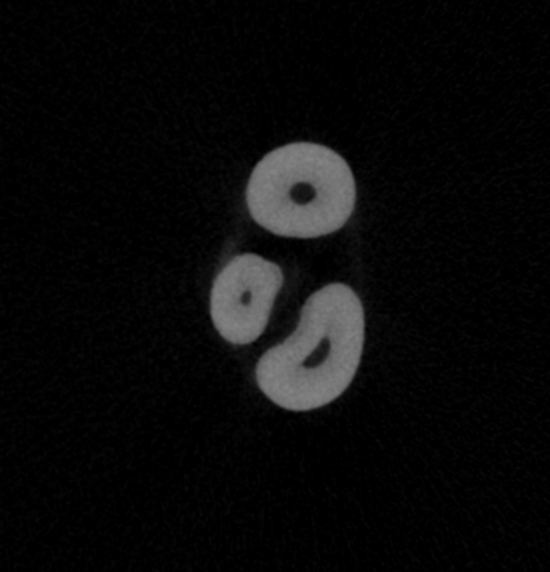

Supplement: S1 File — (ZIP) [file pone.0209698.s001.zip › Skyscan 2211 micro-CT/skyscan2211_0163.tif]

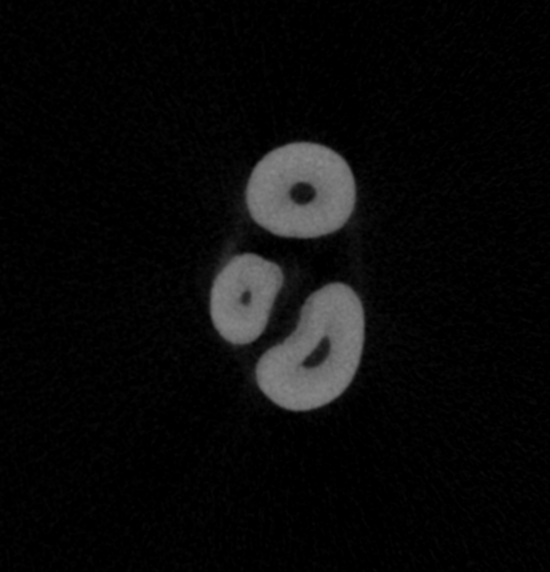

Supplement: S1 File — (ZIP) [file pone.0209698.s001.zip › Skyscan 2211 micro-CT/skyscan2211_0164.tif]

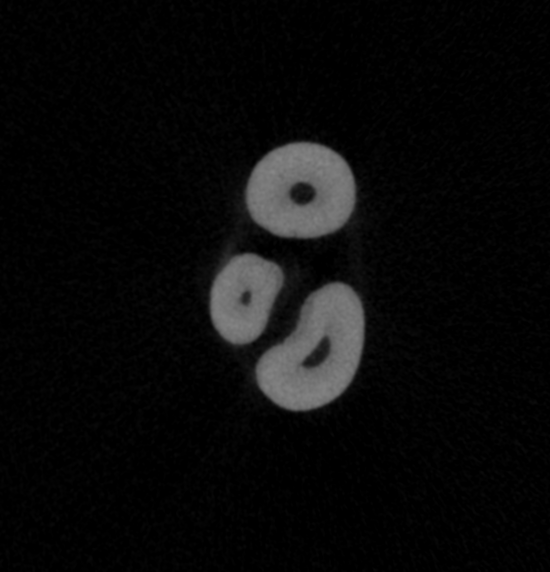

Supplement: S1 File — (ZIP) [file pone.0209698.s001.zip › Skyscan 2211 micro-CT/skyscan2211_0165.tif]

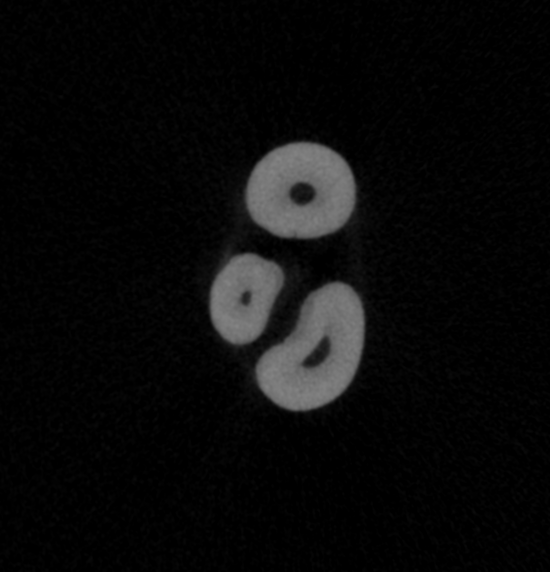

Supplement: S1 File — (ZIP) [file pone.0209698.s001.zip › Skyscan 2211 micro-CT/skyscan2211_0166.tif]

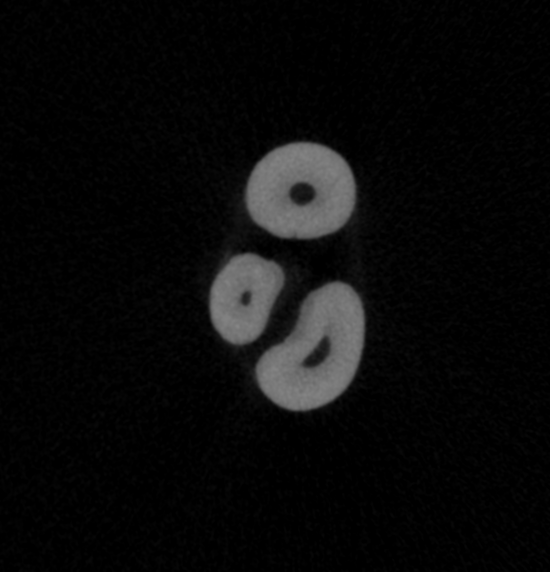

Supplement: S1 File — (ZIP) [file pone.0209698.s001.zip › Skyscan 2211 micro-CT/skyscan2211_0167.tif]

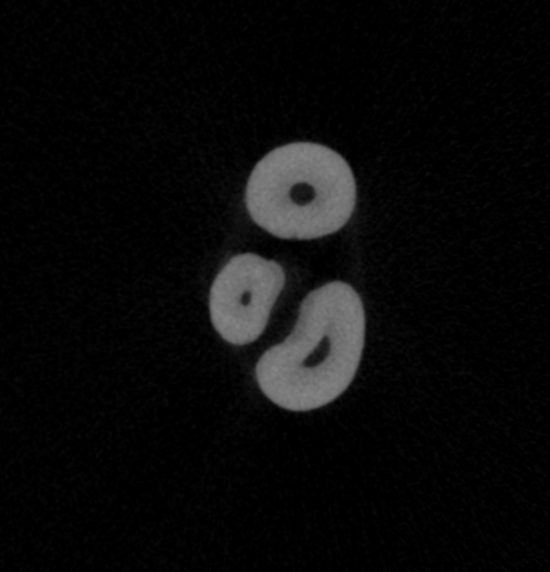

Supplement: S1 File — (ZIP) [file pone.0209698.s001.zip › Skyscan 2211 micro-CT/skyscan2211_0168.tif]

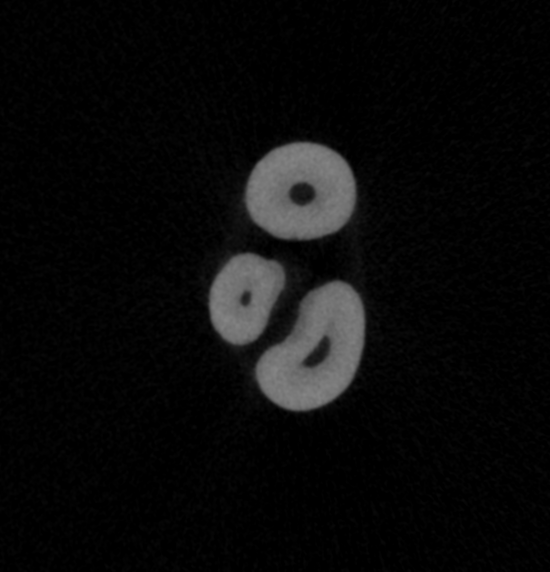

Supplement: S1 File — (ZIP) [file pone.0209698.s001.zip › Skyscan 2211 micro-CT/skyscan2211_0169.tif]

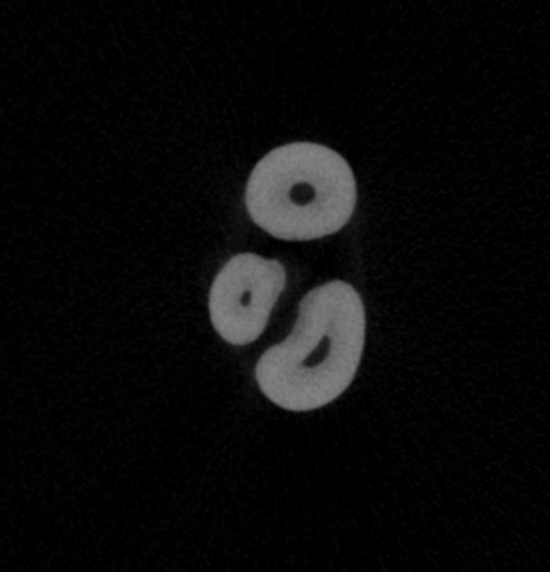

Supplement: S1 File — (ZIP) [file pone.0209698.s001.zip › Skyscan 2211 micro-CT/skyscan2211_0170.tif]

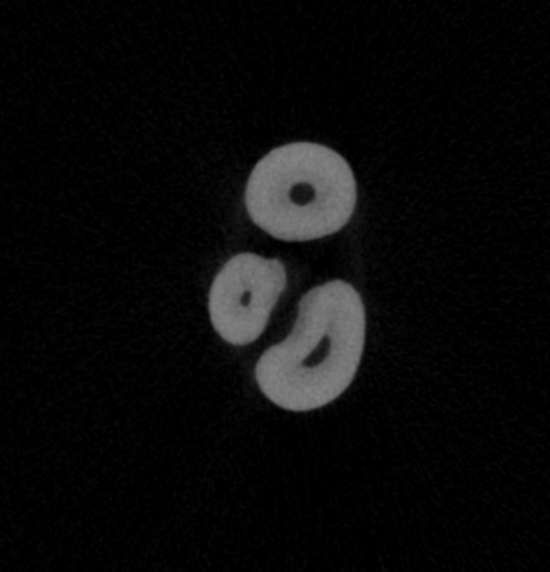

Supplement: S1 File — (ZIP) [file pone.0209698.s001.zip › Skyscan 2211 micro-CT/skyscan2211_0171.tif]

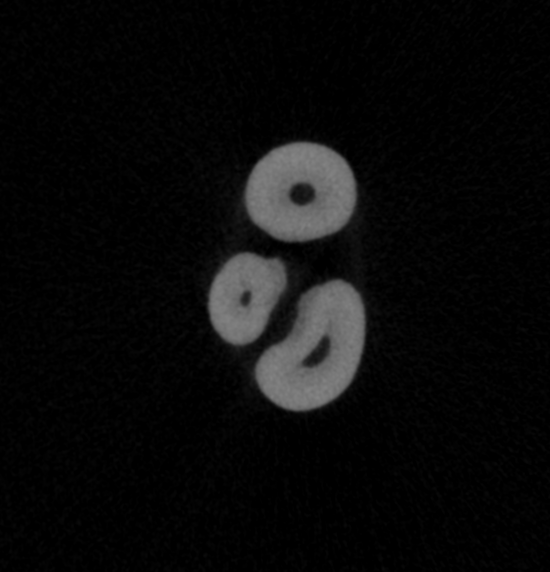

Supplement: S1 File — (ZIP) [file pone.0209698.s001.zip › Skyscan 2211 micro-CT/skyscan2211_0172.tif]

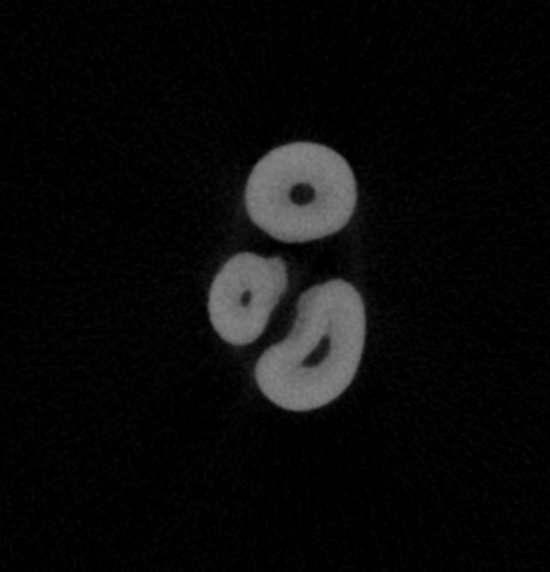

Supplement: S1 File — (ZIP) [file pone.0209698.s001.zip › Skyscan 2211 micro-CT/skyscan2211_0173.tif]

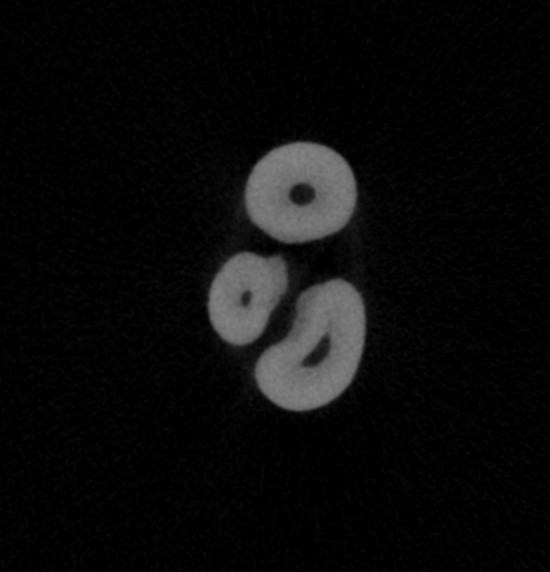

Supplement: S1 File — (ZIP) [file pone.0209698.s001.zip › Skyscan 2211 micro-CT/skyscan2211_0174.tif]

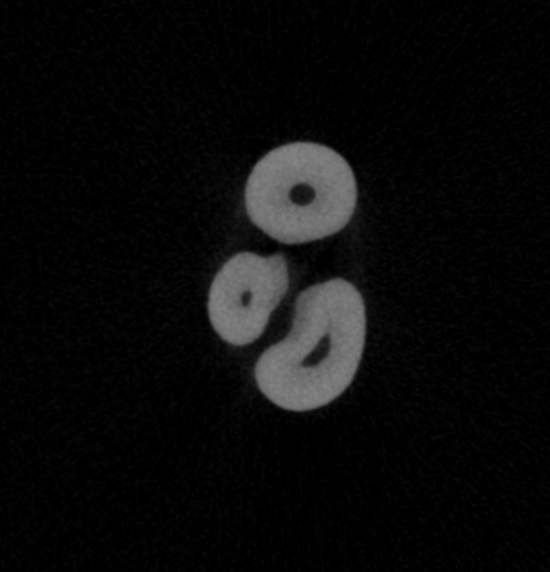

Supplement: S1 File — (ZIP) [file pone.0209698.s001.zip › Skyscan 2211 micro-CT/skyscan2211_0175.tif]

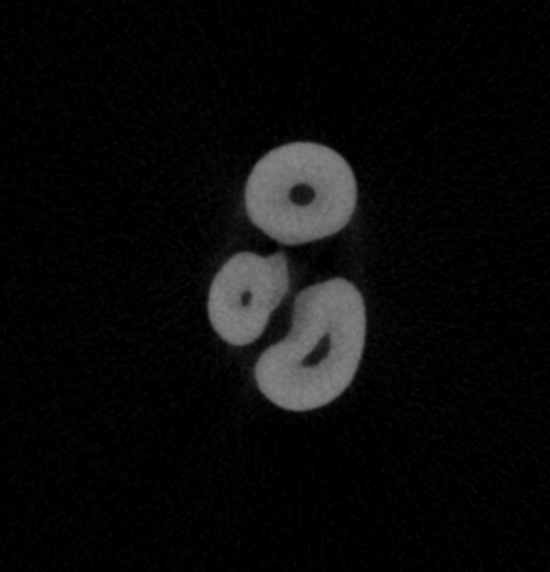

Supplement: S1 File — (ZIP) [file pone.0209698.s001.zip › Skyscan 2211 micro-CT/skyscan2211_0176.tif]

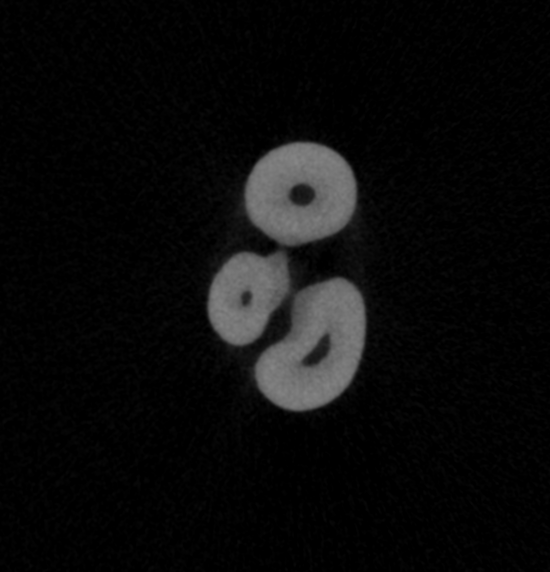

Supplement: S1 File — (ZIP) [file pone.0209698.s001.zip › Skyscan 2211 micro-CT/skyscan2211_0177.tif]

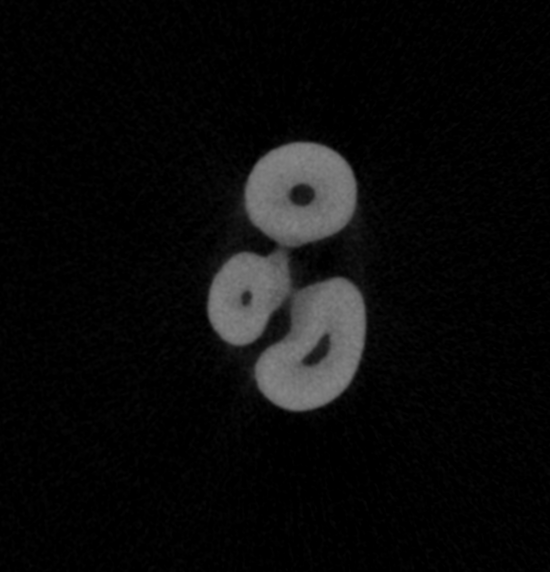

Supplement: S1 File — (ZIP) [file pone.0209698.s001.zip › Skyscan 2211 micro-CT/skyscan2211_0178.tif]

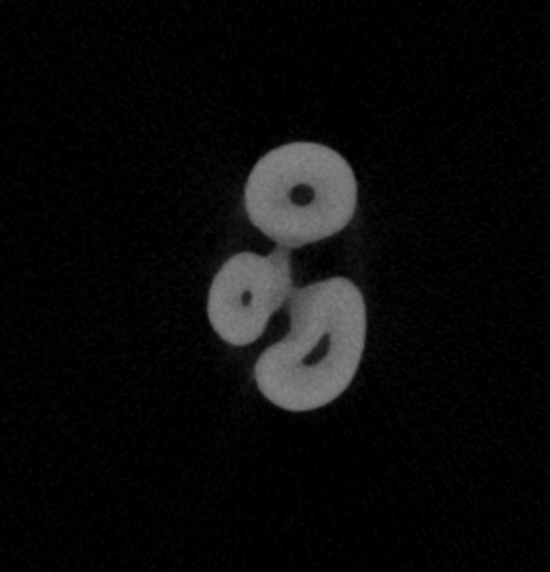

Supplement: S1 File — (ZIP) [file pone.0209698.s001.zip › Skyscan 2211 micro-CT/skyscan2211_0179.tif]

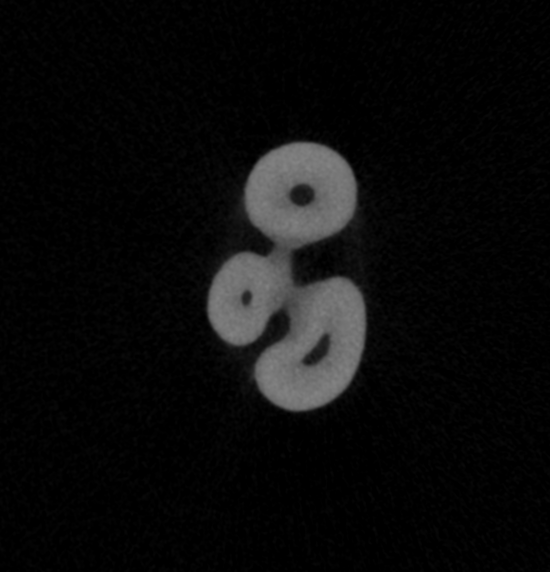

Supplement: S1 File — (ZIP) [file pone.0209698.s001.zip › Skyscan 2211 micro-CT/skyscan2211_0180.tif]

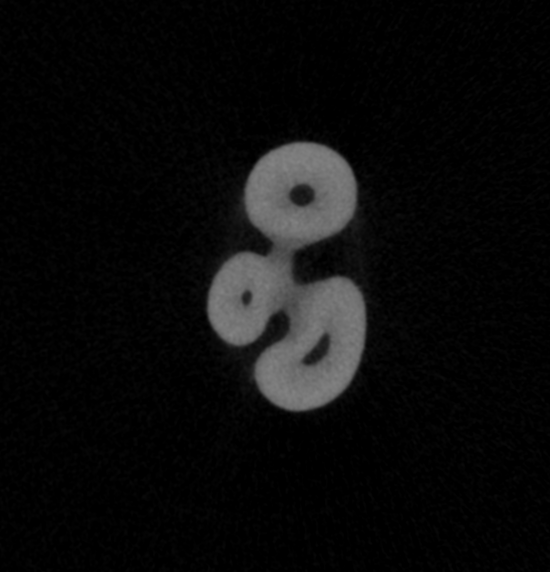

Supplement: S1 File — (ZIP) [file pone.0209698.s001.zip › Skyscan 2211 micro-CT/skyscan2211_0181.tif]

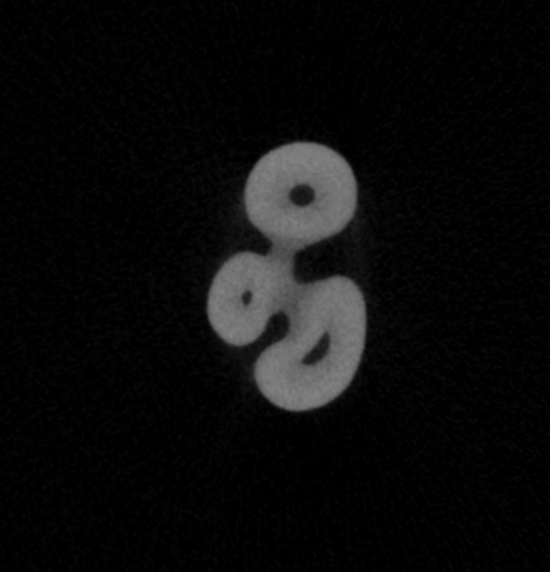

Supplement: S1 File — (ZIP) [file pone.0209698.s001.zip › Skyscan 2211 micro-CT/skyscan2211_0182.tif]

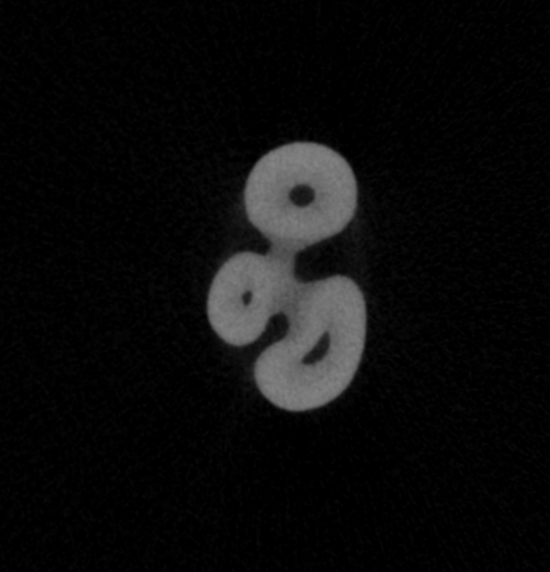

Supplement: S1 File — (ZIP) [file pone.0209698.s001.zip › Skyscan 2211 micro-CT/skyscan2211_0183.tif]

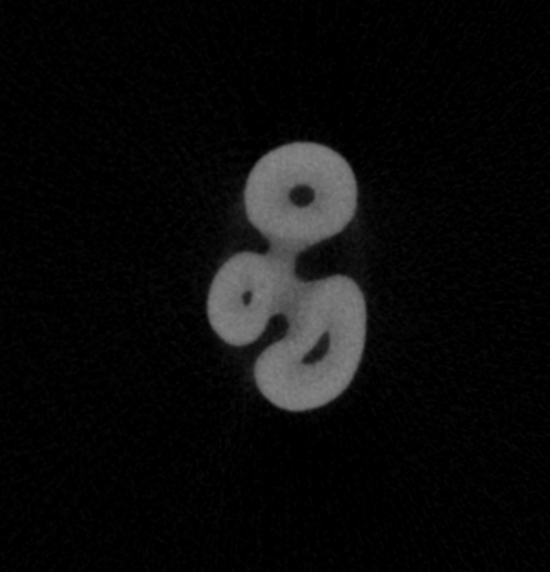

Supplement: S1 File — (ZIP) [file pone.0209698.s001.zip › Skyscan 2211 micro-CT/skyscan2211_0184.tif]

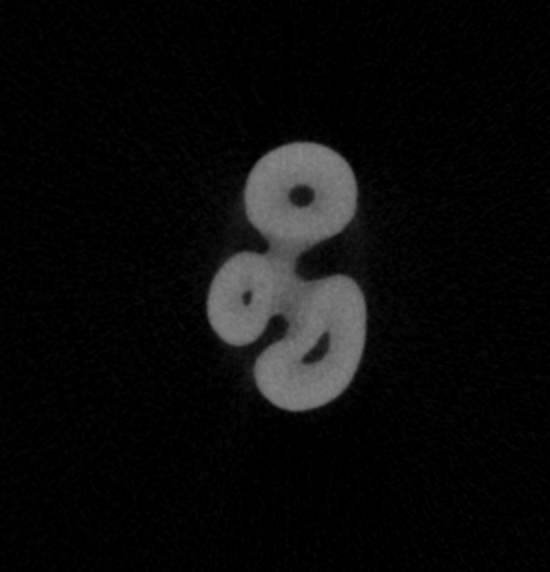

Supplement: S1 File — (ZIP) [file pone.0209698.s001.zip › Skyscan 2211 micro-CT/skyscan2211_0185.tif]

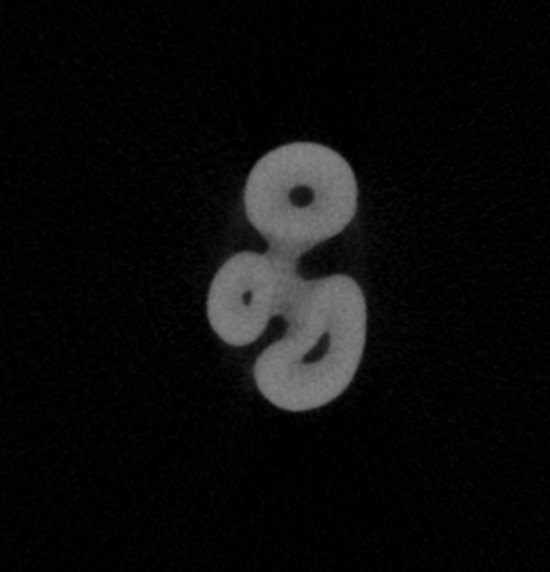

Supplement: S1 File — (ZIP) [file pone.0209698.s001.zip › Skyscan 2211 micro-CT/skyscan2211_0186.tif]

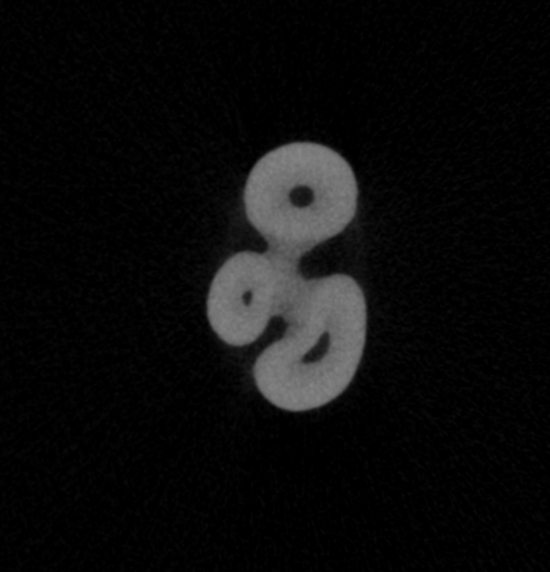

Supplement: S1 File — (ZIP) [file pone.0209698.s001.zip › Skyscan 2211 micro-CT/skyscan2211_0187.tif]

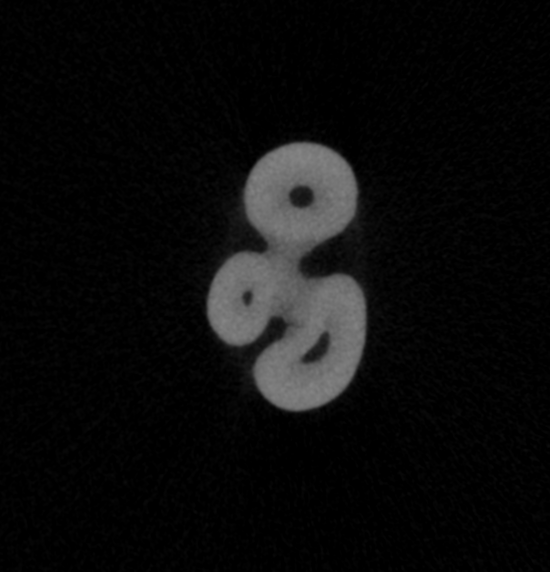

Supplement: S1 File — (ZIP) [file pone.0209698.s001.zip › Skyscan 2211 micro-CT/skyscan2211_0188.tif]

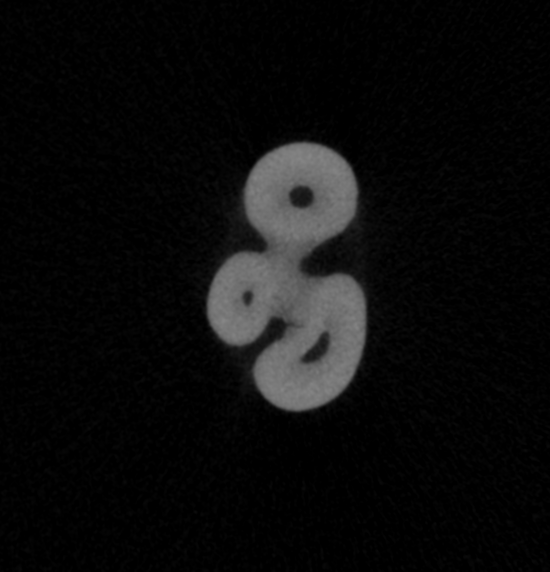

Supplement: S1 File — (ZIP) [file pone.0209698.s001.zip › Skyscan 2211 micro-CT/skyscan2211_0189.tif]

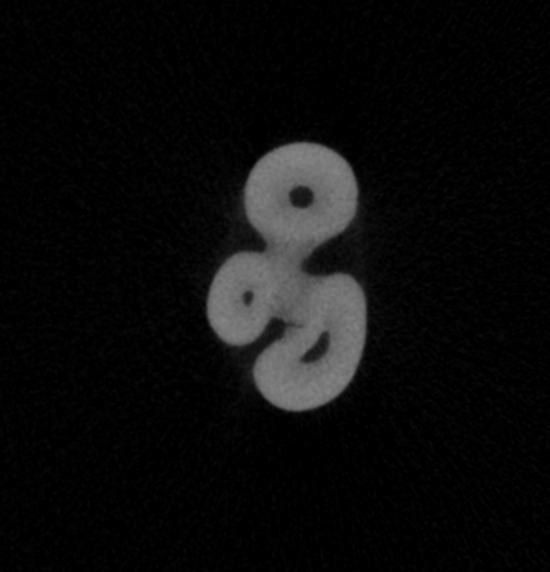

Supplement: S1 File — (ZIP) [file pone.0209698.s001.zip › Skyscan 2211 micro-CT/skyscan2211_0190.tif]

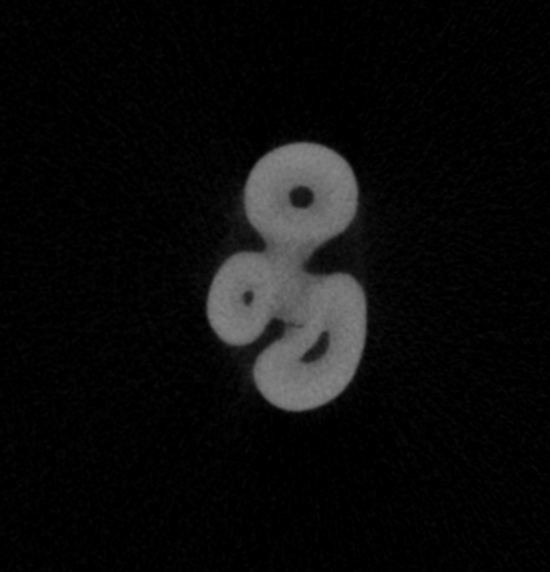

Supplement: S1 File — (ZIP) [file pone.0209698.s001.zip › Skyscan 2211 micro-CT/skyscan2211_0191.tif]

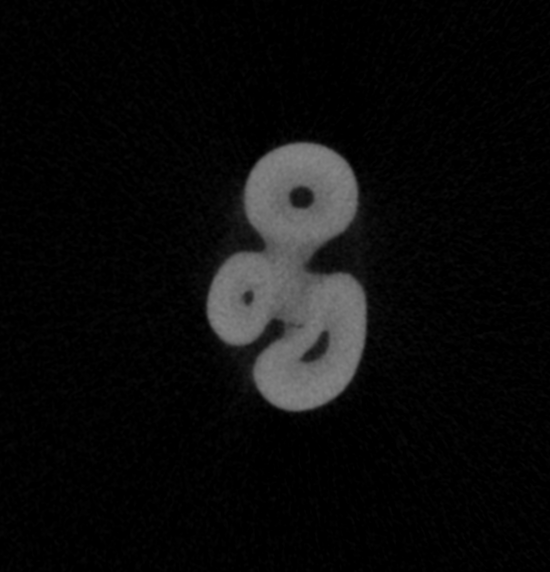

Supplement: S1 File — (ZIP) [file pone.0209698.s001.zip › Skyscan 2211 micro-CT/skyscan2211_0192.tif]

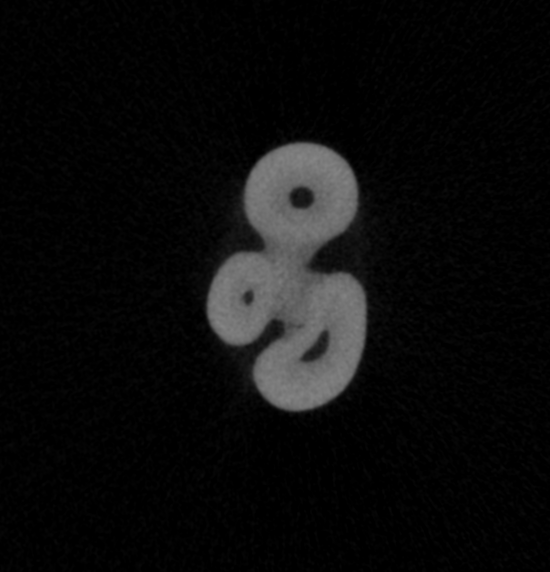

Supplement: S1 File — (ZIP) [file pone.0209698.s001.zip › Skyscan 2211 micro-CT/skyscan2211_0193.tif]

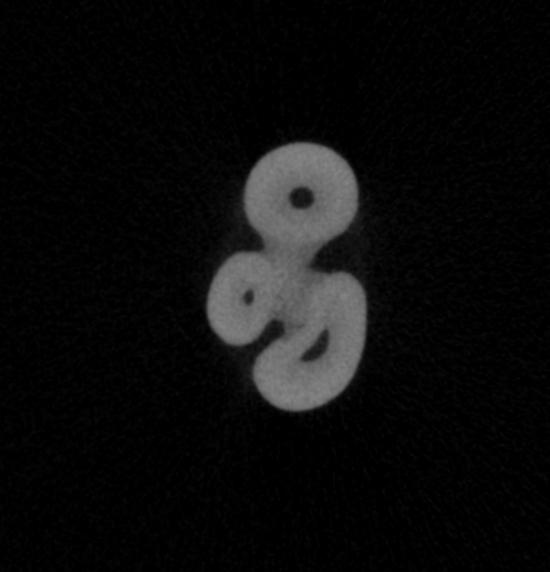

Supplement: S1 File — (ZIP) [file pone.0209698.s001.zip › Skyscan 2211 micro-CT/skyscan2211_0194.tif]

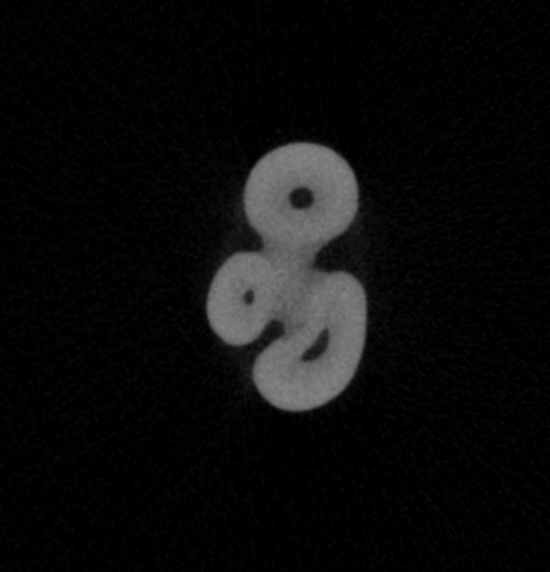

Supplement: S1 File — (ZIP) [file pone.0209698.s001.zip › Skyscan 2211 micro-CT/skyscan2211_0195.tif]

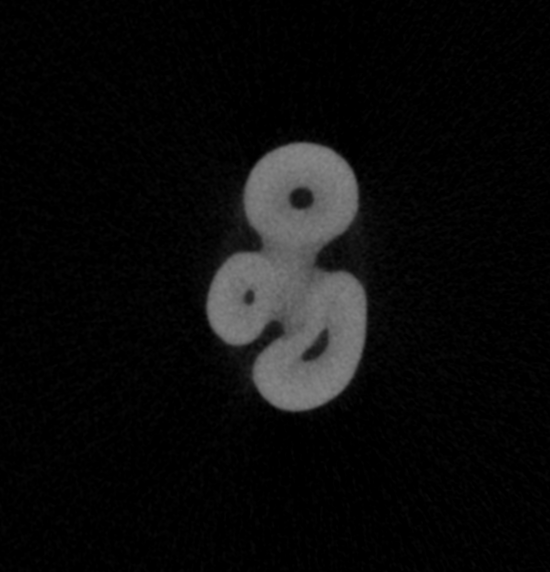

Supplement: S1 File — (ZIP) [file pone.0209698.s001.zip › Skyscan 2211 micro-CT/skyscan2211_0196.tif]

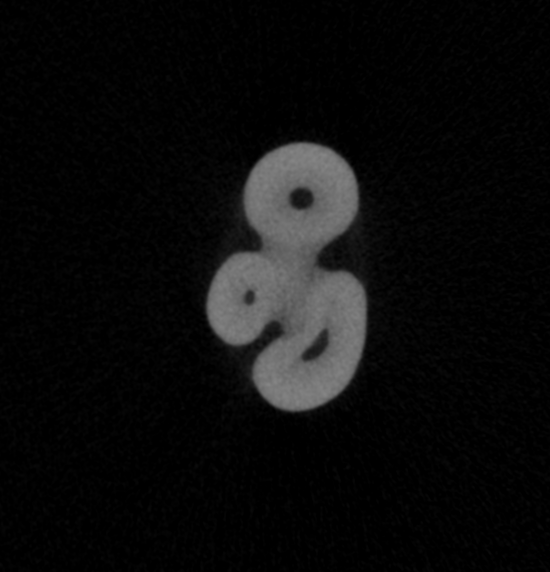

Supplement: S1 File — (ZIP) [file pone.0209698.s001.zip › Skyscan 2211 micro-CT/skyscan2211_0197.tif]

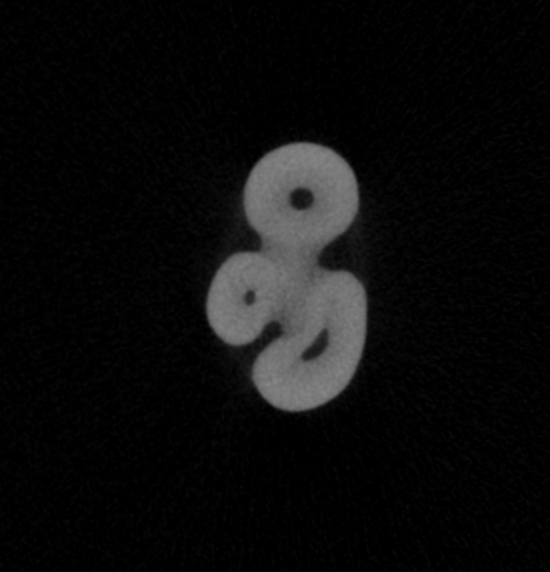

Supplement: S1 File — (ZIP) [file pone.0209698.s001.zip › Skyscan 2211 micro-CT/skyscan2211_0198.tif]

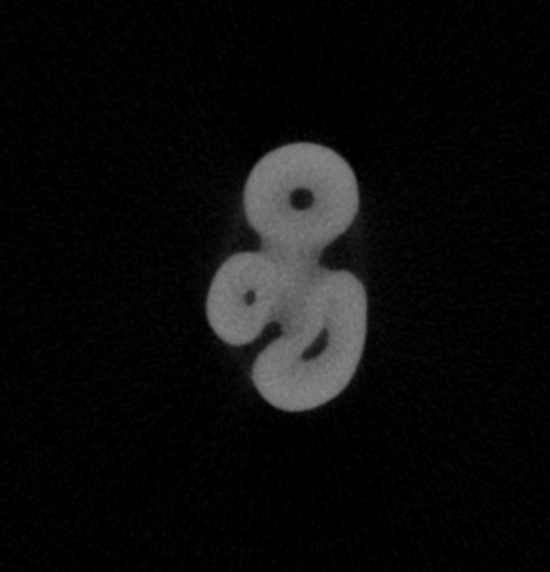

Supplement: S1 File — (ZIP) [file pone.0209698.s001.zip › Skyscan 2211 micro-CT/skyscan2211_0199.tif]

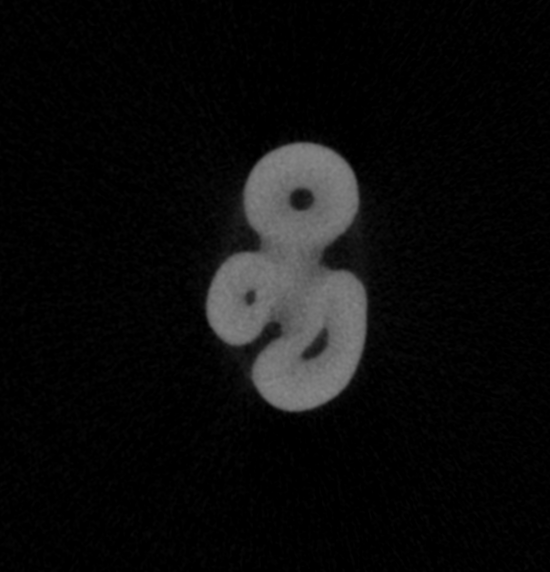

Supplement: S1 File — (ZIP) [file pone.0209698.s001.zip › Skyscan 2211 micro-CT/skyscan2211_0200.tif]

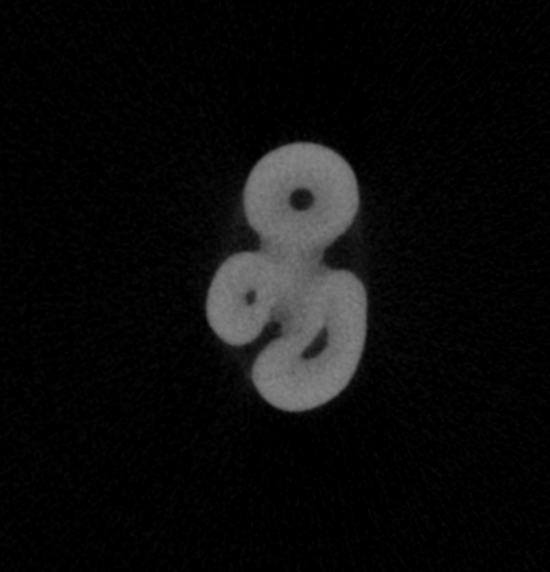

Supplement: S1 File — (ZIP) [file pone.0209698.s001.zip › Skyscan 2211 micro-CT/skyscan2211_0201.tif]
