# Supplementary figures and images for: Self-assembled micro-computed tomography for dental education (part 1 of 4)
Source: PLoS One. 2018 Dec 26;13(12):e0209698. doi: 10.1371/journal.pone.0209698 (PMC6306236; doi:10.1371/journal.pone.0209698)

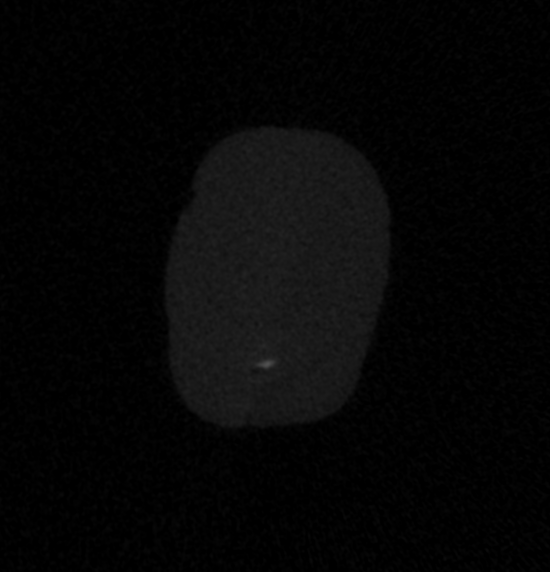

Supplement: S1 File — (ZIP) [file pone.0209698.s001.zip › Skyscan 2211 micro-CT/skyscan2211_0002.tif]

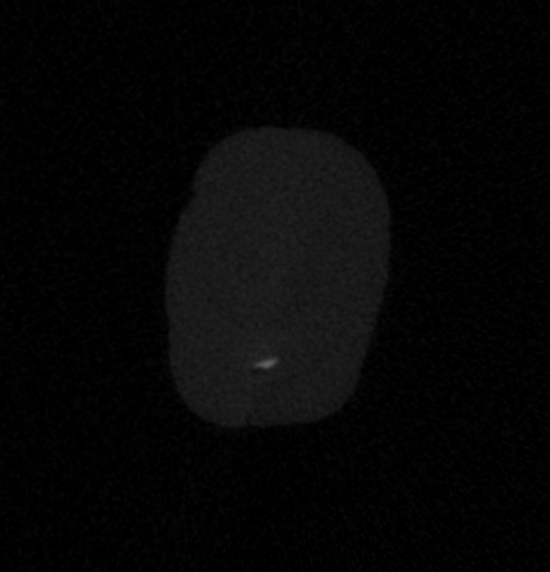

Supplement: S1 File — (ZIP) [file pone.0209698.s001.zip › Skyscan 2211 micro-CT/skyscan2211_0003.tif]

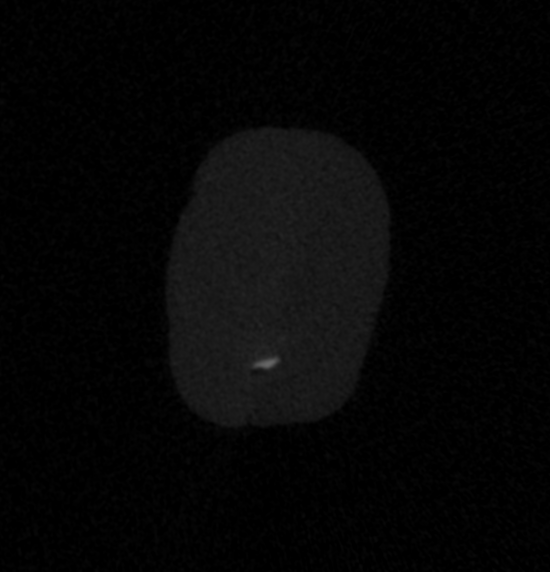

Supplement: S1 File — (ZIP) [file pone.0209698.s001.zip › Skyscan 2211 micro-CT/skyscan2211_0004.tif]

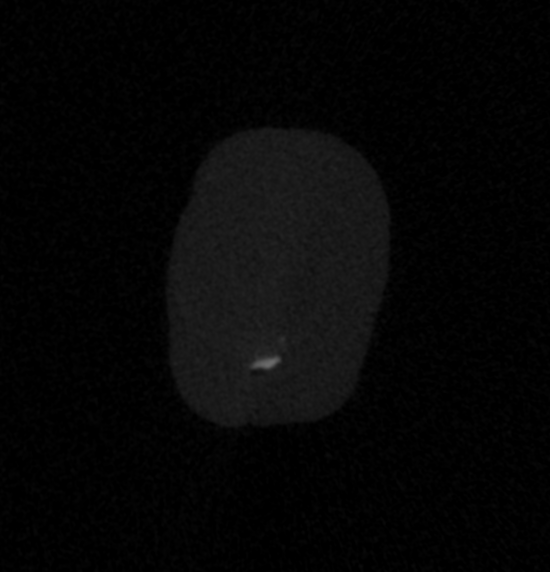

Supplement: S1 File — (ZIP) [file pone.0209698.s001.zip › Skyscan 2211 micro-CT/skyscan2211_0005.tif]

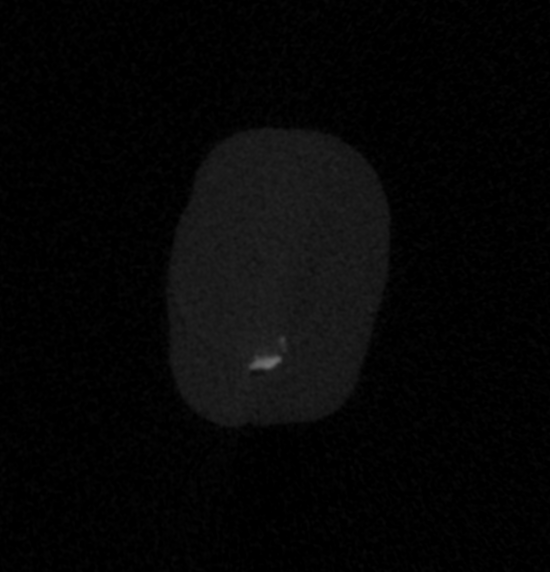

Supplement: S1 File — (ZIP) [file pone.0209698.s001.zip › Skyscan 2211 micro-CT/skyscan2211_0006.tif]

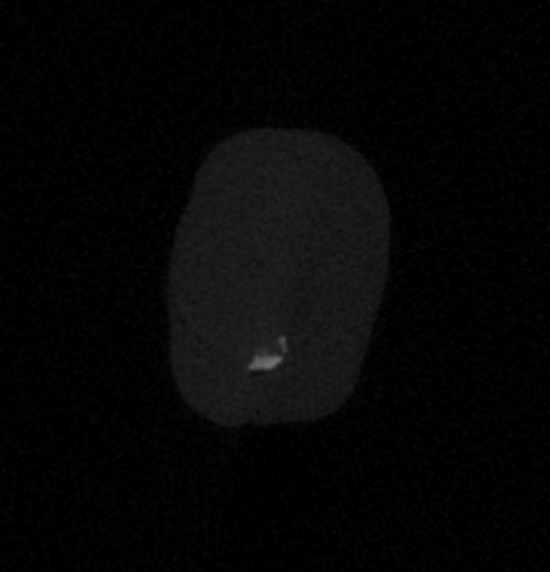

Supplement: S1 File — (ZIP) [file pone.0209698.s001.zip › Skyscan 2211 micro-CT/skyscan2211_0007.tif]

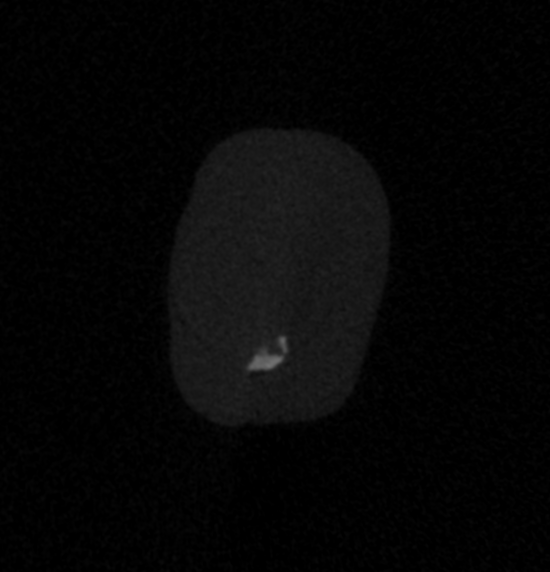

Supplement: S1 File — (ZIP) [file pone.0209698.s001.zip › Skyscan 2211 micro-CT/skyscan2211_0008.tif]

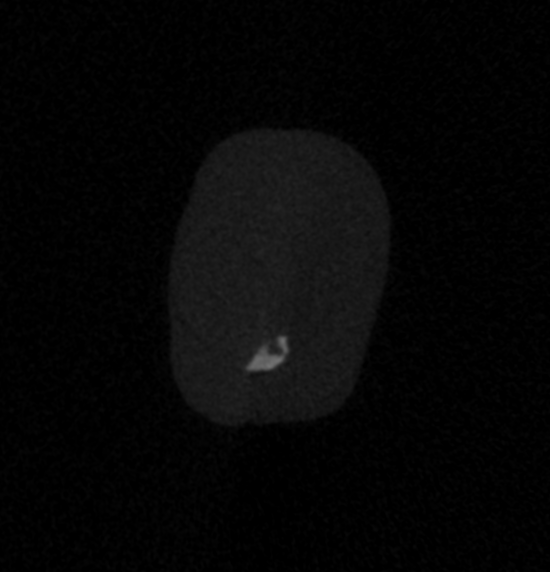

Supplement: S1 File — (ZIP) [file pone.0209698.s001.zip › Skyscan 2211 micro-CT/skyscan2211_0009.tif]

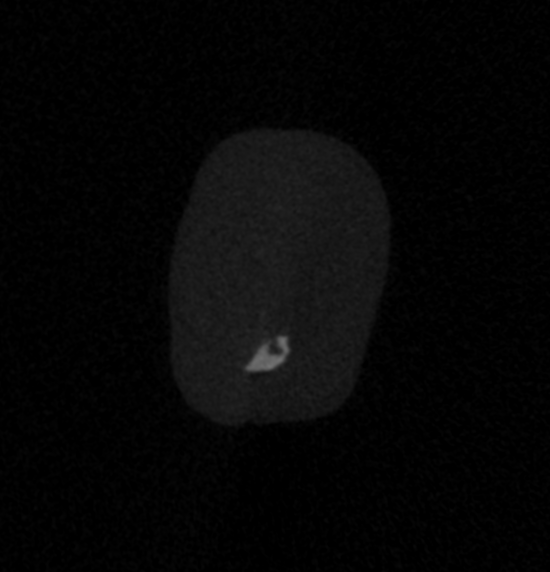

Supplement: S1 File — (ZIP) [file pone.0209698.s001.zip › Skyscan 2211 micro-CT/skyscan2211_0010.tif]

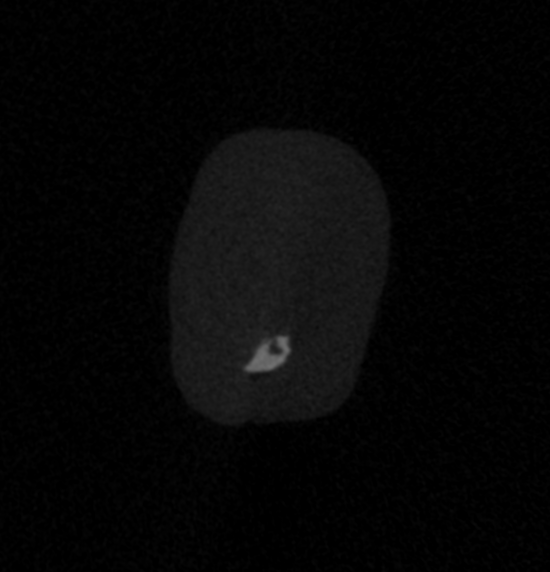

Supplement: S1 File — (ZIP) [file pone.0209698.s001.zip › Skyscan 2211 micro-CT/skyscan2211_0011.tif]

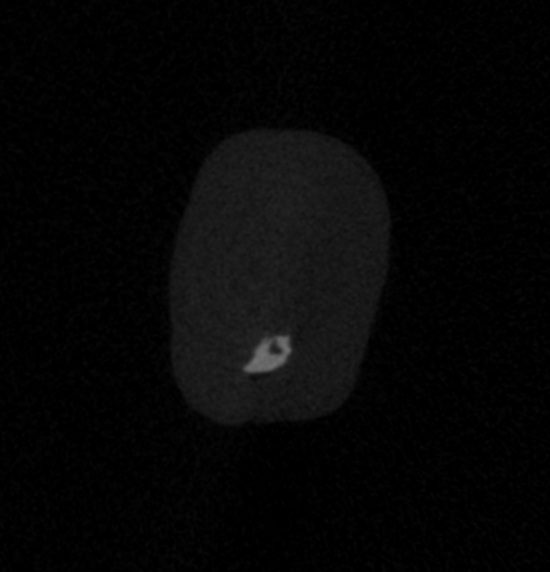

Supplement: S1 File — (ZIP) [file pone.0209698.s001.zip › Skyscan 2211 micro-CT/skyscan2211_0012.tif]

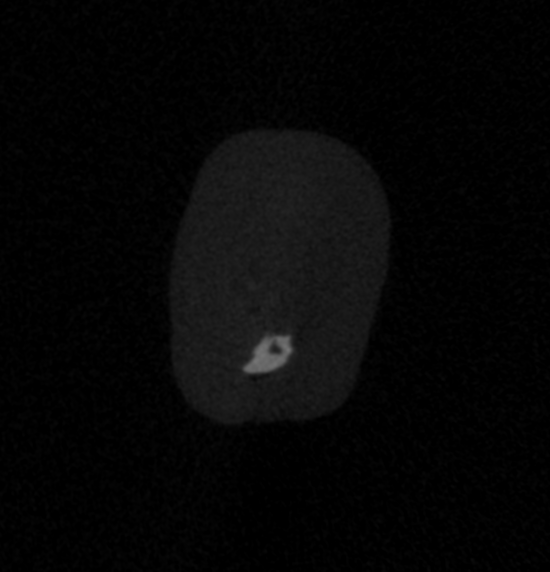

Supplement: S1 File — (ZIP) [file pone.0209698.s001.zip › Skyscan 2211 micro-CT/skyscan2211_0013.tif]

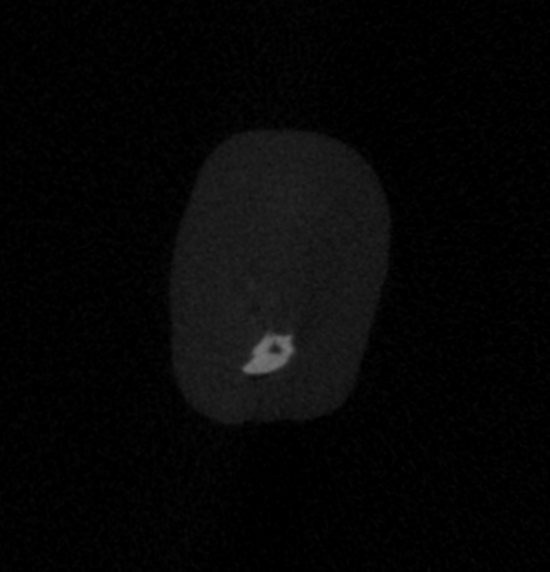

Supplement: S1 File — (ZIP) [file pone.0209698.s001.zip › Skyscan 2211 micro-CT/skyscan2211_0014.tif]

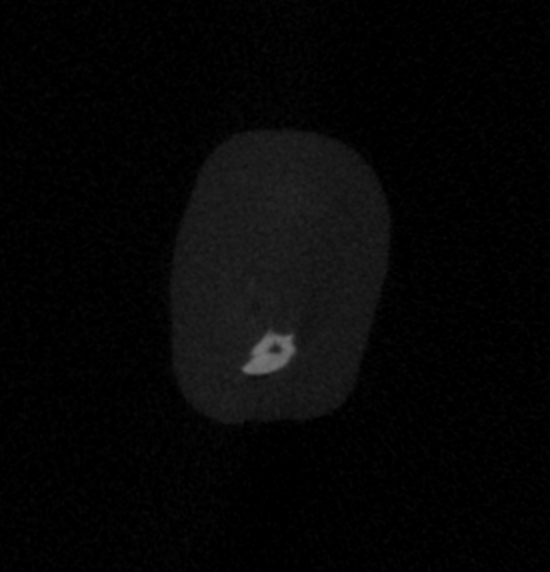

Supplement: S1 File — (ZIP) [file pone.0209698.s001.zip › Skyscan 2211 micro-CT/skyscan2211_0015.tif]

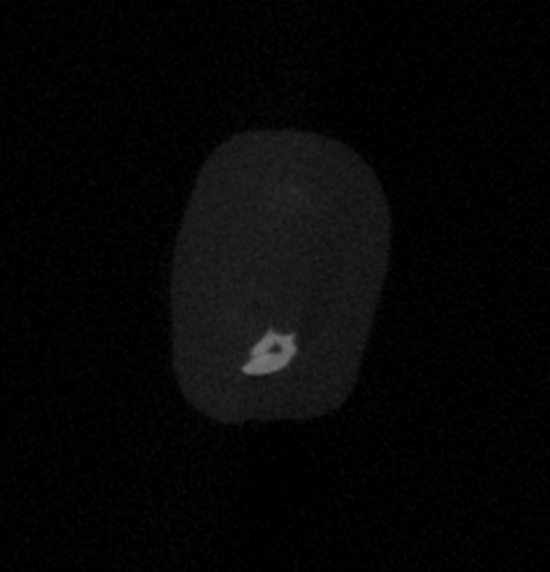

Supplement: S1 File — (ZIP) [file pone.0209698.s001.zip › Skyscan 2211 micro-CT/skyscan2211_0016.tif]

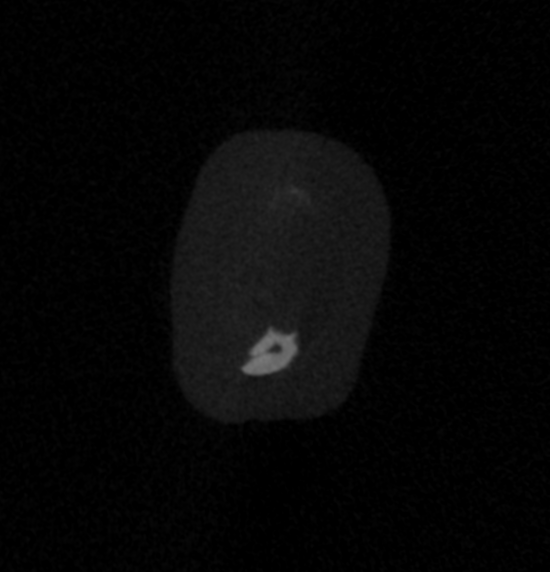

Supplement: S1 File — (ZIP) [file pone.0209698.s001.zip › Skyscan 2211 micro-CT/skyscan2211_0017.tif]

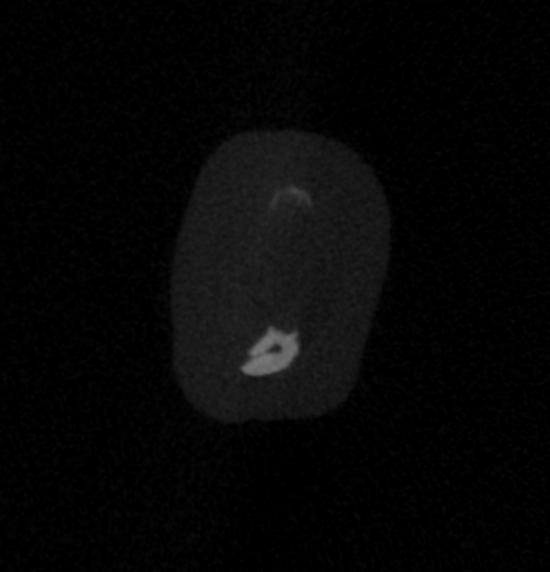

Supplement: S1 File — (ZIP) [file pone.0209698.s001.zip › Skyscan 2211 micro-CT/skyscan2211_0018.tif]

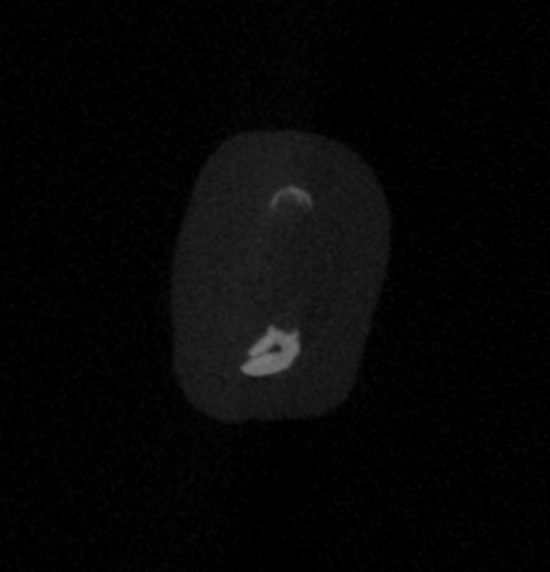

Supplement: S1 File — (ZIP) [file pone.0209698.s001.zip › Skyscan 2211 micro-CT/skyscan2211_0019.tif]

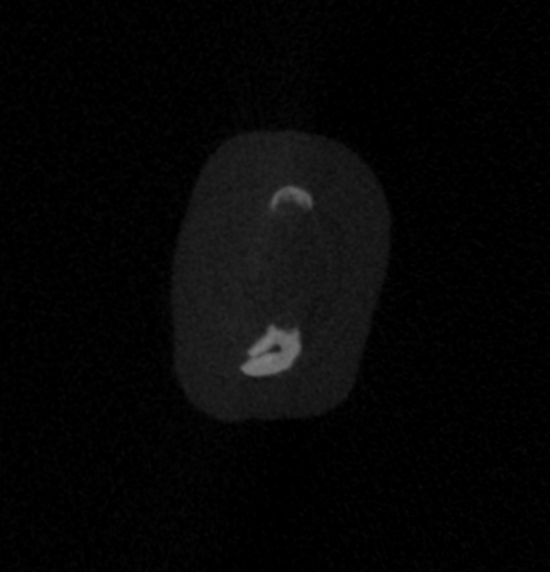

Supplement: S1 File — (ZIP) [file pone.0209698.s001.zip › Skyscan 2211 micro-CT/skyscan2211_0020.tif]

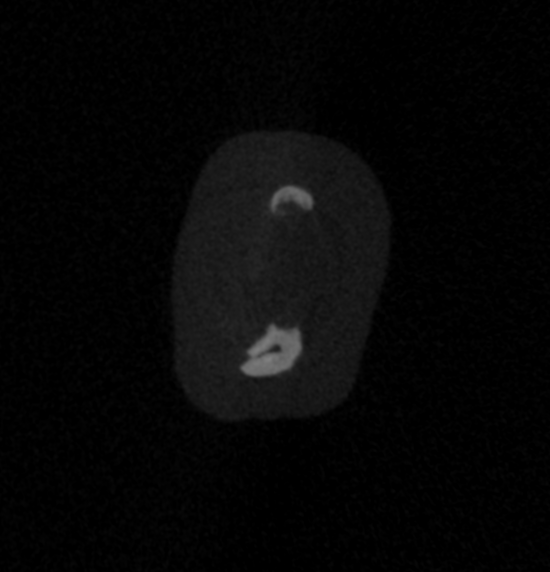

Supplement: S1 File — (ZIP) [file pone.0209698.s001.zip › Skyscan 2211 micro-CT/skyscan2211_0021.tif]

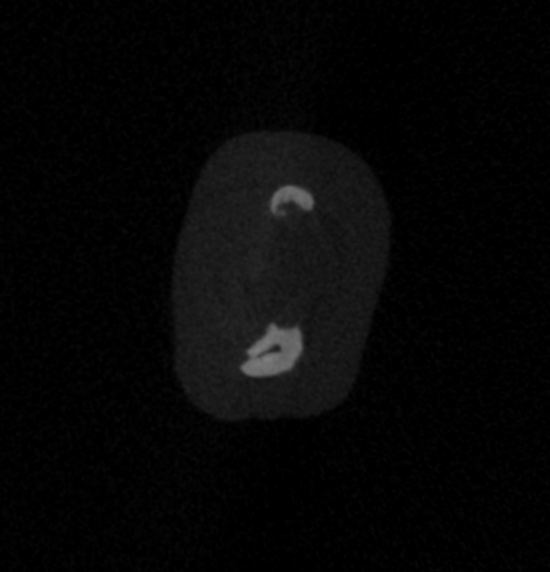

Supplement: S1 File — (ZIP) [file pone.0209698.s001.zip › Skyscan 2211 micro-CT/skyscan2211_0022.tif]

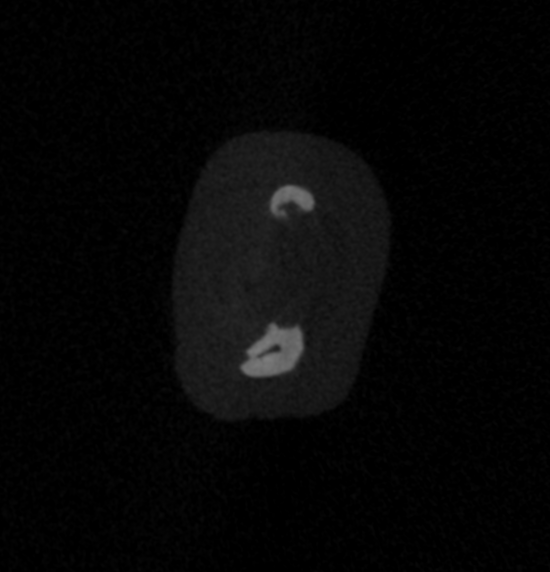

Supplement: S1 File — (ZIP) [file pone.0209698.s001.zip › Skyscan 2211 micro-CT/skyscan2211_0023.tif]

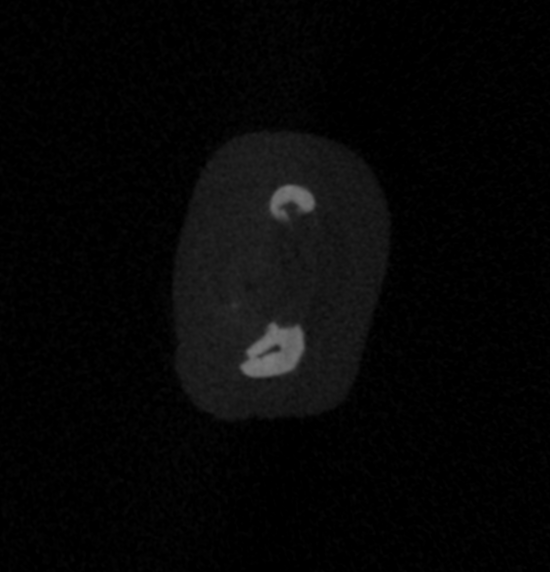

Supplement: S1 File — (ZIP) [file pone.0209698.s001.zip › Skyscan 2211 micro-CT/skyscan2211_0024.tif]

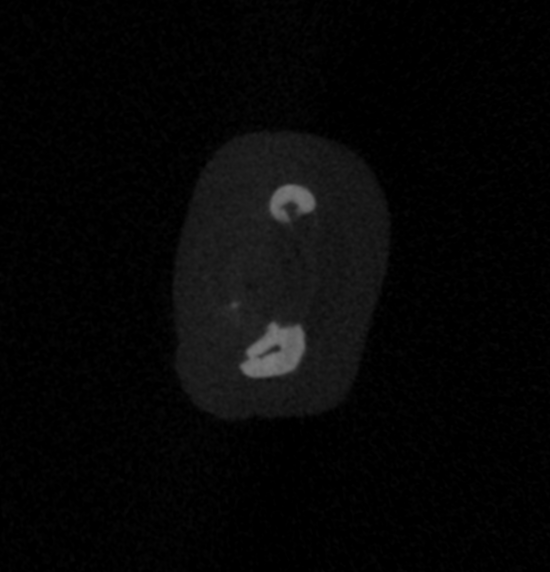

Supplement: S1 File — (ZIP) [file pone.0209698.s001.zip › Skyscan 2211 micro-CT/skyscan2211_0025.tif]

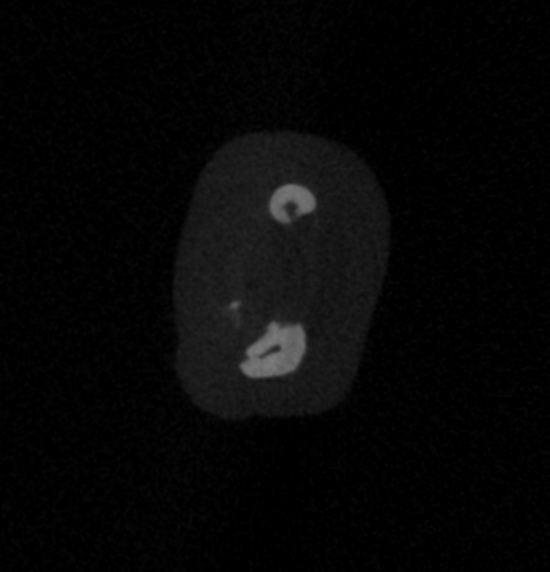

Supplement: S1 File — (ZIP) [file pone.0209698.s001.zip › Skyscan 2211 micro-CT/skyscan2211_0026.tif]

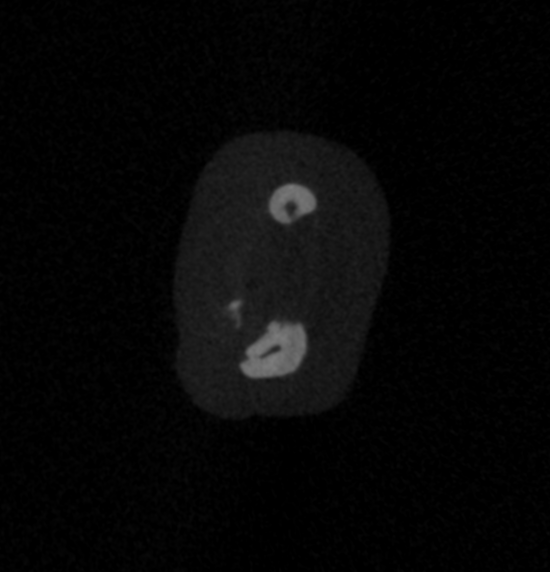

Supplement: S1 File — (ZIP) [file pone.0209698.s001.zip › Skyscan 2211 micro-CT/skyscan2211_0027.tif]

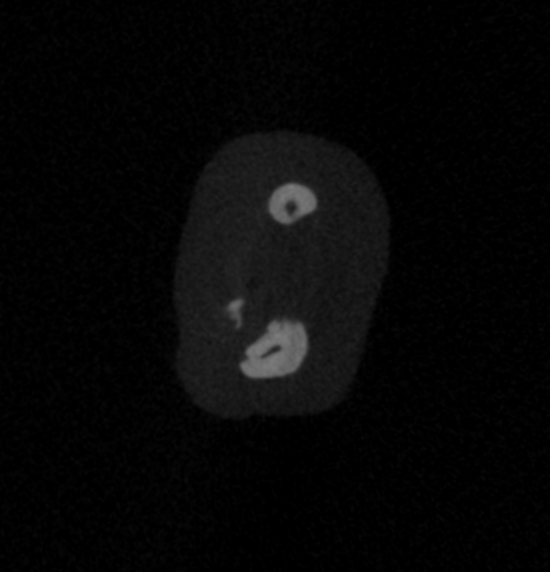

Supplement: S1 File — (ZIP) [file pone.0209698.s001.zip › Skyscan 2211 micro-CT/skyscan2211_0028.tif]

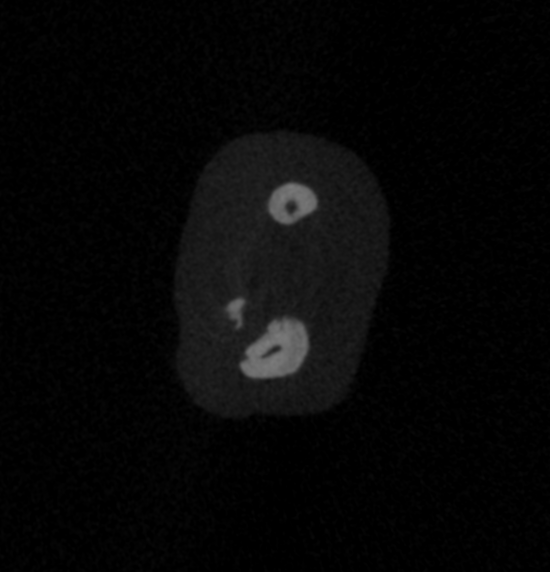

Supplement: S1 File — (ZIP) [file pone.0209698.s001.zip › Skyscan 2211 micro-CT/skyscan2211_0029.tif]

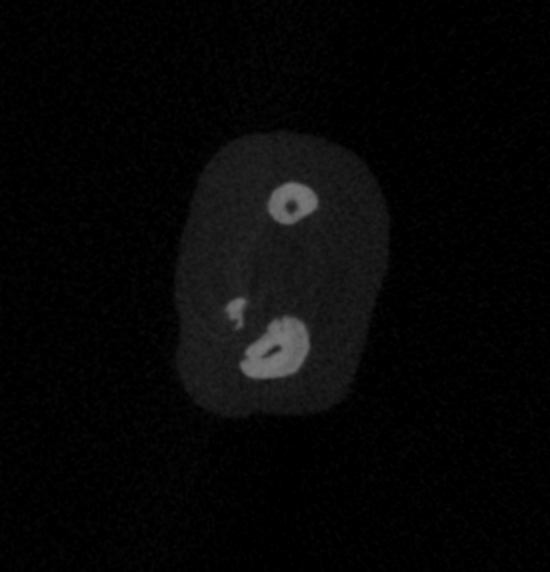

Supplement: S1 File — (ZIP) [file pone.0209698.s001.zip › Skyscan 2211 micro-CT/skyscan2211_0030.tif]

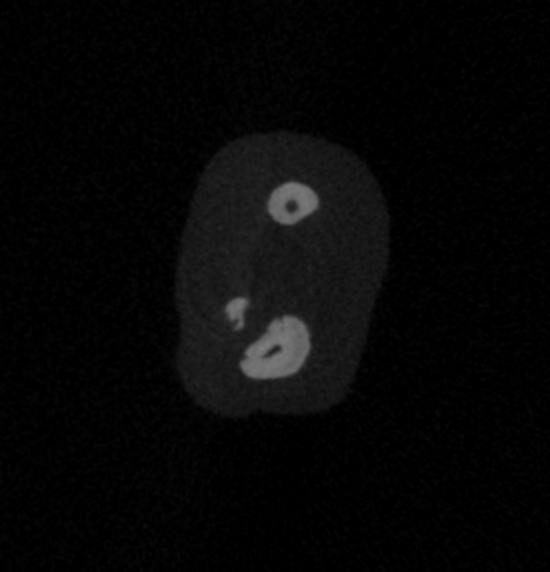

Supplement: S1 File — (ZIP) [file pone.0209698.s001.zip › Skyscan 2211 micro-CT/skyscan2211_0031.tif]

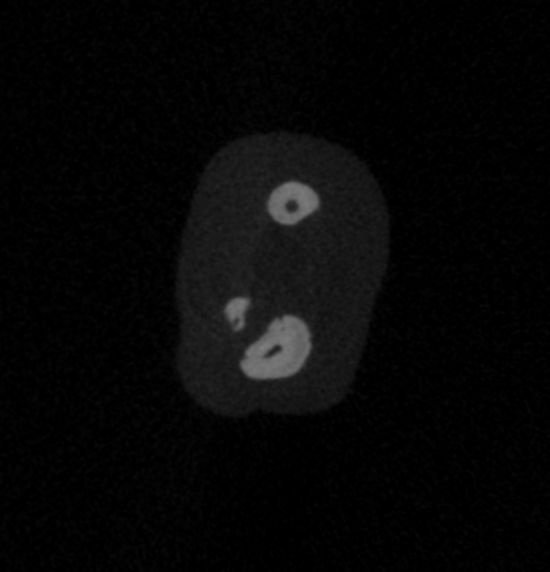

Supplement: S1 File — (ZIP) [file pone.0209698.s001.zip › Skyscan 2211 micro-CT/skyscan2211_0032.tif]

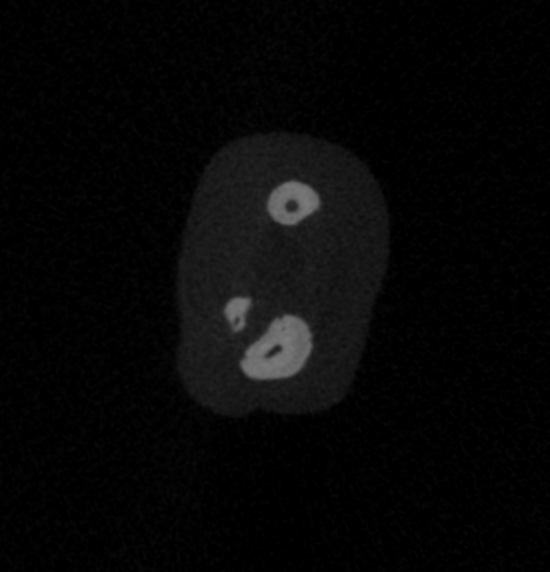

Supplement: S1 File — (ZIP) [file pone.0209698.s001.zip › Skyscan 2211 micro-CT/skyscan2211_0033.tif]

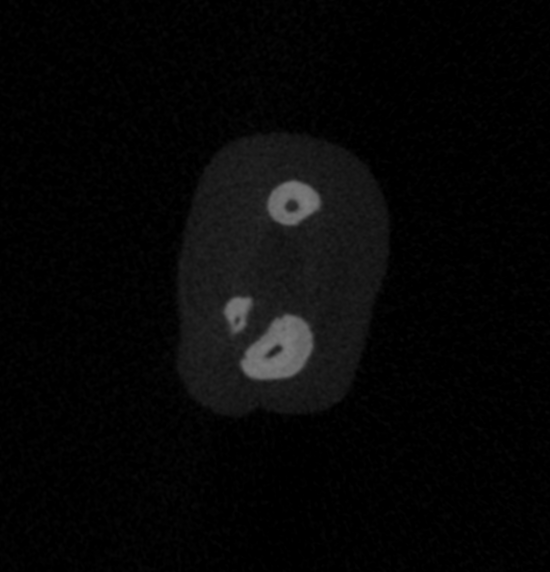

Supplement: S1 File — (ZIP) [file pone.0209698.s001.zip › Skyscan 2211 micro-CT/skyscan2211_0034.tif]

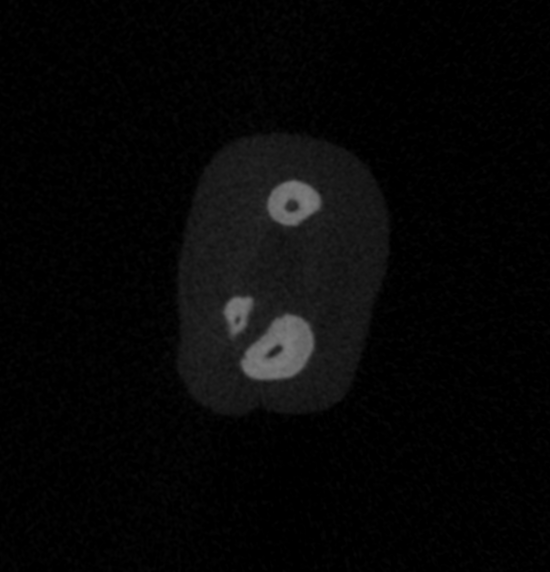

Supplement: S1 File — (ZIP) [file pone.0209698.s001.zip › Skyscan 2211 micro-CT/skyscan2211_0035.tif]

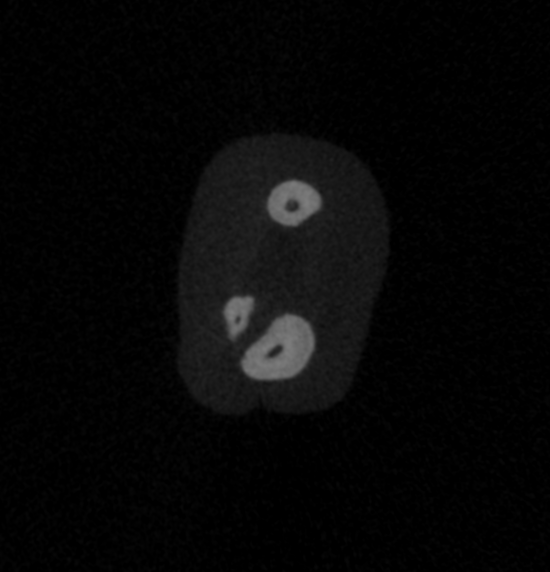

Supplement: S1 File — (ZIP) [file pone.0209698.s001.zip › Skyscan 2211 micro-CT/skyscan2211_0036.tif]

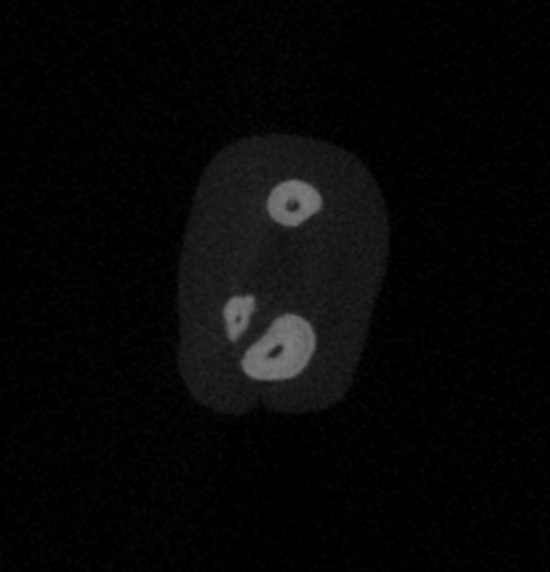

Supplement: S1 File — (ZIP) [file pone.0209698.s001.zip › Skyscan 2211 micro-CT/skyscan2211_0037.tif]

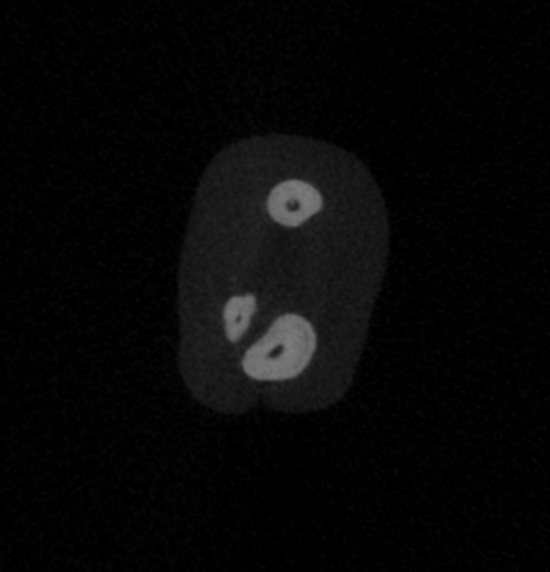

Supplement: S1 File — (ZIP) [file pone.0209698.s001.zip › Skyscan 2211 micro-CT/skyscan2211_0038.tif]

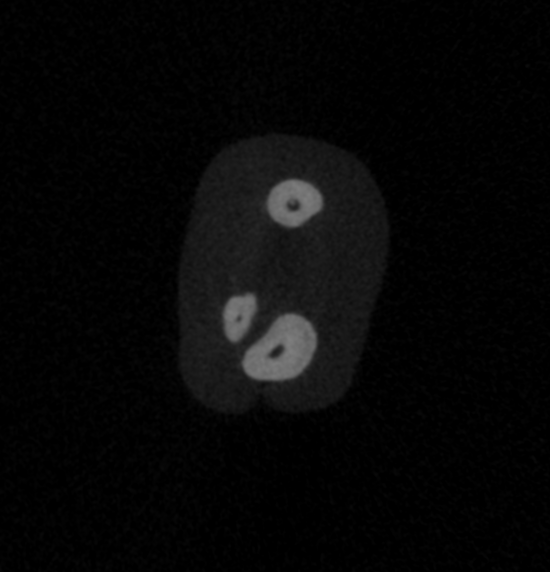

Supplement: S1 File — (ZIP) [file pone.0209698.s001.zip › Skyscan 2211 micro-CT/skyscan2211_0039.tif]

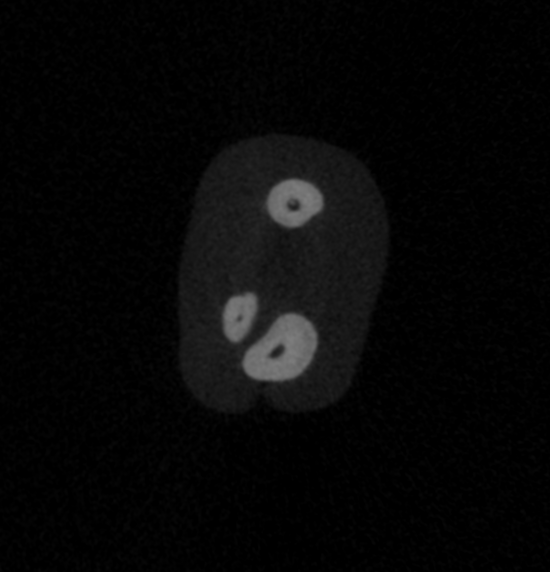

Supplement: S1 File — (ZIP) [file pone.0209698.s001.zip › Skyscan 2211 micro-CT/skyscan2211_0040.tif]

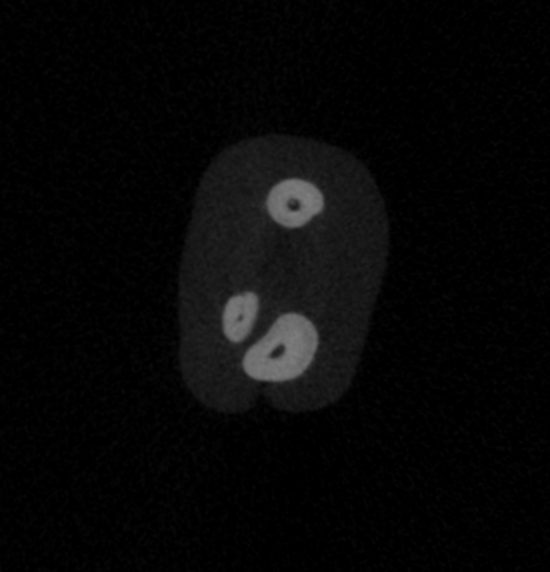

Supplement: S1 File — (ZIP) [file pone.0209698.s001.zip › Skyscan 2211 micro-CT/skyscan2211_0041.tif]

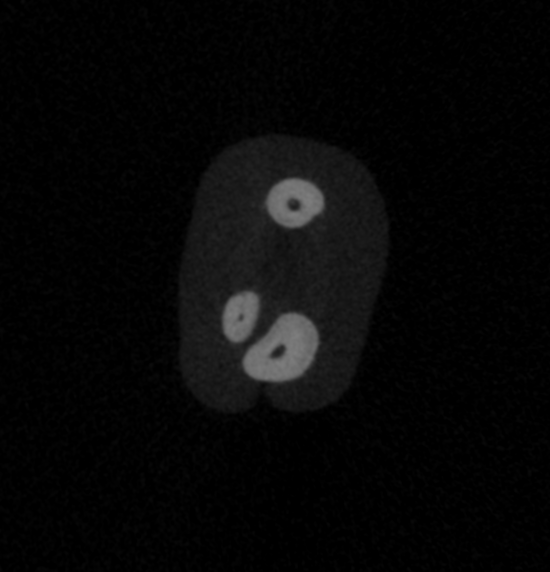

Supplement: S1 File — (ZIP) [file pone.0209698.s001.zip › Skyscan 2211 micro-CT/skyscan2211_0042.tif]

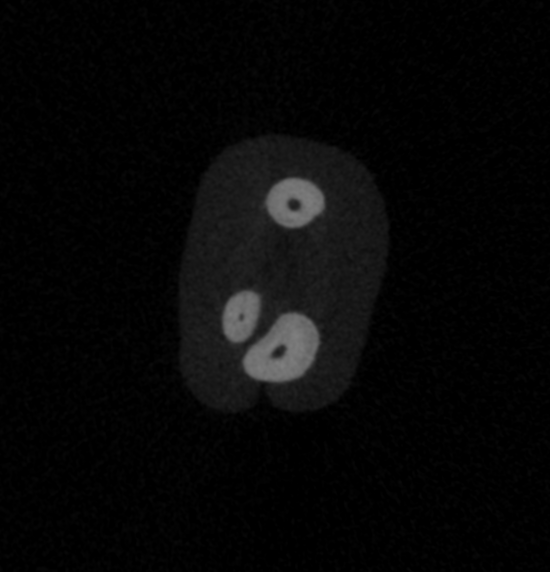

Supplement: S1 File — (ZIP) [file pone.0209698.s001.zip › Skyscan 2211 micro-CT/skyscan2211_0043.tif]

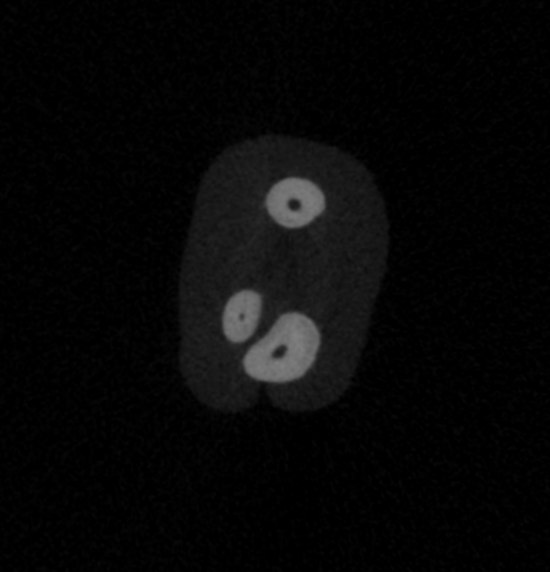

Supplement: S1 File — (ZIP) [file pone.0209698.s001.zip › Skyscan 2211 micro-CT/skyscan2211_0044.tif]

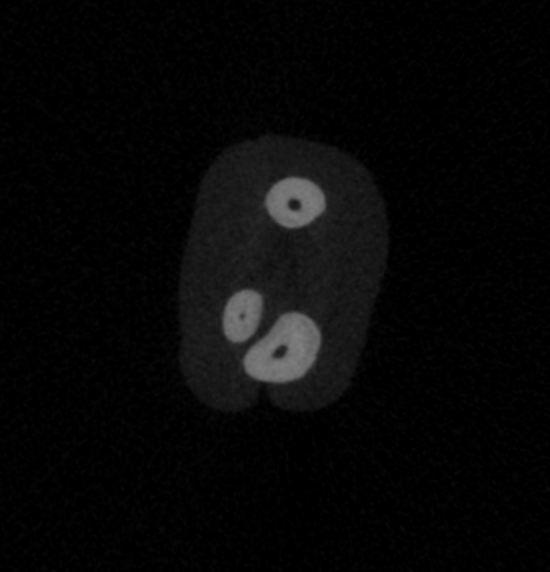

Supplement: S1 File — (ZIP) [file pone.0209698.s001.zip › Skyscan 2211 micro-CT/skyscan2211_0045.tif]

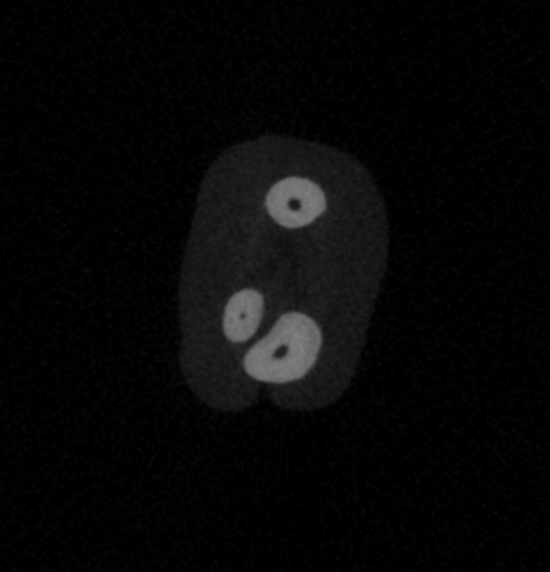

Supplement: S1 File — (ZIP) [file pone.0209698.s001.zip › Skyscan 2211 micro-CT/skyscan2211_0046.tif]

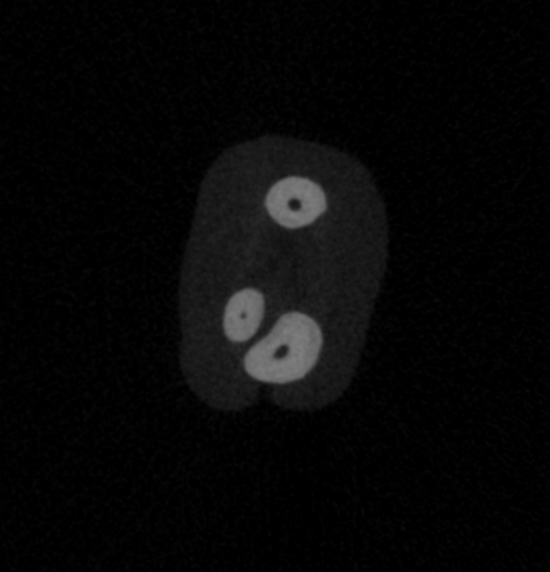

Supplement: S1 File — (ZIP) [file pone.0209698.s001.zip › Skyscan 2211 micro-CT/skyscan2211_0047.tif]

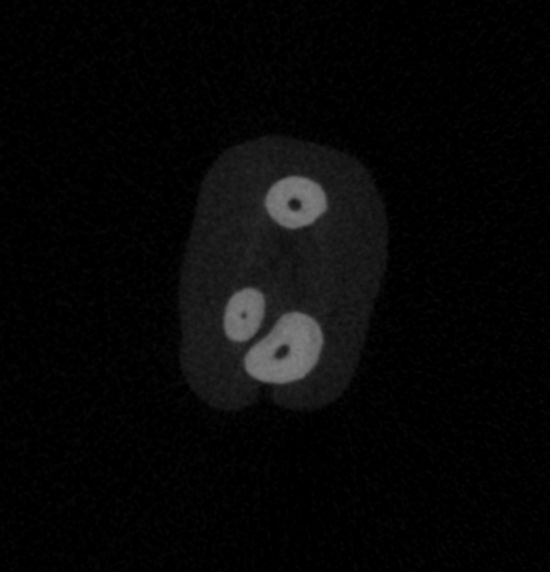

Supplement: S1 File — (ZIP) [file pone.0209698.s001.zip › Skyscan 2211 micro-CT/skyscan2211_0048.tif]

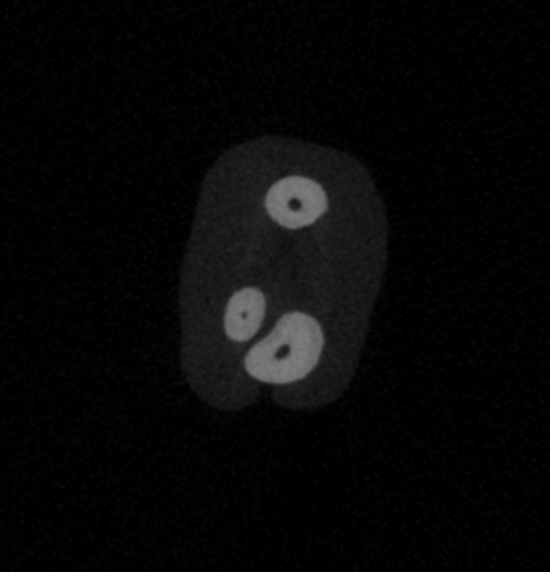

Supplement: S1 File — (ZIP) [file pone.0209698.s001.zip › Skyscan 2211 micro-CT/skyscan2211_0049.tif]

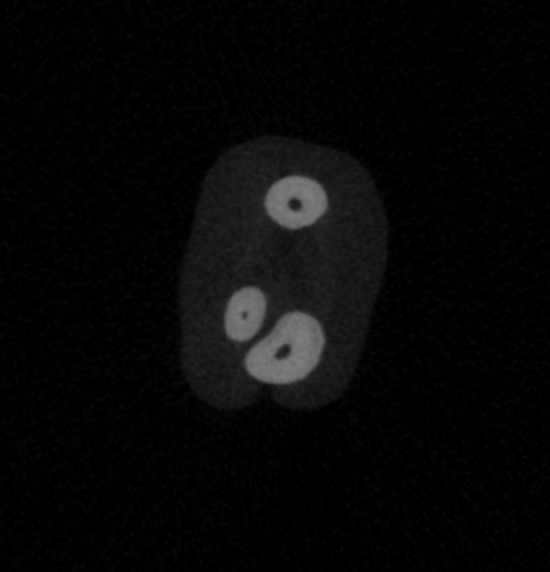

Supplement: S1 File — (ZIP) [file pone.0209698.s001.zip › Skyscan 2211 micro-CT/skyscan2211_0050.tif]

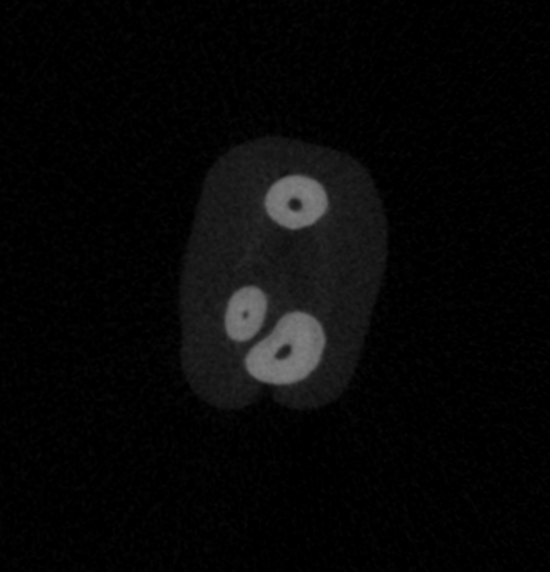

Supplement: S1 File — (ZIP) [file pone.0209698.s001.zip › Skyscan 2211 micro-CT/skyscan2211_0051.tif]

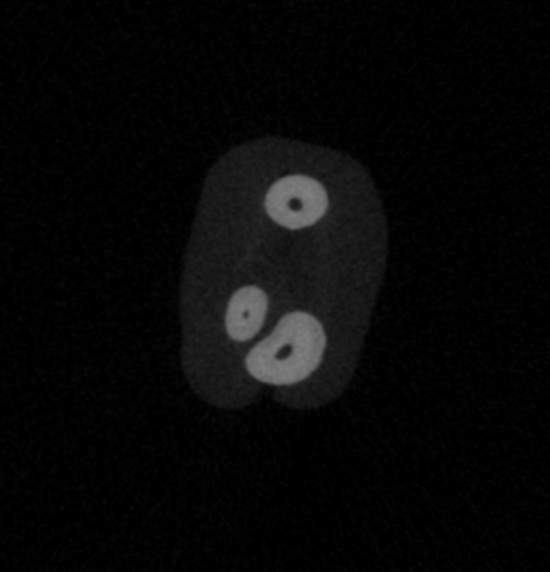

Supplement: S1 File — (ZIP) [file pone.0209698.s001.zip › Skyscan 2211 micro-CT/skyscan2211_0052.tif]

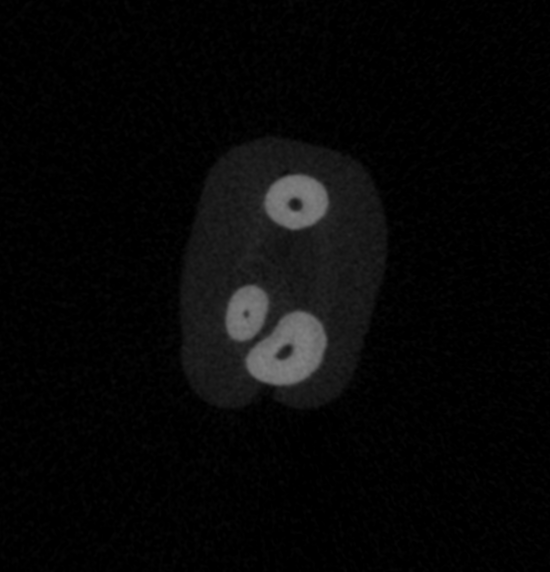

Supplement: S1 File — (ZIP) [file pone.0209698.s001.zip › Skyscan 2211 micro-CT/skyscan2211_0053.tif]

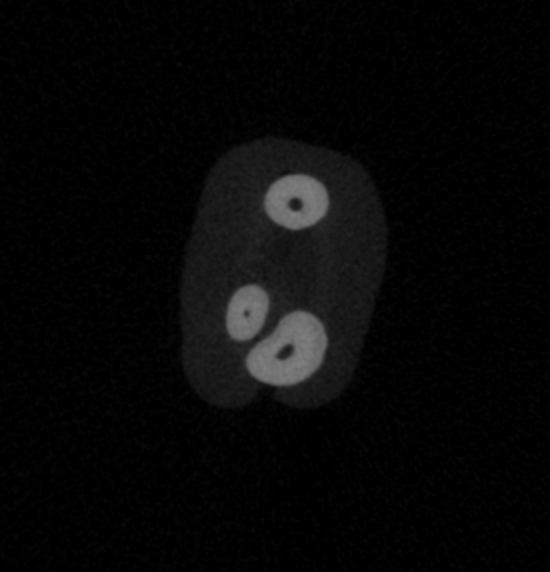

Supplement: S1 File — (ZIP) [file pone.0209698.s001.zip › Skyscan 2211 micro-CT/skyscan2211_0054.tif]

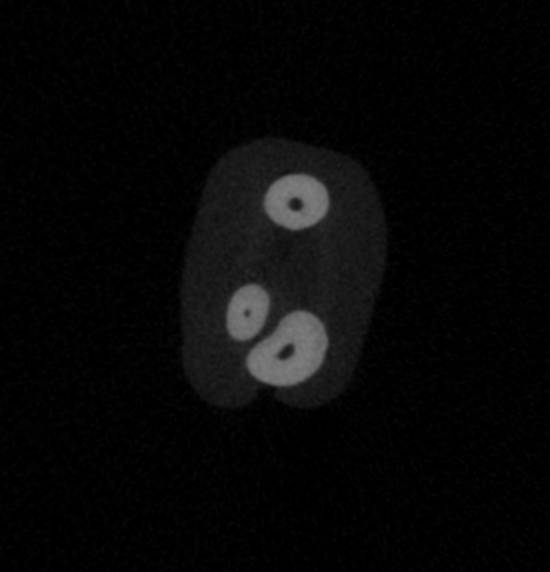

Supplement: S1 File — (ZIP) [file pone.0209698.s001.zip › Skyscan 2211 micro-CT/skyscan2211_0055.tif]

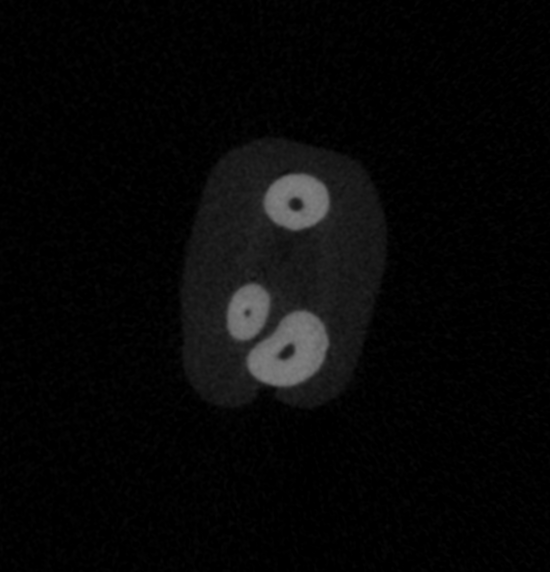

Supplement: S1 File — (ZIP) [file pone.0209698.s001.zip › Skyscan 2211 micro-CT/skyscan2211_0056.tif]

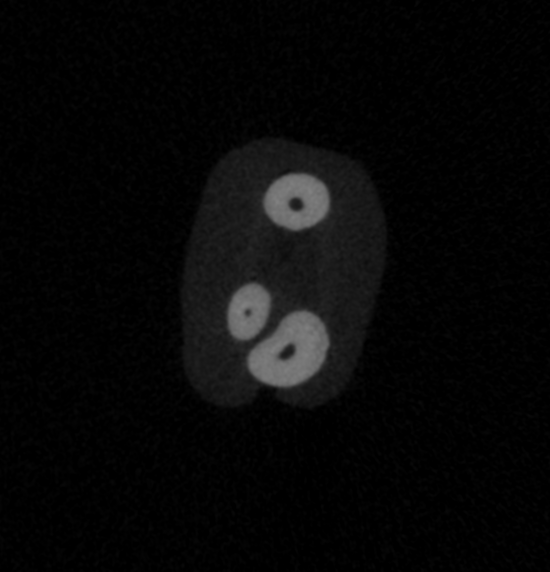

Supplement: S1 File — (ZIP) [file pone.0209698.s001.zip › Skyscan 2211 micro-CT/skyscan2211_0057.tif]

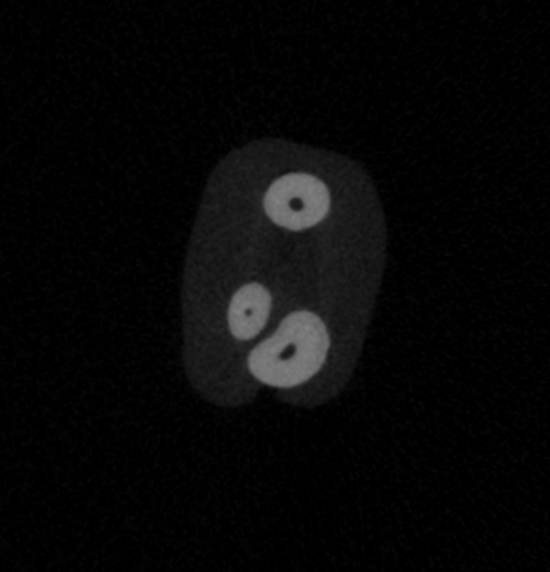

Supplement: S1 File — (ZIP) [file pone.0209698.s001.zip › Skyscan 2211 micro-CT/skyscan2211_0058.tif]

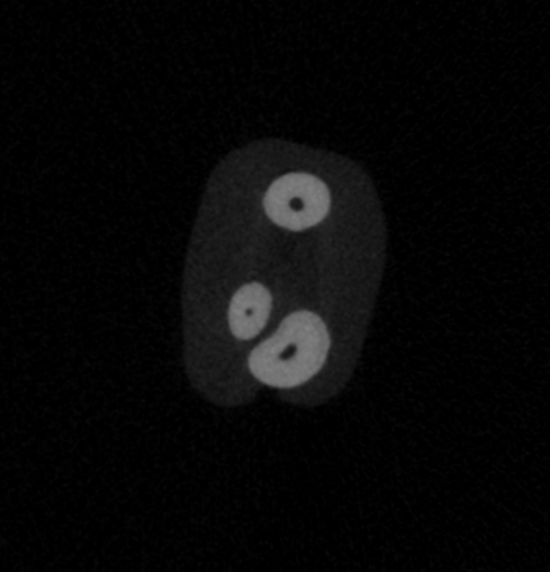

Supplement: S1 File — (ZIP) [file pone.0209698.s001.zip › Skyscan 2211 micro-CT/skyscan2211_0059.tif]

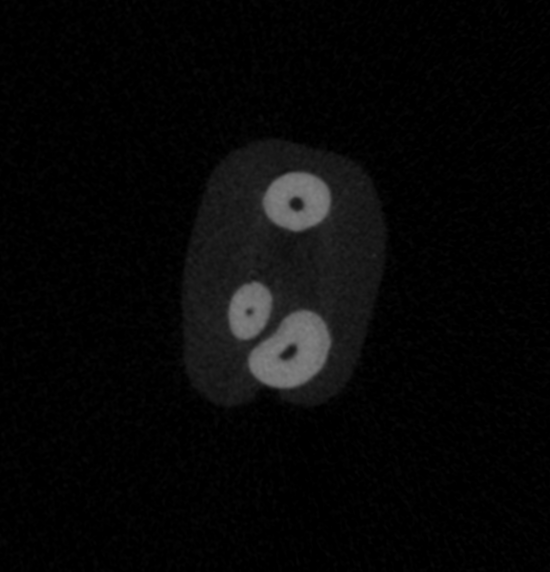

Supplement: S1 File — (ZIP) [file pone.0209698.s001.zip › Skyscan 2211 micro-CT/skyscan2211_0060.tif]

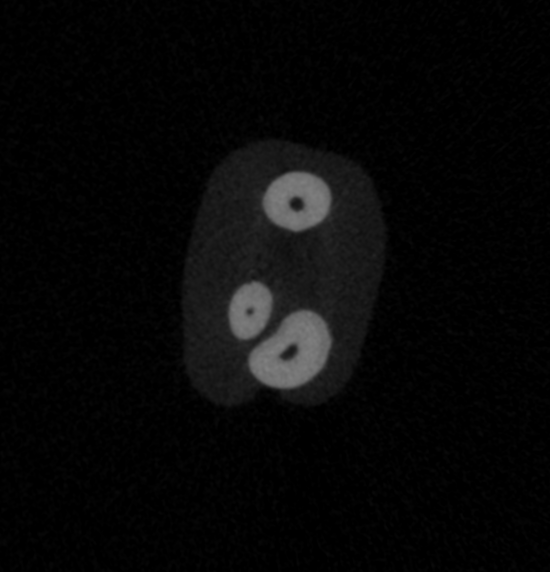

Supplement: S1 File — (ZIP) [file pone.0209698.s001.zip › Skyscan 2211 micro-CT/skyscan2211_0061.tif]

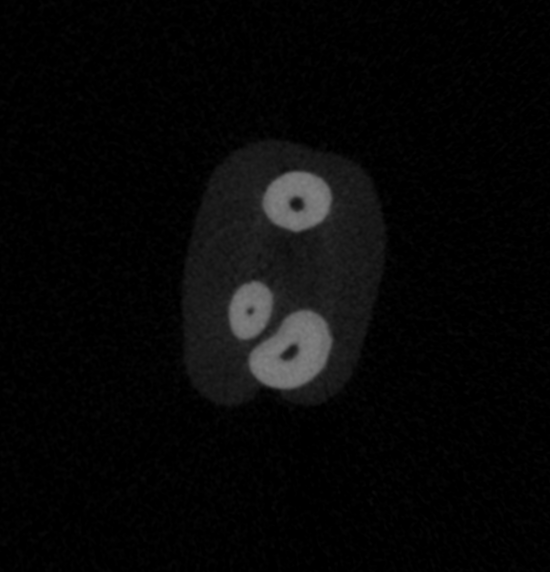

Supplement: S1 File — (ZIP) [file pone.0209698.s001.zip › Skyscan 2211 micro-CT/skyscan2211_0062.tif]

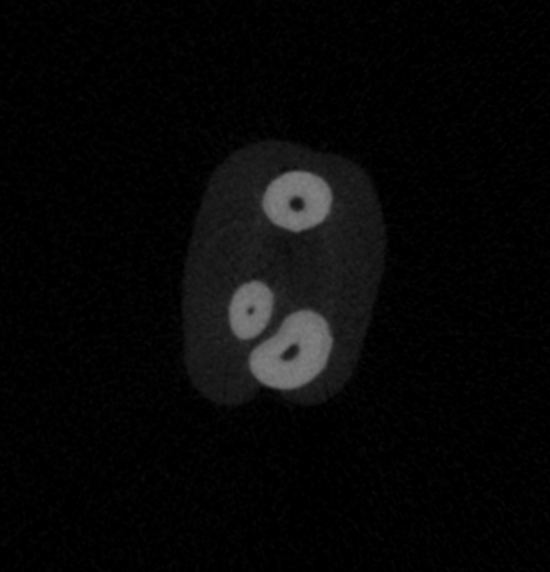

Supplement: S1 File — (ZIP) [file pone.0209698.s001.zip › Skyscan 2211 micro-CT/skyscan2211_0063.tif]

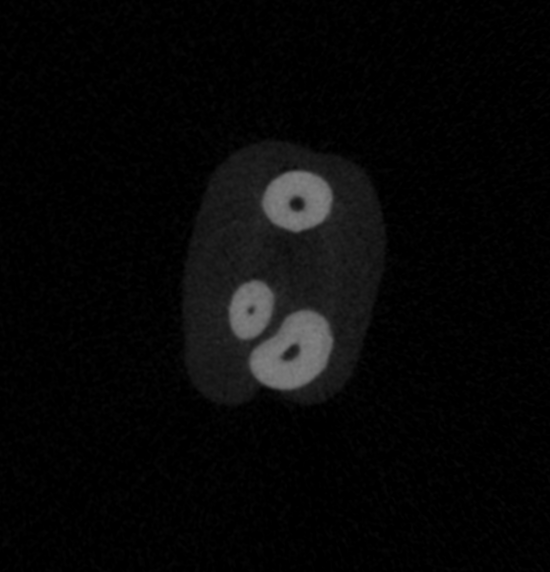

Supplement: S1 File — (ZIP) [file pone.0209698.s001.zip › Skyscan 2211 micro-CT/skyscan2211_0064.tif]

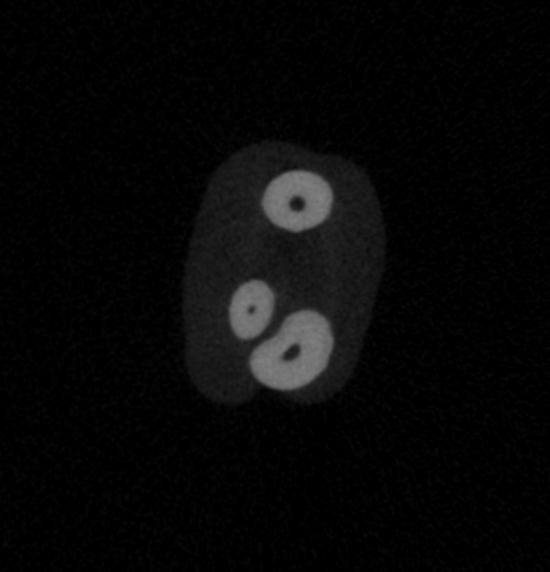

Supplement: S1 File — (ZIP) [file pone.0209698.s001.zip › Skyscan 2211 micro-CT/skyscan2211_0065.tif]

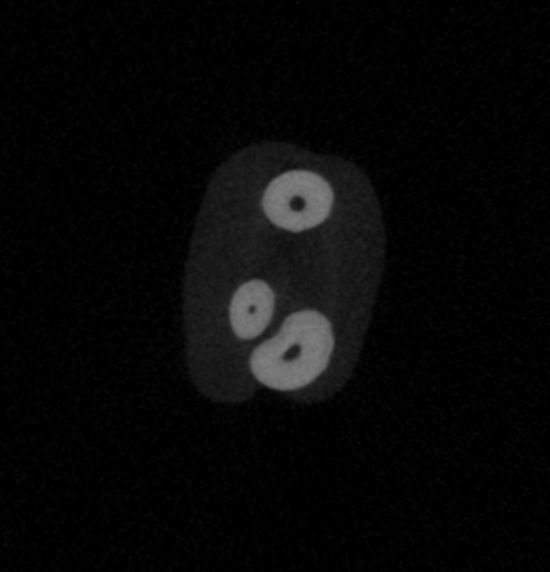

Supplement: S1 File — (ZIP) [file pone.0209698.s001.zip › Skyscan 2211 micro-CT/skyscan2211_0066.tif]

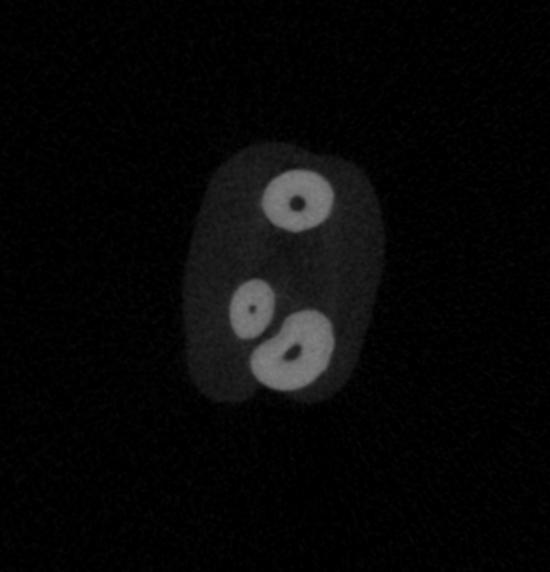

Supplement: S1 File — (ZIP) [file pone.0209698.s001.zip › Skyscan 2211 micro-CT/skyscan2211_0067.tif]

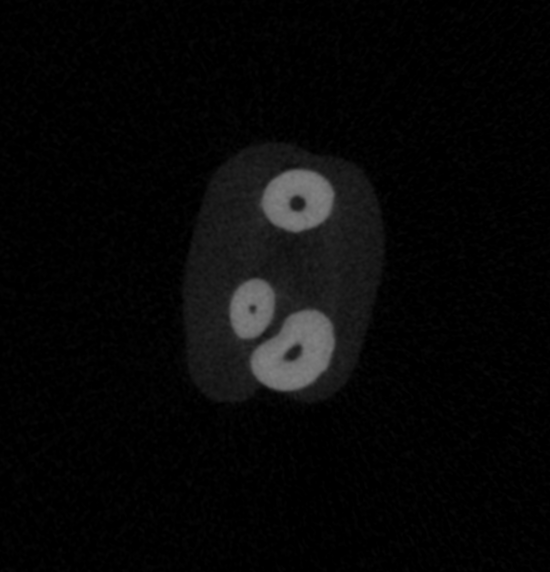

Supplement: S1 File — (ZIP) [file pone.0209698.s001.zip › Skyscan 2211 micro-CT/skyscan2211_0068.tif]

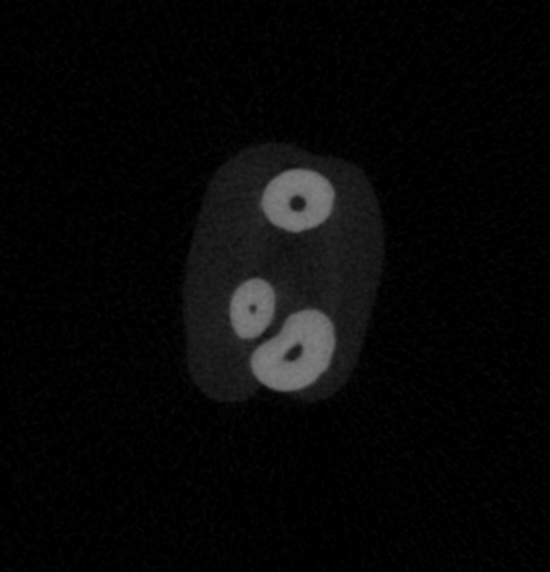

Supplement: S1 File — (ZIP) [file pone.0209698.s001.zip › Skyscan 2211 micro-CT/skyscan2211_0069.tif]

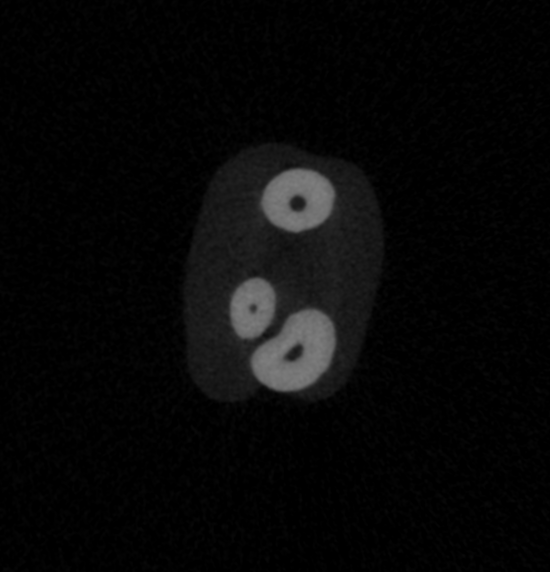

Supplement: S1 File — (ZIP) [file pone.0209698.s001.zip › Skyscan 2211 micro-CT/skyscan2211_0070.tif]

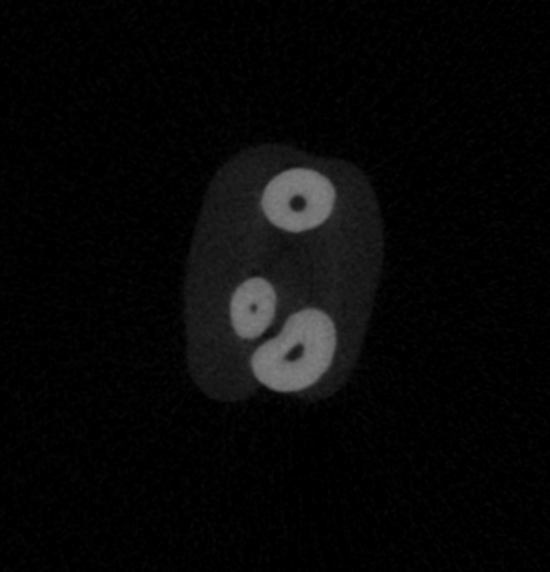

Supplement: S1 File — (ZIP) [file pone.0209698.s001.zip › Skyscan 2211 micro-CT/skyscan2211_0071.tif]

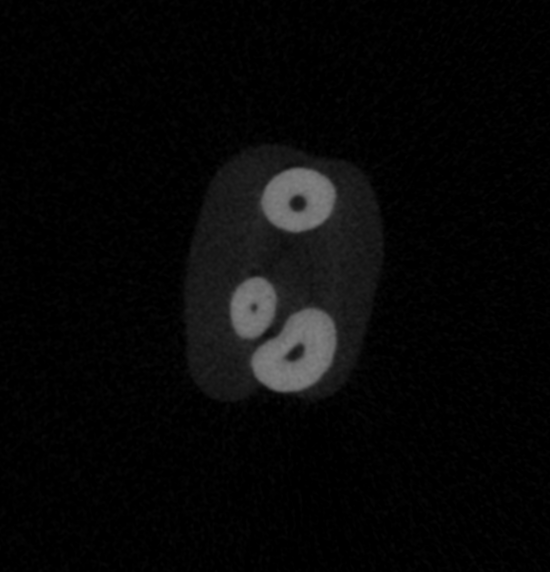

Supplement: S1 File — (ZIP) [file pone.0209698.s001.zip › Skyscan 2211 micro-CT/skyscan2211_0072.tif]

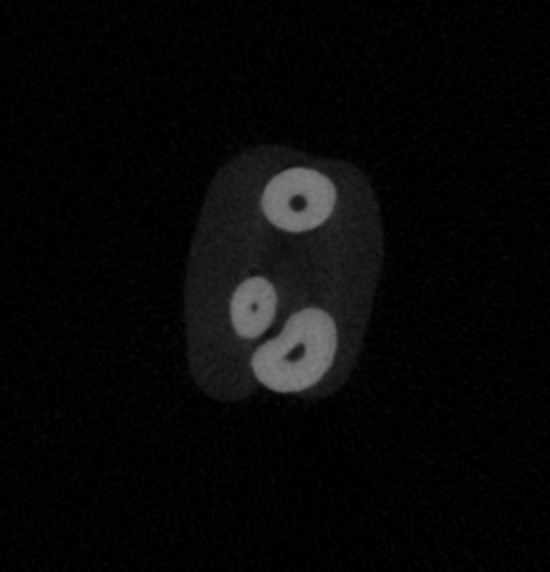

Supplement: S1 File — (ZIP) [file pone.0209698.s001.zip › Skyscan 2211 micro-CT/skyscan2211_0073.tif]

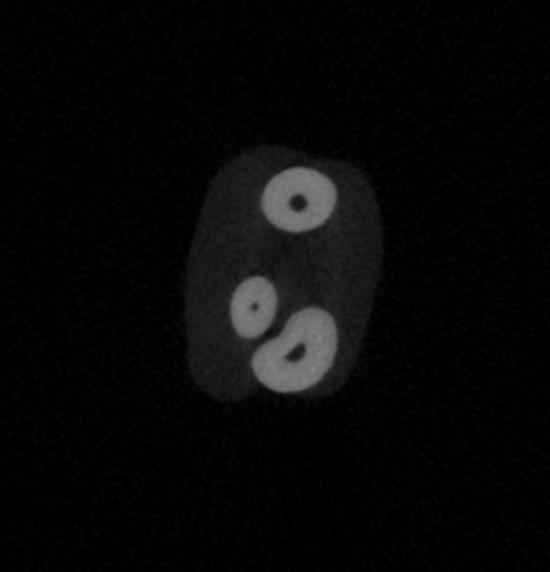

Supplement: S1 File — (ZIP) [file pone.0209698.s001.zip › Skyscan 2211 micro-CT/skyscan2211_0074.tif]

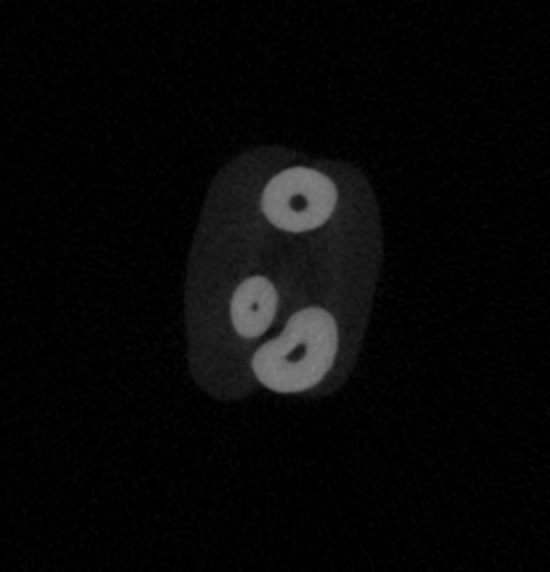

Supplement: S1 File — (ZIP) [file pone.0209698.s001.zip › Skyscan 2211 micro-CT/skyscan2211_0075.tif]

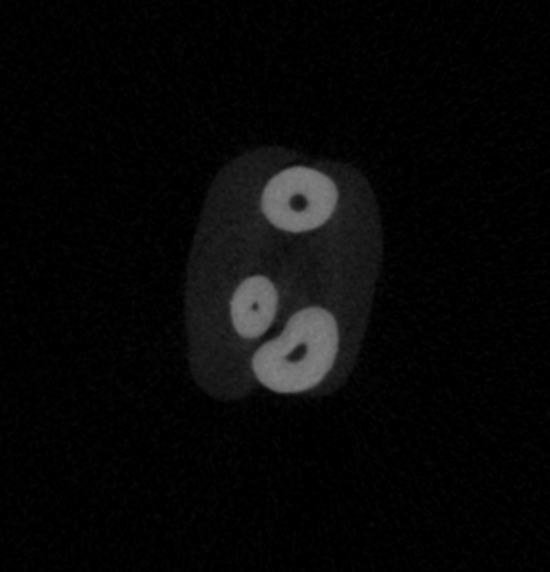

Supplement: S1 File — (ZIP) [file pone.0209698.s001.zip › Skyscan 2211 micro-CT/skyscan2211_0076.tif]

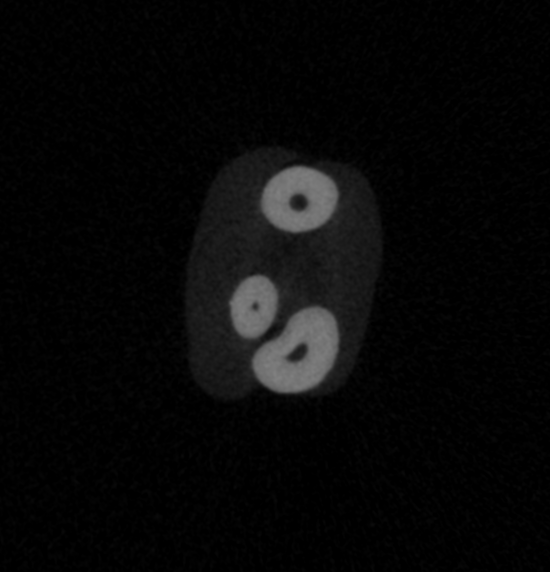

Supplement: S1 File — (ZIP) [file pone.0209698.s001.zip › Skyscan 2211 micro-CT/skyscan2211_0077.tif]

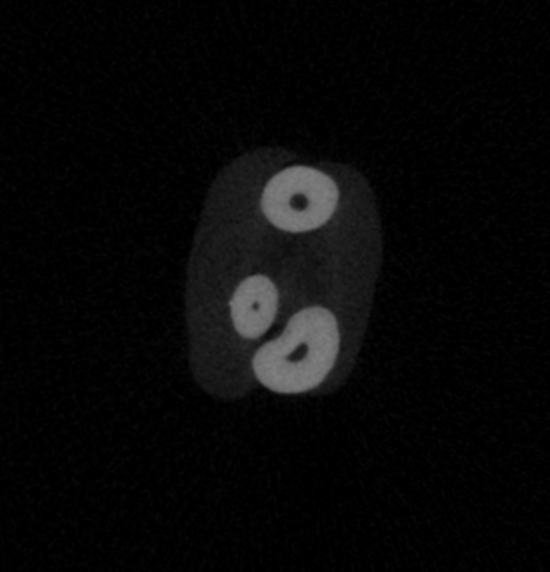

Supplement: S1 File — (ZIP) [file pone.0209698.s001.zip › Skyscan 2211 micro-CT/skyscan2211_0078.tif]

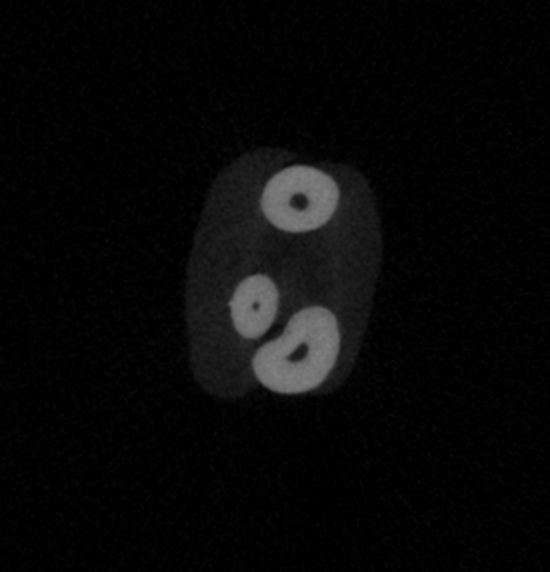

Supplement: S1 File — (ZIP) [file pone.0209698.s001.zip › Skyscan 2211 micro-CT/skyscan2211_0079.tif]

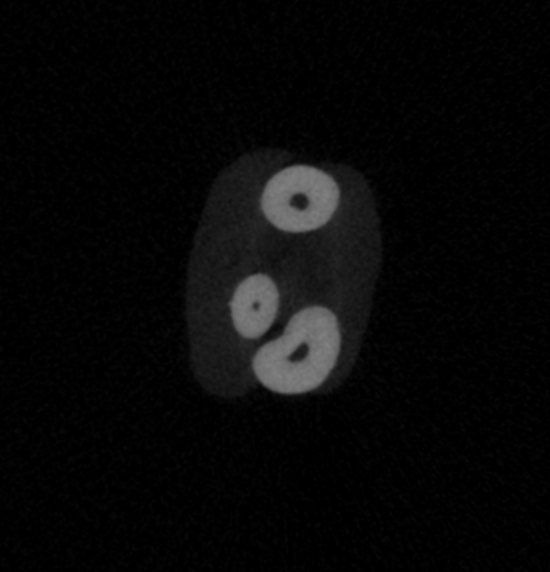

Supplement: S1 File — (ZIP) [file pone.0209698.s001.zip › Skyscan 2211 micro-CT/skyscan2211_0080.tif]

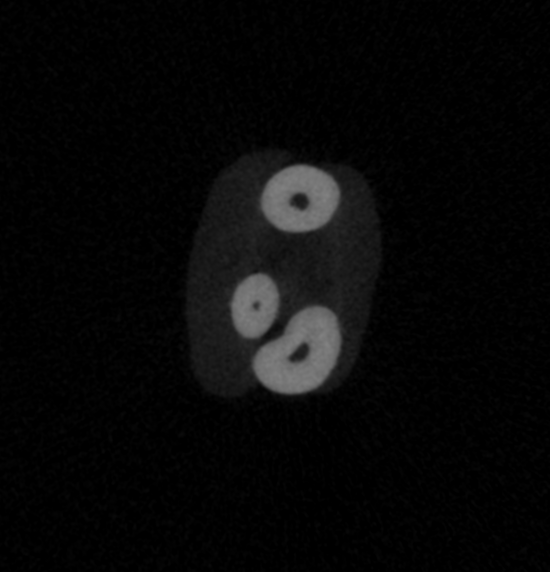

Supplement: S1 File — (ZIP) [file pone.0209698.s001.zip › Skyscan 2211 micro-CT/skyscan2211_0081.tif]

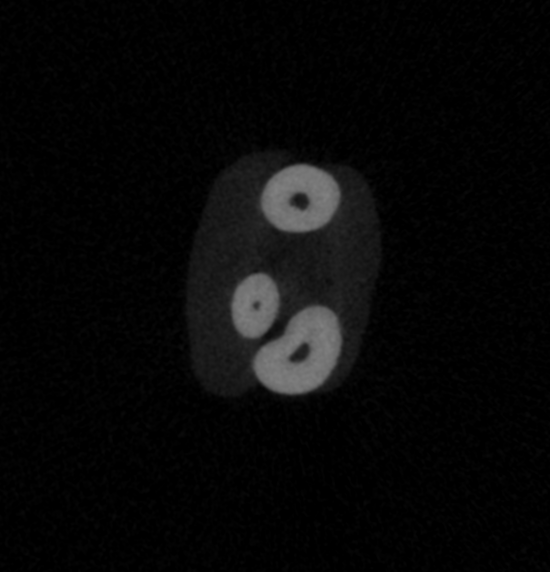

Supplement: S1 File — (ZIP) [file pone.0209698.s001.zip › Skyscan 2211 micro-CT/skyscan2211_0082.tif]

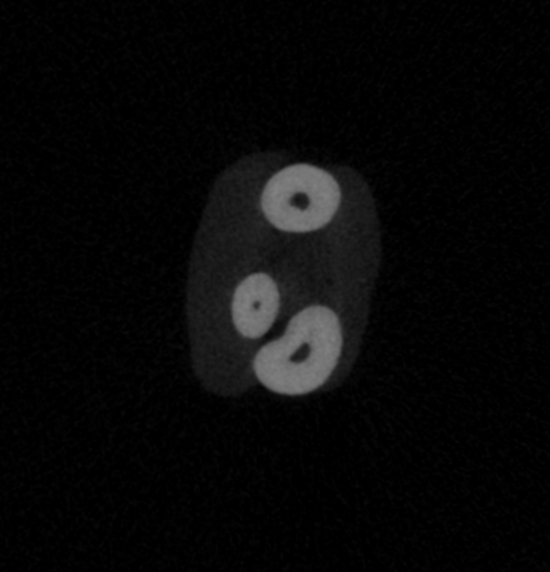

Supplement: S1 File — (ZIP) [file pone.0209698.s001.zip › Skyscan 2211 micro-CT/skyscan2211_0083.tif]

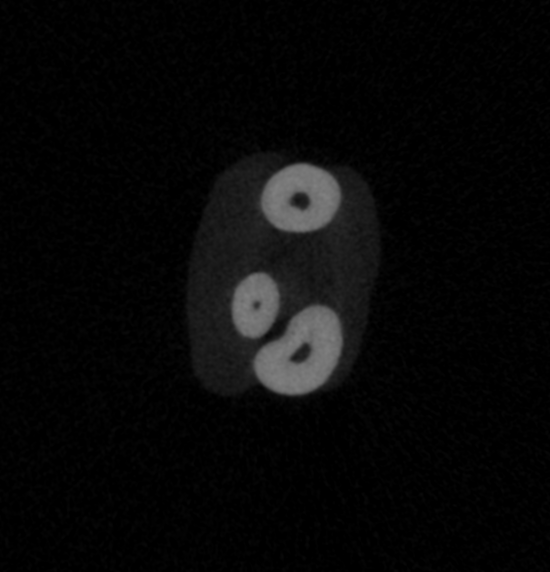

Supplement: S1 File — (ZIP) [file pone.0209698.s001.zip › Skyscan 2211 micro-CT/skyscan2211_0084.tif]

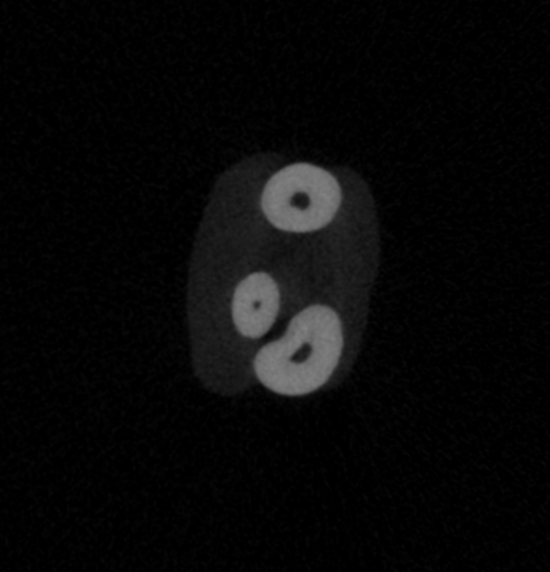

Supplement: S1 File — (ZIP) [file pone.0209698.s001.zip › Skyscan 2211 micro-CT/skyscan2211_0085.tif]

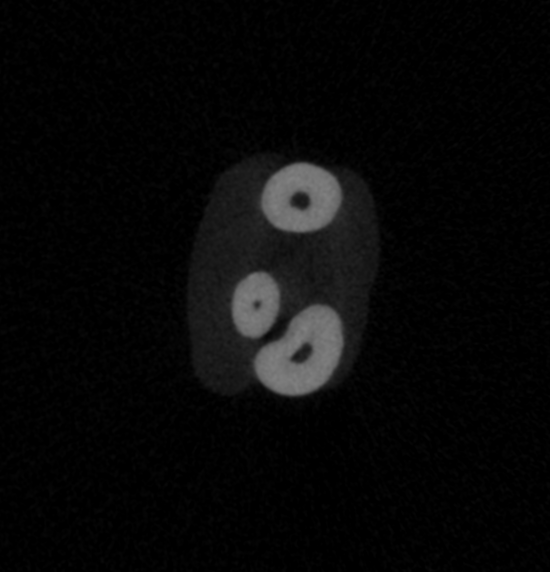

Supplement: S1 File — (ZIP) [file pone.0209698.s001.zip › Skyscan 2211 micro-CT/skyscan2211_0086.tif]

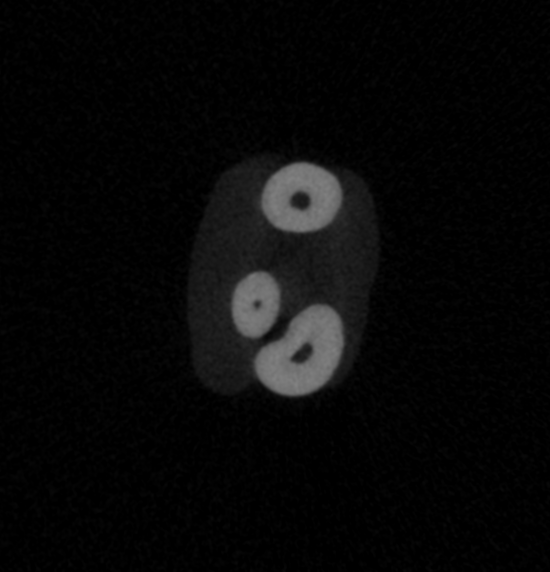

Supplement: S1 File — (ZIP) [file pone.0209698.s001.zip › Skyscan 2211 micro-CT/skyscan2211_0087.tif]

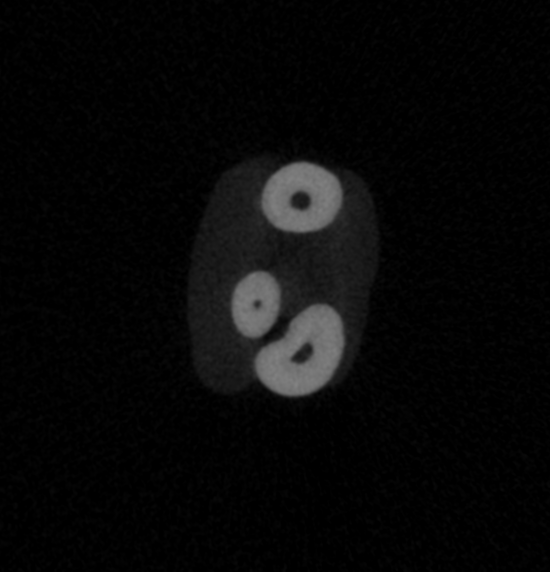

Supplement: S1 File — (ZIP) [file pone.0209698.s001.zip › Skyscan 2211 micro-CT/skyscan2211_0088.tif]

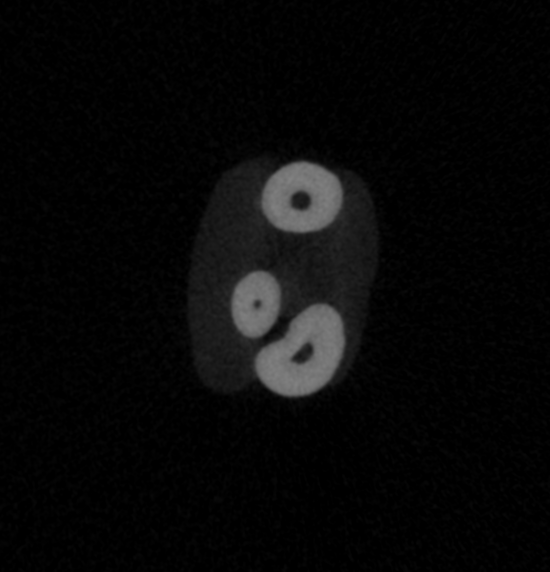

Supplement: S1 File — (ZIP) [file pone.0209698.s001.zip › Skyscan 2211 micro-CT/skyscan2211_0089.tif]

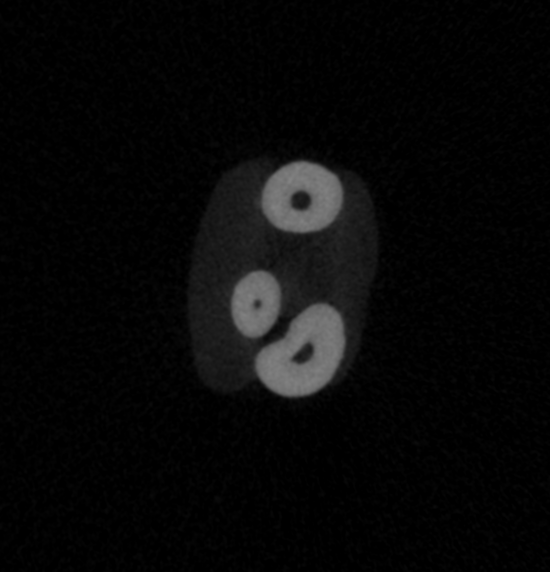

Supplement: S1 File — (ZIP) [file pone.0209698.s001.zip › Skyscan 2211 micro-CT/skyscan2211_0090.tif]

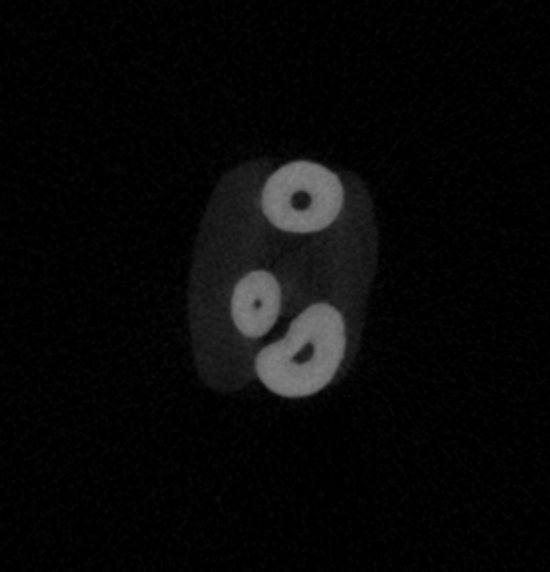

Supplement: S1 File — (ZIP) [file pone.0209698.s001.zip › Skyscan 2211 micro-CT/skyscan2211_0091.tif]

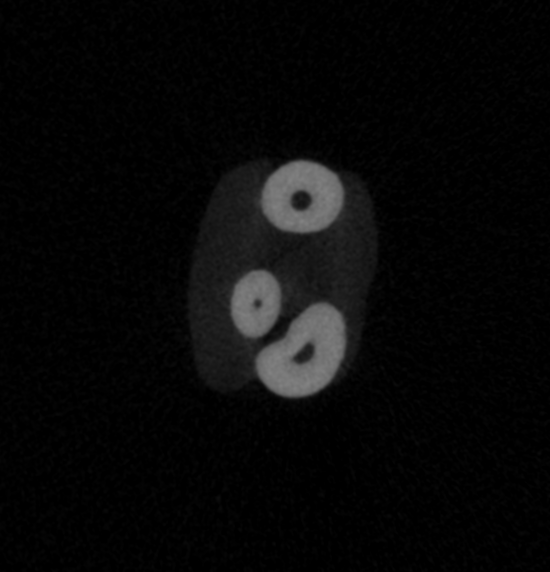

Supplement: S1 File — (ZIP) [file pone.0209698.s001.zip › Skyscan 2211 micro-CT/skyscan2211_0092.tif]

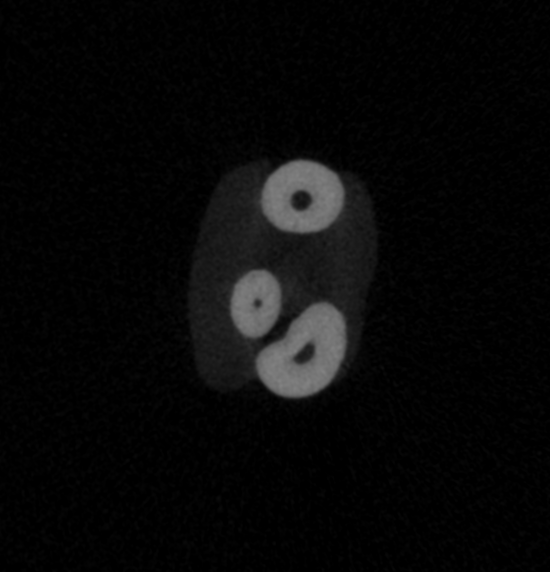

Supplement: S1 File — (ZIP) [file pone.0209698.s001.zip › Skyscan 2211 micro-CT/skyscan2211_0093.tif]

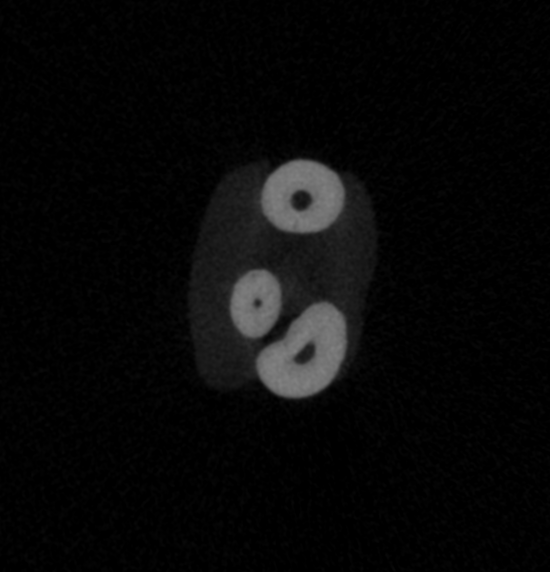

Supplement: S1 File — (ZIP) [file pone.0209698.s001.zip › Skyscan 2211 micro-CT/skyscan2211_0094.tif]

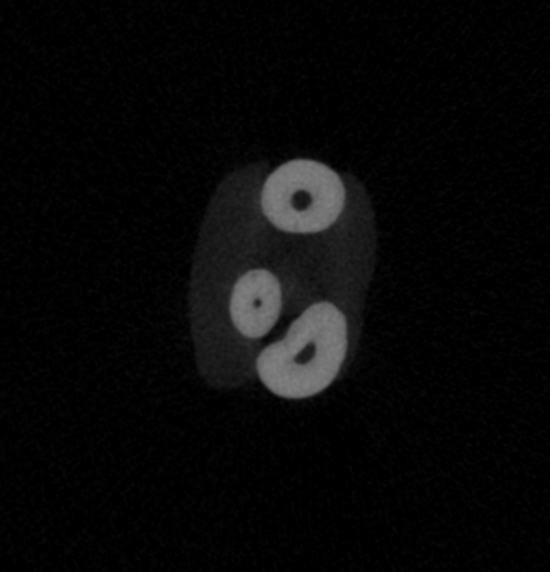

Supplement: S1 File — (ZIP) [file pone.0209698.s001.zip › Skyscan 2211 micro-CT/skyscan2211_0095.tif]

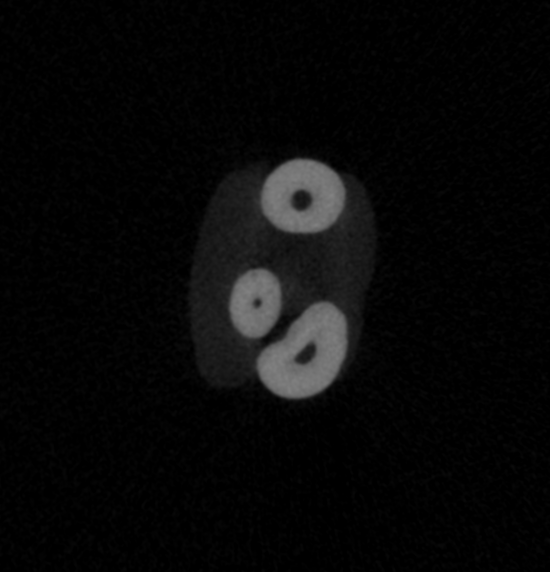

Supplement: S1 File — (ZIP) [file pone.0209698.s001.zip › Skyscan 2211 micro-CT/skyscan2211_0096.tif]

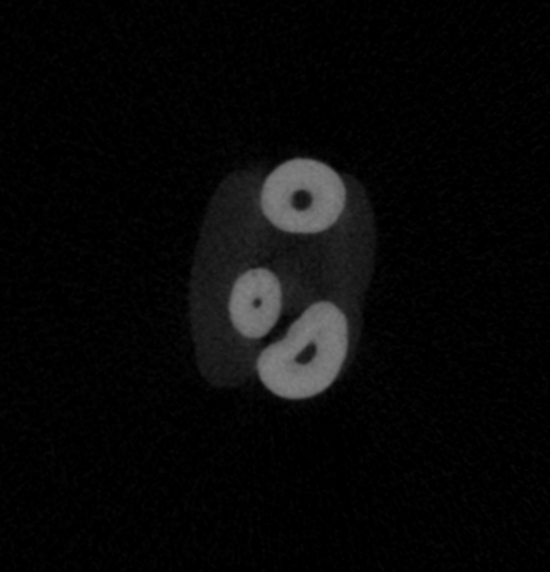

Supplement: S1 File — (ZIP) [file pone.0209698.s001.zip › Skyscan 2211 micro-CT/skyscan2211_0097.tif]

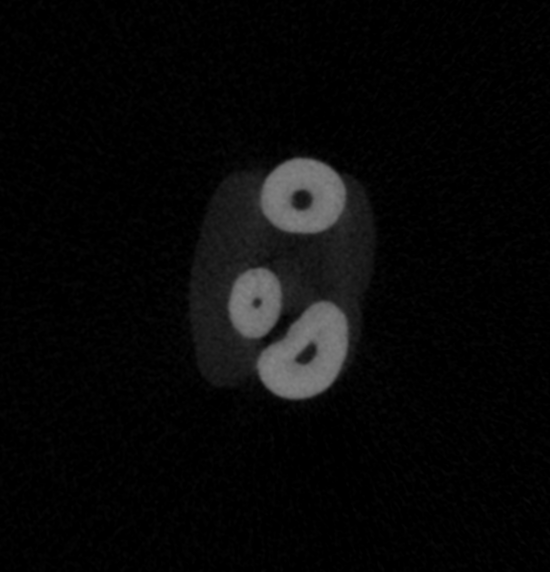

Supplement: S1 File — (ZIP) [file pone.0209698.s001.zip › Skyscan 2211 micro-CT/skyscan2211_0098.tif]

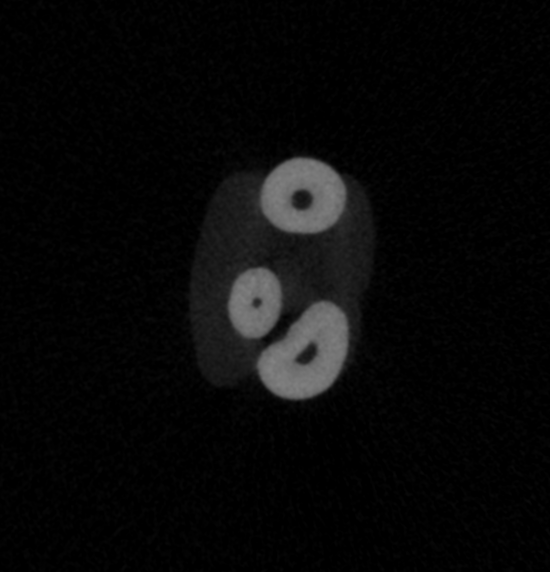

Supplement: S1 File — (ZIP) [file pone.0209698.s001.zip › Skyscan 2211 micro-CT/skyscan2211_0099.tif]

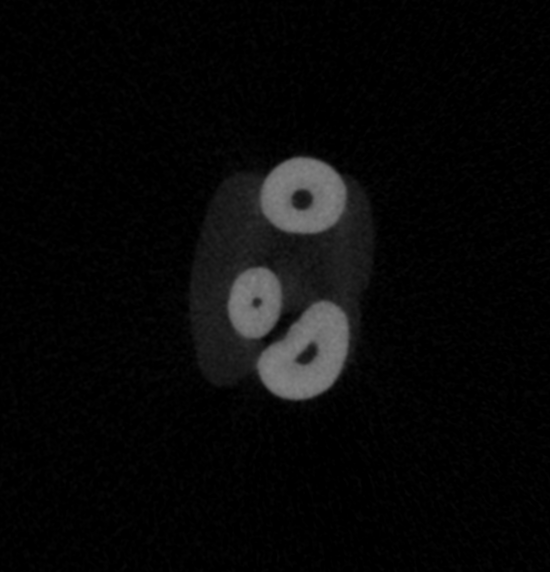

Supplement: S1 File — (ZIP) [file pone.0209698.s001.zip › Skyscan 2211 micro-CT/skyscan2211_0100.tif]

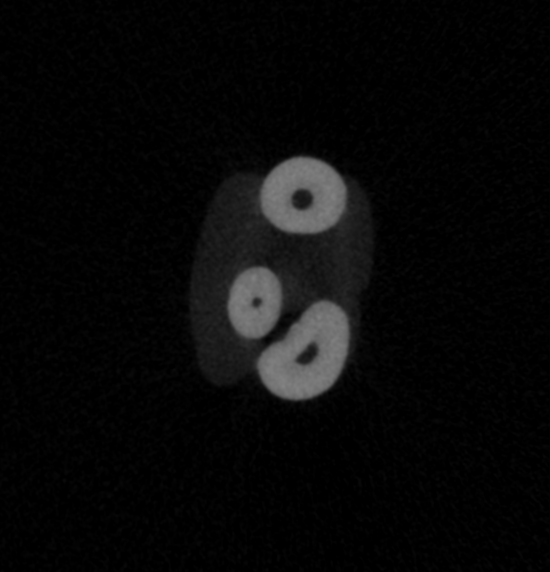

Supplement: S1 File — (ZIP) [file pone.0209698.s001.zip › Skyscan 2211 micro-CT/skyscan2211_0101.tif]
