# Supplementary figures and images for: Self-assembled micro-computed tomography for dental education (part 4 of 4)
Source: PLoS One. 2018 Dec 26;13(12):e0209698. doi: 10.1371/journal.pone.0209698 (PMC6306236; doi:10.1371/journal.pone.0209698)

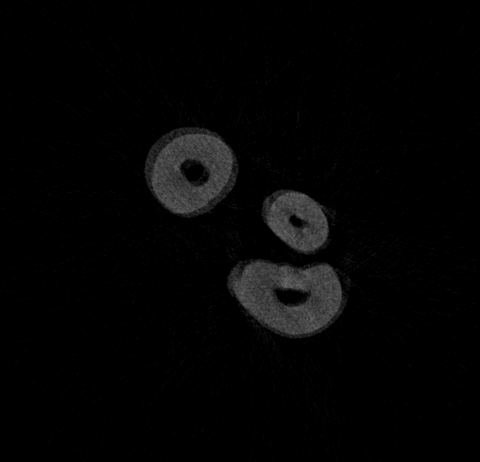

Supplement: S2 File — (ZIP) [file pone.0209698.s002.zip › Self-Assembled micro-CT/Self-Assembled micro CT_0152.tif]

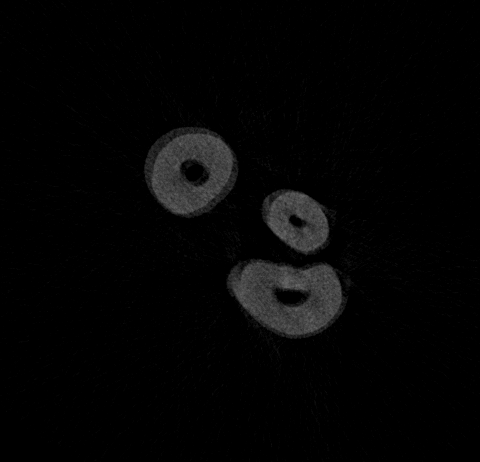

Supplement: S2 File — (ZIP) [file pone.0209698.s002.zip › Self-Assembled micro-CT/Self-Assembled micro CT_0153.tif]

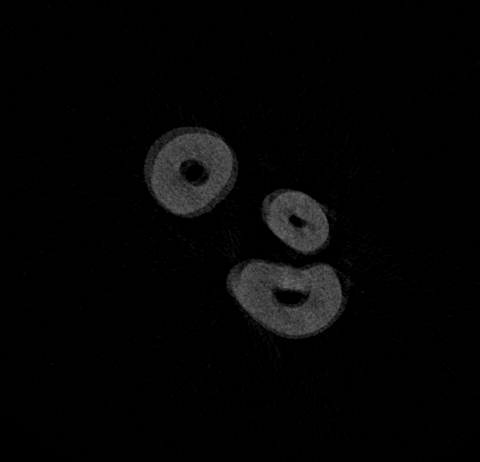

Supplement: S2 File — (ZIP) [file pone.0209698.s002.zip › Self-Assembled micro-CT/Self-Assembled micro CT_0154.tif]

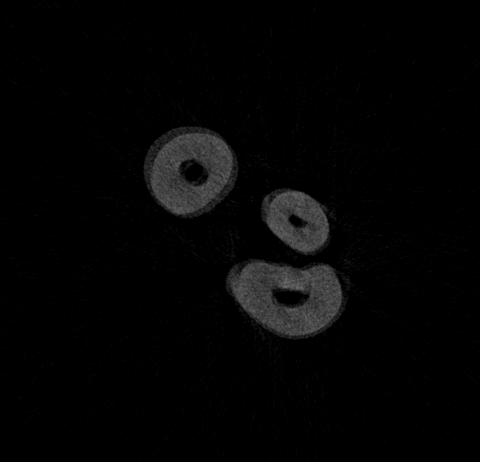

Supplement: S2 File — (ZIP) [file pone.0209698.s002.zip › Self-Assembled micro-CT/Self-Assembled micro CT_0155.tif]

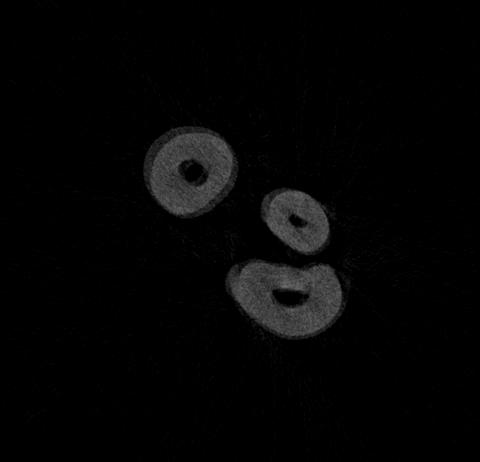

Supplement: S2 File — (ZIP) [file pone.0209698.s002.zip › Self-Assembled micro-CT/Self-Assembled micro CT_0156.tif]

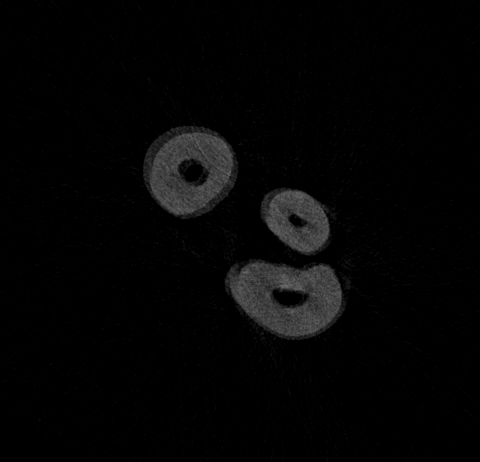

Supplement: S2 File — (ZIP) [file pone.0209698.s002.zip › Self-Assembled micro-CT/Self-Assembled micro CT_0157.tif]

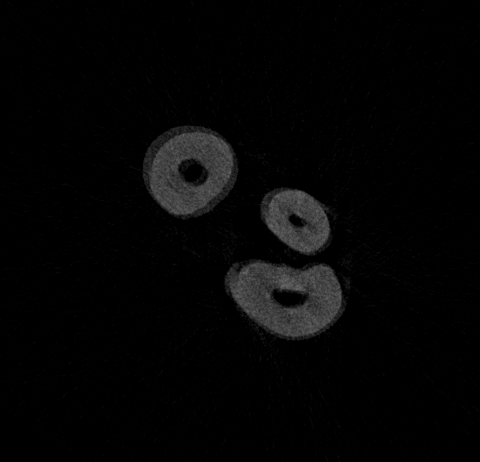

Supplement: S2 File — (ZIP) [file pone.0209698.s002.zip › Self-Assembled micro-CT/Self-Assembled micro CT_0158.tif]

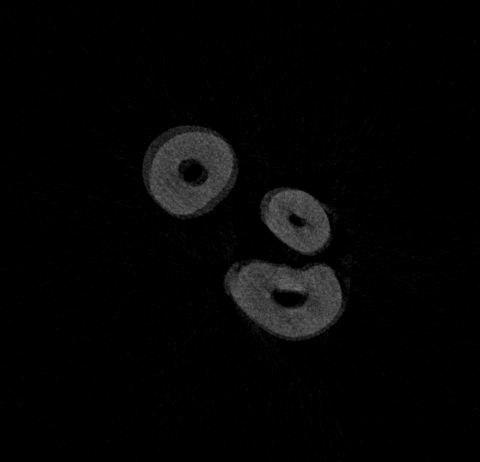

Supplement: S2 File — (ZIP) [file pone.0209698.s002.zip › Self-Assembled micro-CT/Self-Assembled micro CT_0159.tif]

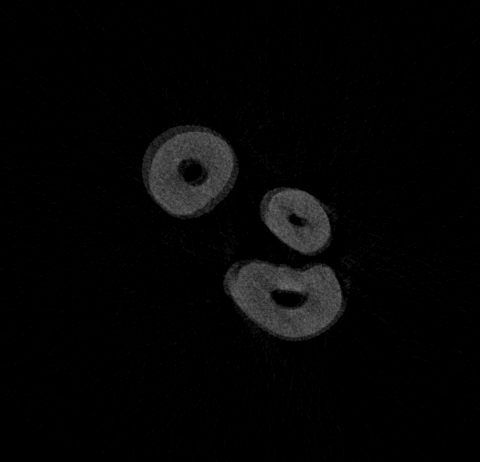

Supplement: S2 File — (ZIP) [file pone.0209698.s002.zip › Self-Assembled micro-CT/Self-Assembled micro CT_0160.tif]

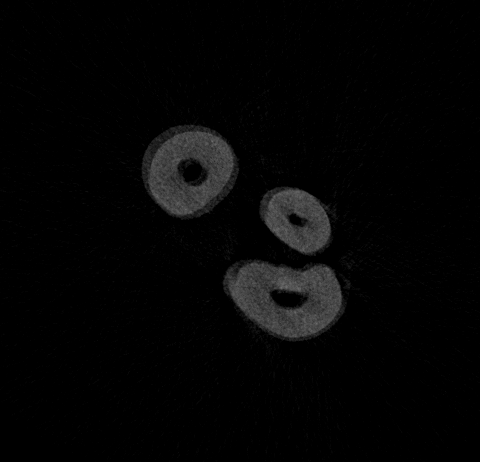

Supplement: S2 File — (ZIP) [file pone.0209698.s002.zip › Self-Assembled micro-CT/Self-Assembled micro CT_0161.tif]

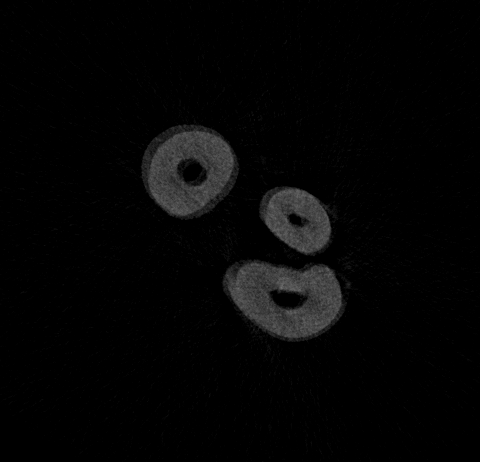

Supplement: S2 File — (ZIP) [file pone.0209698.s002.zip › Self-Assembled micro-CT/Self-Assembled micro CT_0162.tif]

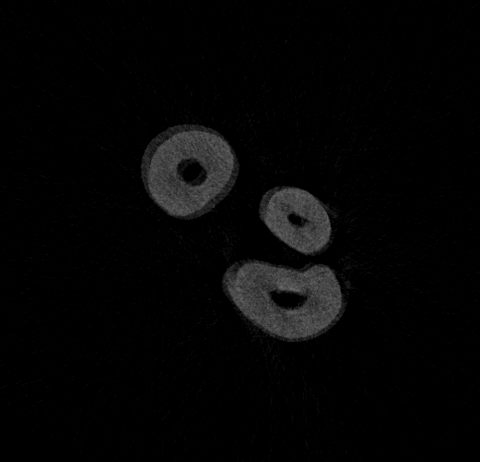

Supplement: S2 File — (ZIP) [file pone.0209698.s002.zip › Self-Assembled micro-CT/Self-Assembled micro CT_0163.tif]

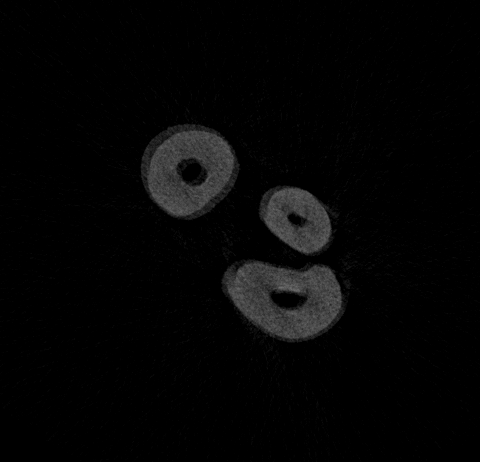

Supplement: S2 File — (ZIP) [file pone.0209698.s002.zip › Self-Assembled micro-CT/Self-Assembled micro CT_0164.tif]

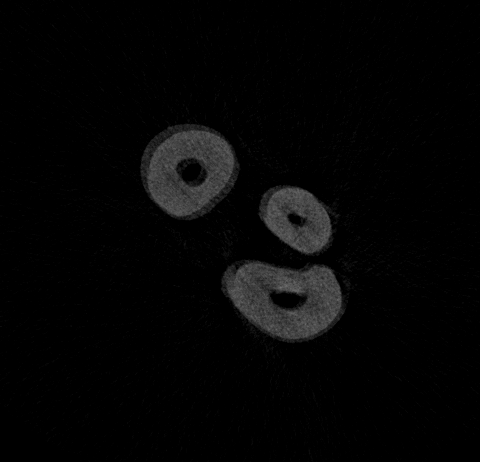

Supplement: S2 File — (ZIP) [file pone.0209698.s002.zip › Self-Assembled micro-CT/Self-Assembled micro CT_0165.tif]

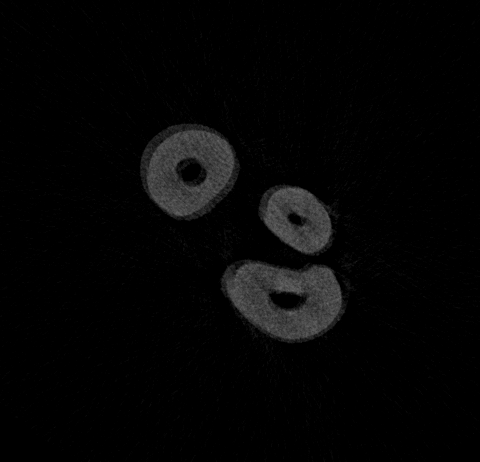

Supplement: S2 File — (ZIP) [file pone.0209698.s002.zip › Self-Assembled micro-CT/Self-Assembled micro CT_0166.tif]

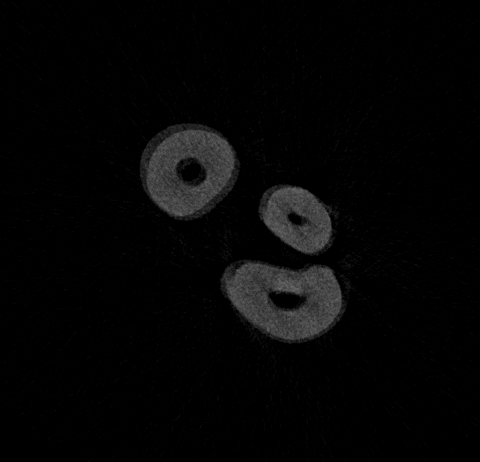

Supplement: S2 File — (ZIP) [file pone.0209698.s002.zip › Self-Assembled micro-CT/Self-Assembled micro CT_0167.tif]

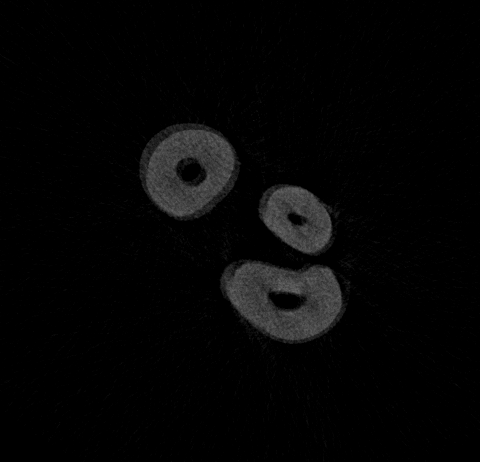

Supplement: S2 File — (ZIP) [file pone.0209698.s002.zip › Self-Assembled micro-CT/Self-Assembled micro CT_0168.tif]

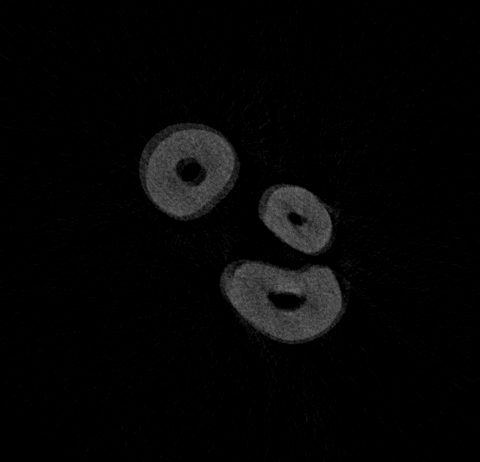

Supplement: S2 File — (ZIP) [file pone.0209698.s002.zip › Self-Assembled micro-CT/Self-Assembled micro CT_0169.tif]

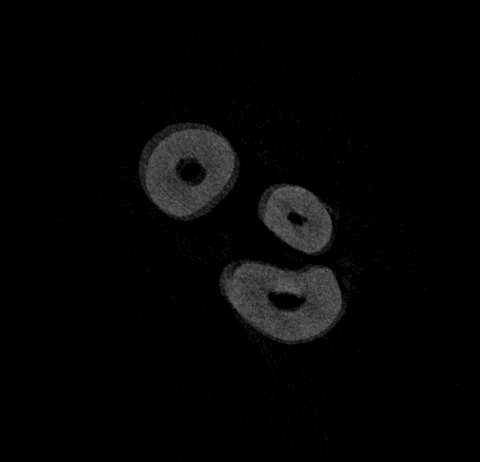

Supplement: S2 File — (ZIP) [file pone.0209698.s002.zip › Self-Assembled micro-CT/Self-Assembled micro CT_0170.tif]

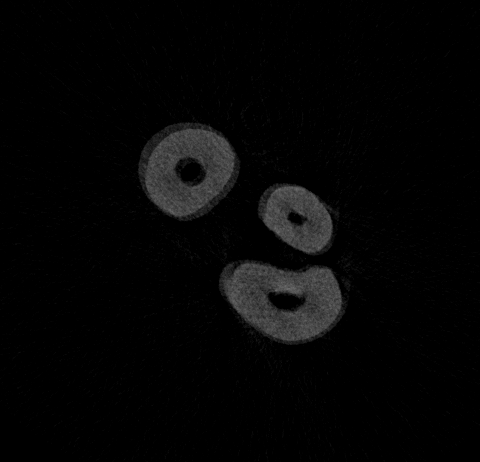

Supplement: S2 File — (ZIP) [file pone.0209698.s002.zip › Self-Assembled micro-CT/Self-Assembled micro CT_0171.tif]

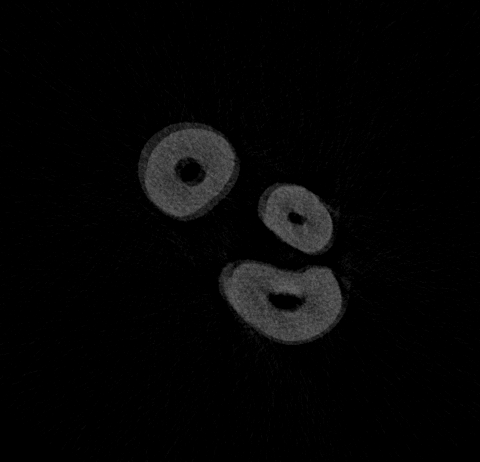

Supplement: S2 File — (ZIP) [file pone.0209698.s002.zip › Self-Assembled micro-CT/Self-Assembled micro CT_0172.tif]

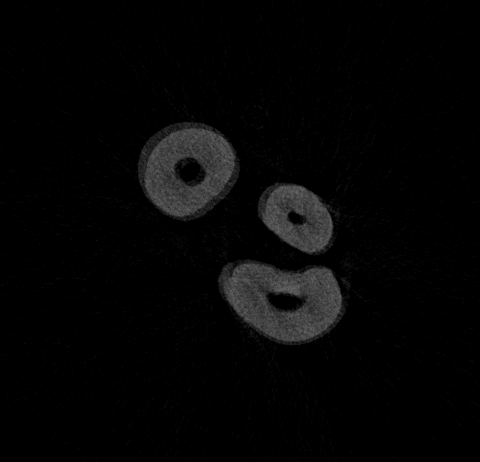

Supplement: S2 File — (ZIP) [file pone.0209698.s002.zip › Self-Assembled micro-CT/Self-Assembled micro CT_0173.tif]

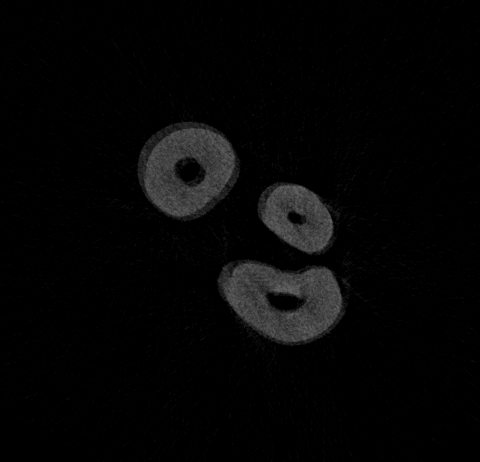

Supplement: S2 File — (ZIP) [file pone.0209698.s002.zip › Self-Assembled micro-CT/Self-Assembled micro CT_0174.tif]

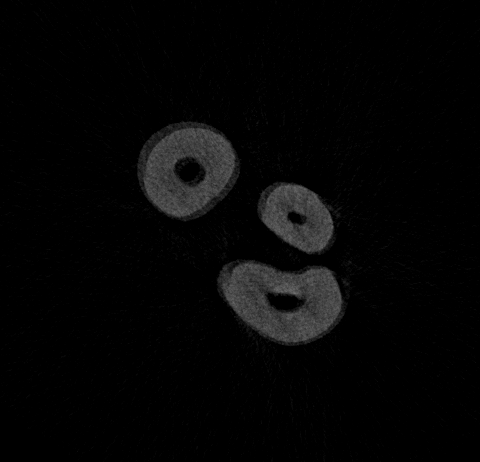

Supplement: S2 File — (ZIP) [file pone.0209698.s002.zip › Self-Assembled micro-CT/Self-Assembled micro CT_0175.tif]

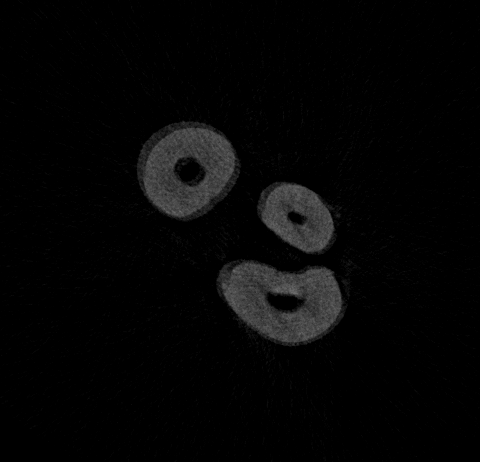

Supplement: S2 File — (ZIP) [file pone.0209698.s002.zip › Self-Assembled micro-CT/Self-Assembled micro CT_0176.tif]

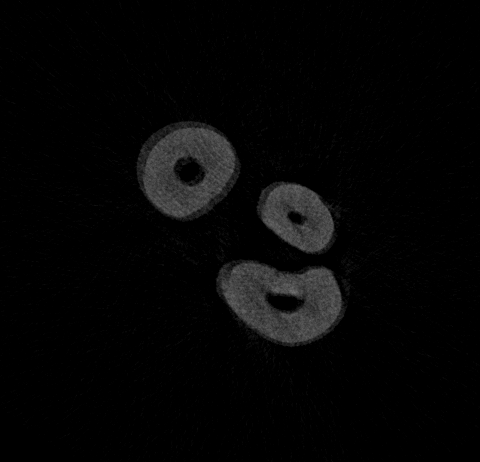

Supplement: S2 File — (ZIP) [file pone.0209698.s002.zip › Self-Assembled micro-CT/Self-Assembled micro CT_0177.tif]

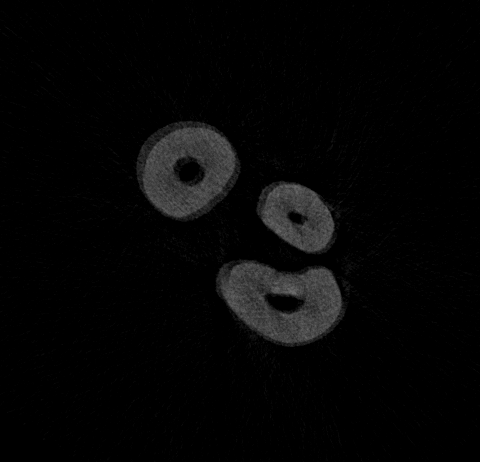

Supplement: S2 File — (ZIP) [file pone.0209698.s002.zip › Self-Assembled micro-CT/Self-Assembled micro CT_0178.tif]

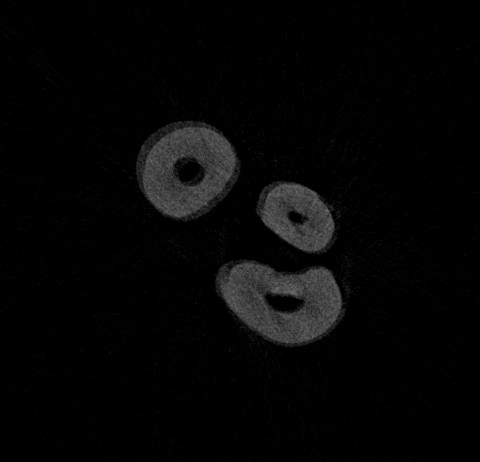

Supplement: S2 File — (ZIP) [file pone.0209698.s002.zip › Self-Assembled micro-CT/Self-Assembled micro CT_0179.tif]

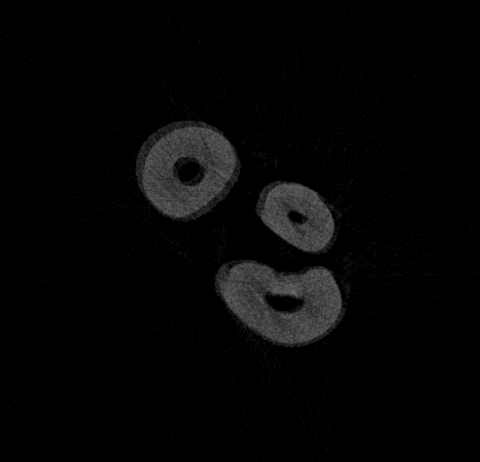

Supplement: S2 File — (ZIP) [file pone.0209698.s002.zip › Self-Assembled micro-CT/Self-Assembled micro CT_0180.tif]

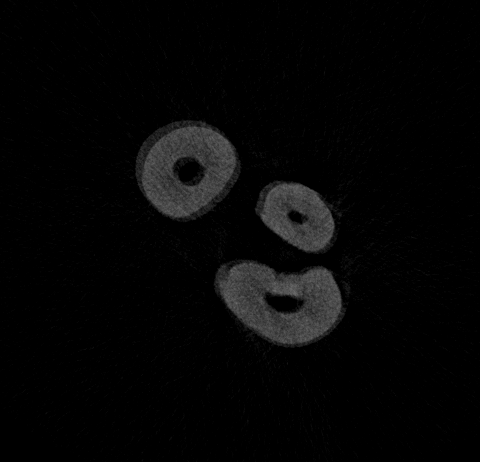

Supplement: S2 File — (ZIP) [file pone.0209698.s002.zip › Self-Assembled micro-CT/Self-Assembled micro CT_0181.tif]

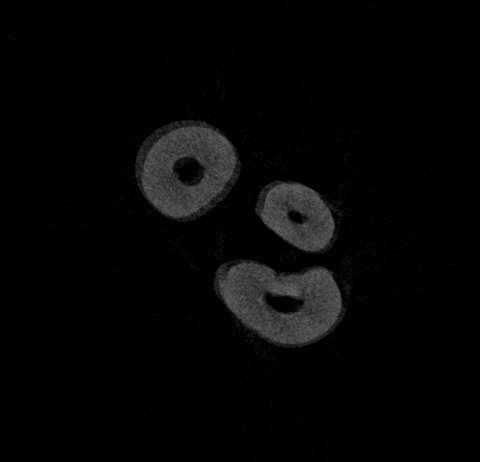

Supplement: S2 File — (ZIP) [file pone.0209698.s002.zip › Self-Assembled micro-CT/Self-Assembled micro CT_0182.tif]

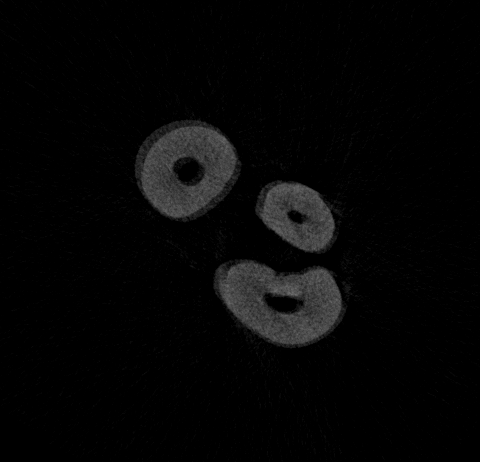

Supplement: S2 File — (ZIP) [file pone.0209698.s002.zip › Self-Assembled micro-CT/Self-Assembled micro CT_0183.tif]

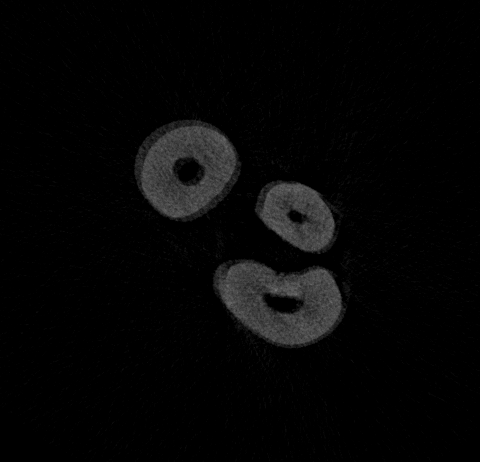

Supplement: S2 File — (ZIP) [file pone.0209698.s002.zip › Self-Assembled micro-CT/Self-Assembled micro CT_0184.tif]

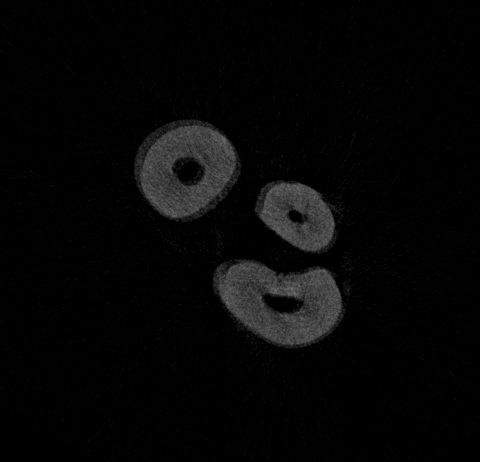

Supplement: S2 File — (ZIP) [file pone.0209698.s002.zip › Self-Assembled micro-CT/Self-Assembled micro CT_0185.tif]

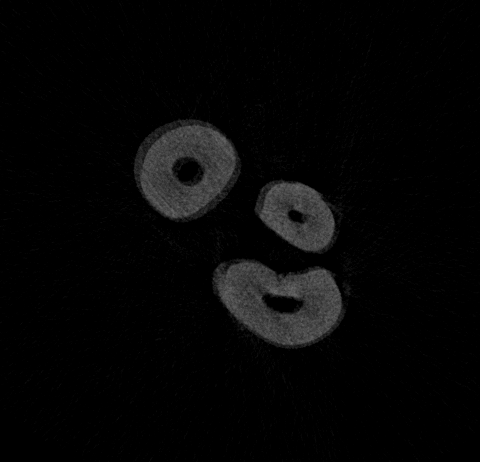

Supplement: S2 File — (ZIP) [file pone.0209698.s002.zip › Self-Assembled micro-CT/Self-Assembled micro CT_0186.tif]

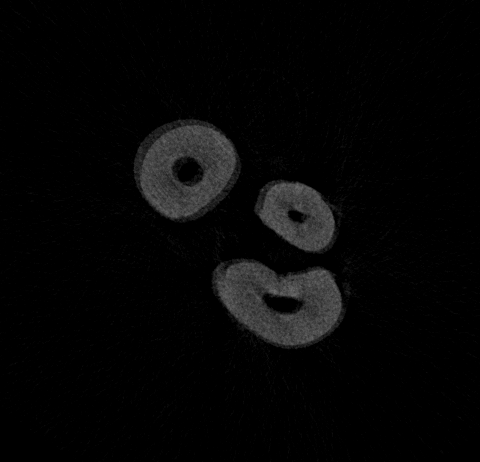

Supplement: S2 File — (ZIP) [file pone.0209698.s002.zip › Self-Assembled micro-CT/Self-Assembled micro CT_0187.tif]

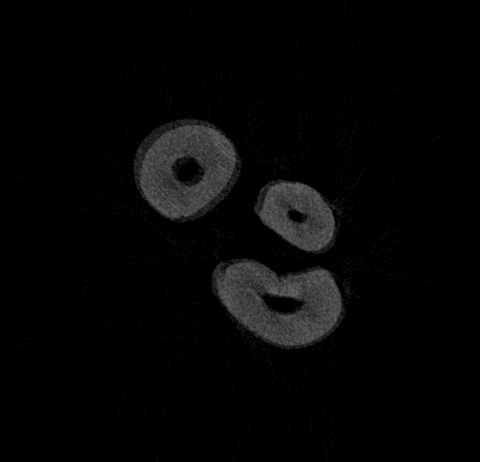

Supplement: S2 File — (ZIP) [file pone.0209698.s002.zip › Self-Assembled micro-CT/Self-Assembled micro CT_0188.tif]

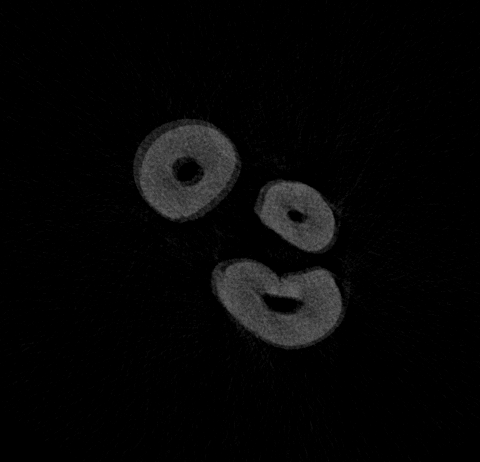

Supplement: S2 File — (ZIP) [file pone.0209698.s002.zip › Self-Assembled micro-CT/Self-Assembled micro CT_0189.tif]

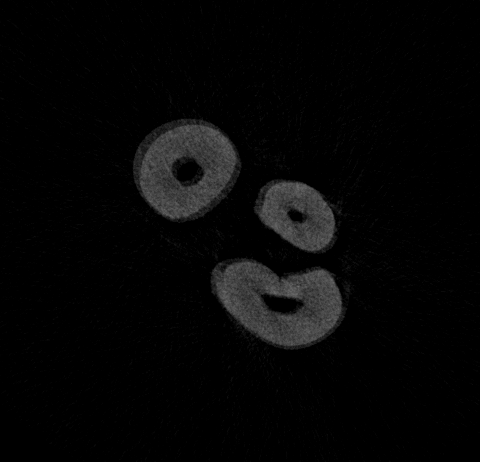

Supplement: S2 File — (ZIP) [file pone.0209698.s002.zip › Self-Assembled micro-CT/Self-Assembled micro CT_0190.tif]

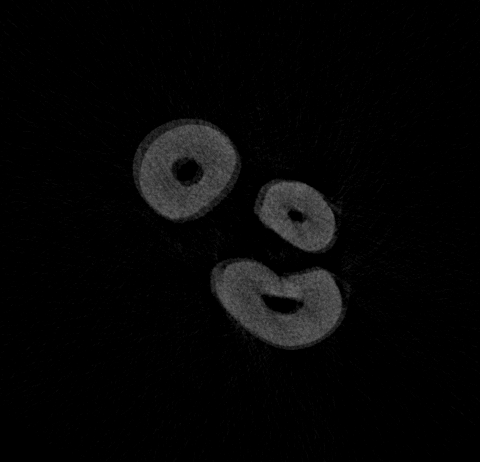

Supplement: S2 File — (ZIP) [file pone.0209698.s002.zip › Self-Assembled micro-CT/Self-Assembled micro CT_0191.tif]

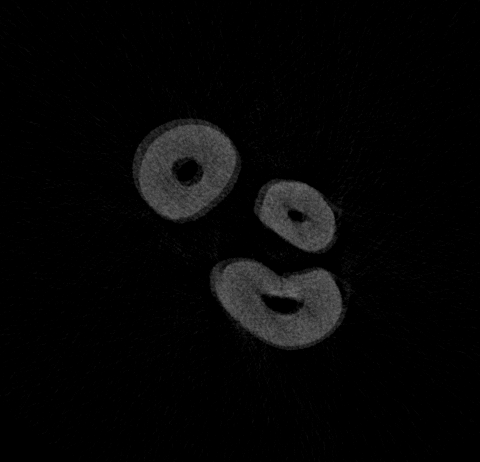

Supplement: S2 File — (ZIP) [file pone.0209698.s002.zip › Self-Assembled micro-CT/Self-Assembled micro CT_0192.tif]

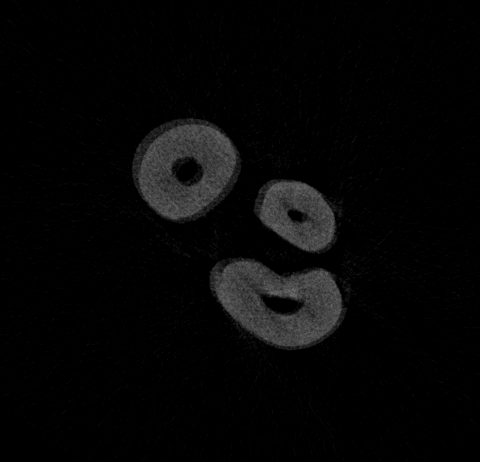

Supplement: S2 File — (ZIP) [file pone.0209698.s002.zip › Self-Assembled micro-CT/Self-Assembled micro CT_0193.tif]

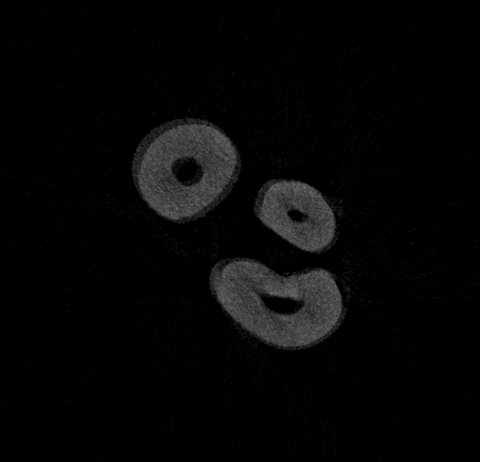

Supplement: S2 File — (ZIP) [file pone.0209698.s002.zip › Self-Assembled micro-CT/Self-Assembled micro CT_0194.tif]

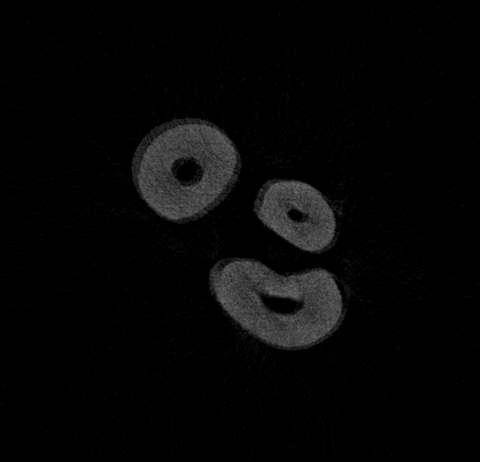

Supplement: S2 File — (ZIP) [file pone.0209698.s002.zip › Self-Assembled micro-CT/Self-Assembled micro CT_0195.tif]

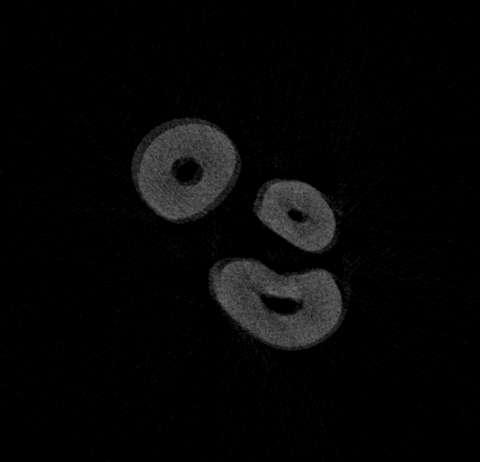

Supplement: S2 File — (ZIP) [file pone.0209698.s002.zip › Self-Assembled micro-CT/Self-Assembled micro CT_0196.tif]

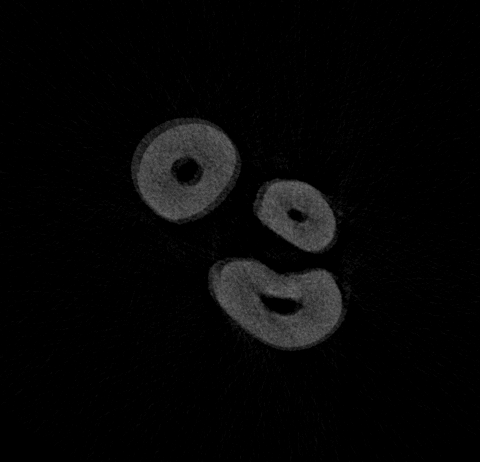

Supplement: S2 File — (ZIP) [file pone.0209698.s002.zip › Self-Assembled micro-CT/Self-Assembled micro CT_0197.tif]

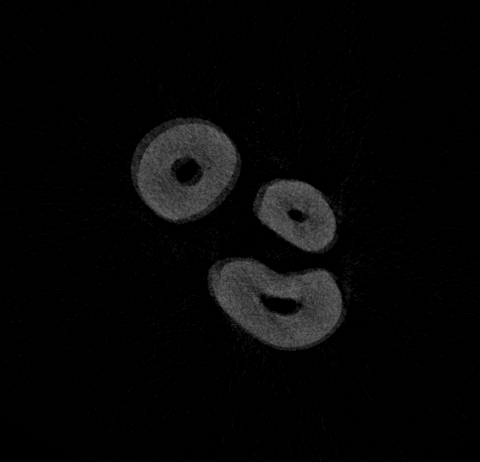

Supplement: S2 File — (ZIP) [file pone.0209698.s002.zip › Self-Assembled micro-CT/Self-Assembled micro CT_0198.tif]

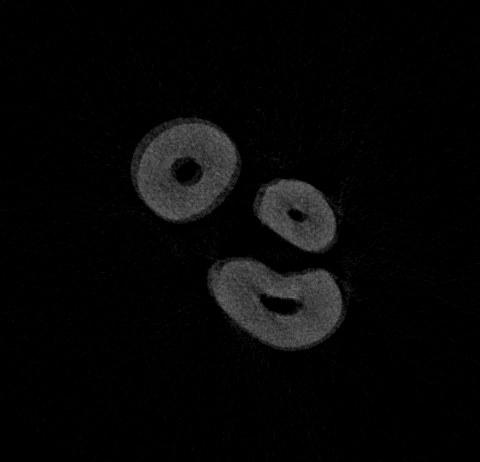

Supplement: S2 File — (ZIP) [file pone.0209698.s002.zip › Self-Assembled micro-CT/Self-Assembled micro CT_0199.tif]

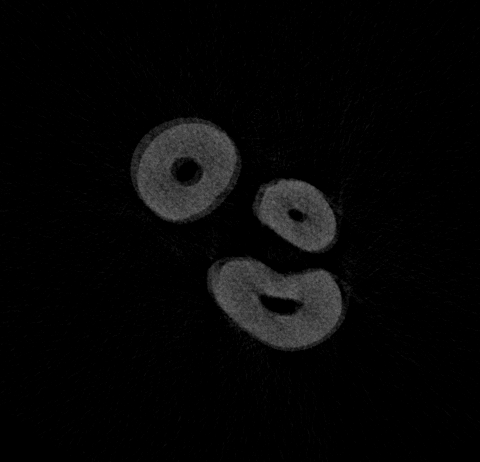

Supplement: S2 File — (ZIP) [file pone.0209698.s002.zip › Self-Assembled micro-CT/Self-Assembled micro CT_0200.tif]

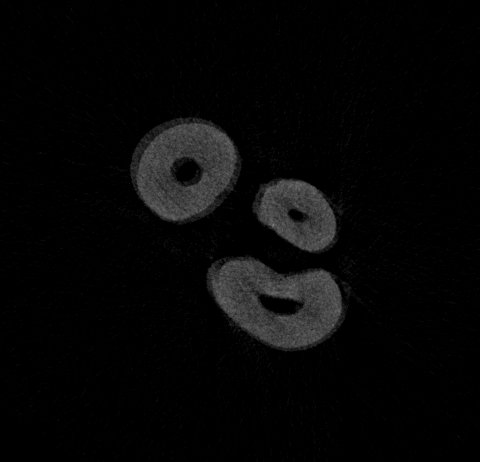

Supplement: S2 File — (ZIP) [file pone.0209698.s002.zip › Self-Assembled micro-CT/Self-Assembled micro CT_0201.tif]

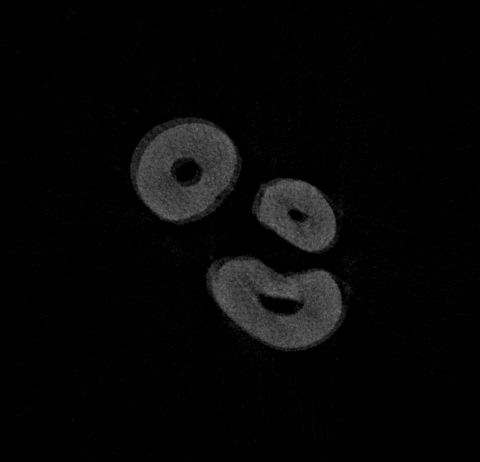

Supplement: S2 File — (ZIP) [file pone.0209698.s002.zip › Self-Assembled micro-CT/Self-Assembled micro CT_0202.tif]

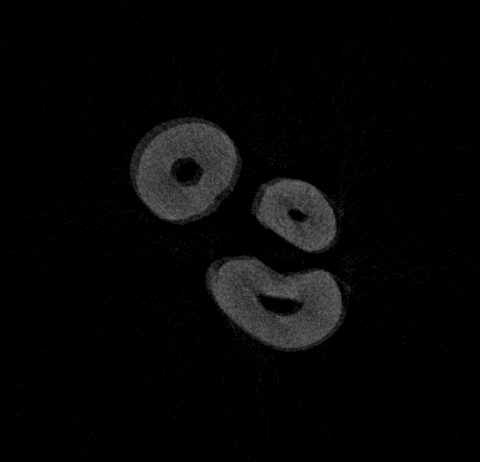

Supplement: S2 File — (ZIP) [file pone.0209698.s002.zip › Self-Assembled micro-CT/Self-Assembled micro CT_0203.tif]

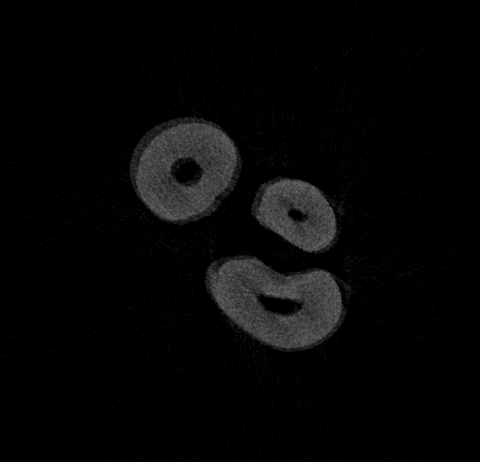

Supplement: S2 File — (ZIP) [file pone.0209698.s002.zip › Self-Assembled micro-CT/Self-Assembled micro CT_0204.tif]

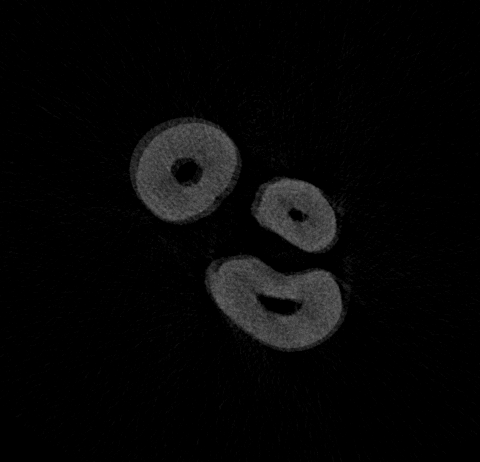

Supplement: S2 File — (ZIP) [file pone.0209698.s002.zip › Self-Assembled micro-CT/Self-Assembled micro CT_0205.tif]

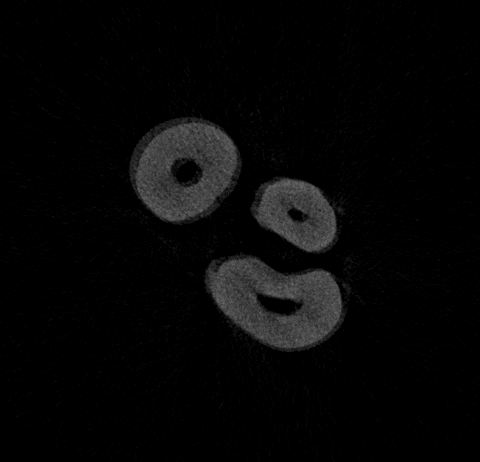

Supplement: S2 File — (ZIP) [file pone.0209698.s002.zip › Self-Assembled micro-CT/Self-Assembled micro CT_0206.tif]

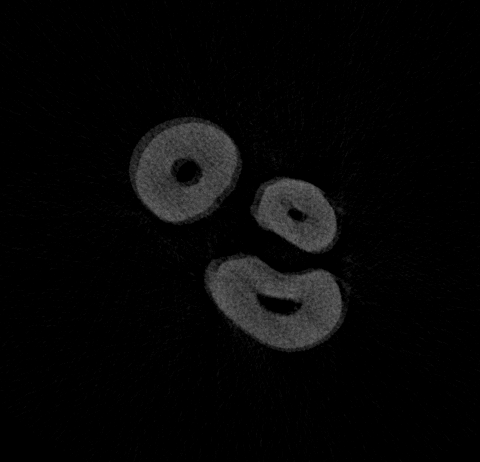

Supplement: S2 File — (ZIP) [file pone.0209698.s002.zip › Self-Assembled micro-CT/Self-Assembled micro CT_0207.tif]

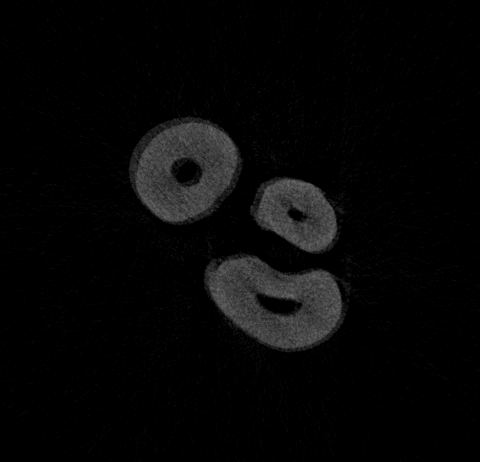

Supplement: S2 File — (ZIP) [file pone.0209698.s002.zip › Self-Assembled micro-CT/Self-Assembled micro CT_0208.tif]

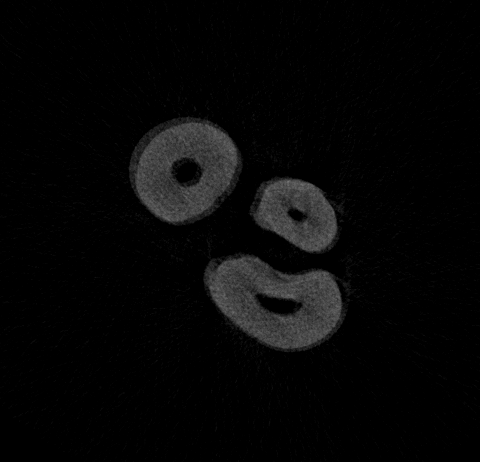

Supplement: S2 File — (ZIP) [file pone.0209698.s002.zip › Self-Assembled micro-CT/Self-Assembled micro CT_0209.tif]

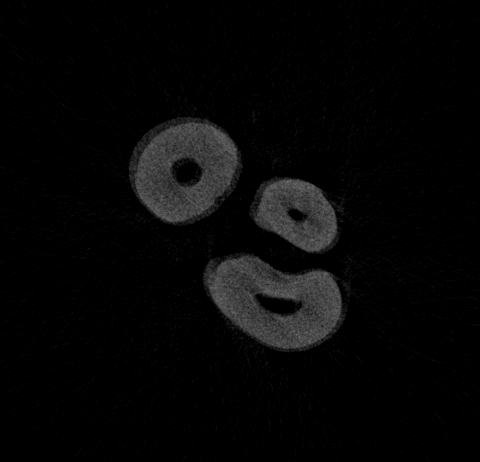

Supplement: S2 File — (ZIP) [file pone.0209698.s002.zip › Self-Assembled micro-CT/Self-Assembled micro CT_0210.tif]

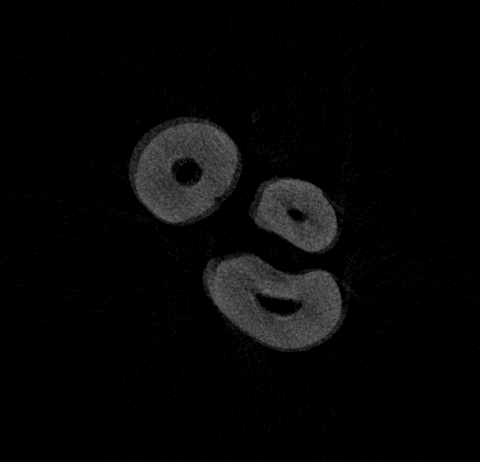

Supplement: S2 File — (ZIP) [file pone.0209698.s002.zip › Self-Assembled micro-CT/Self-Assembled micro CT_0211.tif]

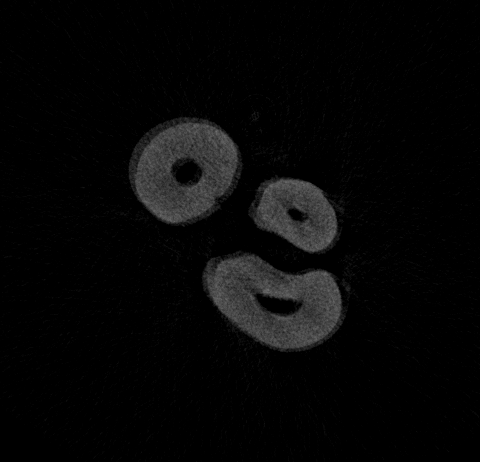

Supplement: S2 File — (ZIP) [file pone.0209698.s002.zip › Self-Assembled micro-CT/Self-Assembled micro CT_0212.tif]

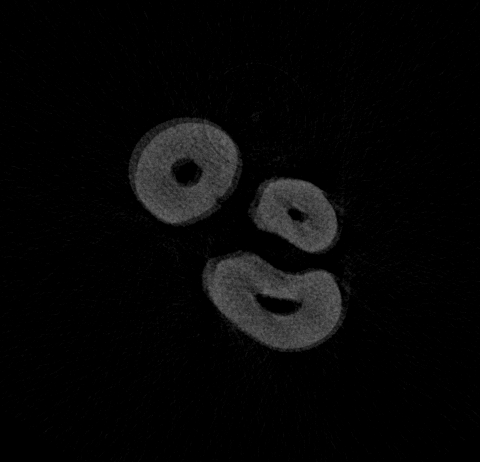

Supplement: S2 File — (ZIP) [file pone.0209698.s002.zip › Self-Assembled micro-CT/Self-Assembled micro CT_0213.tif]

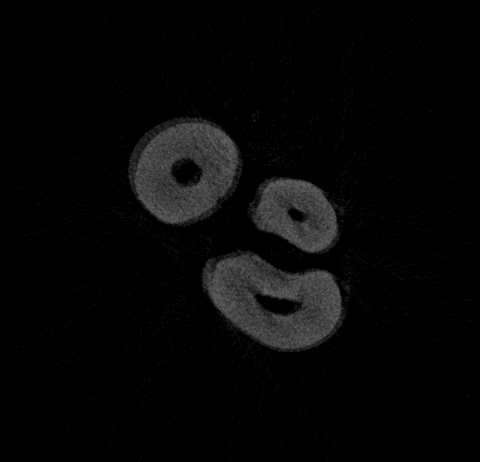

Supplement: S2 File — (ZIP) [file pone.0209698.s002.zip › Self-Assembled micro-CT/Self-Assembled micro CT_0214.tif]

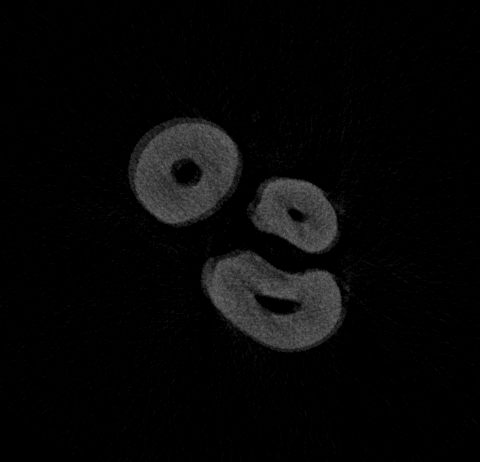

Supplement: S2 File — (ZIP) [file pone.0209698.s002.zip › Self-Assembled micro-CT/Self-Assembled micro CT_0215.tif]

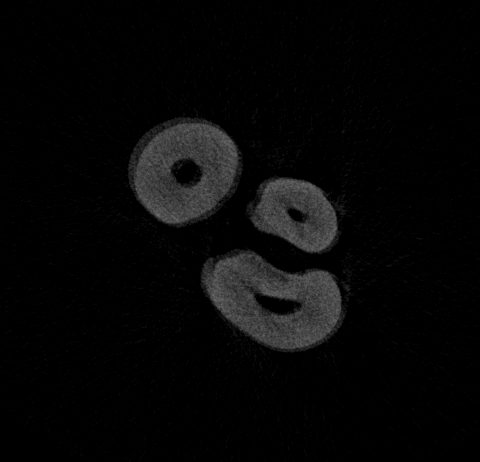

Supplement: S2 File — (ZIP) [file pone.0209698.s002.zip › Self-Assembled micro-CT/Self-Assembled micro CT_0216.tif]

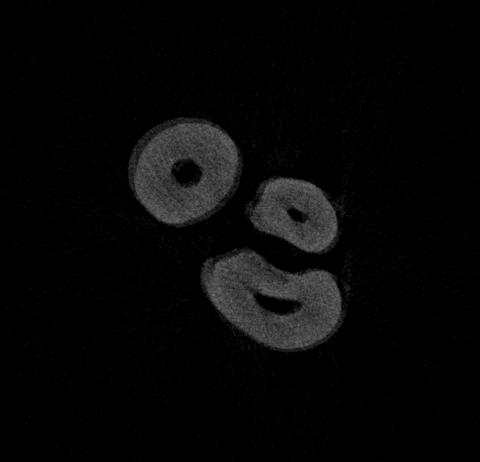

Supplement: S2 File — (ZIP) [file pone.0209698.s002.zip › Self-Assembled micro-CT/Self-Assembled micro CT_0217.tif]

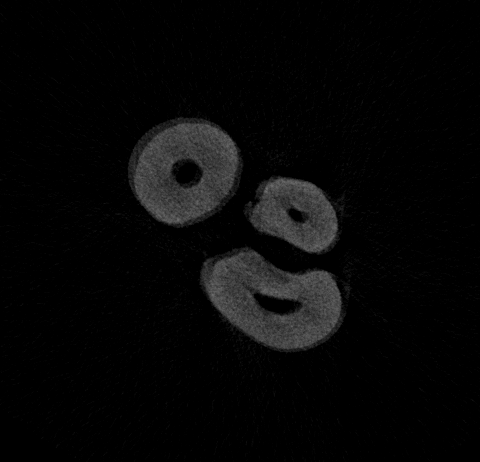

Supplement: S2 File — (ZIP) [file pone.0209698.s002.zip › Self-Assembled micro-CT/Self-Assembled micro CT_0218.tif]

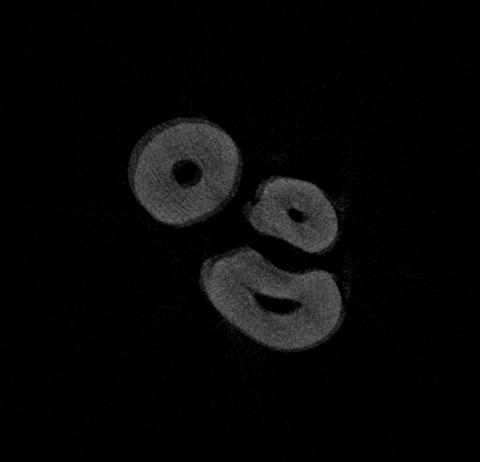

Supplement: S2 File — (ZIP) [file pone.0209698.s002.zip › Self-Assembled micro-CT/Self-Assembled micro CT_0219.tif]

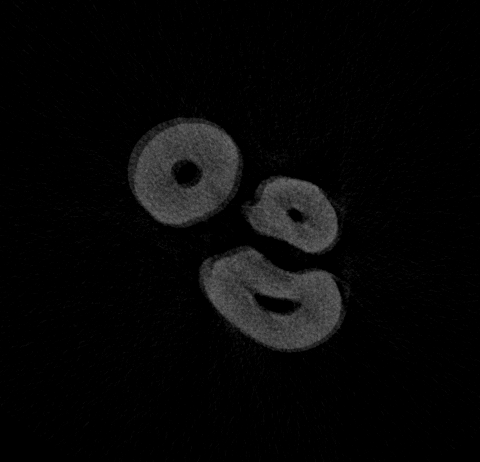

Supplement: S2 File — (ZIP) [file pone.0209698.s002.zip › Self-Assembled micro-CT/Self-Assembled micro CT_0220.tif]

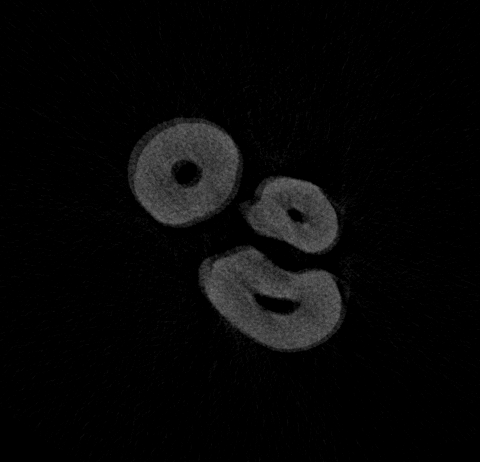

Supplement: S2 File — (ZIP) [file pone.0209698.s002.zip › Self-Assembled micro-CT/Self-Assembled micro CT_0221.tif]

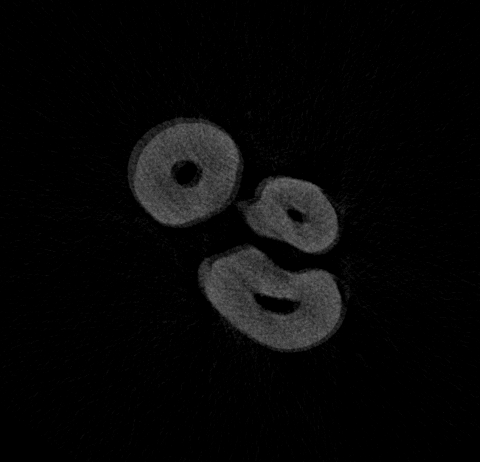

Supplement: S2 File — (ZIP) [file pone.0209698.s002.zip › Self-Assembled micro-CT/Self-Assembled micro CT_0222.tif]

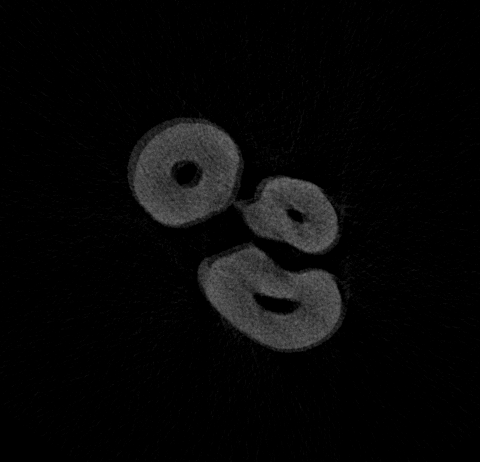

Supplement: S2 File — (ZIP) [file pone.0209698.s002.zip › Self-Assembled micro-CT/Self-Assembled micro CT_0223.tif]

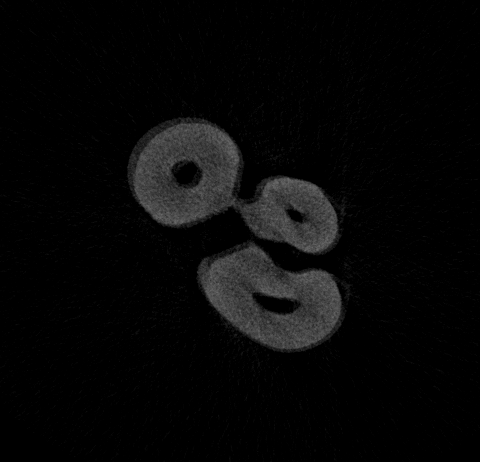

Supplement: S2 File — (ZIP) [file pone.0209698.s002.zip › Self-Assembled micro-CT/Self-Assembled micro CT_0224.tif]

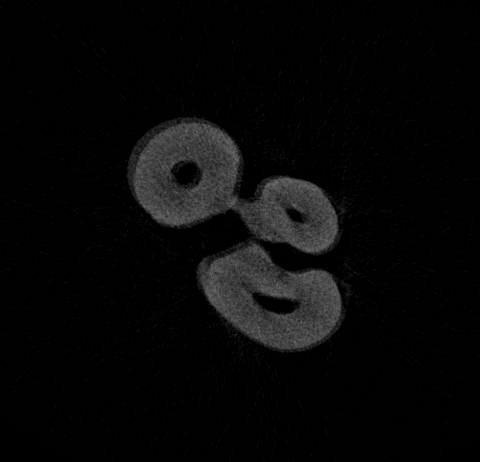

Supplement: S2 File — (ZIP) [file pone.0209698.s002.zip › Self-Assembled micro-CT/Self-Assembled micro CT_0225.tif]

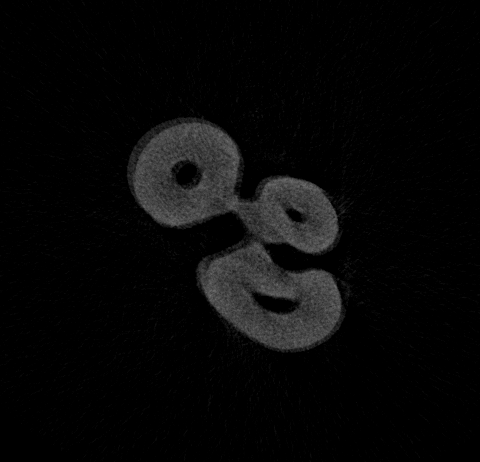

Supplement: S2 File — (ZIP) [file pone.0209698.s002.zip › Self-Assembled micro-CT/Self-Assembled micro CT_0226.tif]

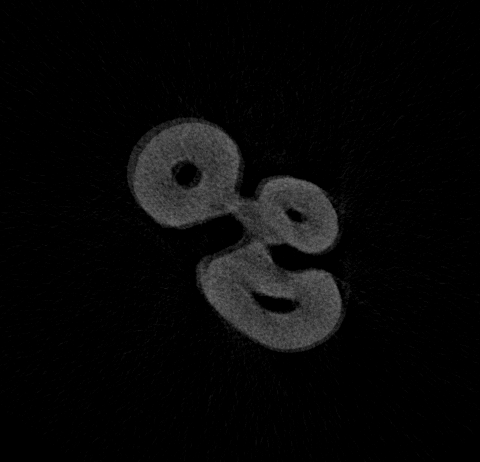

Supplement: S2 File — (ZIP) [file pone.0209698.s002.zip › Self-Assembled micro-CT/Self-Assembled micro CT_0227.tif]

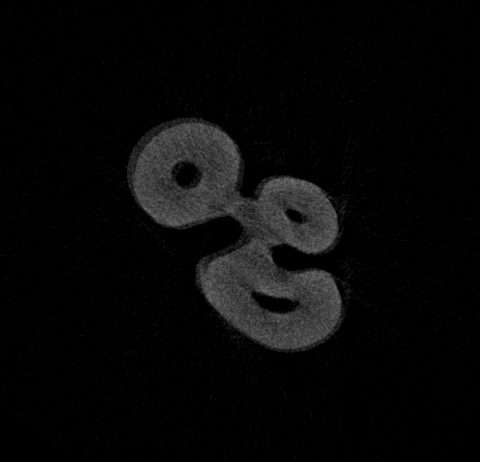

Supplement: S2 File — (ZIP) [file pone.0209698.s002.zip › Self-Assembled micro-CT/Self-Assembled micro CT_0228.tif]

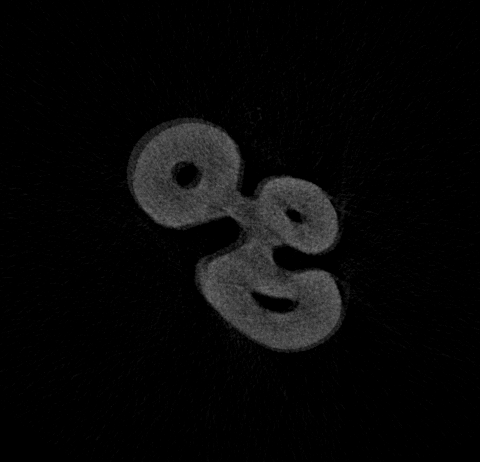

Supplement: S2 File — (ZIP) [file pone.0209698.s002.zip › Self-Assembled micro-CT/Self-Assembled micro CT_0229.tif]

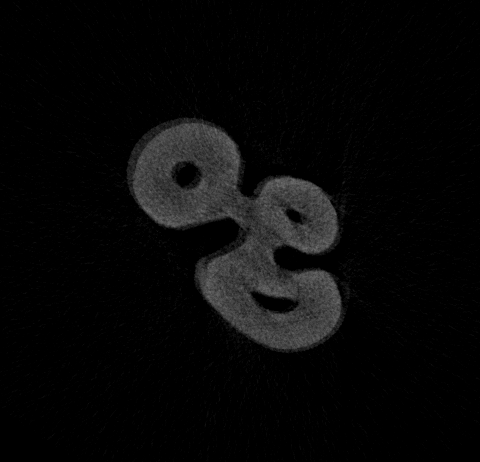

Supplement: S2 File — (ZIP) [file pone.0209698.s002.zip › Self-Assembled micro-CT/Self-Assembled micro CT_0230.tif]

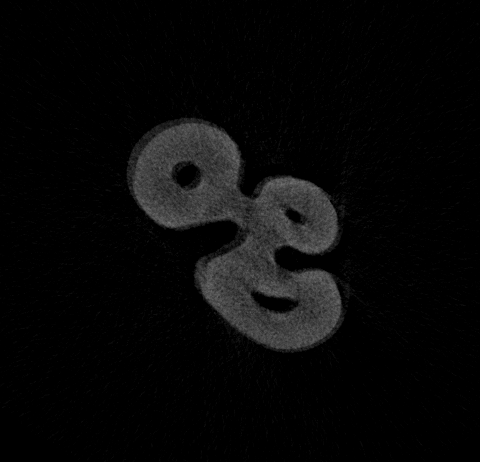

Supplement: S2 File — (ZIP) [file pone.0209698.s002.zip › Self-Assembled micro-CT/Self-Assembled micro CT_0231.tif]

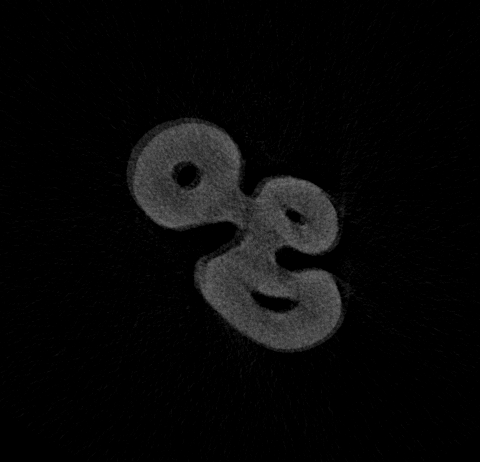

Supplement: S2 File — (ZIP) [file pone.0209698.s002.zip › Self-Assembled micro-CT/Self-Assembled micro CT_0232.tif]

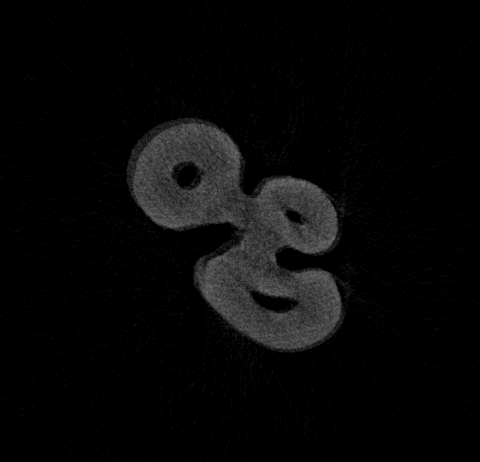

Supplement: S2 File — (ZIP) [file pone.0209698.s002.zip › Self-Assembled micro-CT/Self-Assembled micro CT_0233.tif]

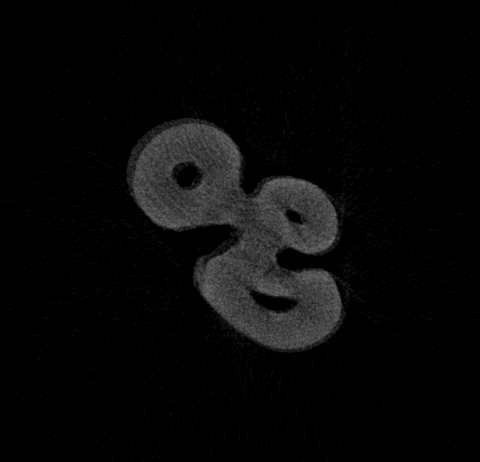

Supplement: S2 File — (ZIP) [file pone.0209698.s002.zip › Self-Assembled micro-CT/Self-Assembled micro CT_0234.tif]

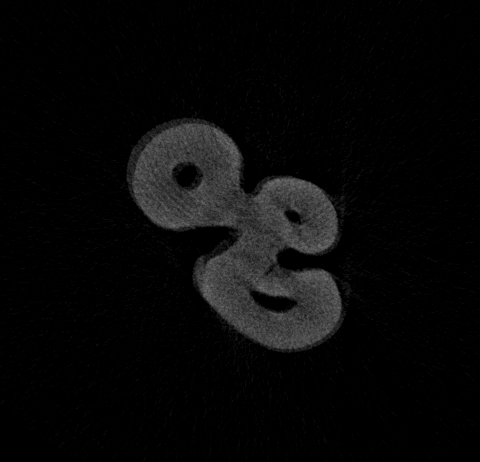

Supplement: S2 File — (ZIP) [file pone.0209698.s002.zip › Self-Assembled micro-CT/Self-Assembled micro CT_0235.tif]

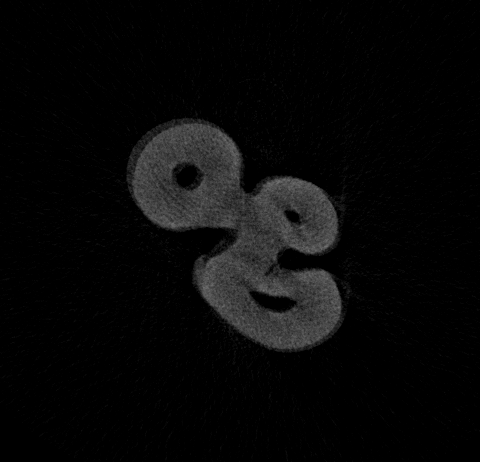

Supplement: S2 File — (ZIP) [file pone.0209698.s002.zip › Self-Assembled micro-CT/Self-Assembled micro CT_0236.tif]

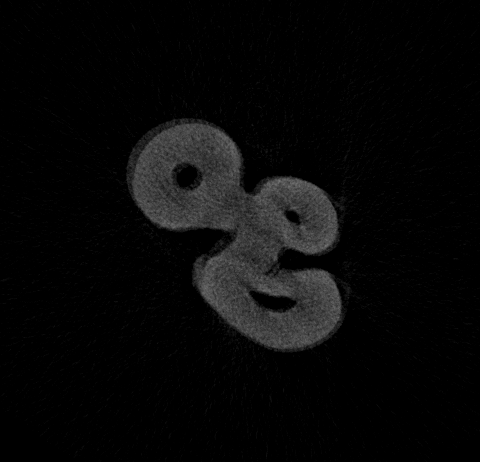

Supplement: S2 File — (ZIP) [file pone.0209698.s002.zip › Self-Assembled micro-CT/Self-Assembled micro CT_0237.tif]

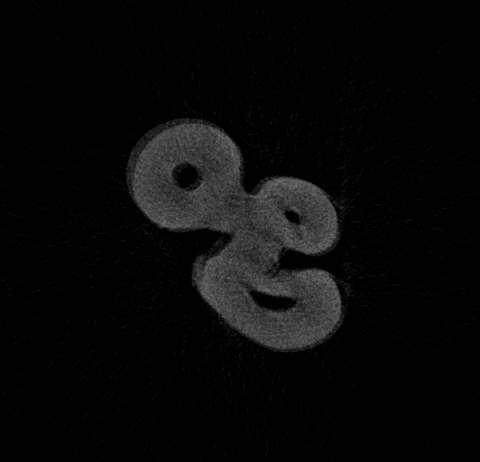

Supplement: S2 File — (ZIP) [file pone.0209698.s002.zip › Self-Assembled micro-CT/Self-Assembled micro CT_0238.tif]

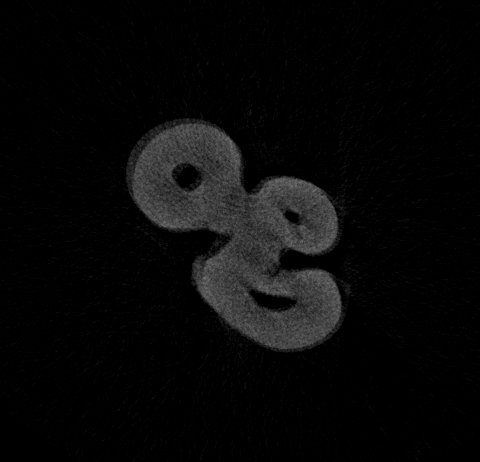

Supplement: S2 File — (ZIP) [file pone.0209698.s002.zip › Self-Assembled micro-CT/Self-Assembled micro CT_0239.tif]

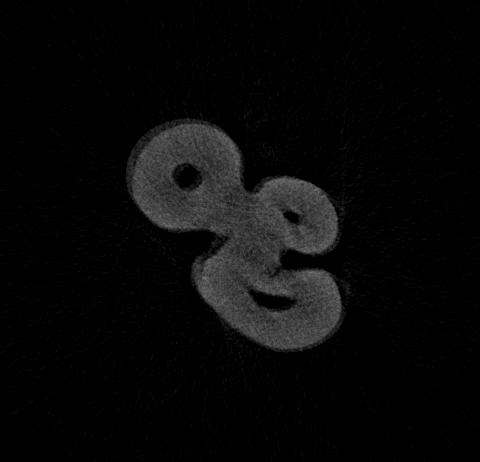

Supplement: S2 File — (ZIP) [file pone.0209698.s002.zip › Self-Assembled micro-CT/Self-Assembled micro CT_0240.tif]

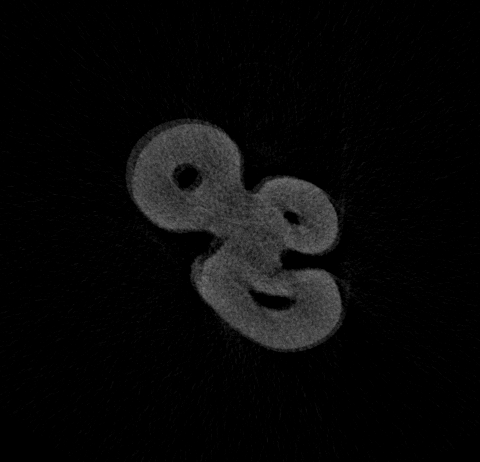

Supplement: S2 File — (ZIP) [file pone.0209698.s002.zip › Self-Assembled micro-CT/Self-Assembled micro CT_0241.tif]

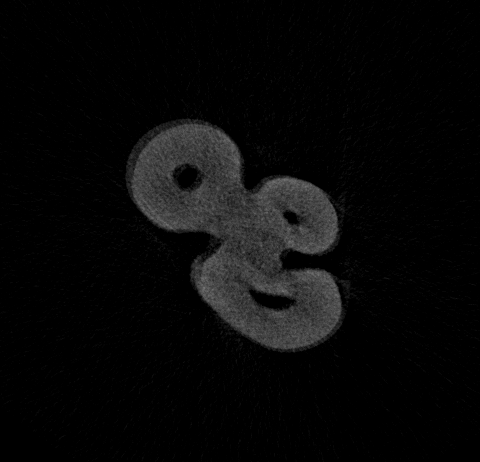

Supplement: S2 File — (ZIP) [file pone.0209698.s002.zip › Self-Assembled micro-CT/Self-Assembled micro CT_0242.tif]

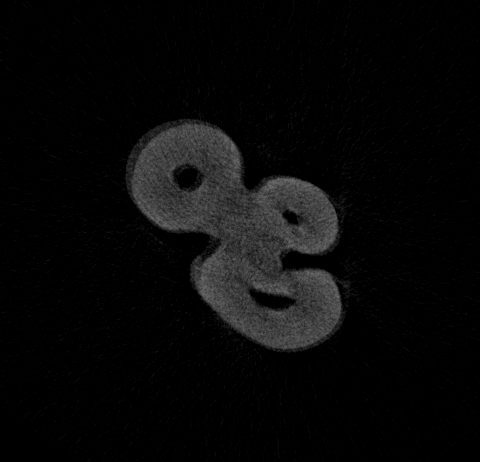

Supplement: S2 File — (ZIP) [file pone.0209698.s002.zip › Self-Assembled micro-CT/Self-Assembled micro CT_0243.tif]

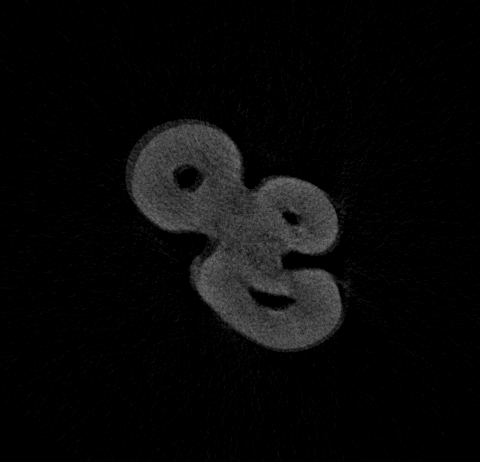

Supplement: S2 File — (ZIP) [file pone.0209698.s002.zip › Self-Assembled micro-CT/Self-Assembled micro CT_0244.tif]

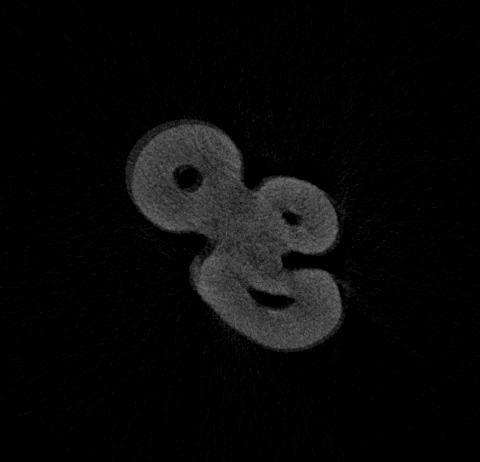

Supplement: S2 File — (ZIP) [file pone.0209698.s002.zip › Self-Assembled micro-CT/Self-Assembled micro CT_0245.tif]

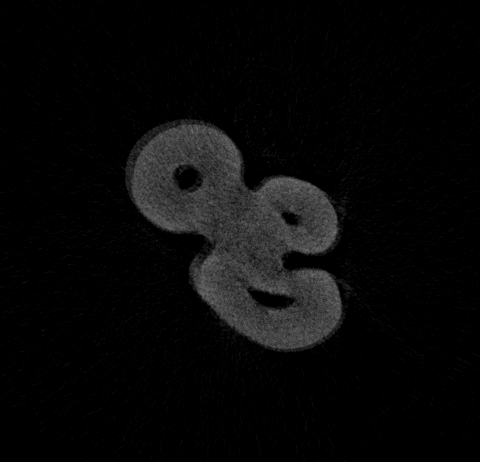

Supplement: S2 File — (ZIP) [file pone.0209698.s002.zip › Self-Assembled micro-CT/Self-Assembled micro CT_0246.tif]

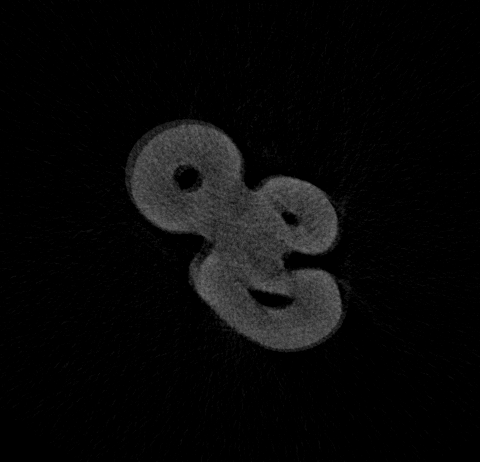

Supplement: S2 File — (ZIP) [file pone.0209698.s002.zip › Self-Assembled micro-CT/Self-Assembled micro CT_0247.tif]

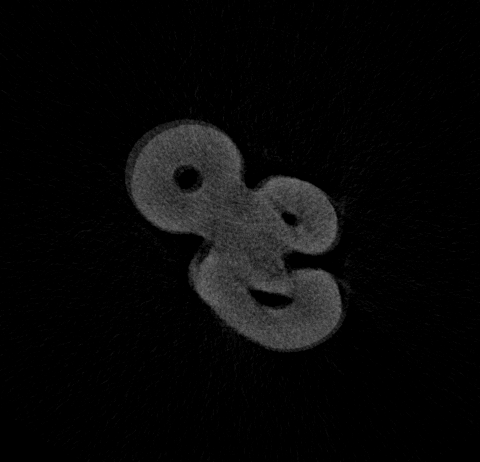

Supplement: S2 File — (ZIP) [file pone.0209698.s002.zip › Self-Assembled micro-CT/Self-Assembled micro CT_0248.tif]

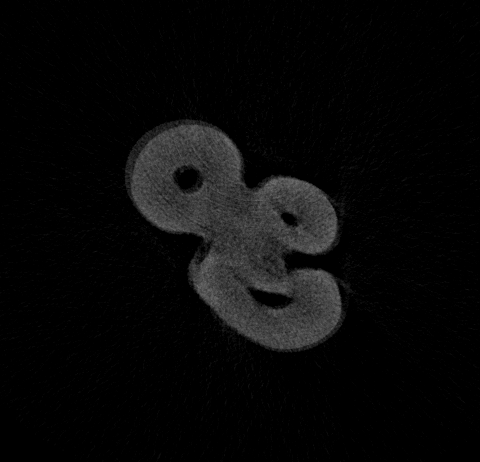

Supplement: S2 File — (ZIP) [file pone.0209698.s002.zip › Self-Assembled micro-CT/Self-Assembled micro CT_0249.tif]

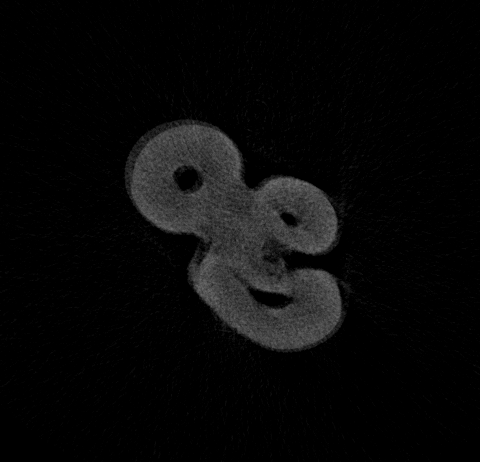

Supplement: S2 File — (ZIP) [file pone.0209698.s002.zip › Self-Assembled micro-CT/Self-Assembled micro CT_0250.tif]

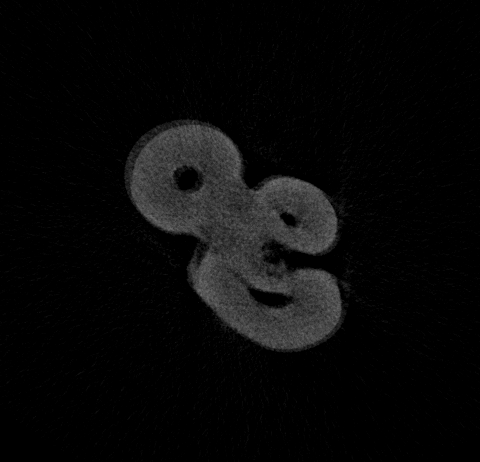

Supplement: S2 File — (ZIP) [file pone.0209698.s002.zip › Self-Assembled micro-CT/Self-Assembled micro CT_0251.tif]
