# Supplementary figures and images for: Self-assembled micro-computed tomography for dental education (part 3 of 4)
Source: PLoS One. 2018 Dec 26;13(12):e0209698. doi: 10.1371/journal.pone.0209698 (PMC6306236; doi:10.1371/journal.pone.0209698)

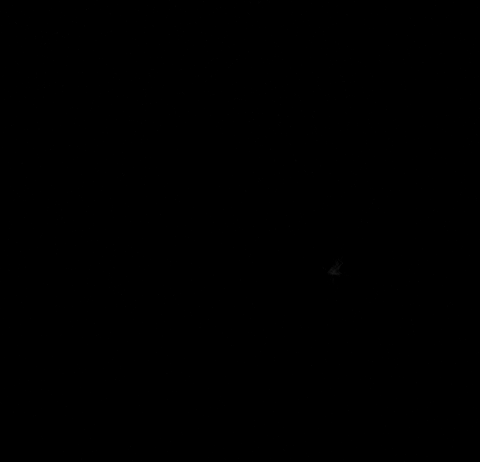

Supplement: S2 File — (ZIP) [file pone.0209698.s002.zip › Self-Assembled micro-CT/Self-Assembled micro CT_0052.tif]

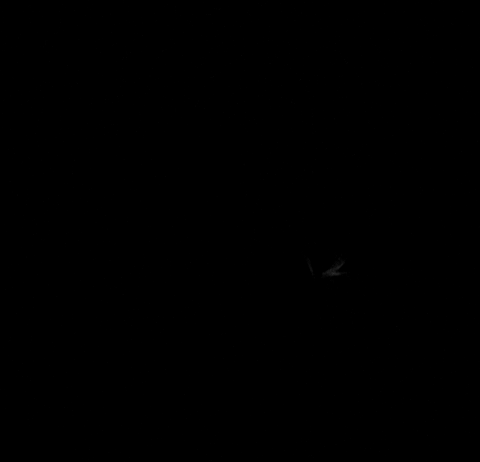

Supplement: S2 File — (ZIP) [file pone.0209698.s002.zip › Self-Assembled micro-CT/Self-Assembled micro CT_0053.tif]

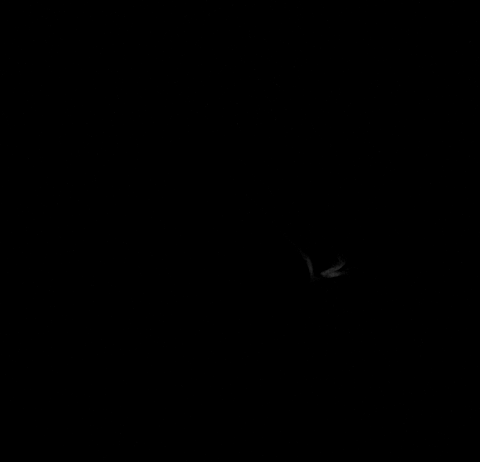

Supplement: S2 File — (ZIP) [file pone.0209698.s002.zip › Self-Assembled micro-CT/Self-Assembled micro CT_0054.tif]

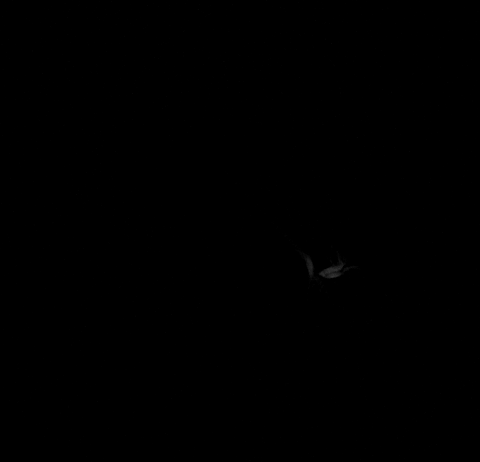

Supplement: S2 File — (ZIP) [file pone.0209698.s002.zip › Self-Assembled micro-CT/Self-Assembled micro CT_0055.tif]

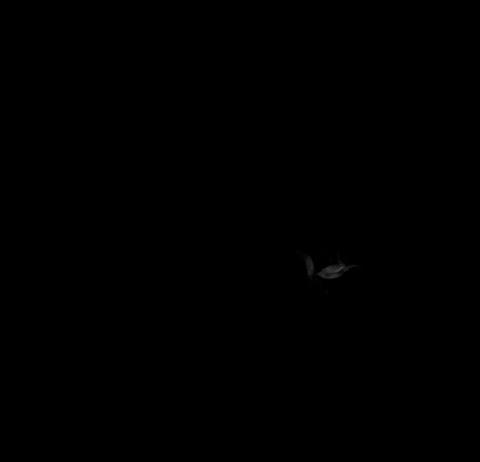

Supplement: S2 File — (ZIP) [file pone.0209698.s002.zip › Self-Assembled micro-CT/Self-Assembled micro CT_0056.tif]

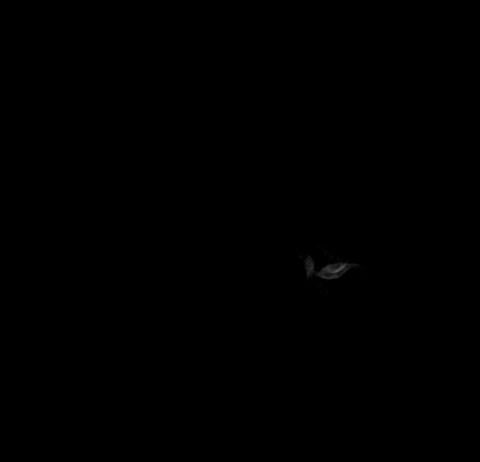

Supplement: S2 File — (ZIP) [file pone.0209698.s002.zip › Self-Assembled micro-CT/Self-Assembled micro CT_0057.tif]

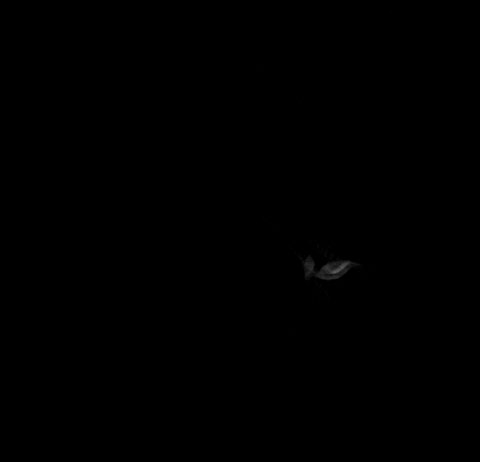

Supplement: S2 File — (ZIP) [file pone.0209698.s002.zip › Self-Assembled micro-CT/Self-Assembled micro CT_0058.tif]

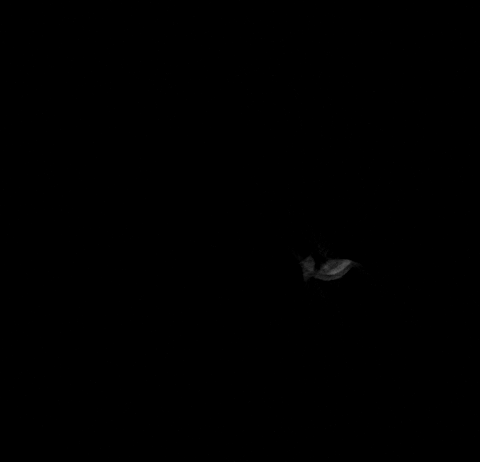

Supplement: S2 File — (ZIP) [file pone.0209698.s002.zip › Self-Assembled micro-CT/Self-Assembled micro CT_0059.tif]

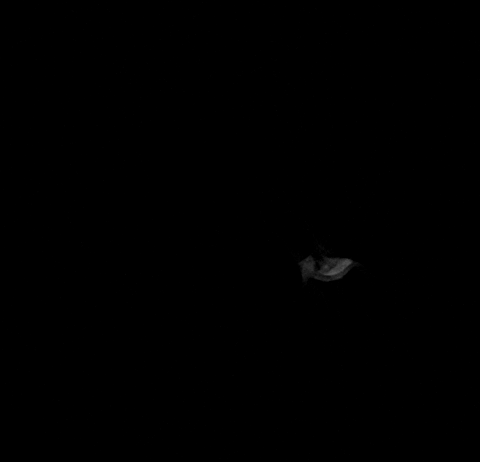

Supplement: S2 File — (ZIP) [file pone.0209698.s002.zip › Self-Assembled micro-CT/Self-Assembled micro CT_0060.tif]

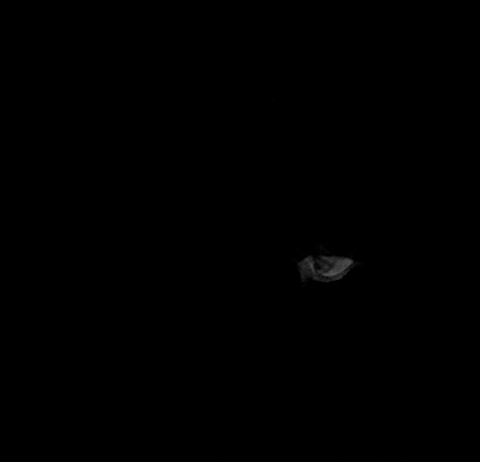

Supplement: S2 File — (ZIP) [file pone.0209698.s002.zip › Self-Assembled micro-CT/Self-Assembled micro CT_0061.tif]

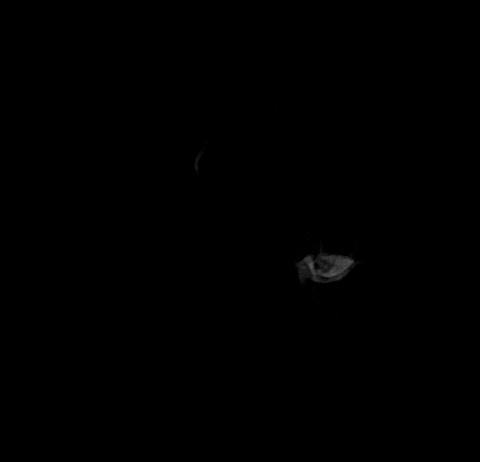

Supplement: S2 File — (ZIP) [file pone.0209698.s002.zip › Self-Assembled micro-CT/Self-Assembled micro CT_0062.tif]

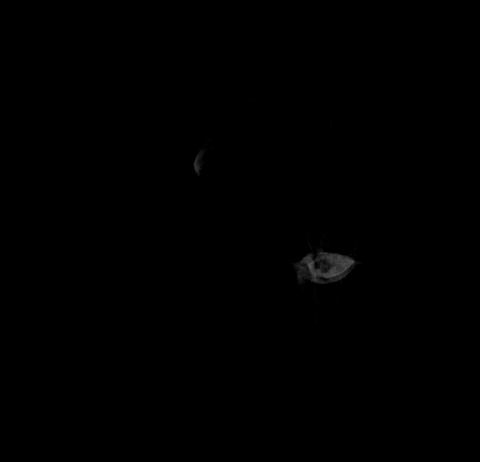

Supplement: S2 File — (ZIP) [file pone.0209698.s002.zip › Self-Assembled micro-CT/Self-Assembled micro CT_0063.tif]

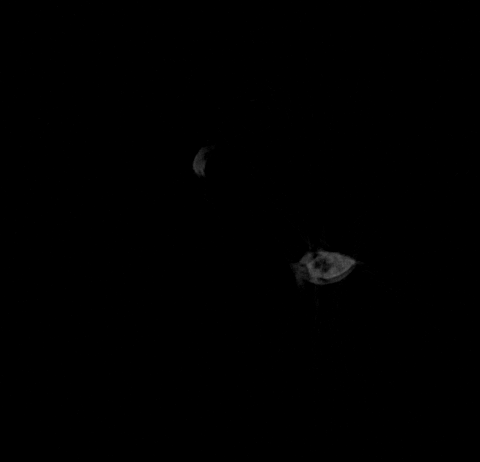

Supplement: S2 File — (ZIP) [file pone.0209698.s002.zip › Self-Assembled micro-CT/Self-Assembled micro CT_0064.tif]

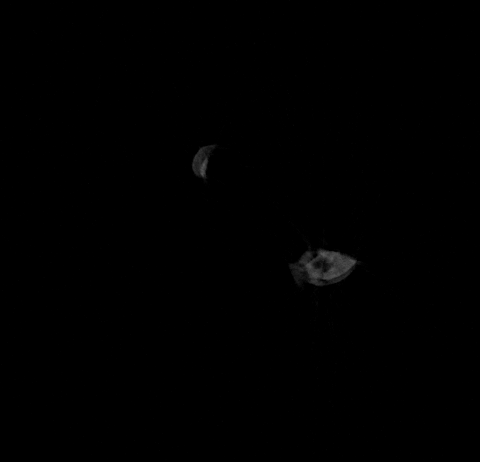

Supplement: S2 File — (ZIP) [file pone.0209698.s002.zip › Self-Assembled micro-CT/Self-Assembled micro CT_0065.tif]

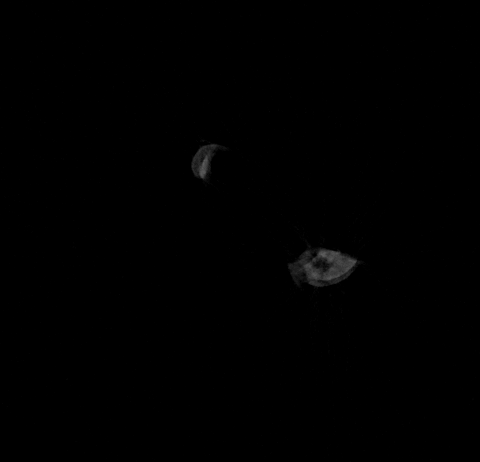

Supplement: S2 File — (ZIP) [file pone.0209698.s002.zip › Self-Assembled micro-CT/Self-Assembled micro CT_0066.tif]

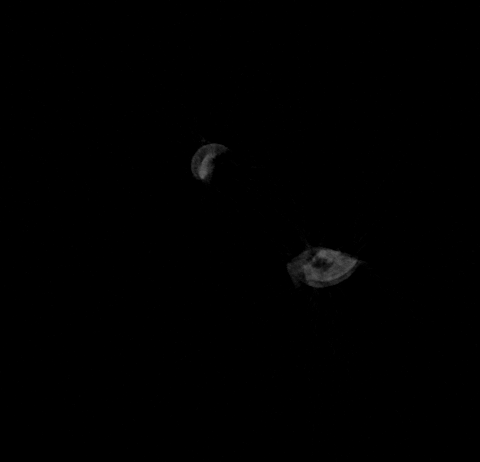

Supplement: S2 File — (ZIP) [file pone.0209698.s002.zip › Self-Assembled micro-CT/Self-Assembled micro CT_0067.tif]

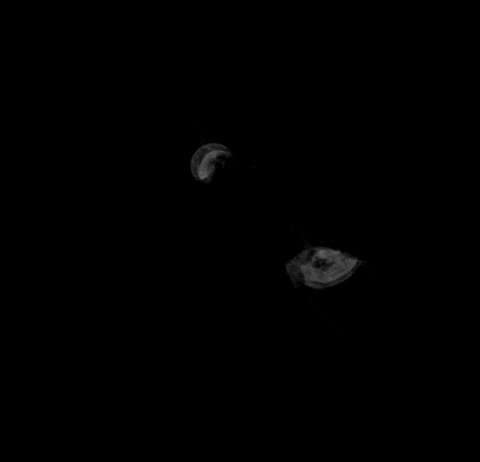

Supplement: S2 File — (ZIP) [file pone.0209698.s002.zip › Self-Assembled micro-CT/Self-Assembled micro CT_0068.tif]

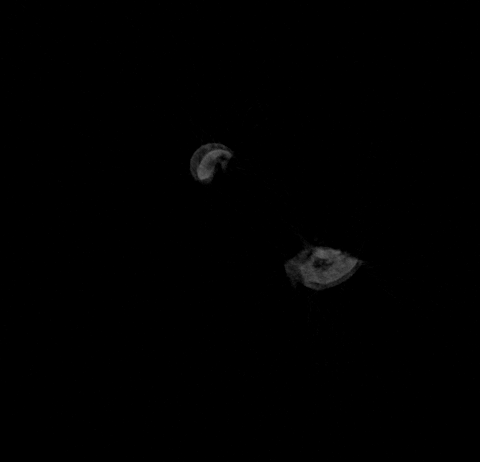

Supplement: S2 File — (ZIP) [file pone.0209698.s002.zip › Self-Assembled micro-CT/Self-Assembled micro CT_0069.tif]

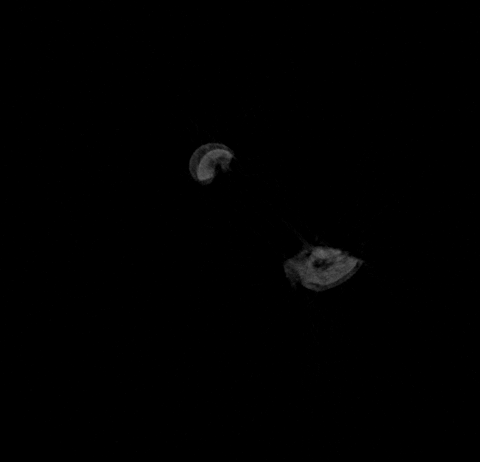

Supplement: S2 File — (ZIP) [file pone.0209698.s002.zip › Self-Assembled micro-CT/Self-Assembled micro CT_0070.tif]

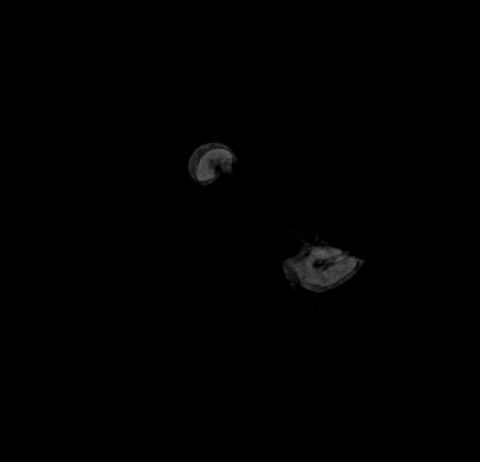

Supplement: S2 File — (ZIP) [file pone.0209698.s002.zip › Self-Assembled micro-CT/Self-Assembled micro CT_0071.tif]

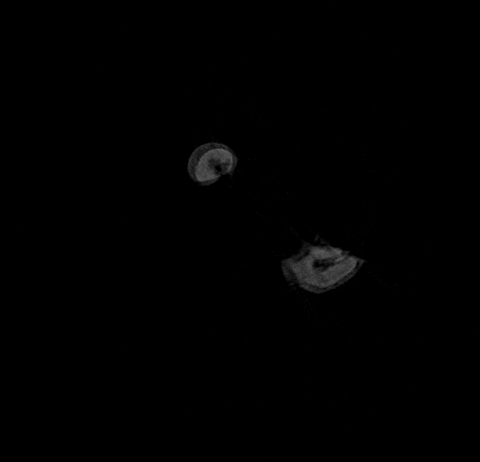

Supplement: S2 File — (ZIP) [file pone.0209698.s002.zip › Self-Assembled micro-CT/Self-Assembled micro CT_0072.tif]

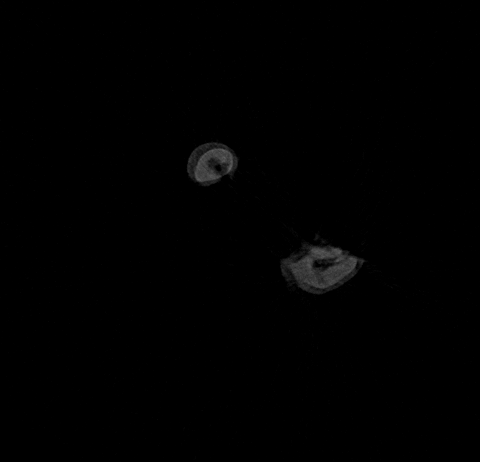

Supplement: S2 File — (ZIP) [file pone.0209698.s002.zip › Self-Assembled micro-CT/Self-Assembled micro CT_0073.tif]

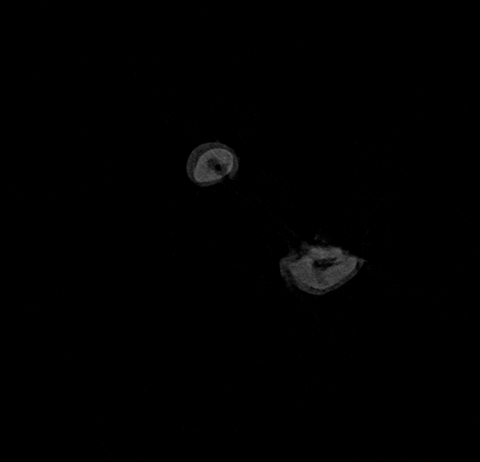

Supplement: S2 File — (ZIP) [file pone.0209698.s002.zip › Self-Assembled micro-CT/Self-Assembled micro CT_0074.tif]

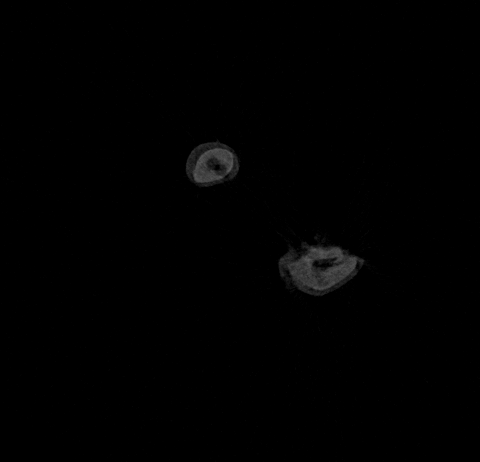

Supplement: S2 File — (ZIP) [file pone.0209698.s002.zip › Self-Assembled micro-CT/Self-Assembled micro CT_0075.tif]

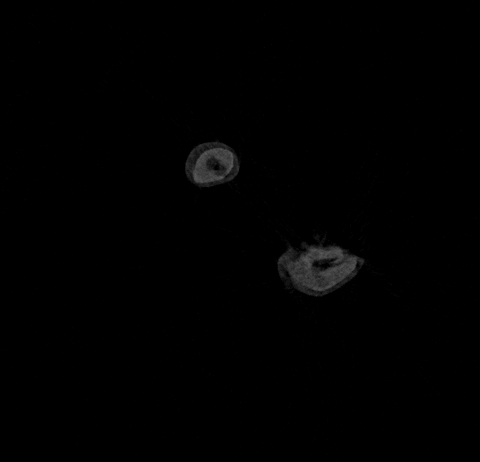

Supplement: S2 File — (ZIP) [file pone.0209698.s002.zip › Self-Assembled micro-CT/Self-Assembled micro CT_0076.tif]

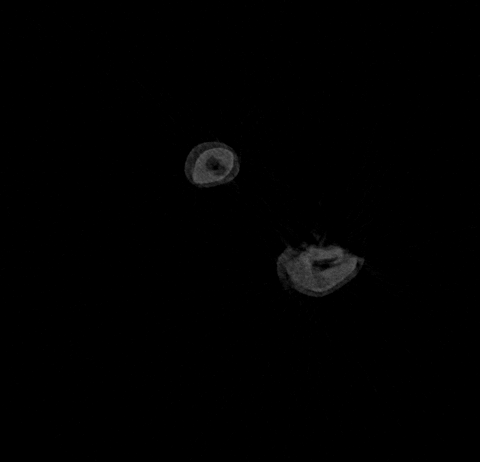

Supplement: S2 File — (ZIP) [file pone.0209698.s002.zip › Self-Assembled micro-CT/Self-Assembled micro CT_0077.tif]

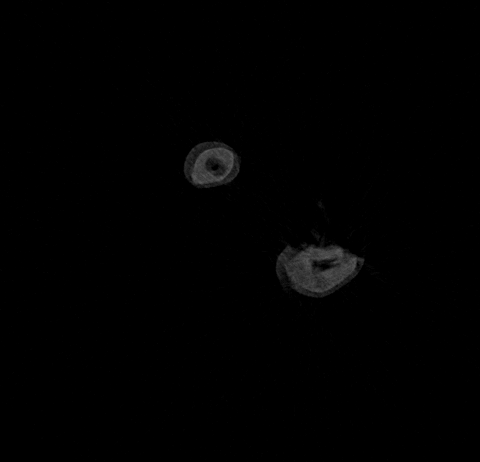

Supplement: S2 File — (ZIP) [file pone.0209698.s002.zip › Self-Assembled micro-CT/Self-Assembled micro CT_0078.tif]

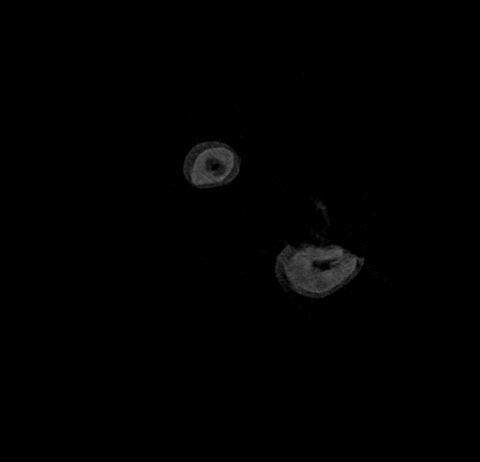

Supplement: S2 File — (ZIP) [file pone.0209698.s002.zip › Self-Assembled micro-CT/Self-Assembled micro CT_0079.tif]

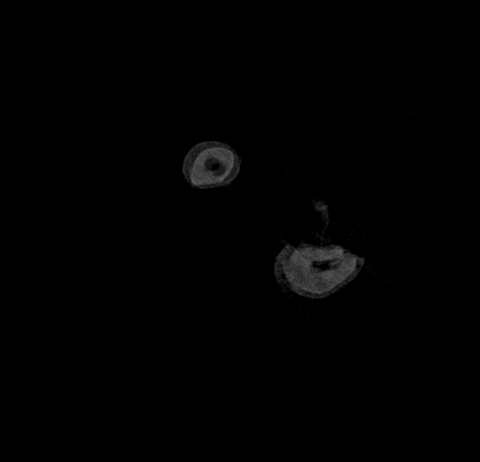

Supplement: S2 File — (ZIP) [file pone.0209698.s002.zip › Self-Assembled micro-CT/Self-Assembled micro CT_0080.tif]

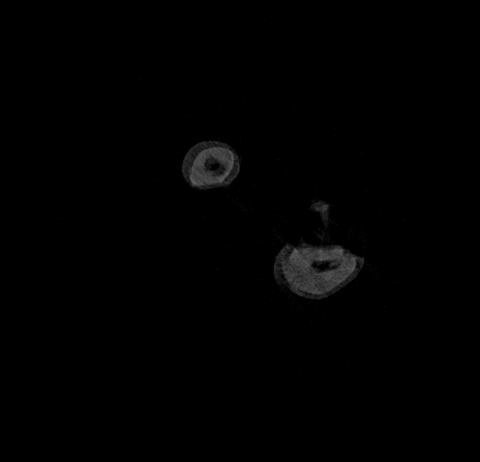

Supplement: S2 File — (ZIP) [file pone.0209698.s002.zip › Self-Assembled micro-CT/Self-Assembled micro CT_0081.tif]

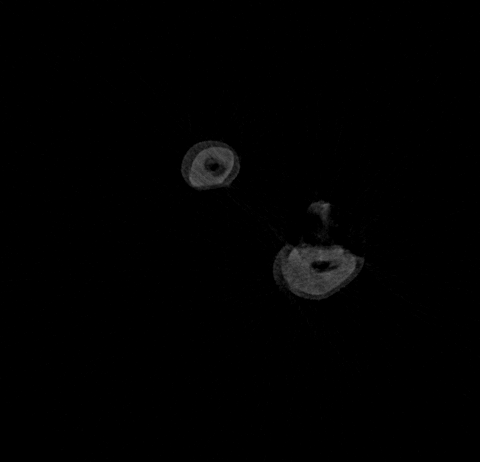

Supplement: S2 File — (ZIP) [file pone.0209698.s002.zip › Self-Assembled micro-CT/Self-Assembled micro CT_0082.tif]

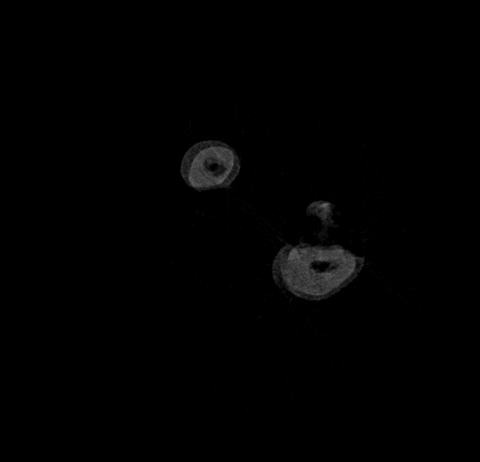

Supplement: S2 File — (ZIP) [file pone.0209698.s002.zip › Self-Assembled micro-CT/Self-Assembled micro CT_0083.tif]

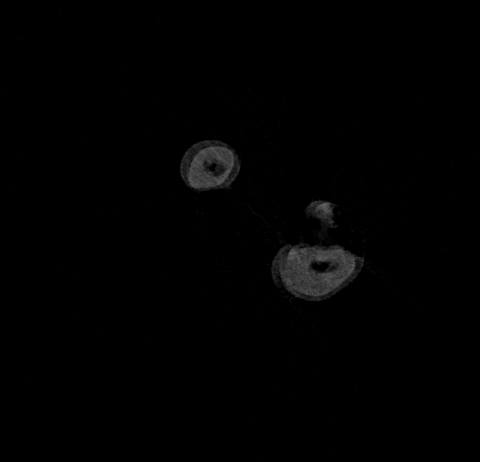

Supplement: S2 File — (ZIP) [file pone.0209698.s002.zip › Self-Assembled micro-CT/Self-Assembled micro CT_0084.tif]

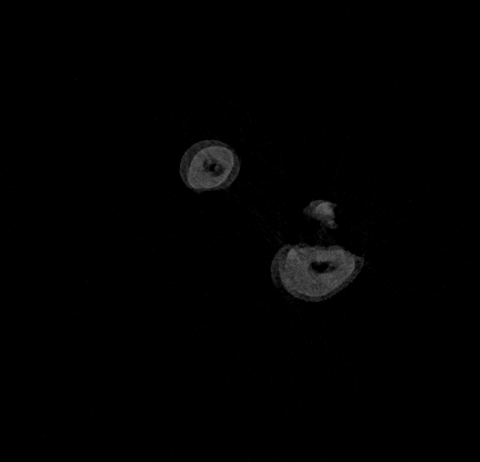

Supplement: S2 File — (ZIP) [file pone.0209698.s002.zip › Self-Assembled micro-CT/Self-Assembled micro CT_0085.tif]

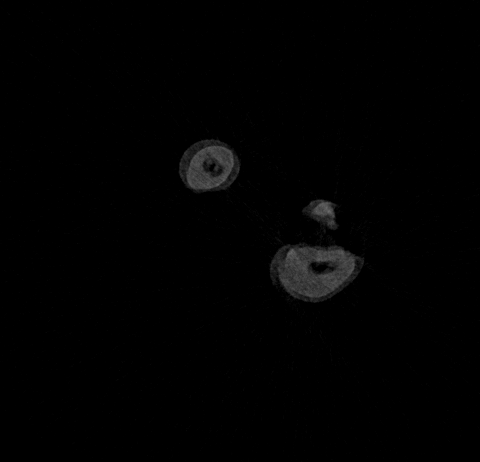

Supplement: S2 File — (ZIP) [file pone.0209698.s002.zip › Self-Assembled micro-CT/Self-Assembled micro CT_0086.tif]

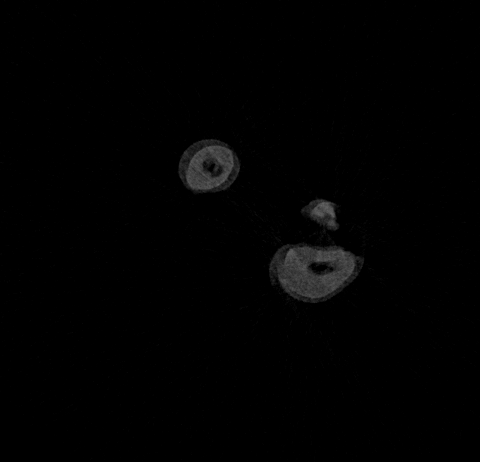

Supplement: S2 File — (ZIP) [file pone.0209698.s002.zip › Self-Assembled micro-CT/Self-Assembled micro CT_0087.tif]

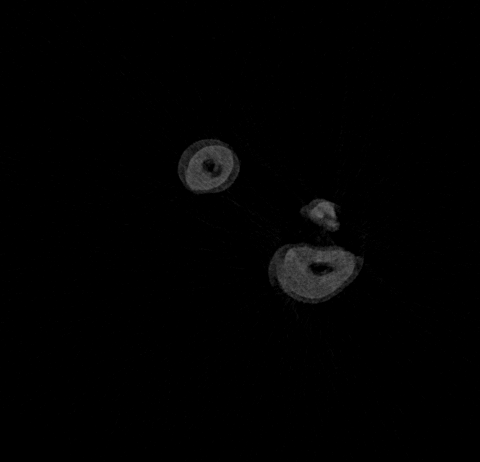

Supplement: S2 File — (ZIP) [file pone.0209698.s002.zip › Self-Assembled micro-CT/Self-Assembled micro CT_0088.tif]

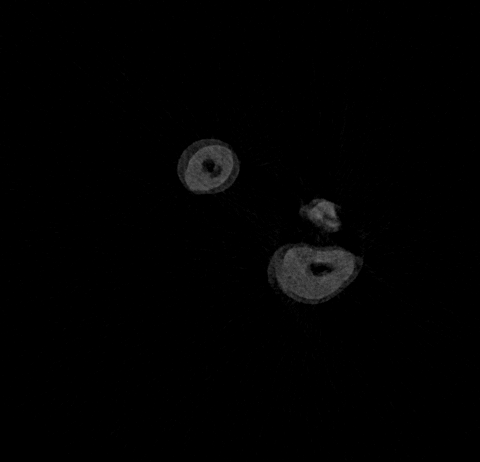

Supplement: S2 File — (ZIP) [file pone.0209698.s002.zip › Self-Assembled micro-CT/Self-Assembled micro CT_0089.tif]

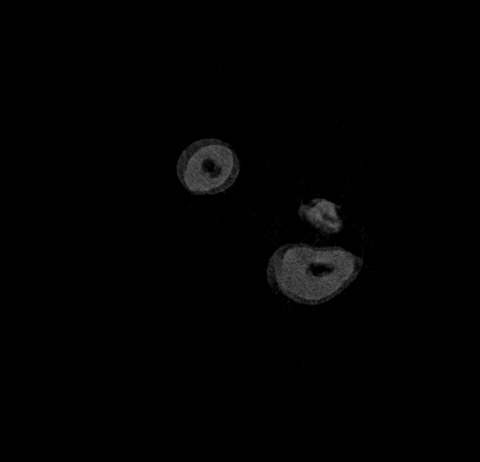

Supplement: S2 File — (ZIP) [file pone.0209698.s002.zip › Self-Assembled micro-CT/Self-Assembled micro CT_0090.tif]

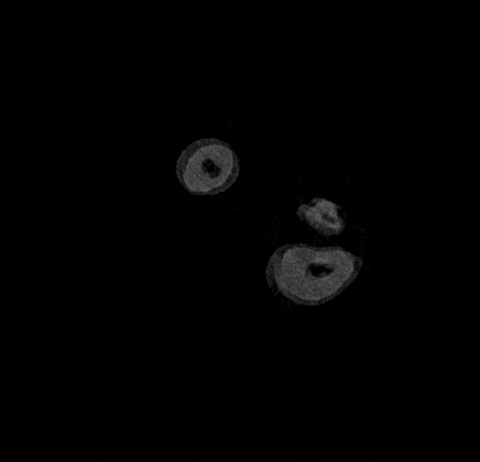

Supplement: S2 File — (ZIP) [file pone.0209698.s002.zip › Self-Assembled micro-CT/Self-Assembled micro CT_0091.tif]

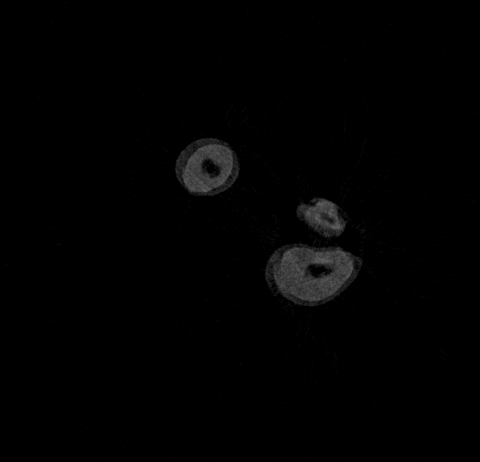

Supplement: S2 File — (ZIP) [file pone.0209698.s002.zip › Self-Assembled micro-CT/Self-Assembled micro CT_0092.tif]

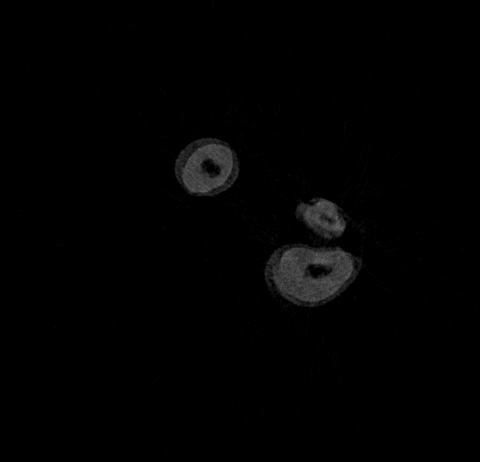

Supplement: S2 File — (ZIP) [file pone.0209698.s002.zip › Self-Assembled micro-CT/Self-Assembled micro CT_0093.tif]

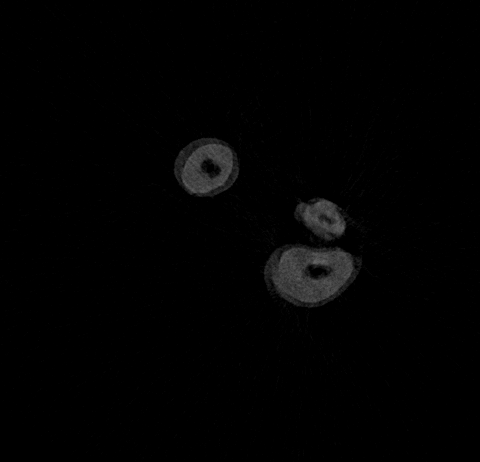

Supplement: S2 File — (ZIP) [file pone.0209698.s002.zip › Self-Assembled micro-CT/Self-Assembled micro CT_0094.tif]

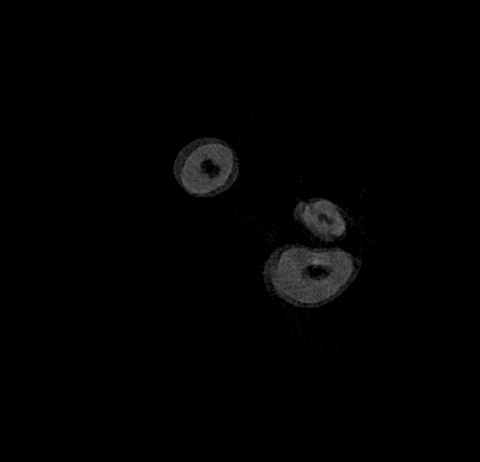

Supplement: S2 File — (ZIP) [file pone.0209698.s002.zip › Self-Assembled micro-CT/Self-Assembled micro CT_0095.tif]

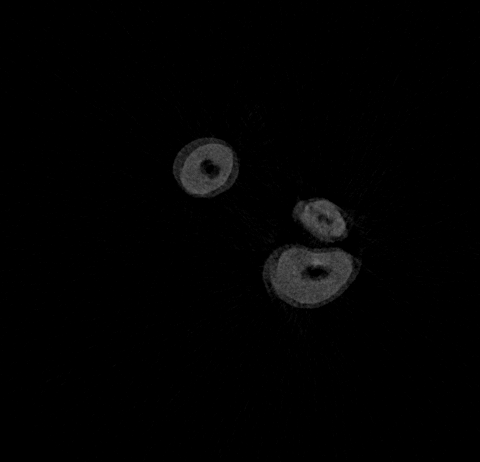

Supplement: S2 File — (ZIP) [file pone.0209698.s002.zip › Self-Assembled micro-CT/Self-Assembled micro CT_0096.tif]

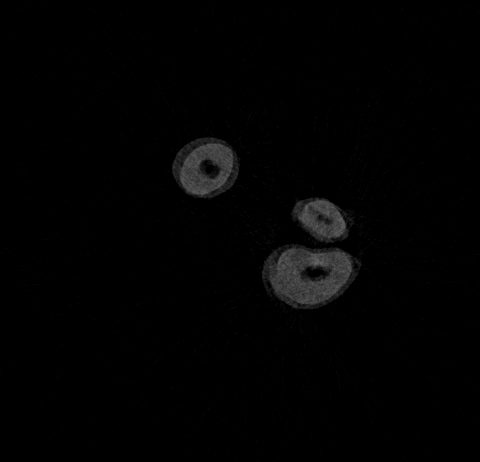

Supplement: S2 File — (ZIP) [file pone.0209698.s002.zip › Self-Assembled micro-CT/Self-Assembled micro CT_0097.tif]

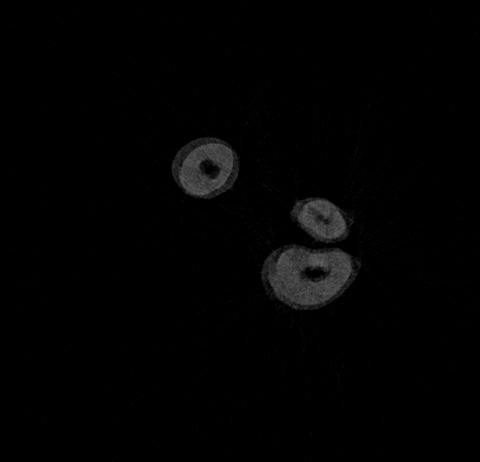

Supplement: S2 File — (ZIP) [file pone.0209698.s002.zip › Self-Assembled micro-CT/Self-Assembled micro CT_0098.tif]

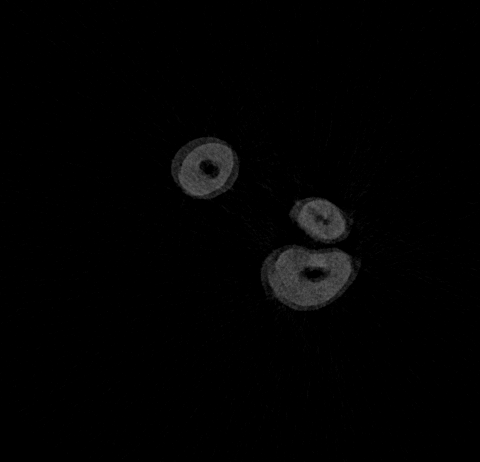

Supplement: S2 File — (ZIP) [file pone.0209698.s002.zip › Self-Assembled micro-CT/Self-Assembled micro CT_0099.tif]

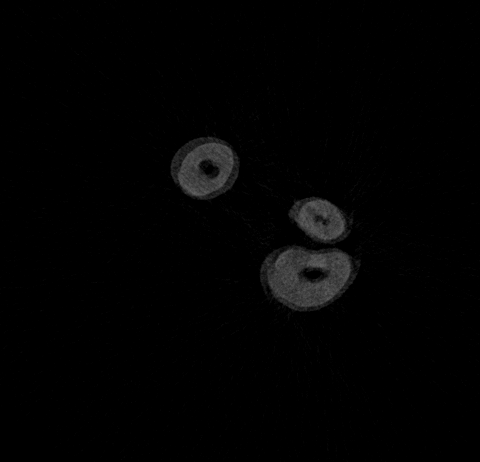

Supplement: S2 File — (ZIP) [file pone.0209698.s002.zip › Self-Assembled micro-CT/Self-Assembled micro CT_0100.tif]

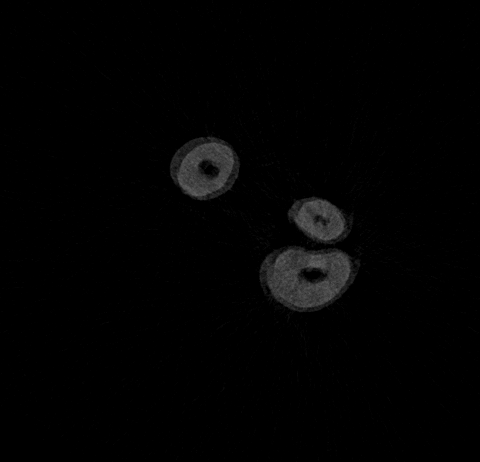

Supplement: S2 File — (ZIP) [file pone.0209698.s002.zip › Self-Assembled micro-CT/Self-Assembled micro CT_0101.tif]

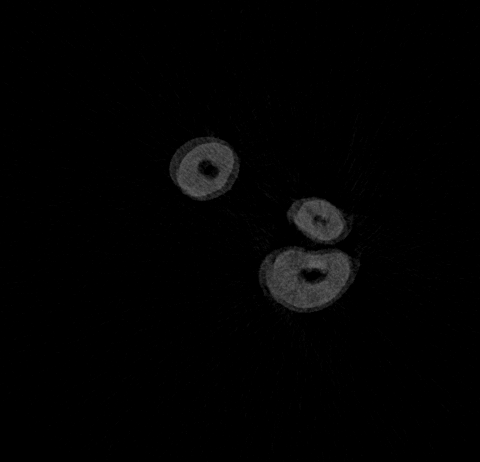

Supplement: S2 File — (ZIP) [file pone.0209698.s002.zip › Self-Assembled micro-CT/Self-Assembled micro CT_0102.tif]

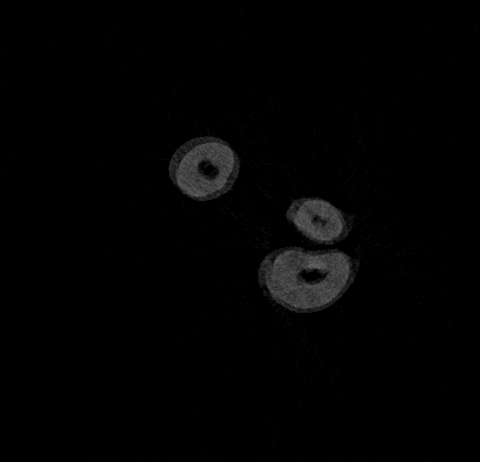

Supplement: S2 File — (ZIP) [file pone.0209698.s002.zip › Self-Assembled micro-CT/Self-Assembled micro CT_0103.tif]

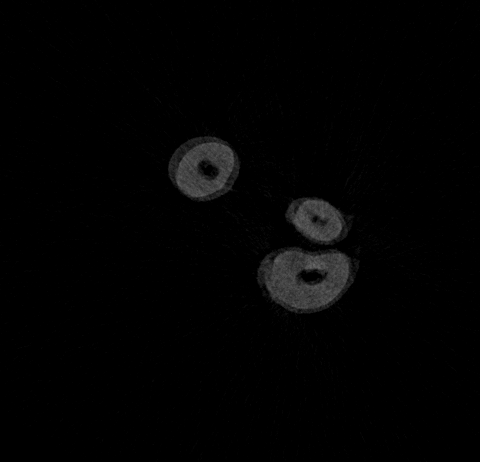

Supplement: S2 File — (ZIP) [file pone.0209698.s002.zip › Self-Assembled micro-CT/Self-Assembled micro CT_0104.tif]

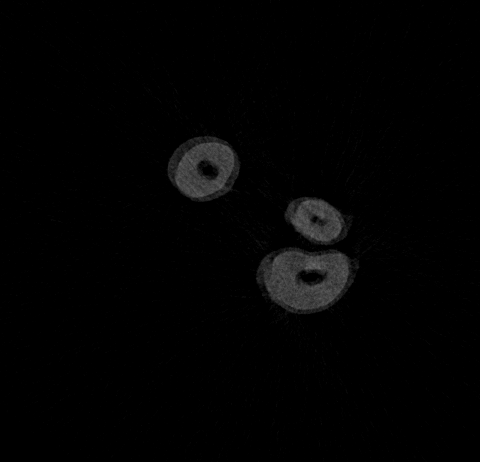

Supplement: S2 File — (ZIP) [file pone.0209698.s002.zip › Self-Assembled micro-CT/Self-Assembled micro CT_0105.tif]

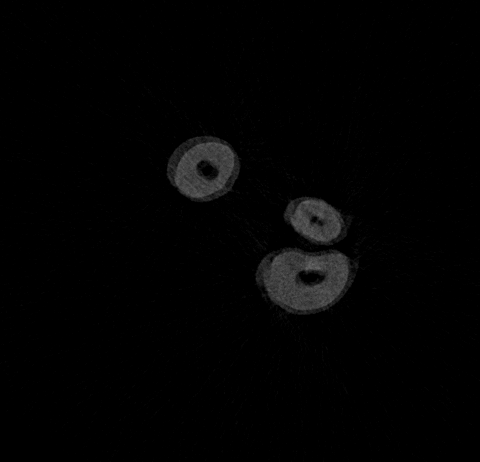

Supplement: S2 File — (ZIP) [file pone.0209698.s002.zip › Self-Assembled micro-CT/Self-Assembled micro CT_0106.tif]

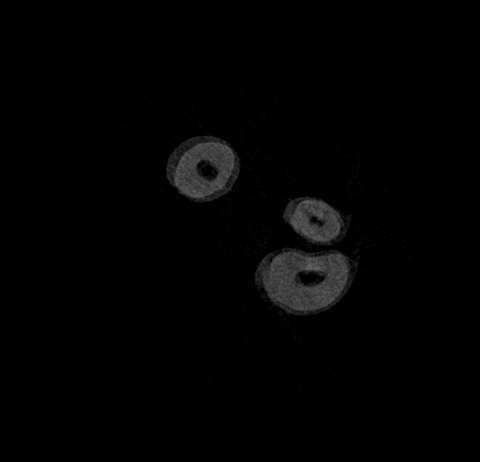

Supplement: S2 File — (ZIP) [file pone.0209698.s002.zip › Self-Assembled micro-CT/Self-Assembled micro CT_0107.tif]

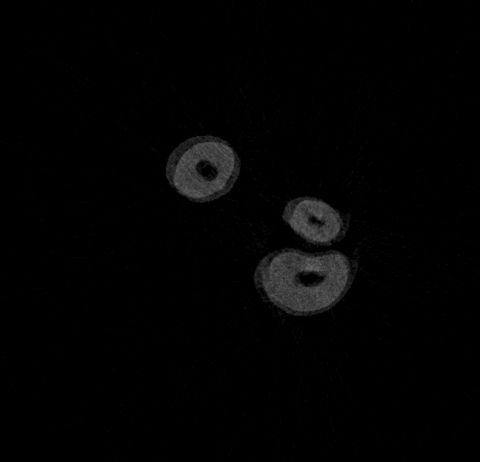

Supplement: S2 File — (ZIP) [file pone.0209698.s002.zip › Self-Assembled micro-CT/Self-Assembled micro CT_0108.tif]

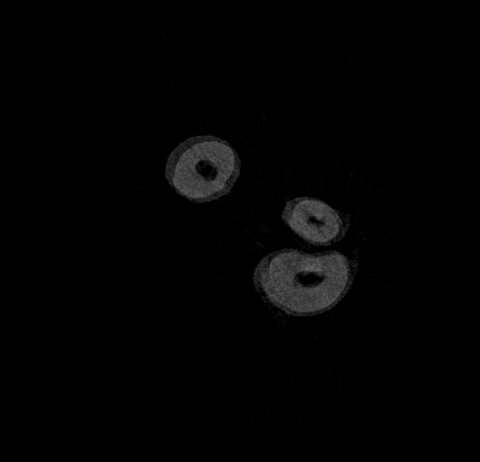

Supplement: S2 File — (ZIP) [file pone.0209698.s002.zip › Self-Assembled micro-CT/Self-Assembled micro CT_0109.tif]

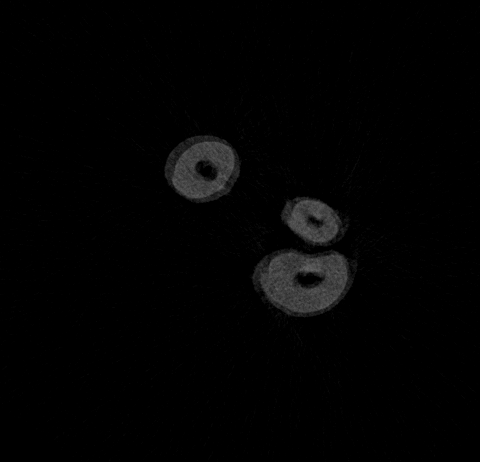

Supplement: S2 File — (ZIP) [file pone.0209698.s002.zip › Self-Assembled micro-CT/Self-Assembled micro CT_0110.tif]

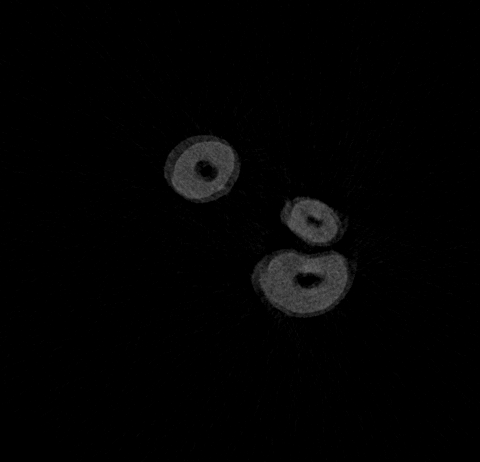

Supplement: S2 File — (ZIP) [file pone.0209698.s002.zip › Self-Assembled micro-CT/Self-Assembled micro CT_0111.tif]

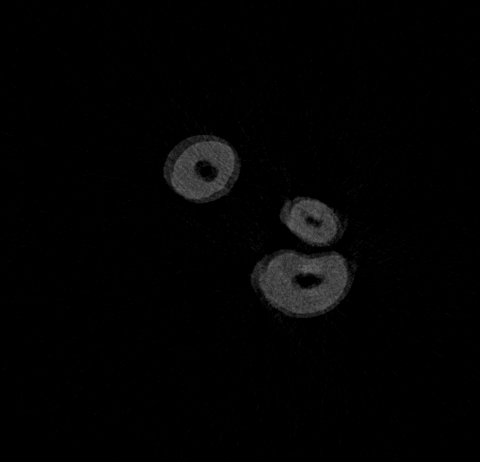

Supplement: S2 File — (ZIP) [file pone.0209698.s002.zip › Self-Assembled micro-CT/Self-Assembled micro CT_0112.tif]

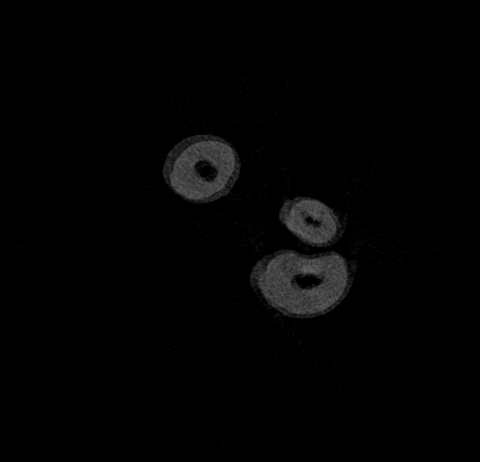

Supplement: S2 File — (ZIP) [file pone.0209698.s002.zip › Self-Assembled micro-CT/Self-Assembled micro CT_0113.tif]

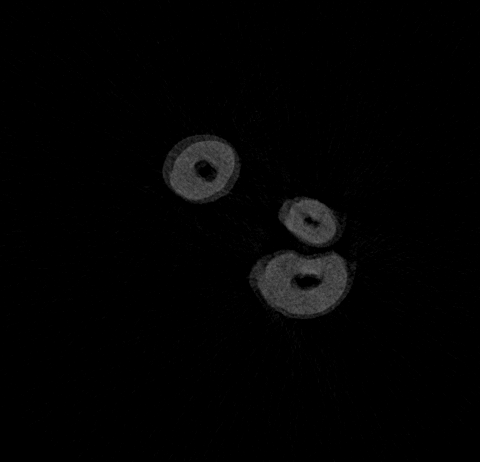

Supplement: S2 File — (ZIP) [file pone.0209698.s002.zip › Self-Assembled micro-CT/Self-Assembled micro CT_0114.tif]

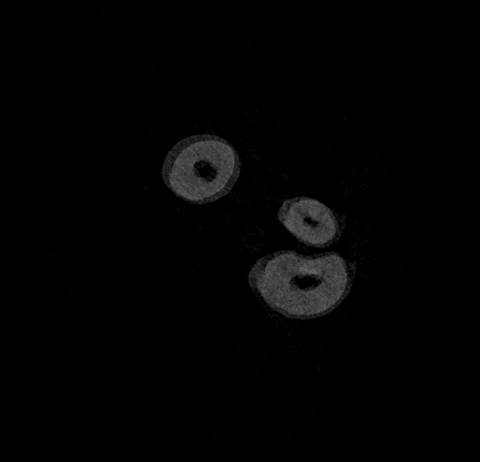

Supplement: S2 File — (ZIP) [file pone.0209698.s002.zip › Self-Assembled micro-CT/Self-Assembled micro CT_0115.tif]

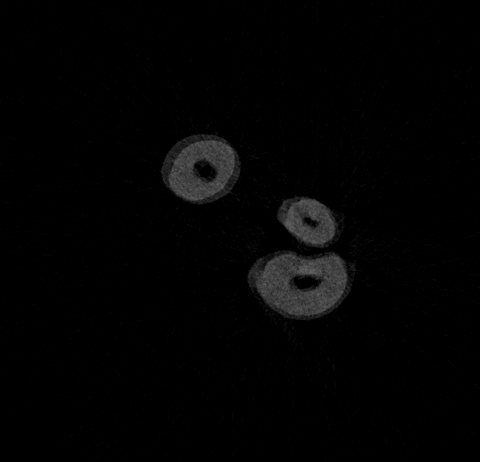

Supplement: S2 File — (ZIP) [file pone.0209698.s002.zip › Self-Assembled micro-CT/Self-Assembled micro CT_0116.tif]

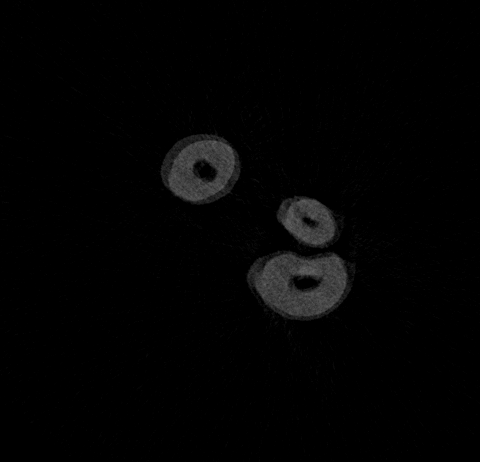

Supplement: S2 File — (ZIP) [file pone.0209698.s002.zip › Self-Assembled micro-CT/Self-Assembled micro CT_0117.tif]

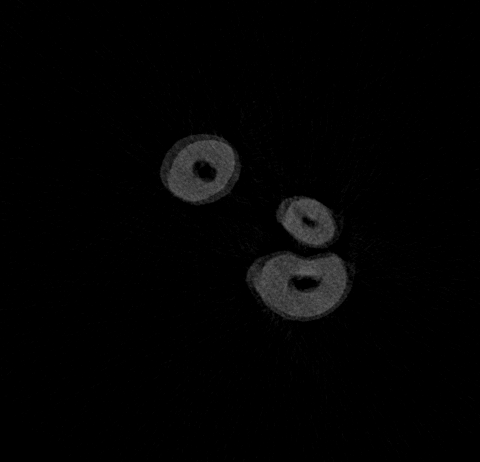

Supplement: S2 File — (ZIP) [file pone.0209698.s002.zip › Self-Assembled micro-CT/Self-Assembled micro CT_0118.tif]

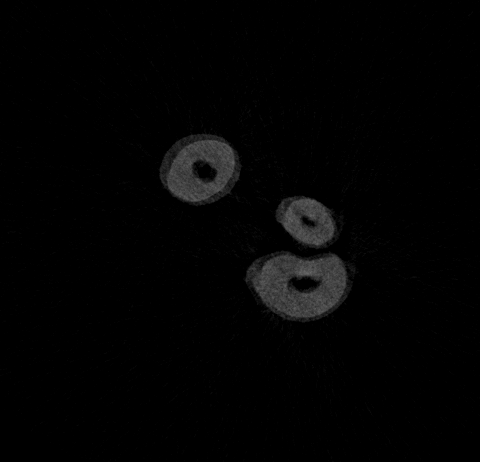

Supplement: S2 File — (ZIP) [file pone.0209698.s002.zip › Self-Assembled micro-CT/Self-Assembled micro CT_0119.tif]

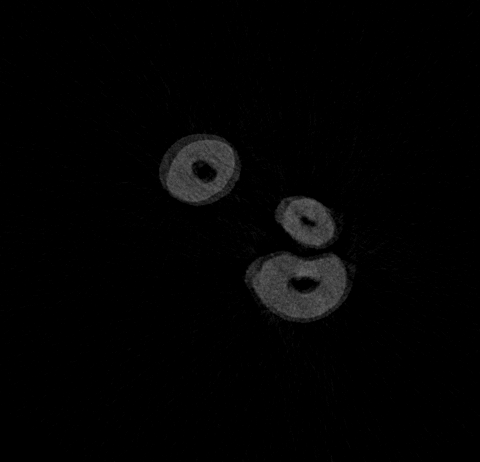

Supplement: S2 File — (ZIP) [file pone.0209698.s002.zip › Self-Assembled micro-CT/Self-Assembled micro CT_0120.tif]

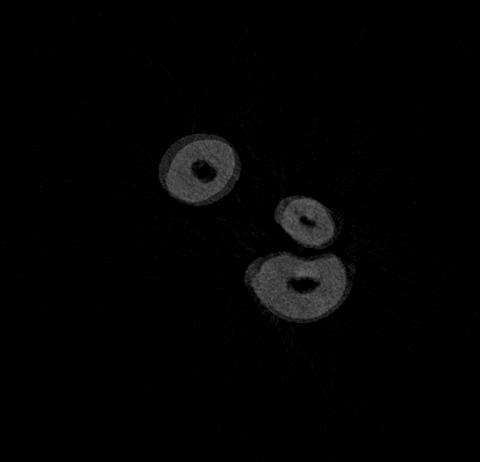

Supplement: S2 File — (ZIP) [file pone.0209698.s002.zip › Self-Assembled micro-CT/Self-Assembled micro CT_0121.tif]

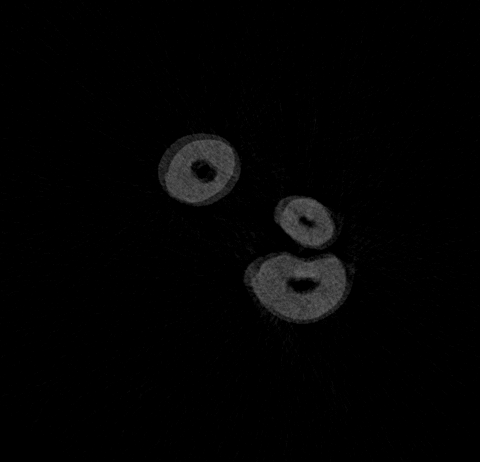

Supplement: S2 File — (ZIP) [file pone.0209698.s002.zip › Self-Assembled micro-CT/Self-Assembled micro CT_0122.tif]

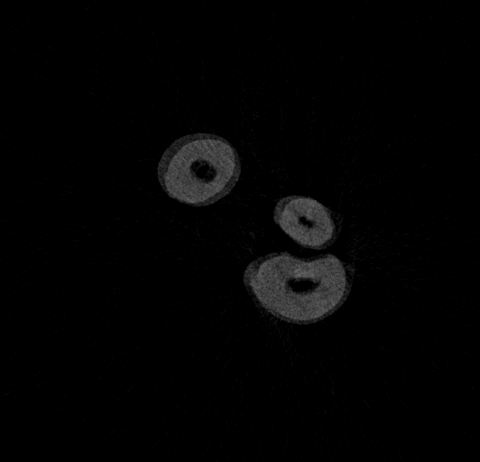

Supplement: S2 File — (ZIP) [file pone.0209698.s002.zip › Self-Assembled micro-CT/Self-Assembled micro CT_0123.tif]

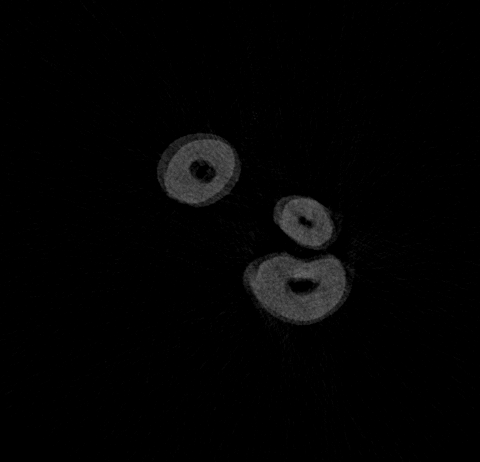

Supplement: S2 File — (ZIP) [file pone.0209698.s002.zip › Self-Assembled micro-CT/Self-Assembled micro CT_0124.tif]

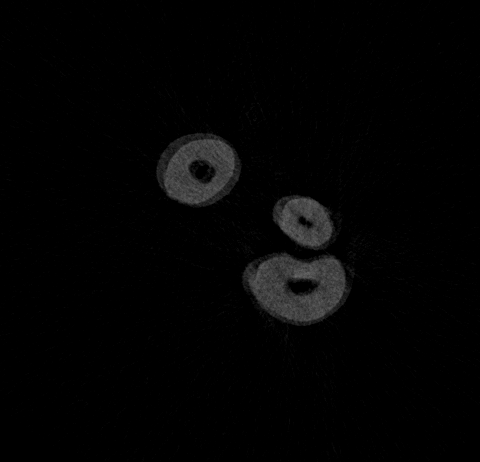

Supplement: S2 File — (ZIP) [file pone.0209698.s002.zip › Self-Assembled micro-CT/Self-Assembled micro CT_0125.tif]

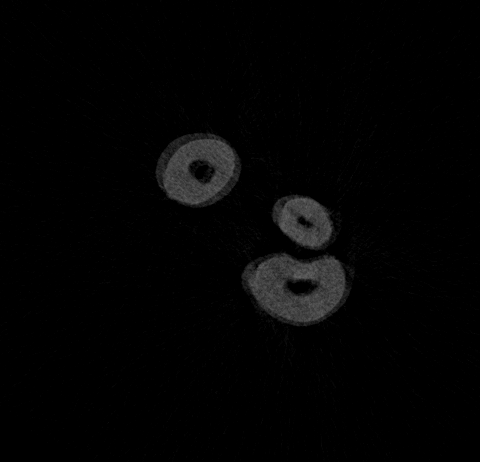

Supplement: S2 File — (ZIP) [file pone.0209698.s002.zip › Self-Assembled micro-CT/Self-Assembled micro CT_0126.tif]

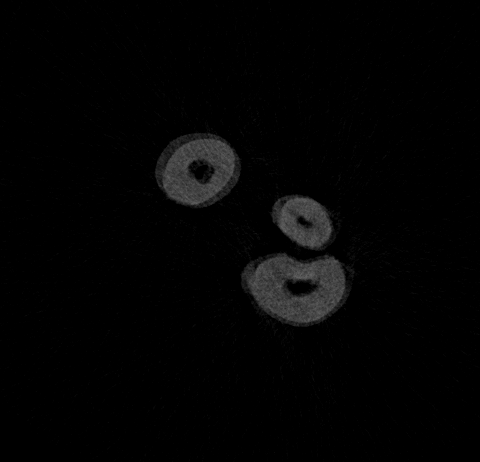

Supplement: S2 File — (ZIP) [file pone.0209698.s002.zip › Self-Assembled micro-CT/Self-Assembled micro CT_0127.tif]

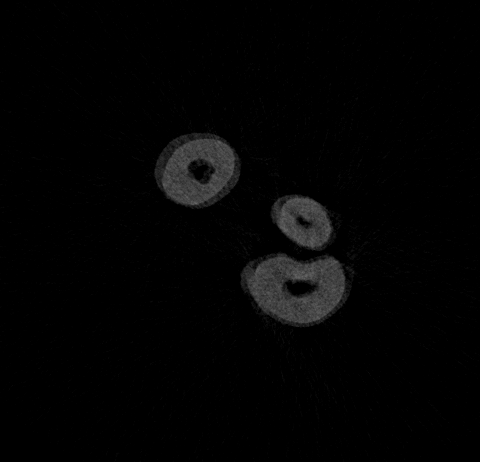

Supplement: S2 File — (ZIP) [file pone.0209698.s002.zip › Self-Assembled micro-CT/Self-Assembled micro CT_0128.tif]

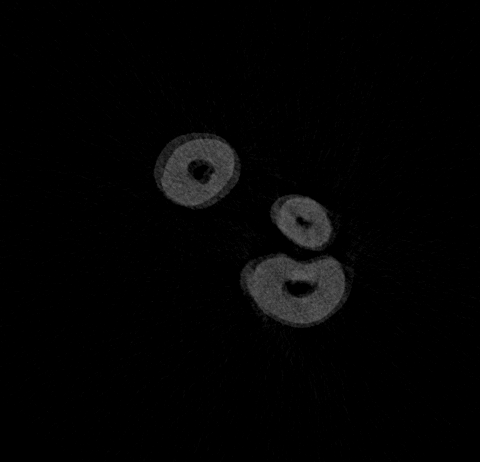

Supplement: S2 File — (ZIP) [file pone.0209698.s002.zip › Self-Assembled micro-CT/Self-Assembled micro CT_0129.tif]

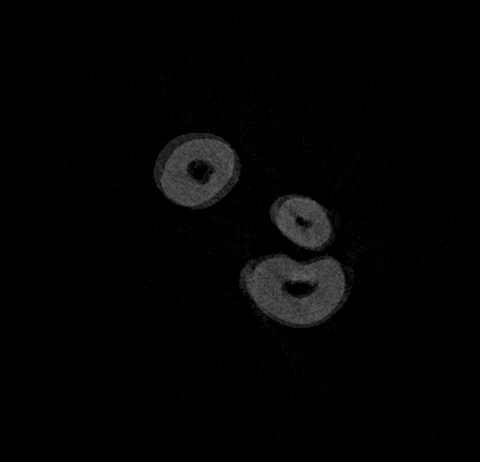

Supplement: S2 File — (ZIP) [file pone.0209698.s002.zip › Self-Assembled micro-CT/Self-Assembled micro CT_0130.tif]

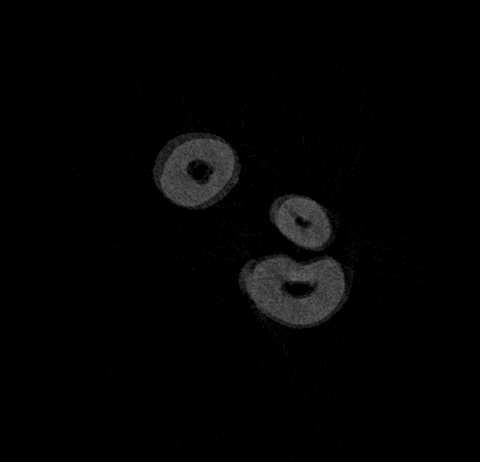

Supplement: S2 File — (ZIP) [file pone.0209698.s002.zip › Self-Assembled micro-CT/Self-Assembled micro CT_0131.tif]

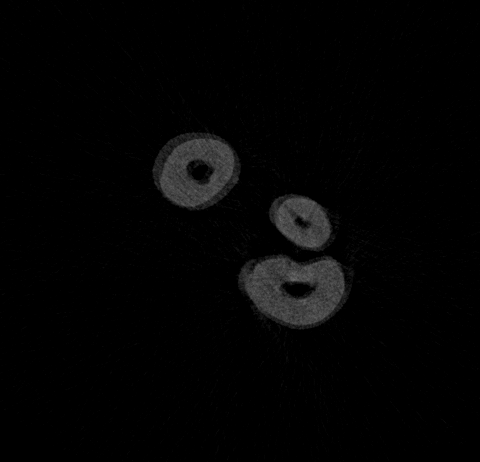

Supplement: S2 File — (ZIP) [file pone.0209698.s002.zip › Self-Assembled micro-CT/Self-Assembled micro CT_0132.tif]

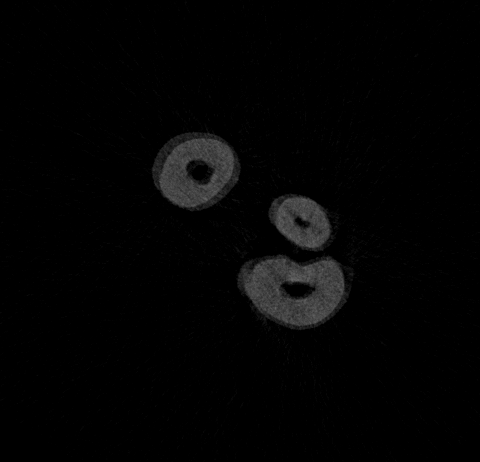

Supplement: S2 File — (ZIP) [file pone.0209698.s002.zip › Self-Assembled micro-CT/Self-Assembled micro CT_0133.tif]

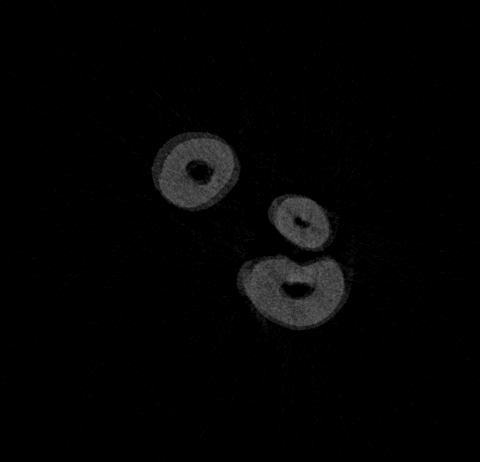

Supplement: S2 File — (ZIP) [file pone.0209698.s002.zip › Self-Assembled micro-CT/Self-Assembled micro CT_0134.tif]

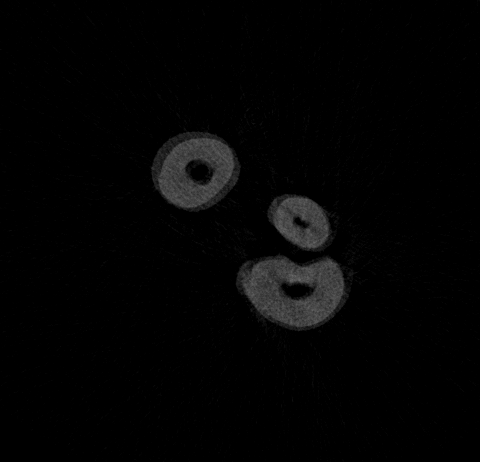

Supplement: S2 File — (ZIP) [file pone.0209698.s002.zip › Self-Assembled micro-CT/Self-Assembled micro CT_0135.tif]

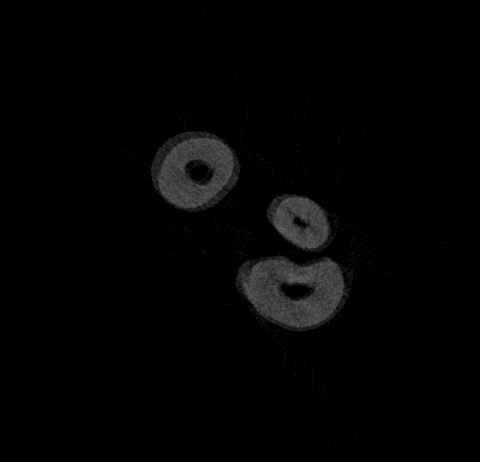

Supplement: S2 File — (ZIP) [file pone.0209698.s002.zip › Self-Assembled micro-CT/Self-Assembled micro CT_0136.tif]

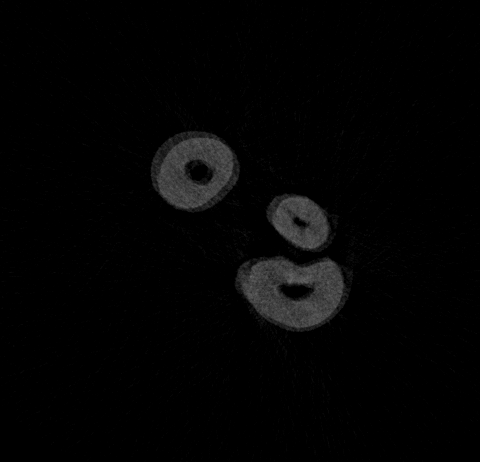

Supplement: S2 File — (ZIP) [file pone.0209698.s002.zip › Self-Assembled micro-CT/Self-Assembled micro CT_0137.tif]

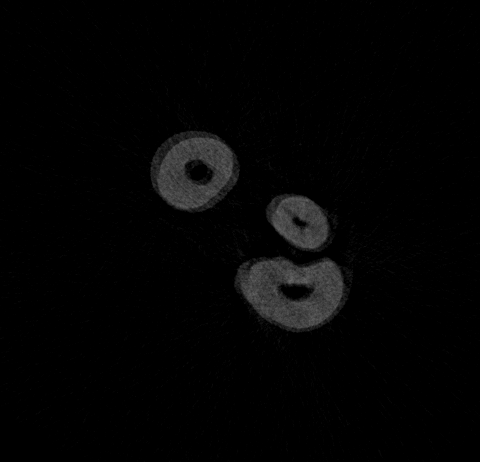

Supplement: S2 File — (ZIP) [file pone.0209698.s002.zip › Self-Assembled micro-CT/Self-Assembled micro CT_0138.tif]

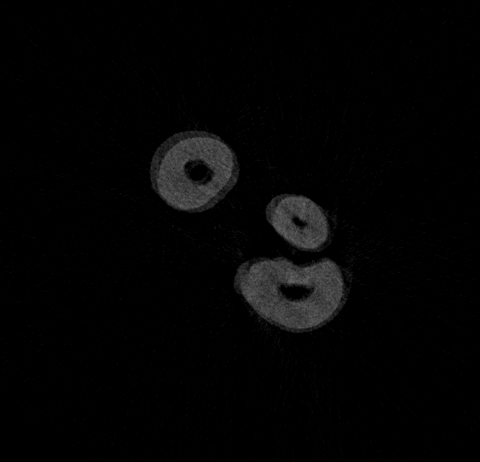

Supplement: S2 File — (ZIP) [file pone.0209698.s002.zip › Self-Assembled micro-CT/Self-Assembled micro CT_0139.tif]

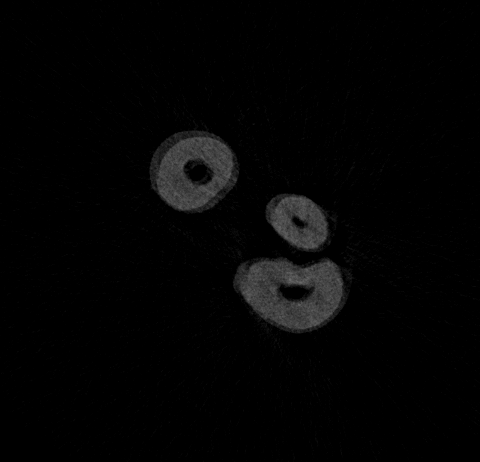

Supplement: S2 File — (ZIP) [file pone.0209698.s002.zip › Self-Assembled micro-CT/Self-Assembled micro CT_0140.tif]

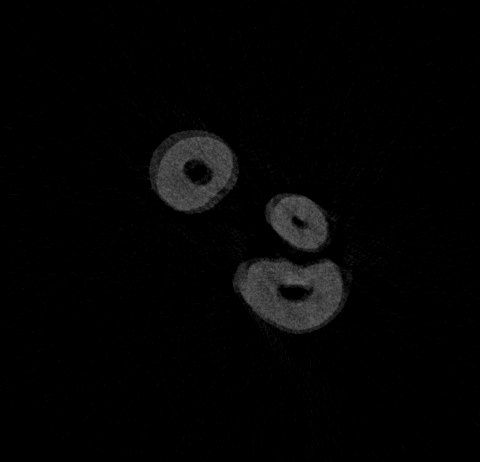

Supplement: S2 File — (ZIP) [file pone.0209698.s002.zip › Self-Assembled micro-CT/Self-Assembled micro CT_0141.tif]

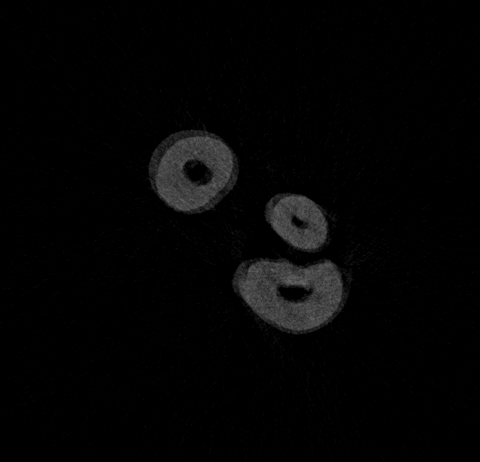

Supplement: S2 File — (ZIP) [file pone.0209698.s002.zip › Self-Assembled micro-CT/Self-Assembled micro CT_0142.tif]

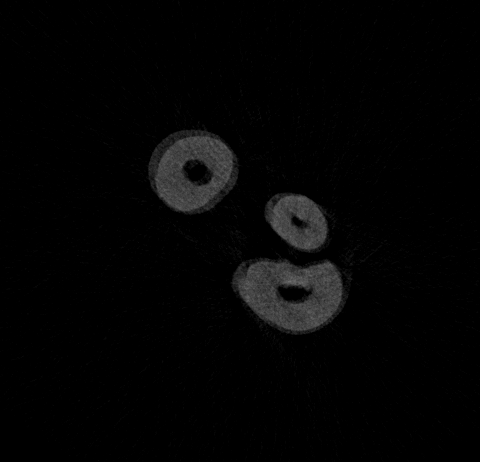

Supplement: S2 File — (ZIP) [file pone.0209698.s002.zip › Self-Assembled micro-CT/Self-Assembled micro CT_0143.tif]

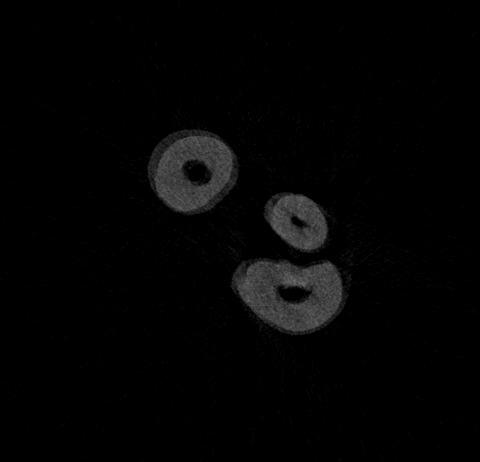

Supplement: S2 File — (ZIP) [file pone.0209698.s002.zip › Self-Assembled micro-CT/Self-Assembled micro CT_0144.tif]

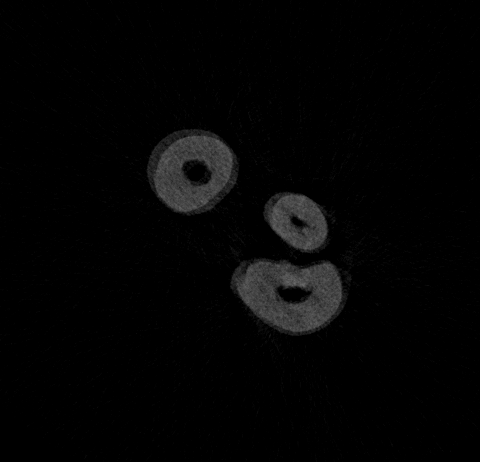

Supplement: S2 File — (ZIP) [file pone.0209698.s002.zip › Self-Assembled micro-CT/Self-Assembled micro CT_0145.tif]

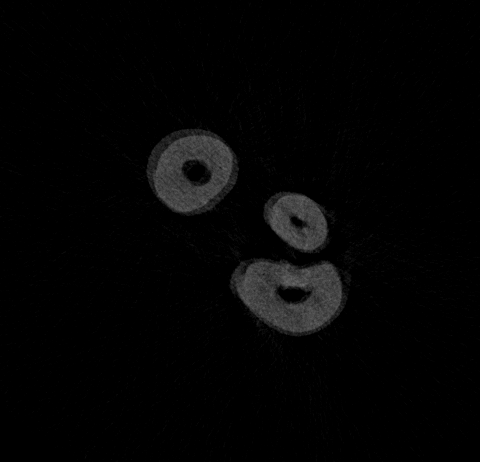

Supplement: S2 File — (ZIP) [file pone.0209698.s002.zip › Self-Assembled micro-CT/Self-Assembled micro CT_0146.tif]

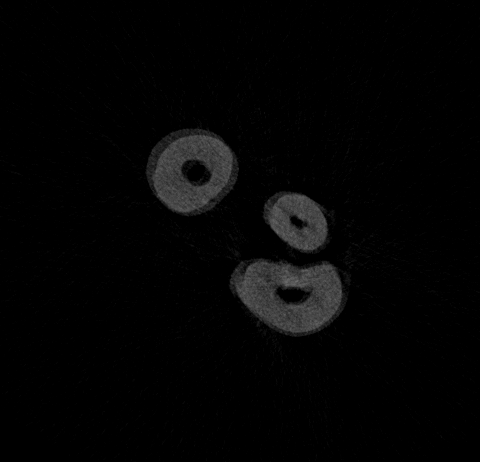

Supplement: S2 File — (ZIP) [file pone.0209698.s002.zip › Self-Assembled micro-CT/Self-Assembled micro CT_0147.tif]

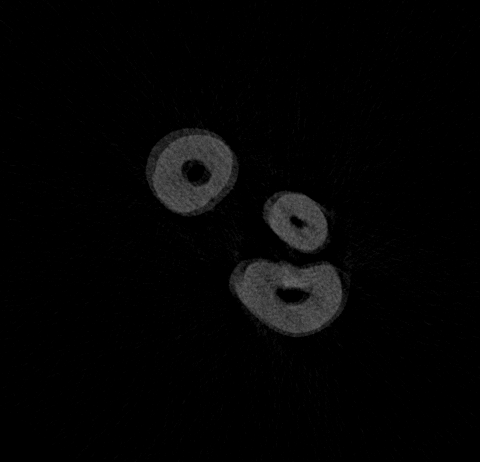

Supplement: S2 File — (ZIP) [file pone.0209698.s002.zip › Self-Assembled micro-CT/Self-Assembled micro CT_0148.tif]

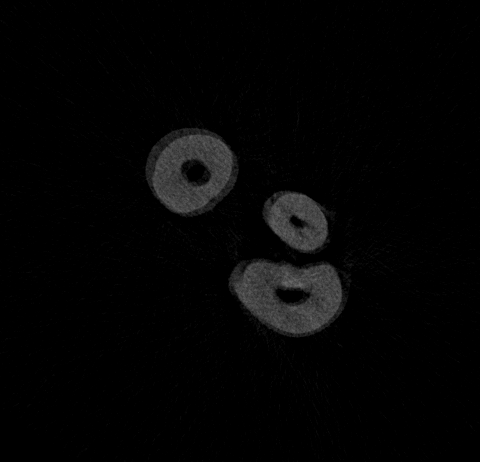

Supplement: S2 File — (ZIP) [file pone.0209698.s002.zip › Self-Assembled micro-CT/Self-Assembled micro CT_0149.tif]

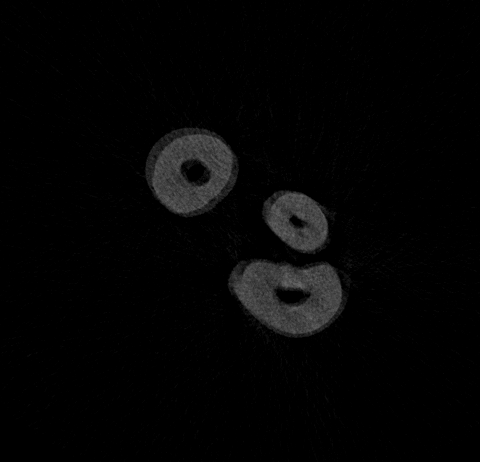

Supplement: S2 File — (ZIP) [file pone.0209698.s002.zip › Self-Assembled micro-CT/Self-Assembled micro CT_0150.tif]

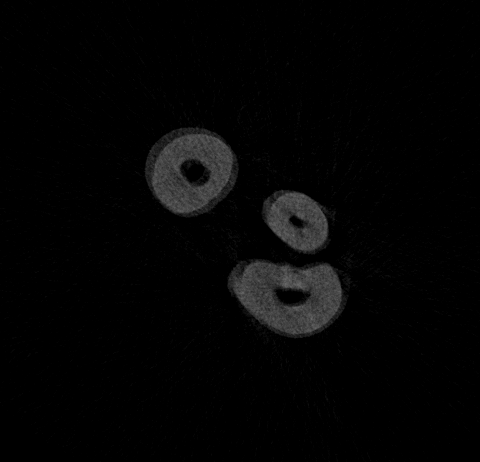

Supplement: S2 File — (ZIP) [file pone.0209698.s002.zip › Self-Assembled micro-CT/Self-Assembled micro CT_0151.tif]
